# Metabolic, mitochondrial, renal and hepatic safety of enfuvirtide and raltegravir antiretroviral administration: Randomized crossover clinical trial in healthy volunteers

**DOI:** 10.1371/journal.pone.0216712

**Published:** 2019-05-23

**Authors:** Sergio Barroso, Constanza Morén, Àlex González-Segura, Neus Riba, Joan A. Arnaiz, Marcela Manriquez, Gemina Santana, José L. Blanco, María Larousse, Montse Loncà, Elisa de Lazzari, Jaume Llopis, Josep Mallolas, Oscar Miró, Xavier Carné, Jose M. Gatell, Glòria Garrabou, Esteban Martínez

**Affiliations:** 1 Muscle Research and Mitochondrial Function Laboratory, Cellex-IDIBAPS, Faculty of Medicine and Health Sciences, University of Barcelona, Barcelona, Spain; 2 Internal Medicine Department—Hospital Clínic of Barcelona (HCB), Barcelona, Spain; 3 Biomedical Research Networking Centres of Rare Diseases CIBERER (ISCIII), Barcelona, Spain; 4 Pompeu Fabra University, Barcelona, Spain; 5 Autonomous University of Barcelona, Barcelona, Spain; 6 Phase I Unit, Clinical Pharmacology Department, HCB, Barcelona, Spain; 7 Infectious Diseases Department, HCB, Barcelona, Spain; 8 Department of Statistics, University of Barcelona, Barcelona, Spain; 9 Emergency Department, HCB, Barcelona, Spain; Imperial College London, UNITED KINGDOM

## Abstract

**Context:**

Classical antiretroviral agents may acutely impact on metabolic, mitochondrial, renal and hepatic function in HIV-infected and uninfected persons. Fusion and integrase inhibitors are supposed to be safer, but have been scarcely investigated. To avoid any interference with HIV or other antiretrovirals, we assessed markers of these toxicities in healthy adult volunteers treated with Enfuvirtide (T20) or Raltegravir (RAL).

**Methods:**

Twenty-six healthy participants were randomized to T20/90mg vs. placebo (n = 12) or RAL/400mg vs. placebo (n = 14) every 12h in two 7-day periods separated by a 4-week washout period. Major end-points were changes in lipid profile (total cholesterol, high-density-lipoprotein (HDL)-cholesterol, low-density-lipoprotein (LDL)-cholesterol, triglycerides), insulin resistance (glucose) and mitochondrial toxicity (mitochondrial DNA content–mtDNA–in peripheral blood mononuclear cells). Renal and hepatic toxicity (creatinine, alanine transaminase (AST), alanine aminotransferase (ALT), bilirubin and total plasma proteins) and overall safety were also analysed. Effect of period, treatment, and basal measures were evaluated for each end-point.

**Results:**

Neither T20-administration nor RAL-administration yielded to any statistic significant change in the markers of metabolic, mitochondrial, renal or hepatic toxicity assessed. No symptoms indicative of drug toxicity were neither found in any subject.

**Conclusions:**

In absence of HIV infection, or concomitant treatment, short-term exposure to T20 or RAL in healthy adult volunteers did not lead to any indicative changes in toxicity markers thus presuming the safe profile of both drugs.

## Introduction

A combination of two nucleoside reverse transcriptase inhibitors (NRTIs) plus either a boosted protease inhibitor (PI) or a non-nucleoside reverse transcriptase inhibitor (NNRTI) has constituted the standard highly active antiretroviral treatment (HAART) for many years [1–4]. Despite harbouring validated therapeutic efficacy, some of these drugs as the virus itself have been associated with early metabolic, mitochondrial, renal and hepatic toxicity that may induce late organ-specific diseases including hyperlipidaemia, hyperlactatemia, insulin resistance and lipoatrophy [4–11].These alterations may result in an increased risk for cardiovascular disease, together with renal or hepatic disease [5,7,10,12]. Adverse clinical effects of PIs, NRTIs and NNRTIs have been associated with secondary interactions of these drugs with molecular pathways essential for cell function. Off-target molecular effects include, in case of PI drugs, interaction with cell proteins such as cellular retinoic acid binding protein 1 or lipoprotein receptor-related protein, responsible for alterations in lipid profile leading to metabolic syndrome, lipodystrophy and hepatic steatosis [13–16]. Moreover, PIs interact with GLUT1 and GLUT4 glucose transporters, responsible for glucose profile disturbances and insulin resistance [6,9]. Secondary adverse molecular effects of NRTIs drugs include interaction with γDNA-polymerase, the mitochondrial enzyme devoted to the replication and repair of the mitochondrial DNA (mtDNA) [7,17–19], leading to hyperlactatemia, lactic acidosis, myopathy, renal or hepatic disease. On the other hand, cell toxicity of NNRTIs has been associated to potential apoptotic events (controversial) [20,21], alterations in lymphocytes T proliferation [22], lipid differentiation via sterol regulatory element-binding protein 1c pathway [23,24], and clinically associated to mild hepatoxicity [25]. Consequently, metabolic, mitochondrial, renal and hepatic biomarkers have been implemented in clinical and research settings for early detection and follow up of HAART toxicity. In an effort to decrease the risk of associated toxicities, safer PIs, NRTIs and NNRTIs are constantly have been being developed [1,2]. Furthermore, drugs from alternative families such as fusion, entry or integrase inhibitors have become clinically available. They may offer, not only offers potential alternative options for patients with multiple resistances to standard antiretroviral drugs, but also safer therapeutic options.

Enfuvirtide (T20) is the first entry inhibitor available for salvage combination therapy [26,27]. Local reactions at the injection site is the most common adverse effect [28]. Despite T20 is falling into disuse and often limited to rescue therapies, novel similar formulations may emerge in this family of antiretrovirals, thus supporting the rationale of this study.

On the other hand, raltegravir (RAL) is the first integrase inhibitor available [29]. The most frequently reported adverse effects in clinical trials were headache, nausea and abdominal pain. New studies have shown that this drug can be safely administrated during pregnancy [30], in contrast with other drugs such zidovudine that were once recommended [31].

Controversially, post-marketing data have recently raised concerns on the safety of these new antiretroviral drugs. Interestingly, this is the case for RAL and other integrase inhibitors, as associated weight gain has been reported in cohort studies [32].

Importantly, for both RAL and T20 medications there are no studies assessing changes in insulin resistance and mtDNA parameters (exclusively in lipid, renal and hepatic markers) and, in all cases, all were assessed in concomitant HAART schedules, after drug discontinuation, or in the presence of HIV infection acting as a confounder [33,34].

Although the target and the chemical structure of both T20 and RAL are different from those classical antiretrovirals, it is unknown whether T20 and/or RAL have any early effect in metabolic, mitochondrial, renal and hepatic toxicity markers. All these reasons provided the basis for performing post-commercialization Phase I studies of T20 and RAL administration in healthy volunteers to assess univocal causality of drug safety/toxicity without the interference of previous/concomitant medication or HIV infection [35]. We designed a prospective randomized crossover clinical trial to assess whether a short period of mono-therapy with T20 or RAL may have any early metabolic, mitochondrial, renal and hepatic impact in healthy adult volunteers.

## Materials and methods

### Subjects

The present work includes two different studies to assess T20 and RAL toxicity performed at the Hospital Clinic of Barcelona (Barcelona, Spain) at different time periods. Clinical Research Ethics Committee of our hospital approved T20 and RAL studies in June 2005 and November 2008, respectively. Potential candidates were recruited in 2006 for T20 and 2011 for RAL, following Helsinki guidelines. Ongoing and related trials for this drug/intervention are registered. Since both studies were performed at different timing, they were subjected to different legal registry requirements. According to legal obligations, in 2006 we performed the registry of T20 study (14th April 2008) after patient inclusion (March 2006). Conversely, by the time we performed the inclusion of patients for RAL trial (March 2011), we were required to prior register the study (15^th^ October 2008). In all cases specific legal and institutional requirements at the time of the studies were followed.

Eligible volunteers were required to be healthy males between 18 and 45 years, with normal blood biochemical parameters, BMI between 19–24.9 kg/m^2^, no active concomitant clinical conditions, negative HIV, hepatitis B and C serologies or drug urine test. Exclusion criteria were: prior psychiatric illness, dyslipidaemia, alcohol consumption >30g/day, caffeine consumption >5 units/day, current smoking, known drug allergies, participation in other drug trials in the previous 3 months or no medications in the previous 30 days. Written informed consent was obtained from all volunteers before selection visit. Volunteers were randomized in a 1:1 mode to receive one of the following sequences: T20-placebo (n = 6) vs. placebo-T20 (n = 6) or RAL-placebo (n = 7) vs. placebo-RAL (n = 7). The study was performed at the Phase I Unit of Hospital Clínic (Barcelona, Spain).

#### Ethical approval and informed consent

All procedures performed in studies involving human participants were in accordance with the ethical standards of the institutional and/or national research committee and with the 1964 Helsinki declaration and its later amendments or comparable ethical standards.

Informed consent was obtained from all individual participants included in the study.

### Design

The study was designed as a single-centre, randomized, cross-over, double-blind, placebo-controlled trial in healthy adult volunteers (clinicaltrial.gov identifier: NCT00657761 for T20 and NCT00772720 for RAL). Randomization was performed with the Glaxo software: MAS V 2.1 Glaxo Wellcome 2001 by the CTU (Clinical Trials Unit) of the Clinical Pharmacology Service of the Hospital Clínic of Barcelona. The Trials Agency of the Hospital Clínic was provided by the CTU with the random assignment list so that they can proceed with the preparation of the medication for the study, which was administered following a double-blind scheme. It was also provided, in a sealed envelope, with the code of random assignment of each participant to the Principal Investigator.

**Fig 1:**
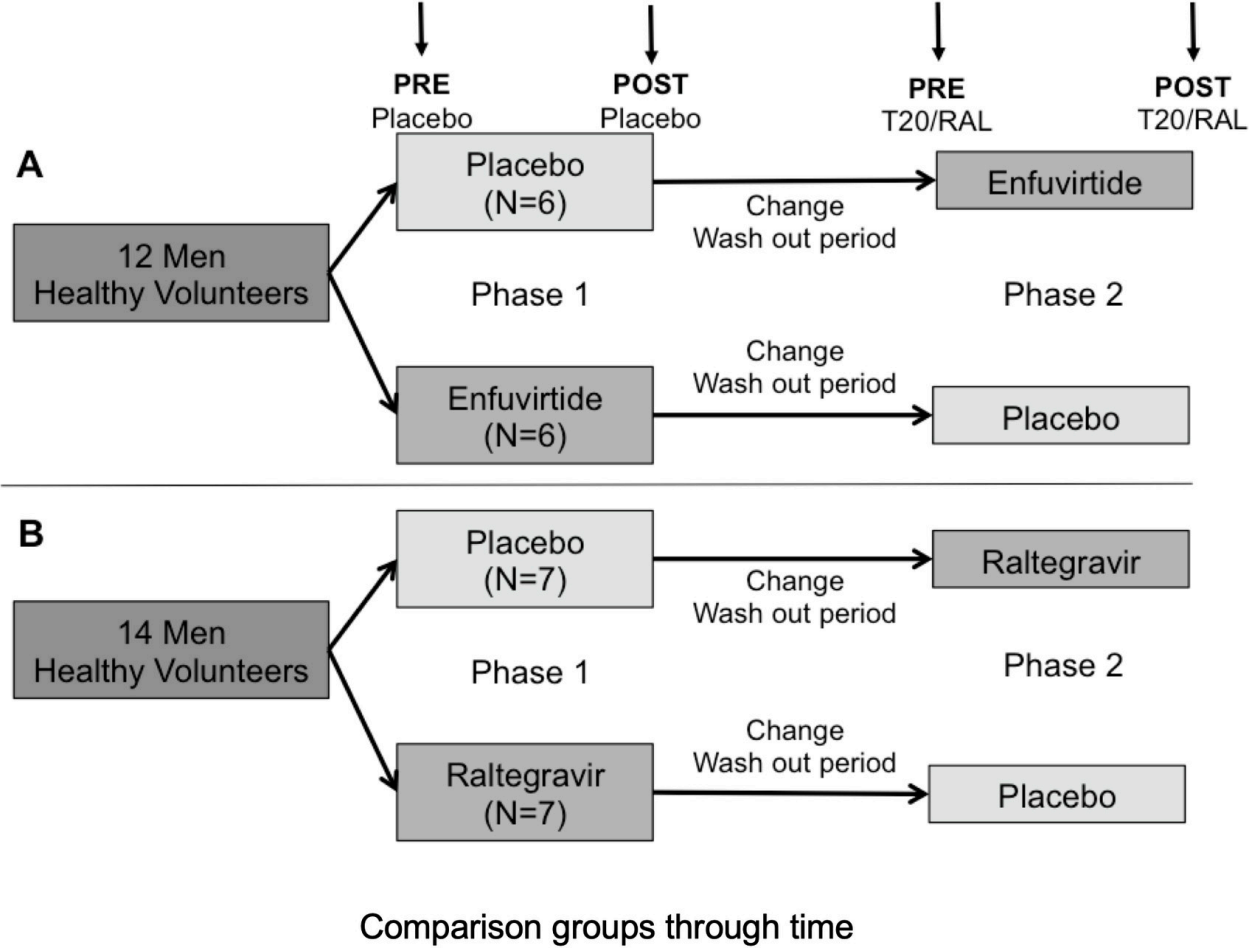
**A: Flow diagram of enfuvirtide crossover.** From a total of 12 healthy volunteers; 50% were randomized to placebo/enfuviride sequence. **B: Flow diagram of raltegravir crossover.** From a total of 14 healthy volunteers; 50% were randomized to placebo/raltegravir sequence. Blood samples (upper arrows) were obtained before (PRE) and after (POST) both phases. Each phase was 1 week long, separated from each other by a washout period of four weeks. Volunteers started with phase 1 trial (placebo/treatment) followed by a washout period and posterior phase 2 (treatment/placebo, according to the first made intervention).

In order to balance all patients into comparable basal conditions and avoid food interferences, participants were instructed to follow a specific diet within the World Health Organization standards one week prior to the initiation of the trial and during the periods of medication administration. A simple randomization was performed. For both T20/RAL studies, volunteers were scheduled to a 1-week period of either T20/RAL or placebo followed by a 4-weeks washout period and finally a 1-week period of placebo or T20/RAL, respectively. Washout period was set in order to avoid carry-over effect [36]. Fig 1 also summarises the design of the trial.

### Study medication

T20 (Fuzeon, Hoffmann-La Roche AG, Germany) was subcutaneously administered at 90 mg/mL doses. As placebo we used 0.9% saline solution (Bieffe Medital Sabiñánigo, Spain). Both T20 and placebo were prepared for administration at the hospital pharmacy. Subcutaneous syringes with either 1mL of T20 or placebo were wrapped with aluminium foil for blinding and kept in a temperature-controlled refrigerator for a maximum of 24 hours.

RAL, formulated as Isentress (Merck Sharp & Dohme Laboratories, Spain), with 400 mg of active substances and 26.06 mg of lactose-monohydrate. Placebo was also formulated for oral intake (Merck Sharp & Dohme).

Medication was administrated every 12 hours for 7 consecutive days. All medication was administered by one of the study investigators (NR, MM, GS) at the Phase I Unit, and volunteers were directly monitored for potential immediate adverse effects for at least one hour after subcutaneous administration. Volunteers were routinely asked about potential adverse events prior to each dose of study medication.

### Outcomes

Major end-points were changes in lipid profile (total cholesterol, high density lipoprotein (HDL)-cholesterol, low density lipoprotein (LDL)-cholesterol, triglycerides), insulin resistance (glucose) and mitochondrial toxicity (peripheral blood mononuclear cells -PBMC–mtDNA content). Renal and hepatic toxicity markers (creatinine, aspartate aminotransferase (AST), alanine aminotransferase (ALT), bilirubin and total plasma proteins were also analysed. Moreover, data regarding overall safety was recruited. The effects of period, sequence (carry-over), and treatment were evaluated for each end-point from absolute values and results were expressed as percentage of change.

### Metabolic and mitochondrial toxicity assessment

Venous blood sampling was withdrawn in the morning after at least 12-hours overnight fasting at the beginning and the end of each 1-week period (Fig 1). A urine sample for detection control of abuse drugs (alcohol, amphetamine, cocaine, cannabis, methadone, barbiturates or benzodiazepine) was collected at the time of each blood sampling.

Blood samples were tested for metabolic markers of lipid metabolism and insulin resistance toxicity. Total cholesterol and triglycerides levels were determined using enzymatic reagents (Trinder, Bayer Diagnostics, NY) for a Cobas Mira automated analyser (Hoffmann-LaRoche, Switzerland). The HDL-cholesterol level was determined after precipitation with phosphotungstic acid and magnesium chloride, LDL-cholesterol was directly measured. Plasma glucose was measured by the glucose hexokinase method (IRMA, Biosource Diagnostics, Belgium).

MtDNA content was measured in PBMCs as a marker of mitochondrial toxicity. PBMC were isolated by Ficoll-Histopaque technique and stored at -80 ± 5°C until analysis. MtDNA was obtained by standard phenol-chloroform extraction and amplified by quantitative real time PCR (LightCycler FastStart DNA Master SYBR Green I, Roche, Germany). Results were expressed as mean ratio between ND2-mitochondrial-encoded gene respect to 18SrRNA nuclear-encoded gene copies [37].

### Renal and hepatic toxicity markers

Creatinine levels were measured using high-resolution liquid chromatography with ultraviolet-visible detection in the concentration range of 0.1 g/l urine 4.0 g/l urine. Transaminases values were determined using the malate dehydrogenase (MDH)-NADH reaction measured at 340 nm (BioSystems S.A., Spain). Bilirubin determination was made by the diazotized sulfanilic acid reaction and posterior photocolorimetry measured at 530 nm. Total plasma proteins were measured according to the Biuret method.

### Safety analysis

Safety end-points included abnormal laboratory results or any clinical adverse events. The adverse events were graded according to the World Health Organization score. Injection site reactions were assessed according to persistent pain and discomfort at local level, size of the indurations and erythema.

### Statistical analysis

Sample size was calculated based on total cholesterol measurement, established as one of the major endpoints of the study.

According to our previous experience, as well as other investigations [38], the intra-individual variability of fasting normal plasma total cholesterol was expected to be lower than 10% in both T20 and RAL administration. Therefore, we assumed that an increase of levels equal to or over 25 mg/dl of the total cholesterol in healthy subjects with normal plasma values would be considered as an evidence of a real increase of lipid profile toxicity. The formula used to determine the sample size n was: n = (Z_α_+Z_β_)^2^*S^2^/d^2^, where Z_α_ = 1.64 (one-sided α = 0.025), Z_β_ = 0.84 where the effect size *d* was set to 0.07 and the variability *S* to 0.10. Thus, we assumed a minimum sample size per group of 12 volunteers to detect significant increases in fasting plasma total cholesterol.

Due to the small sample size, non-parametric test (Wilcoxon test for paired data) was used to evaluate the effects of the period, treatment (differences between Phase 1 and Phase 2), as well as any potential differences in the basal measurements (analysing differences between pre-Phase 1 and pre-Phase 2). Results were expressed as mean values or percentage of change (final endpoint minus baseline measurement normalised by baseline value). To assess the direction and the magnitude of change and variation of results, Standard Error of the Mean (SEM) values were used. All statistical tests were performed by the Statistical Package of Social Sciences (IBM Corp. Released 2011. IBM SPSS Statistics for Windows, Version 22.0, NY: IBM Corp.). Significance level was set at p value of 0.05.

According to the lack of legal or ethical restrictions for data sharing, we have submitted the anonymous data of both clinical trials into the University of Barcelona repository (http://hdl.handle.net/2445/131036).

## Results

### Study population

Twelve and 14 subjects completed T20 and RAL therapeutic schedule respectively, with 100% of adherence. Subjects were all men with a mean age of 29.2 ± 1.27 for T20, and 23.3 ± 1.52 for RAL. Serologies for HIV and hepatitis B and C, and screening for abuse drugs were negative for all volunteers that completed the study.

### Outcomes

#### Enfuvirtide results

**Fig 2:**
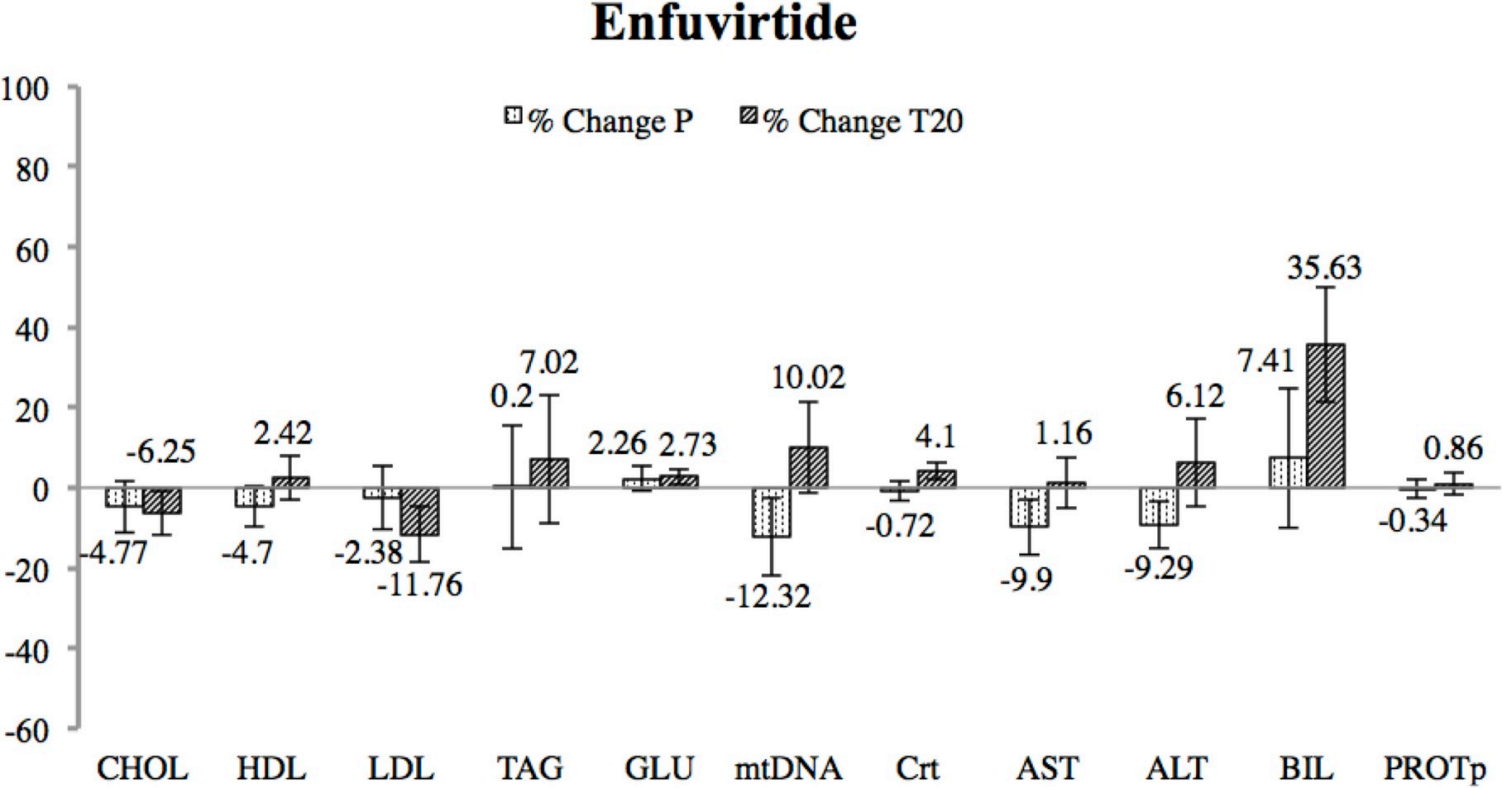
Percentage of change between final and baseline measurements for enfuvirtide (T20) and placebo interventions. Significance was assessed through a Wilcoxon test. Variation is represented with error bars through the SEM value. No significant change was detected after T20 treatment.

Mean values of each metabolic, mitochondrial, renal and hepatic marker are shown in Table 1 and represented in Fig 2 as percentage of change. Data showed no significant changes after T20 treatment.

**Table 1:** Enfuvirtide (T20 Wilcoxon test results). n = 12 Volunteers; all men; mean age 29.2 ± 1.27. Significance was calculated among absolute values. "p value of change" represents the significance between endpoints after Phase 1 and Phase 2. "p value PREs" represents significance between values in basal circumstances. No changes were significant.

Parameters	CHOL	HDL	LDL	TAG	GLU	mtDNA	Crt	AST	ALT	BIL	PROTp
**Mean** **PRE Placebo**	181.83 ± 10.9	50.45 ± 2.8	110.64 ± 7.6	85.08 ± 14.6	81.00 ± 2.4	0.713 ± 0.08	1.15 ± 0.03	26.08 ± 2.8	23.33 ± 1.5	0.90 ± 0.10	74.50 ± 2.0
**Mean** **POST Placebo**	173.17 ± 11.6	48.08 ± 2.6	108.00 ± 8.9	85.25 ± 13.1	82.83 ± 2.4	0.625 ± 0.07	1.14 ± 0.03	23.50 ± 1.8	21.17 ± 1.3	0.97 ± 0.16	74.25 ± 1.8
**Mean** **PRE T20**	183.92 ± 13.5	48.25 ± 2.2	118.33 ± 11.4	86.67 ± 10.8	82.50 ± 2.0	0.620 ± 0.05	1.11 ± 0.03	23.45 ± 1.9	22.27 ± 2.4	0.79 ± 0.05	74.27 ± 1.9
**Mean** **POST T20**	172.42 ± 10.1	49.42 ± 2.6	104.42 ± 8.1	92.75 ± 13.8	84.75 ± 1.7	0.682 ± 0.07	1.15 ± 0.02	23.73 ± 1.5	23.64 ± 2.5	1.07 ± 0.12	74.91 ± 2.1
**Change** **Placebo (%)**	−4.77 ± 6.4	−4.70 ± 5.2	−2.38 ± 8.0	0.20 ± 15.4	2.26 ± 3.0	−12.32 ± 9.6	−0.72 ± 2.5	−9.90 ± 6.8	−9.29 ± 5.8	7.41 ± 17.4	−0.34 ± 2.4
**Change** **T20 (%)**	−6.25 ± 5.5	2.42 ± 5.4	−11.76 ± 6.9	7.02 ± 15.9	2.73 ± 2.0	10.02 ± 11.3	4.10 ± 2.1	1.16 ± 6.3	6.12 ± 10.9	35.63 ± 14.3	0.86 ± 2.7
**p value** **change**	0.937	0.200	0.798	0.695	0.656	0.060	0.194	0.722	0.192	0.349	0.473
**p value** **PREs**	0.695	0.075	0.211	0.784	0.594	0.239	0.334	0.893	0.422	0.177	0.503

CHOL = Total cholesterol; HDL = High density lipoproteins; LDL = Low density lipoproteins; TAG = Triglycerides; GLU = Glucose; mtDNA = Mitochondrial DNA; Crt = Creatinine; AST = Aspartate aminotransferase; ALT = Alanine transferase; BIL = Bilirubin; PROTp = Total plasma proteins; PRE = Values before starting placebo/T20; POST = Values after starting placebo/T20.

#### Raltegravir results

**Fig 3:**
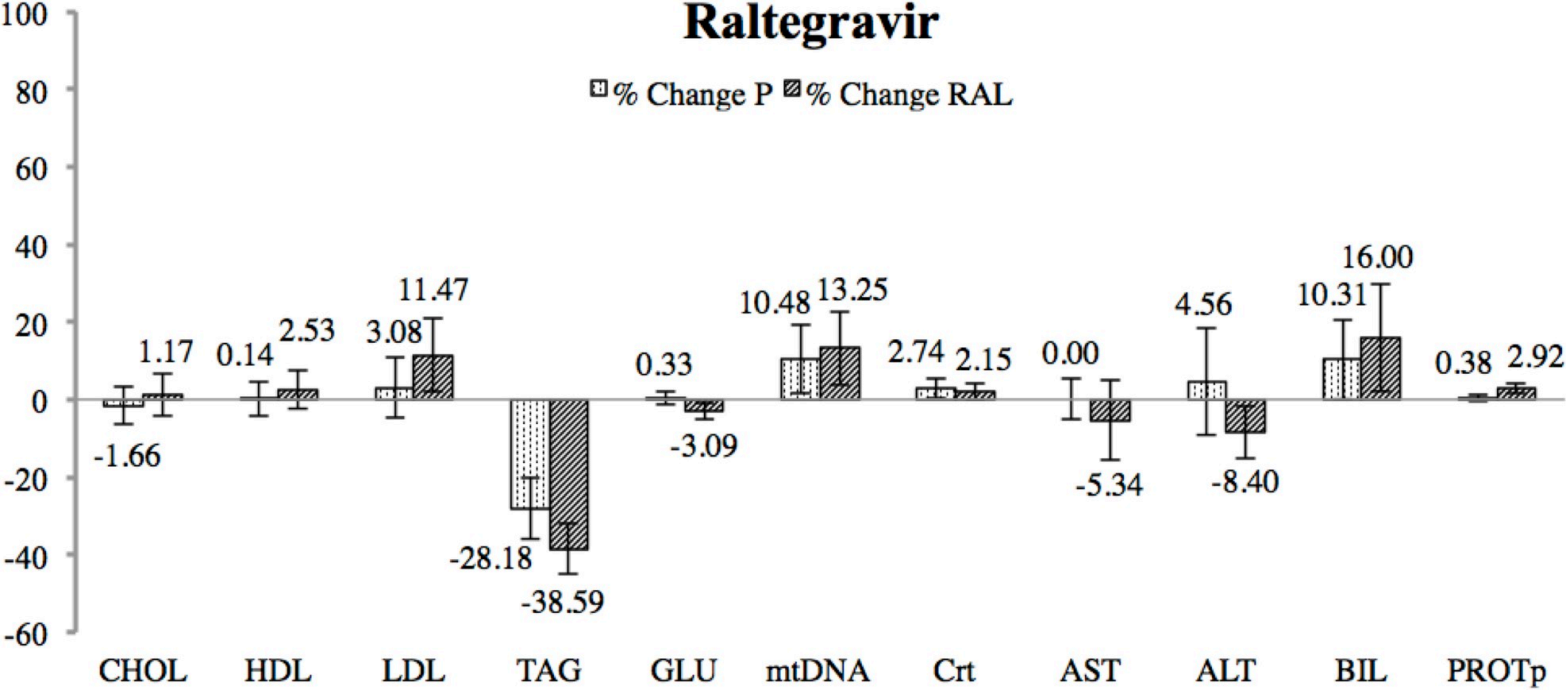
Percentage of change between final and baseline measurements for raltegravir (RAL) and placebo interventions. Significance was assessed through a Wilcoxon test. Variation is represented with error bars through the SEM value. No significant change was detected after T20 treatment.

Mean values of each metabolic, mitochondrial, renal and hepatic outcome are shown in Table 2 and in Fig 3 as percentage of change. Data showed no significant changes after RAL treatment.

**Table 2:** Raltegravir (RAL Wilcoxon test results). n = 14 Volunteers; all men; mean age 23.3 ± 1.52. Significance was calculated among absolute values. "p value of change" represents the significance between endpoints after Phase 1 and Phase 2. "p value PREs" represents significance between values in basal circumstances. No changes were significant.

Parameters	CHOL	HDL	LDL	TAG	GLU	mtDNA	Crt	AST	ALT	BIL	PROTp
**Mean** **PRE Placebo**	155.29 ± 7.9	50.86 ± 2.5	85.79 ± 6.4	92.79 ± 14.5	87.43 ± 2.2	1.747 ± 0.15	0.98 ± 0.02	23.93 ± 1.2	25.07 ± 2.5	0.69 ± 0.1	74.71 ± 0.8
**Mean** **POST Placebo**	152.71 ± 7.6	50.93 ± 2.3	88.43 ± 6.8	66.64 ± 7.2	87.71 ± 1.4	1.917 ± 0.16	1.02 ± 0.03	23.93 ± 1.3	26.21 ± 3.4	0.76 ± 0.1	75.00 ± 0.6
**Mean** **PRE RAL**	152.57 ± 7.7	50.79 ± 2.6	79.71 ± 5.6	110.14 ± 22.3	87.79 ± 1.5	1.762 ± 0.17	1.00 ± 0.02	28.07 ± 3.4	28.07 ± 4.1	0.71 ± 0.1	73.29 ± 0.9
**Mean** **POST RAL**	154.36 ± 8.3	52.07 ± 2.5	88.86 ± 7.6	67.64 ± 7.2	85.07 ± 1.8	1.996 ± 0.17	1.02 ± 0.02	26.57 ± 2.9	25.71 ± 1.9	0.83 ± 0.1	75.43 ± 1.0
**Change** **Placebo (%)**	−1.66 ± 4.9	0.14 ± 4.5	3.08 ± 7.9	−28.18 ± 7.8	0.33 ± 1.6	10.48 ± 8.9	2.74 ± 2.5	0.00 ± 5.2	4.56 ± 13.7	10.31 ± 10.2	0.38 ± 0.8
**Change** **RAL (%)**	1.17 ± 5.4	2.53 ± 4.9	11.47 ± 9.5	−38.59 ± 6.5	−3.09 ± 2.0	13.25 ± 9.5	2.15 ± 2.2	−5.34 ± 10.4	−8.40 ± 6.8	16.00 ± 13.9	2.92 ± 1.3
**p value** **change**	0.414	0.472	0.223	0.397	0.157	0.683	0.693	0.593	0.345	0.929	0.086
**p value** **PREs**	0.366	0.779	0.220	0.510	0.972	0.975	0.925	0.659	0.562	0.549	0.170

CHOL = Total cholesterol; HDL = High density lipoproteins; LDL = Low density lipoproteins; TAG = Triglycerides; GLU = Glucose; mtDNA = Mitochondrial DNA; Crt = Creatinine; AST = Aspartate aminotransferase; ALT = Alanine transferase; BIL = Bilirubin; PROTp = Total plasma proteins; PRE = Values before starting placebo/RAL; POST = Values after starting placebo/RAL.

#### Overall safety

Adverse events were only reported during the T20 study (88%) mainly mild injection site reactions. 12% had other than injection site reactions, including headache, odinophagia and other minor flu-like symptoms. None of these events required any intervention or led to drug discontinuation.

## Discussion

Metabolic, mitochondrial, renal and hepatic toxicity together with overall clinical safety issues have been attributed to antiretrovirals used for HIV management, including PIs, NRTIs and NNRTIs, due to off target interferences with physiological pathways [6,8,9,16,39–41].

Entry and integrase inhibitors are supposed to be safer and free of these toxicities, however there is lack of evidence of *in vivo* studies [21]. Additionally, some safety concerns have been recently raised on RAL administration, as weight gain issues [32]. Moreover, there were no studies assessing changes in insulin resistance and mtDNA parameters (exclusively in lipid, renal and hepatic markers) [33,34] for either RAL or T20 medications. Furthermore, despite T20 is falling into disuse and often limited for rescue therapies, novel formulations may emerge in this family of antiretrovirals, giving sense to the rationale of this study. Thus, this clinical trial may be relevant in assessing potential toxicity of T20 and RAL antiretrovirals in healthy volunteers. Additionally, the design of this study allows testing antiretroviral toxicity in human subjects in the absence of HIV infection, previously associated with metabolic and mitochondrial toxicity [42], or previous or concomitant antiretroviral experience, thus avoiding any confounders of causal toxicity [43].

In the present study, there were no changes indicative of lipid disturbances, insulin resistance, hepatic or renal toxicity were evident after T20 or RAL administration. Considering mitochondrial toxicity, no significant changes were detected in RAL treatment nor after T20 administration. Given the fact that mitochondrial toxicity and primary mitochondrial disorders are associated with a decrease in mtDNA content, none of these treatments should be associated to potential mitochondrial detrimental side effect that may lead to adverse clinical manifestation [8]. Finally, overall clinical safety was excellent for both treatment interventions, except for local injection site reaction for T20 administration, as reported in HIV-infected patients [16].

Our data are in accordance with registries of adverse-drug reactions in HIV-infected patients suggesting null or minimal toxicity for T20 or RAL [4]. Moreover, a recent review confirms the safety profile of RAL [44].

Importantly, some studies have shown that continuous intravenous administration of T20 may be considered *a priori* in selected patients with extensive viral resistance who are unable or unwilling to inject T20 subcutaneously [45]. Other studies have reported a good tolerance in the co-administration of a NNRTI with integrase inhibitors as elvitegravir/ritonavir or RAL with the fusion inhibitor T20, with no need for dose adjustments [46], suggesting their safety and their value as alternative components of HAART schedules.

Our studies had limitations. First, previous studies have shown the safety profile of both T20 and RAL in HAART experienced patients [47–50] which may enclose findings to clinical settings. However the design of our study allowed to test medication effects without HIV and previous treatment interference as potential confounders, as reported in ANRS-138 EASIER trial [43,51]. Second, adverse clinical outcomes usually need months or even years to develop. However, we assumed that subclinical evidence of metabolic changes toxicity markers of antiretroviral drugs may become apparent after a few days of therapy [13,14,22]. In fact, the metabolic effects of switching high toxic HAART regimens to low toxic schedules have been described in HIV-patients after short-periods of treatment [51,52]. However, we cannot rule out that further toxicity or idiosyncratic effects of these drugs may be developed in longer expositions to T20 or RAL. As these effects are extremely rare, large cohort studies should be taken to detect them. Third, our sample was exclusively composed of males. Nonetheless, in clinical settings this bias may reflect the gender associated differences in prevalence of HIV infection and facilitates the eradication of potential gender interference in observed results [53]. Additionally, gender has been reported to modulate some biological parameters used as toxicity markers [54]. For instance, estrogens have been described to interfere mitochondrial parameters in females [55], consequently, the selective inclusion of males in the present study may circumvent this inconvenience. Finally, T20 is rarely used nowadays, but this data can help in supporting the development of potential new family-related agents, with an easier route of administration that can be considered in further decision-making concerning HIV antiretroviral treatment.

In summary, the administration of short-term therapy with T20 or RAL in healthy volunteers was not presumably associated with either: (i) significant changes in metabolic parameters; (ii) decrease of mtDNA levels suggestive of mitochondrial toxicity; (iii) apparent changes in renal or hepatic toxicity markers and; (iv) significant clinical safety issues other than local injection site reactions in the case of T20 administration.

Further studies with bigger sample size and longer antiretroviral exposure should be conducted to confirm herein reported conclusions and elucidate toxic effects of antiretrovirals without HIV or previous ARV interference. Finally, in clinical settings, the assessment of drug toxicity in the context of concomitant HAART medication and HIV infection should be tested, in parallel, to strengthen conclusions and safety for T20 and RAL administration and offer new therapies free of secondary effects for HIV management.

## Supporting information

S1 File Enfuvirtide protocol. (PDF)

S2 File Raltegravir protocol. (PDF)

S3 File CONSORT checklist. (PDF)

S4 File Enfuvirtide statistics. File containing SPSS output for descriptive data and Wilcoxon test for the differences between PRE values and the change.
(PDF)

S5 File Raltegravir statistics. File containing SPSS output for descriptive data and Wilcoxon test for the differences between PRE values and the change.
(PDF)
