# Supplementary material for: Metabolic, mitochondrial, renal and hepatic safety of enfuvirtide and raltegravir antiretroviral administration: Randomized crossover clinical trial in healthy volunteers
Source: PLoS One. 2019 May 23;14(5):e0216712. doi: 10.1371/journal.pone.0216712 (PMC6532851; doi:10.1371/journal.pone.0216712)
Supplement: S1 File — (PDF) [file pone.0216712.s001.pdf]

Influence of enfuvirtide (Fuzeon®) into lipid and hydrocarbonate metabolism and mitochondrial function in healthy volunteers

CLINICAL TRIAL PROTOCOL

PROTOCOL CODE: ENF/01FD - 05/UF1

(Version 1.1, 19.05.05)
Nº EUDRACT: 2005-002018-39

CONFIDENTIAL

DEVELOPER: Dr. José Mª Gatell Artigas
Servicio de Enfermedades Infecciosas
Hospital Clínic de Barcelona
C/ Villarroel 170
08036 Barcelona

1. SUMMARY

1. Type of application

Phase I clinical trial, pharmacodynamics, with a pharmaceutical specialty already authorized. Nº EUDRACT: 2005-002018-39

2. Developer identification

Dr. José Mª Gatell Artigas
Infectious Disease Service
Hospital Clínic de Barcelona
C/ Villarroel 170
08036 Barcelona

3. Clinical trial tittle

Influence of enfuvirtide (Fuzeon®) into lipid and hydrocarbonate metabolism and mitochondrial function in healthy volunteers.

4. Protocol code

- ENF/01FD - 05/UF1
- Nº EUDRACT: 2005-002018-39

5. Principal investigator

Dr. Neus Riba i Garcia
Clinical pharmacology service - UASP Hospital Clínic de Barcelona
C/ Villarroel 170
08036 Barcelona
Tel: 93 227 55 74
Fax: 93 451 44 38

6. Centers where the study will be conducted

<u>Experimental phase:</u>

Clinical pharmacology service - UASP Hospital Clínic de Barcelona
Stairs 2, Basement, door 2e
C/ Villarroel 170
08036 Barcelona

<u>Analytical phase:</u>

Laboratorio Central del Hospital Clínic: CDB (Centre de Diagnòstic Biomèdic)
Hospital Clínic de Barcelona

C/ Villarroel 170
08036 Barcelona

Statistical analysis:

Elisa de Lazzari
Infectious Diseases Service
Hospital Clínic de Barcelona
C/ Villarroel 170
08036 Barcelona
Tel: 93 227 54 00 / Extension 2790
Fax: 934515424

7. Ethical committees that have approved the trial

Clinical Research Ethics Committee of the Hospital Clínic de Barcelona (CEIC Hospital Clínic).

8. Responsible for monitoring

Dr. Joan Albert Arnaiz
Clinical Trials Unit (CTU). Clinical pharmacology service - UASP
Hospital Clínic de Barcelona
C/ Roselló 138
08036 Barcelona
Tel: 93 227 92 38
Fax: 93 227 98 77

9. Experimental and control drug

Experimental drug:

Fuzeon®: contains Enfuvirtide in vials of 108 mg powder for reconstitution. 1 mL of reconstituted solution contains 90 mg of enfuvirtide. It is administered subcutaneously. Therapeuic group: J05AX07. Other antiretrovirals.
A subcutaneous injection of 1 mL (90 mg enfuvirtide) every 12 hours for 7 days will be administered.

Control drug:

Placebo: Saline serum 0.9 Braun.
Administration of 1 mL of saline, subcutaneously, every 12 hours for 7 days.

10. Cinical trial phase

Clinical trial in phase I in healthy volunteers (Phase IV-I), pharmacodynamics.

11. Main objective

Study the modifications on the lipid and hydrocarbon metabolism, and mitochondrial function that enfuvirtide produces in healthy volunteers

12. Design

Unicentric, in healthy volunteers, double-blind, crossed, placebo-controlled clinical trial with random allocation of treatment sequence.

13. Pathology to study

This study will be conducted in healthy volunteers without concurrent pathology.

14. Main variable

Modifications in plasmatic total cholesterol levels.

15. Study population and number of patients

A total of 14 healthy volunteers will be included. All men.

16. Treatment duration

This trial comprises a total of two periods of 7 days each, separated from each other for a 4-week-period. In each of the periods, one of the two possible treatments (enfuvirtide or placebo) will be administered, with one dose taken subcutaneously every 12 for 7 days.

17. Calendar and expected completion date

Once the authorization of the CEIC and the Spanish Agency for Medication and Healthcare Products (AEMPS) will be received, the trial will be conducted. The experimental phase is schedule to December, 2005. Preliminary results may be informed in December, 2006.

2. INDEX

1. **Summary…………………………..…………………………..2**
2. **Index……………………………..……………………………5**
3. **General information……...…………………………...…………7**
   - 3.1 Identification of the assay……….. ……………………………..7
   - 3.2 Type of clinical trial ……………………………………………..7
   - 3.3 Products description (experimental and control) ……...…………7
   - 3.4 Data relative to the promoter ……………………………...…..8
   - 3.5 Identification of the monitor ……………………………………..8
   - 3.6 Data of the investigators of the assay ……………………………8
   - 3.7 Center where the assay will be conducted ………………………9
   - 3.8 Estimated duration of the test ……………………………………9
4. **Justification and Objectives………………………………………9**
   - 4.1 Justification……………………………………………………9
   - 4.2 Objectives…………………………………………………….11
5. **Type of clinical trial and design…………………………………11**
6. **Selection of subjects……………………………………………11**
   - 6.1 Sample size…………………………………………………...12
   - 6.2 Inclusion criteria ……………………………………………..12
   - 6.3 Exclusion criteria ……………………………………………12
   - 6.4 Diagnostic criteria for pathologies in study ……………………13
   - 6.5 Withdrawals and abandonments ………………………………..13
   - 6.6 Treatment of loss prerandomization ……………………………13
   - 6.7 Recruitment period ……………………………………………..13
7. **Description of the treatment……………………………………..13**
   - 7.1 Dosage, dosage, route of administration and format …………...13
   - 7.2 Assignment of treatment………………………………………..14
   - 7.3 Preparation of the study medication ……………………………14
   - 7.4 Storage and handling …………………………………………...17
   - 7.5 Administration of study medication ……………………………17
   - 7.6 Concurring treatments …………………………………………18
   - 7.7 Assessment of the compliance …………………………………18

8. Development of the assay and evaluation of the response……...18

8.1 Selection of volunteers……………………………………………18
8.2 Study development……………………………………………...19
8.3 Specific rules for volunteers ……………………………………20
8.4 Clinical assessment criteria and evaluation ……………………21

9. Adverse events…………………………..…………………………...21

9.1 Definitions ………………………..……………………………21
9.2 Description ………………………..……………………………22
9.3 Registration ………………………..…………………………...23
9.4 Notification……………………….……………………………23

10. Ethical aspects………………………………………………………23

10.1 General considerations…………….……….. ……………...23
10.2 Information and informed consent……………………………24
10.3 Confidentiality……………………………………………….....24
10.4 Insurance policy…………………………….. ………………..24

11. Practical considerations ……………………...………………..24

11.1 Responsibilities of the participants ………...………………24
11.2 Corrections …………………………...……..………………24
11.3 Storage of the assay documentation…………….………….24
11.4 Conditions of publication ……………………………………..25
11.5 Interruption of the assay ……………………………………25

12. Statistical analysis …………………………..………………...25

12.1 Analysis of the main variable……………………………………25
12.2 Analysis of secondary variables………………………………25

13. **Bibliography…………………………..…………………………..27**

3. GENERAL INFORMATION

3.1 IDENTIFICATION OF THE ASSAY

- **Title:** " Influence of enfuvirtide (Fuzeon®) into lipid and hydrocarbonate metabolism and mitochondrial function in healthy volunteers".
- **Protocol code:** 2005-002018-39 (version 1.1, 19.05.05).
- **No EUDRACT**: 2005-002018-39

3.2. TYPE OF CLINICAL TRIAL

A pharmacodynamic phase I clinical trial with healthy volunteers will be conducted to evaluate the effects of enfuvirtide (authorized pharmaceutical specialty for HIV) on lipid and hydrocarbon metabolism, and mitochondrial function. This is a double-blind, placebo-controlled, masked, cross-over study (two formulations, Fuzeon® and placebo and two treatment periods) with random assignment of the treatment sequence. Both formulations, Fuzeon® and placebo will be administered subcutaneously every 12 hours for 7 days. All participants will receive both treatments (crossed) leaving a washing out period of at least 4 week to avoid a "carry over" effect between them.

3.3. PRODUCTS DESCRIPTION (EXPERIMENTAL AND CONTROL)

A. Generic name, trade name

- **Experimental drug:** Enfuvirtide, Fuzeon®.
- **Control drug**: Physiological saline 0.9% Braun.

B. Quantitative and qualitative composition. Notifiable excipients

- **Experimental drug**: Fuzeon® in 1mL 90 mg of enfuvirtide. Exipients: sodium carbonate, manitol, sodium hydroxide, chlorohydrin acid. Water for the injection.
- **Control drug:** Physiological serum 0.9 Braun: 0.9 g of sodium chloride per 100 mL and water for injection in sufficient quantity.

C. Pharmaceutical form

- **Experimental drug:** Fuzeon® powder and injectable solvent. Every vial contains 108 mg of enfuvirtide. 1 ml of reconstituted solution contains 90 mg of enfuvirtide.

- **Control drug**: Injectable solution

D. Sampling entities

- **Experimental drug**: enfuvirtide (Fuzeon®). Roxhe Pharma Laboratories.
- **Drug control:** Physiological serum 0.9 Braun.

E. Dosage and route of administration

Subcutaneous administration of 90 mg every 12 hours of enfuvirtide or saline for 7 days, respectively in each of the two periods of the study.

3.4. DATA RELATIVE TO THE PROMOTER

Dr. José Mª Gatell Artigas
Head of Infectious Diseases Service
Hospital Clínic de Barcelona
C/Villarroel 170, 08036, Barcelona
Tel .: 93 227 54 30 / Fax: 93 451 44 38

3.5. IDENTIFICATION OF THE MONITOR

Dr. Joan Albert Arnaiz Gargallo
Clinical Trials Unit (CTU). Clinical Pharmacology Service - UASP Hospital Clínic de Barcelona
C/Rosselló, 138, 08036, Barcelona.
Tel: 93 227 98 38 / Fax: 93 227 98 77

3.6. DATA OF THE INVESTIGATORS OF THE ASSAY

3.6.1. Principal investigator

Dr. Neus Riba i Garcia
Clinical pharmacology service - UASP
Hospital Clínic de Barcelona
C/ Villarroel 170
08036 Barcelona
Tel: 93 227 55 74
Fax: 93 451 44 38

3.6.2. Collaborating researchers

Dr. Esteban Martínez
Infectious Disease Service
Hospital Clínic de Barcelona
C/ Villarroel 170
08036 Barcelona
Tel: 93 227 55 74
Fax: 93 451 44 38

Dr. Marcela Manriquez
Mrs. Gemina Santana (Nurse)

Dr. Cristina Villarroel
Clinical pharmacology service - UASP
Hospital Clínic de Barcelona
C/ Villarroel 170
08036 Barcelona
Tel: 93 227 55 74
Fax: 93 451 44 38

3.7. CENTER WHERE THE ASSAY WILL BE CONDUCTED

- **Experimental phase:** Clinical pharmacology service - UASP Unit, Hospital Clínic de Barcelona. Stairs 2, Basement, door 2e. C/ Villarroel, 170, 08036, Barcelona.
- **Analytical phase:** Central Laboratory of the Hospital Clínic: CDB ("Center de Diagnòstic Biomèdic") Hospital Clínic de Barcelona. C/ Villarroel, 170, 08036, Barcelona.
- **Statistic analysis:** Elisa de Lazzari. Infectious Diseases Service, Hospital Clínic de Barcelona. C/ Villarroel, 170, 08036, Barcelona. Tel: 93 227 54 00 / Extension 2790 / Fax: 93 451 54 24.
- **Committee approving the trial**: Clinical Research Ethics Committee of the Hospital Clínic de Barcelona (CEIC Hospital Clínic).
- **Laboratory for sample and security analytics:** Central Laboratory of the Hospital Clínic: CDB ("Center de Diagnòstic Biomèdic"). C/ Villarroel, 170, 08036, Barcelona.

3.8. ESTIMATED DURATION OF THE TEST

The clinical phase of the study will last approximately 9 to 11 weeks, time necessary to recruit and review the volunteers (3 weeks) and perform the two experimental periods (6 to 8 weeks).

4. JUSTIFICATION AND OBJECTIVES

4.1. JUSTIFICATION

The current treatment of HIV infection consists in the use of the combination of antiretrovirals (ARV) belonging to different families. These drugs have contributed to the transformation of HIV infection into a chronic disease. But these medications can also cause side effects. Potential side effects are changes in the distribution of body fat (lipodystrophy) and impaired glucose balance, leading in some cases to diabetes. The levels of total cholesterol or its fractions and triglycerides can also rise. A potential impact of antiretroviral treatment has been described in the development of osteoporosis, and the risk of producing mitochondrial toxicity, with its clinical repercussions as well as biochemical values, such as hyperlalctatemia. Some of these

side effects may increase your risk and prevalence over time. Sometimes they can be very serious as is the case of lactic acidosis, the most obvious clinical manifestation of mitochondrial toxicity. In other cases, dyslipidemia may require pharmacological intervention due to an increased cardiovascular risk. Finally, the onset of diabetes would require stopping antiretroviral treatment with drugs from the family of protease inhibitors.

Fuzeon® belongs to a new family of ARV, fusion inhibitors that has shown low toxicity in the group of HIV-infected patients, and minimal side effects. It is administered in SC injections, in doses of 90mg twice a day. Fuzeon® has a promising safety and tolerability profile and improves the prognosis of patients infected with HIV who have received multiple previous treatments. It has also been shown to have no adverse effects similar to other ARV groups, and does not produce pharmacological interactions with them. In the TORO 1 and 2 studies it was demonstrated that the incorporation of Fuzeon® to the optimized treatment had no effect on the body changes, neither on the metabolism of glucose, nor on the metabolism of lipids compared to the patients who carried only the optimized treatment without Fuzeon®. This may lead us to conclude that Fuzeon® probably does not have potential mitochondrial toxicity effects (implicated in the physiological duck effect of these syndromes) and therefore may be a therapeutic option in patients with hyperlalctatemia and lactic acidosis. To test this hypothesis and determine the safety profile of this product (already available in the Spanish Market) we designed the study of the metabolic effects of Fuzeon® in healthy volunteers.

Carrying out metabolic tolerability studies of ARV drugs in healthy subjects is recent. This type of study showed that some protease inhibitors such as indinavir and lopinavir/ ritonavir could induce hyperlipidemia and insulin resistance per se. Studies in healthy volunteers allow the elimination of the bias implied by HIV infection itself, since even in the absence of ARV treatment, it may cause an alteration of plasma lipids and insulin sensitivity, due in part to the chronic inflammatory response produced by HIV infection itself. Initial studies in healthy volunteers were conducted for a short period of time, from a single dose to a period of 4 weeks. The determination of plasma lipids and the oral glucose tolerance test have been tests that have been used in these studies; both are simple and can be performed in any hospital center. Although more complex tests have also been performed in these studies for the determination of insulin sensitivity as the hyperinsulinemic euglycemic clamp, the correlation that showed this more sophisticated test with the oral glucose tolerance test was narrow.

The relative decrease in the amount of mitochondrial DNA with respect to nuclear DNA is the most widely used test to demonstrate the mitochondrial toxicity of ARV drugs. However, HIV infection itself in the absence of antiretroviral treatment may also contribute to a certain degree of mitochondrial toxicity. To avoid this bias, the present study has been proposed in healthy volunteers. In patients infected with HIV, modification of ARV treatment or its suspension has been associated rapidly (days)

with modifications of mitochondrial DNA in peripheral blood mononuclear cells, in hepatocytes or in adipocytes. Therefore, we hope if the treatment with enfuvirtide had any repercussion on the mitochondrial DNA, this would be evident at the time in which the study was proposed. The determination of mitochondrial DNA is a more complex test than the determination of plasma lipids or the performance of an oral glucose tolerance test, although our center has extensive experience in carrying out this test.

Our hypothesis is that treatment with enfuvirtide will not cause clinically detectable metabolic alterations on plasma lipids or insulin sensitivity nor will it produce a reduction in mitochondrial DNA content.

4.2. OBJECTIVES

4.2.1. Main objective

The objective of this project is to determine if the administration of enfuvirtide (Fuzeon®) during 7 days, at the usual doses of 90 mg/12h, causes changes in lipid metabolism.

4.2.2. Secondary objectives

As a secondary objective, it will be evaluated whether this treatment regimen causes modifications in the metabolism of glucose and mitochondrial DNA.

5. TYPE OF CLINICAL TRIAL AND DESIGN

This is a pharmacodynamics-phase I clinical trial in healthy volunteers, to evaluate the effects of enfuvirtide (antiretroviral authorized for HIV) on lipid and hydrocarbon metabolism and mitochondrial function. The study, unicentric, has a double-blind, placebo-controlled design, masked, crossed (two formulations, Fuzeon® and placebo, and two treatment periods) and with random assignment of the treatment sequence. Both formulations, Fuzeon® and placebo (saline) will be administered subcutaneously every 12 hours for 7 days. All the participants will receive both treatments (crossed) leaving between them a washing period of at least 4 week to avoid a "carry over" effect. After the selection and review of the volunteers over three weeks, they will participate in two experimental periods, separated by a washout period of at least 1 week. In one of the two periods the participants will receive 7 days of treatment with enfuvirtide (90 mg, every 12 hours subcutaneously) and in the other period they will be given the placebo (1mL od saline), every 12 hours, also for 7 days and subcutaneously. All participants will receive both treatments; the order of the treatment sequence (enfuvirtide-placebo or placebo-enfuvirtide) will be decided randomly.

6. SELECTION OF SUBJECTS

Fourteen healthy volunteers, all male, who have previously been informed of the study and its risks, and who have signed the consent form will be included. They will be given a clinical history, a physical examination, an ECG and a blood test (biochemistry,

hematology, urine, serologies for hepatitis B and C and HIV, drugs of abuse in urine). (See point 8.1.).

6.1. SAMPLE SIZE

The number of subjects was determined based on the hypothesis that the study drug will not cause changes in lipid metabolism. Elevations ≥25 mg/dl in the values of total cholesterol with respect to baseline values will be considered significant and indicative of lipid metabolism alteration. Based on this and using the values of interindividual variability in healthy population cholesterol levels (data from previous studies conducted with healthy volunteers) which is around 30 mg/dL, it was obtained that (with a power of 80 % and an alpha error of 0.025, for a unilateral test) will require 14 subjects to detect elevations of cholesterol levels ≥25 mg/dl.

6.2. INCLUSION CRITERIA

Those people who meet the following criteria will be included in the study:

- Healthy male subjects.
- Age between 18 and 45 years.
- Body mass greater than 19 and less than 25 kg/m2.
- Non-pathological clinical history.
- Findings of the physical examination, normal or without clinical relevance.
- Analytical findings, normal or without clinical relevance.
- Serology hepatitis B, Hepatitis C and HIV negative.
- Analysis of drugs in negative urine.
- The subject understands and accepts the trial procedures and gives written approval informed consent.

6.3. EXCLUSION CRITERIA

Those subjects who:

- Do not meet the above requirements.
- Suffering from organic disorders or have undergone major surgery in the 90 days prior to the start of the trial.
- Have a serious psychiatric history.
- History of dyslipidemia.
- Consumption of more than 30g of alcohol/day.
- Smokers (smoker is considered ≥1 cigarette, cigar or pipe a day).
- Consumers significant amount of food or drinks with xanthines (more than five units of coffee, tea or cola a day).
- Have been under medical treatment in the 30 days prior to inclusion in the study and/or any symptomatic medication in the 7 days prior to the start of the trial.
- Have participated in another clinical trial during the 3 months prior to the start of the present study.
- History of liver disease, kidney disease and any other disorder that may alter the absorption, metabolism distribution or excretion of the drug.
- History of drug allergy.

- Another type of non-drug allergy that is clinically relevant.
- Subjects with a history of myopathy and rhabdomyolysis

6.4. DIAGNOSTIC CRITERIA FOR PATHOLOGIES IN STUDY

Not applicable since it is a study in healthy volunteers (see inclusion and exclusion criteria).

6.5. WITHDRAWAL CRITERIA FOR AND ANTICIPATED ANALYSIS OF WITHDRAWALS AND ABANDONMENTS

Participants may leave the study at any time they wish without specifying the reasons. All those persons who present adverse reactions that, in the opinion of the researcher, endanger their health, and those who demonstrate lack of collaboration or transgressions of the study norms will be withdrawn from the study.

The reasons for dropouts or withdrawals from the study, if any, should also be included in the corresponding CRD sheets.

The abandonments for non-medical reasons will be replaced by new cases so that the number of inclusions is the one prefixed in each treatment group. For the evaluation of the main variable (modifications of serum cholesterol levels) as well as for the following secondary variables, fractions, triglycerides, glucose metabolism and mitochondrial DNA, only the results of the volunteers who complete the study will be considered. For the assessment of safety and tolerability, all volunteers who have received at least one dose of either of the two study drugs will be considered.

6.6. TREATMENT OF LOSS PRERANDOMIZATION

Not applicable.

6.7. DURATION OF THE RECRUITMENT PERIOD BASED ON THE NUMBER OF AVAILABLE PATIENTS

In the three weeks prior to the start of the first experimental period (administration of the drugs under study/placebo) the volunteers will be selected by clinical history and physical examination, EKG, blood and urine tests.

7. DESCRIPTION OF THE TREATMENT

7.1. DOSAGE, DOSAGE, ROUTE OF ADMINISTRATION AND FORMAT

The subject will receive the two formulations (enfuvirtide and placebo) in two different periods (Period 1 and 2) separated by at least one week of washing-out period. Each period will last 7 days, where volunteers will receive two doses per day (one every 12 h) of active treatment (enfuvirtide 90 mg, 1mL) or placebo (1mL of saline)

subcutaneously. This means that participants will receive a first period of treatment with enfuvirtide or placebo for 7 days with a regimen of 90 mg every 12 hours (Period 1). After the 7 days of treatment there will be a washout period (participants do not receive any medication) of 1 week ($t_{1/2\beta}$ of enfuvirtide: 9 hours). After washing-out period, participants will receive the other treatment (Period 2) with a schedule, dosage and duration identical to period 1 but those volunteers who received enfuvirtide in the first period, will receive placebo in the second and vice versa. The order in which the participants will receive the two treatments (enfuvirtide-placebo or placebo-enfuvirtide) will be established randomly (see point 7.2). The treatments will be administered in the Clinical pharmacology service - UASP Unit, Hospital Clínic de Barcelona by the research staff to ensure compliance. The administration of the medication will take place at 9 a.m. and 9 p.m. Both treatments (active and control) will be administered subcutaneously.

7.2. ASSIGNMENT OF TREATMENT

All participants will receive both treatments (enfuvirtide and placebo). The allocation of the treatment sequences (enfuvirtide-placebo or placebo-enfuvirtide) will be done in a randomized and balanced manner. The randomization list will be prepared through a computer program by the CTU (Clinical Trials Unit) of the Clinical Pharmacology Service of the Hospital Clínic of Barcelona. The CTU will provide the Trials Agency of the Hospital Clínic with the random assignment list so that they can proceed with the preparation of the medication for the study, which is administered following a double-blind scheme (see point 7.3). It will also provide, in a sealed envelope, the code of random assignment of each participant to the Principal Investigator.

7.3. PREPARATION OF THE STUDY MEDICATION

The preparation of the medication will be carried out by specialized staff of the Agency of Clinical Trials Units of the Hospital Clínic of Barcelona.

As this is a double-blind trial, the CTU will provide the staff of the Trial Agency with the list of random assignments to the treatment sequence (as specified in point 7.2.). Based on this list, the staff of the Agency will be in charge of administer the corresponding medication for each volunteer and study period (1 mL of reconstituted enfuvirtide solution or 1 mL of physiological saline placebo) in the administration syringes. The syringes with the medication already prepared for administration will be wrapped and well covered with aluminum foil, opaque, to avoid breaking the blind. A label will be affixed to the aluminum foil to identify which volunteer corresponds to each syringe. The label will contain the following information:

- Study
- Code
- Promoter
- Principal Investigator
- No Volunteer (from 1 to 14)
- Period 1 or 2

- Reconstitution date
- Reconstitution time
- Control digit

Prepared syringes should be stored in the refrigerator between 2-8ºC until their administration, according to the indications of the product data sheet. To prevent the investigator responsible for administering the study medication from seeing a difference in temperature between the different syringes, all syringes, including those loaded with placebo, should be kept in the refrigerator until collected by the investigator responsible for Phase I, for its administration.

Since the enfuvirtide reconstitution solution must be administered within 24 hours, according to the technical data sheet, the personnel of the Testing Agency must prepare the syringes for administration of the medication no earlier than this time.

7.3.1. Instructions for the reconstitution and preservation of Fuzeon® and placebo

1. Fuzeon® 90 mg/mL comes in the form of a white lyophilized powder for solution for injection. Each vial contains 108 mg of enfuvirtide, powder. Once the solution is reconstituted, 1 mL contains 90 mg of enfuvirtide.
2. Fuzeon® should only be reconstituted with 1.1 ml of Water for Injection (solvent that accompanies the powder vial).
3. Add the water for injection and tap the vial gently with the fingertips until the powder starts to dissolve. Never shake the vial or invert it to mix it, as this will cause too much foam to be produced.
4. Once the powder starts to dissolve, the vial can be allowed to stand to allow complete dissolution. The powder can take up to 45 minutes to dissolve. The vial can be rolled gently between hands after adding the water for injection until the powder is completely dissolved, which can reduce the time it takes for it to dissolve.
5. Before removing the solution for administration (1 mL), the person in charge of the preparation should make a visual inspection of the vial to verify that all the contents have dissolved, that the solution is transparent and that it does not present bubbles or particles. If particles in suspension are observed, the vial should not be used, but should be discarded.
6. Fuzeon® does not contain preservatives. Once reconstituted, you should inject immediately. If the reconstituted solution can not be injected immediately, it should be kept in the refrigerator between 2 and 8 ºC, protected from light, and used within 24 hours. The reconstituted and refrigerated solution should be brought to room temperature before its injection.

7.3.2. Masking

Experimental drug: Enfuvirtide, Fuzeon®.

For those volunteers who receive the experimental treatment, the staff of the Testing Agency of the Hospital Clínic will be responsible for reconstituting the Fuzeon® vial powder according to the instructions of point 7.3.1. Once reconstituted, it will load 1 mL of the reconstitution solution (containing 90 mg of enfuvirtide) into a syringe. Wrap the syringe with aluminum foil to preserve the drug from the light, as indicated in the product's data sheet and, at the same time, to maintain the "double blind" of the study. In case the preparation should not be administered immediately, it will be stored in the refrigerator at a temperature between 2 and 8ºC. The aluminum foil will be affixed with a label with the following information:

- Study
- Code
- Promoter
- Principal Investigator
- No Volunteer (from 1 to 14)
- Period 1 or 2
- Reconstitution date
- Reconstitution time
- Control digit

Prepared syringes should be stored in the refrigerator between 2-8ºC until their administration, according to the indications of the product data sheet. To prevent the investigator responsible for administering the study medication from seeing a difference in temperature between the different syringes, all syringes, including those loaded with placebo, should be kept in the refrigerator until collected by the investigator responsible for Phase I, for its administration.

Since the enfuvirtide reconstitution solution must be administered within 24 hours, according to the technical data sheet, the personnel of the Testing Agency must prepare the syringes for administration of the medication no earlier than this time.

Control drug: Physiological saline 0.9% Braun.

For those volunteers who receive the control treatment (placebo), the staff of the Testing Agency of the Hospital Clínic will load in a syringe identical to the one used to administer the active treatment, 1 mL of saline solution. The syringe will be wrapped with aluminum foil to keep the "double blind" of the study and it will attach a label with the information specified above. These syringes will also be stored in the refrigerator at a temperature between 2 and 8ºC. Once the study medication is prepared, the staff of the Trial Agency of the Hospital Clínic will deliver it to the researchers of the Phase I Unit, for its administration, to the participating volunteers.

7.4. STORAGE AND HANDLING

The promoter will provide the samples of the drugs to be studied (Fuzeon® and physiological saline) to the Pharmacy Service, in particular to the Clinical Trials Agency of the Hospital Clínic of Barcelona with a minimum of 15 days before the start date of the trial, and will be guarded by it, until they are delivered to the researcher of Phase I for administration. In addition to storage and custody of the study samples, the Testing Agency will be responsible for preparing the medication for administration, as specified in section 7.3. Clinical investigators will collect the syringes with the medication already loaded, duly wrapped in opaque paper and labeled, a few minutes before the administration to allow the samples to return to room temperature, such as specify the product's technical sheet. If for organizational reasons Phase I researchers should pick up the medication more than 2 hours before the administration time, it would be stored in the refrigerator of the Phase I Unit between 2-8ºC, until minutes before administration. Once all the syringes are empty, all the empty syringes will be saved until the test has been monitored and the medication has been correctly administered. To verify that there have been no medication errors, the investigator responsible for administering it will copy in the CRD, on the corresponding page, the control digit of the syringe label, each time you make an injection. The date and time of the collection of the medication to the Testing Agency, by the researcher of Phase I responsible for the administration, will be recorded in a document (according to PNTs of Phase I) in which the person must sign. delivers the medication and the person who receives it.

7.5. ADMINISTRATION OF STUDY MEDICATION

The administration of the treatments of the study will be carried out by one of the investigators who must write down in the corresponding CRD the time of administration, the place of administration (right or left arm, right or left thigh or abdomen) and his initials and signature. You must also copy in the CRD the date and time of reconstitution and the check digit on the syringe label. The medication will be administered around 9.00 a.m. and 9.00 p.m., approximately (the schedule is one injection every 12 hours). The researcher responsible for administering the medication will collect the loaded syringes (with enfuvirtide or placebo), duly wrapped in opaque paper and labeled (as mentioned in section 7.3.), Thus maintaining the double blind.

The medication will be collected a few minutes before the scheduled time of administration. If for organizational reasons Phase I researchers should pick up the medication more than 2 hours before the administration time, it would be stored in the refrigerator of the Phase I Unit between 2-8ºC, until minutes before administration.

Once the test is finished and it has been verified that the medication has been administered correctly. To verify that there have been no medication errors, the investigator responsible for administering it will copy in the CRD, on the corresponding page, the control digit of the syringe label, each time you make an injection. The date and time of the collection of the medication to the Testing Agency by the researcher of Phase I responsible for the administration, will be recorded in a document (according to

PNTs of Phase I) in which the person who delivers must sign. the medication and the person who receives it.

7.6. CONCURRING TREATMENTS

During the trial, additional medications are not authorized. Exceptionally, acetaminophen will be allowed. Other drugs for symptomatic treatment will be allowed, at a single dose, in case it is administered during the bleaching period and if it is not expected that this drug could interfere in the evaluation of cholesterol levels and other parameters of the trial. Dose, time and cause will be recorded in the corresponding sheets of the register of the subject in question. The principal investigator, together with the monitor, will assess the possible exclusion of the volunteer in case any of the aforementioned conditions is not met.

The minimum time that should elapse from the suspension of any prescription treatment until the inclusion of the subject in the study will be 30 days. In the case of single-dose symptomatic therapy, it will be allowed up to one week before the study day, assuming that the drug has been completely eliminated.

7.7. ASSESSMENT OF THE COMPLIANCE

The administration of the study medication to the volunteer participating in the study will be directly observed by the researcher, ensuring that the volunteer receives all doses.

7.7.1. Assessment of compliance with the diet

To verify that the volunteers have followed the recommended diet, they will be provided with a notebook in which they should write down what they eat each day. The notebook will be checked by the investigators daily (at the time to receive the study medication).

8. DEVELOPMENT OF THE ASSAY AND EVALUATION OF THE RESPONSE

8.1. SELECTION OF VOLUNTEERS

In the 3 weeks prior to the start of the study, volunteers will be selected by clinical history, physical examination, EKG, blood and urine tests. The general blood test will consist of a biochemical profile, blood count, coagulation, serology, urine analysis and determination of drugs of abuse in urine. The selection tests will be carried out to as many people as necessary to get 14 subjects that meet all the inclusion criteria and none of the exclusion criteria.

8.1.1. Selection tests

The selection tests will be based on:

- Information and signature of informed consent.

- Clinical history: filiation, allergies, pathological history, toxic habits, anamnesis per systems.
- Complete physical examination, including height, weight and calculation of BMI.
- Vital signs collection: Axillary temperature, heart rate and blood pressure after 5 minutes of sitting rest.
- 12-lead ECG.
- Blood biochemistry: glucose, urea, creatinine, sodium, potassium, total bilirubin, AST (GOT), ALT (GPT), gamma-GT (GGT), alkaline phosphatase, calcium, phosphorus, total proteins, cholesterol, HDL cholesterol, cholesterol LDL, triglycerides and lactate.
- Hemogram: red blood cells, hemoglobin, hematocrit, MCV, HCM, CCMH, count and leukocyte formula.
- Serologies: determinations for the hepatitis B virus, hepatitis C and HIV.
- Urine test: density, pH, glucose, ketone bodies, bilirubin, urobilinogen, proteins, red blood cells/hemoglobin, leukocytes and nitrites.
- Drugs of abuse in urine.

Blood tests will be performed with the volunteers fasting for 12 hours.

All volunteers who, after the selection tests, meet all the inclusion criteria and do not meet any of the exclusion criteria, will be included in the study.

8.2. STUDY DEVELOPMENT

All subjects included in the trial will participate in two experimental periods (period 1 and period 2) separated by one week of washing-out period. Both experimental periods will be identical, following the same development scheme.

The 14 volunteers that are included in the study (after the screening tests have been carried out) will begin, on Day 1 of Period 1, a standard diet (equal for all) with the intention of minimizing alterations in cholesterol levels. This diet maintains the proportion of 10-15% of proteins, 50-60% of carbohydrates and 30-35% of fats, as recommended by the WHO. To facilitate the following-up of this, the Dietetic Service of the Hospital Clínic of Barcelona will elaborate different menu options.

After 7 days of the standard diet, on day 8 of Period 1, the volunteers will undergo a blood test identical to that of the selection, plus a standard oral glucose tolerance test and a mitochondrial DNA quantification test. These are baseline tests before the administration of the study medication. The day after the pretreatment blood test (day 9 of Period 1), the volunteers will receive the corresponding medication according to the randomization sequence, enfuvirtide or placebo (see point 7.3). Since then and for 7 days, volunteers must go to the Inther Unit of the Hospital Clínic every 12 hours (approximately at 9 a.m. and 9 p.m.) where they will take a 90 mg dose of enfuvirtide or placebo subcutaneously. During all this time the volunteers should follow the standard diet.

After 7 days of treatment (day 16 of Period 1), volunteers will perform a blood test identical to the baseline (day 8 of Period 1) to assess possible changes in lipid and hydrocarbon metabolism, and mitochondrial function due to the treatment. Once the first period of the study ends, the standard diet ends, a wash-out period of at least 1 week is then started.

After the washing period, Period 2 will start identical to Period 1: 7 days of standard diet, pretreatment blood test (identical to days 8 and 16 of Period 1), 7 days of treatment, blood test after finishing the treatment. Thus, end of diet and end of the study.

Both periods of the study are identical, the only thing that varies is the order in which they receive the study treatment, that is, some will receive enfuvirtide in Period 1 and placebo in Period 2, while others will receive placebo in Period 1 and enfuvirtide in Period 2. The assignment to the treatment sequence in Period 1 and Period 2 will be carried out as specified in point 7.2. The administration of the medication by the investigators will be done double-blind.

All the analytical controls of the study will be carried out after 12 hours of fasting.

In case the deviations of the results are found in the last visit, the volunteer will be followed up until its normalization.

The two study periods are identical in terms of methodology, varying only the medication that participants will receive in each of them.

8.2.1. Safety

In addition to evaluating pharmacodynamic parameters (main objective of the study), tolerability and safety data will be collected. Before the administration of each of the doses of the treatments under study, blood pressure and heart rate will be recorded (after 5 minutes of sitting rest) and they will be asked about the appearance of adverse events by direct question. (How are you? Have you noticed any discomfort?). Likewise, possible adverse events will be recorded when the subject verbalizes them spontaneously, or is evident to the researchers (see section 9). All those analytical alterations that are observed in any of the visits that will be carried out throughout the study (specified in the previous section), will be recorded as an adverse event according to the WHO Toxicity scale, for the classification of acute or subacute toxic effects.

8.3. SPECIFIC RULES FOR VOLUNTEERS

Volunteers will be given, during the selection visit, a sheet containing all the recommendations to follow and a menu for the standard diet during the specified days, which they must commit to comply during the entire duration of the study. They will also be given a calendar with the activities to do during the entire study. The recommendations or rules to follow are the following:

- You must go to the Phase 1 unit Unit on the days indicated by the researcher and that will appear on your calendar, at the indicated time, on time.

- A minimum of 8 h of night rest will be carried out throughout the duration of the study, except in the washout period, when it will not be mandatory.
- It is not allowed to perform intense physical exercise from the day the standard diet starts, each of the two study periods, and until the end of it.
- The intake of foods rich in xanthines (coffee, cola, tea or chocolate) or grapefruit juice is not allowed on the day that the standard diet is started each of the two periods of the study and until the end of the same, part of the amounts that come specified in the menu that has been delivered.
- Smoking and drinking alcoholic beverages are not allowed from the day the standard diet starts each of the two periods of the study and until the end of it.
- The ingestion of any drug is prohibited. If you need to take a drug as a symptomatic treatment, you must inform the researcher in advance
- It is recommended not to donate blood during the three months following the end of your participation in the study.
- For the proper development of this work it is essential that you follow the instructions provided by the researcher at all times, and that it be extremely timely.

8.4. CLINICAL ASSESSMENT CRITERIA AND EVALUATION

8.4.1 Main variable: Total serum cholesterol levels.

8.4.2. Secondary variables:

- Modifications of HDL and LDL cholesterol and triglycerides levels.
- Modifications of glycemic metabolism after an oral glucose tolerance test.
- Modifications of mitochondrial DNA.
- Safety: the safety of the study drug will be assessed by altering the analytical parameters and recording vital signs (see point 8.2.) as collecting adverse events. The adverse events manifested by the volunteers or evidenced by the researchers, and the subclinical alterations in blood and urine will be recorded.

9. ADVERSE EVENTS.

9.1. DEFINITIONS

An adverse event is any unfavorable medical event that occurs in a patient or subject undergoing clinical research to which a pharmaceutical product is administered, regardless of whether or not it is related to the pharmaceutical product. Any sign (including the alteration of a laboratory parameter), symptom or illness, unfavorable and unintentional, which are temporarily associated with the use of a medicinal product may constitute an adverse event.

A **serious adverse** event is considered to be one that:

- Causes the death of the subject.
- Threatens the life of the subject.

- Produces permanent disability.
- Gives rise to hospitalization or prolongs it.
- Congenital anomalies and malignant processes will always be considered as serious events.

Unexpected adverse events are those experiences not described (in nature, severity or frequency) in the researcher's manual.

9.2. DESCRIPTION

The collection of adverse events should be carried out by the investigator (s) of the trial, describing the adverse event according to its duration, intensity, frequency and causality between the treatment and the adverse event.

The duration refers to the time elapsed from the occurrence of the event until its completion.

The intensity will be defined according to a scale of three degrees:

- **Slight**: Banal adverse events, of minor importance and short duration, which do not substantially affect the patient's life.
- **Moderate**: Adverse events that cause enough discomfort to interfere with the patient's normal life.
- **Severe**: Adverse events that imply an inability to work or perform the patient's usual activity and even force them to abandon the treatment.

The frequency will be defined as: punctual, recurrent (intermittent) or continuous (persistent).

The causal link of an adverse event with the study medication will be established according to the modified Karch and Lasagna classification, in which the following categories are defined:

- **Not related**:
  - Does not meet any of the criteria mentioned.
- **Unlikely**:
  - Reasonable temporal sequence
  - It does not coincide with the scheme of known adverse reactions
  - It can not be explained by the clinical situation of the subject
- **Possible**:
  - Reasonable time sequence
  - Coincides with the scheme of known adverse reactions.
  - It may be due to the clinical situation of the subject or to other drugs administered concomitantly.
- **Probable**:
  - Reasonable time sequence
  - Coincides with the scheme of known adverse reactions
  - Improvement by interrupting the administration of the drug

- It is not explained by the clinical situation of the subject.

- **Certain**:
  - There is a reasonable temporal sequence in relation to the administration of the drug or its plasma or tissue levels.
  - The observed manifestation coincides with the scheme of known adverse reactions of the drug involved.
  - Improvement by interrupting the administration of the drug.
  - Reappears when administered again.

9.3. REGISTRATION

Possible adverse events will be recorded by the investigators as described in section 8.2.1, when they manifest spontaneously by the subjects, are communicated after a direct question from the investigator or are evident to the investigators. The information obtained will be noted in the corresponding section of the individual data collection notebook for each subject, which will include the type of adverse event, its duration, intensity, frequency, final result, causality and the type of corrective measures, if they are used.

If adverse events (clinical or subclinical) are observed, the subjects will be monitored by the investigator during their evolution and until its resolution. An adverse event is considered to be that variation with respect to the reference values that, according to the WHO Toxicity scale for the classification of acute or subacute toxic effects, are within Grade II and successive. If the undesirable effects observed are clinically significant, the affected volunteer will be excluded from the trial.

9.4. NOTIFICATION

The investigator will notify the promoter of the study within 24 hours of serious or unexpected adverse events, by telephone or telefax. Within a maximum period of 72 hours, the Notification Sheet of serious or unexpected adverse events will be delivered to the promoter. If not all the information is available, it can be completed within 15 days. The promoter will notify the Spanish Agency for Medicines and Health Products and the Clinical Research Ethics Committees involved in the trial of serious and unexpected events that may be related with the research treatments. Information on expected serious adverse events, non-serious adverse events and those considered unrelated to the treatments under study will be included in a tabulated form in the final report of the clinical trial.

10. ETHICAL ASPECTS

10.1. GENERAL CONSIDERATIONS

At all times the clinical trial will respect the international ethical declarations of Helsinki (revised version of Edinburgh 2000), the recommendations of the WHO, the

deontological code and those derived from the Spanish legislation on clinical trials (Medication Law 25/1990, Royal Decree 223/2004). Before including any subject in the study, the CEIC and the Spanish Agency for Medicines and Health Products must approve and authorize respectively the study protocol, the information that will be given to the subject and the model of informed consent that will be used. The study will be conducted under the Good Clinical Practice standards.

10.2. INFORMATION AND INFORMED CONSENT

The volunteers will be informed about the objectives, development and scope of the study by the principal investigator or collaborators, and will also receive the consent form where they will have in written the characteristics of the trial. Before being included, all volunteers must sign the informed consent form.

10.3. CONFIDENTIALITY

The researchers agree to keep discretion about the progress of the trial. The protocol and its attached documentation as well as the data collection notebooks and all the information generated during the study are considered confidential. They can only be used by researchers for the specific purposes of this protocol. To preserve the confidentiality of the personal data of the subjects, only the principal investigator, his collaborators and the technical staff who participate in the study will have access. For the same reason, complete filiation data and written consent will be kept in the investigator's file. In accordance with the Organic Law 15/99 of December 13, Protection of Personal Data, in all cases the confidentiality of the data will be strictly maintained. Participants may exercise their right of cancellation, rectification, etc. as provided in the legislation.

10.4. INSURANCE POLICY

The promoter of the trial has an insurance that covers the incidents that may arise from the action of the drugs tested.

11. PRACTICAL CONSIDERATIONS

11.1. RESPONSIBILITIES OF THE PARTICIPANTS IN THE ASSAY

The promoter, the monitor and the researcher will comply with the obligations described in articles 35, 36 and 37 of RD 223/2004 of February 6.

11.2. CORRECTIONS

The corrections of any data in the data collection register should be made by drawing a straight line on the incorrect data (allowing it to be readable), next to it the correct data will be noted. The corrections must always be accompanied by the corresponding clarification signed and dated by the researcher.

11.3. STORAGE OF THE ASSAY DOCUMENTATION

The promoter is responsible for the storage of the trial documentation. Will comply with minimum times that are mentioned in the Royal Decree on Clinical Trials. The investigator will be responsible for keeping the identification codes of the subjects for at least fifteen years after the conclusion or interruption of the trial. The clinical records of the subjects and other original data will be kept for the maximum period of time allowed by the hospital, the institution or the private consultation where the trial was conducted.

11.4. CONDITIONS OF PUBLICATION

All individual and collective data, as well as the results derived from them, will be confidential, and may not be disseminated, commented or published without the knowledge and authorization of the trial promoter.

11.5. INTERRUPTION OF THE ASSAY

The clinical trial may be interrupted at the discretion of the principal investigator and / or the promoter at any time.

12. STATISTICAL ANALYSIS

A report on the statistical analysis of the data will be included in the final report.

12.1 ANALYSIS OF THE MAIN VARIABLE

With the main variable, a descriptive analysis will be made with mean and standard deviation (SD) in case the variable is distributed normally, otherwise it will be described with median and interquartile range.

The period, treatment and sequence effect will be evaluated with the variance test (ANOVA). To verify that there is no "carry-over" effect, a Student’s T test will be performed on the ratio and absolute values in both periods, to verify that the means between the two periods are the same in both sequences.

To assess the magnitude of the change in total cholesterol during each period, this variable will be transformed into a ratio (final value/initial value), so this quotient will show an appreciation of the effect of the change between the values at the beginning of the period with respect to the values of the end of the period.

It was established that elevations of ≥25 mg/dl in the values of total cholesterol with respect to baseline values will be considered significant and indicative of impaired lipid metabolism. To assess this aspect, the difference between the final values of cholesterol in each period minus the initial values will be made.

12.2. ANALYSIS OF SECONDARY VARIABLES

12.2.1. Secondary variables.

For the secondary variables triglycerides, fractions of HDL and LDL cholesterol, oral glucose tolerance test and mitochondrial DNA, a description will be made with means

and standard deviation (SD) in case they show a normal distribution or with medians and interquartile range. A Student`s T test will be made to all the variables to verify the existence of a "carry-over" effect.

For the variables triglycerides and HDL and LDL cholesterol, the magnitude of change between both periods will be evaluated as a percentage.

A variance test (ANOVA) will be performed to evaluate if there was a period, treatment or sequence effect.

The values of the oral glucose tolerance test curves will be classified according to their values: not altered (glucose at 120 minutes <140mg/dL) or altered (glucose at 120 minutes >140 mg/dL).

In each period and in each sequence, a contingency table (2 x 2) will be made and the number of volunteers that show changes in the state of the curve will be described.

12.2.2. Tolerability analysis

The individual values grouped by treatment received will be tabulated, applying a descriptive analysis (calculation of means and standard deviations) for the quantitative variables (blood pressure and heart rate), and frequencies for the qualitative variables (notification of symptoms). The clinical and analytical alterations will be analyzed individually, as well as considering their meaning in the treatment group. In any case, its relevance in terms of "clinically relevant changes" will be assessed. The adverse events observed during the development of the study will be described and discussed, specifying its duration, intensity, frequency and causality (as described in section 9.2).

12.2.3. Size of the sample

The number of subjects was determined based on the hypothesis that the study drug would not cause changes in lipid metabolism. Elevations ≥25 mg/dl in the values of total cholesterol with respect to baseline values would be considered significant and indicative of lipid metabolism alteration. Based on this and using the values of interindividual variability in healthy population cholesterol levels (data from previous studies conducted with healthy volunteers) which is around 30 mg/dL, it was obtained that (with a power of 80 % and an alpha error of 0.025, for a unilateral test) 14 subjects were needed to detect elevations of cholesterol levels ≥25 mg/dl.

13. BIBLIOGRAFÍA

1. Lalezari JP, Henry K, O'Hearn M, et al. Enfuvirtide, an HIV-1 fusion inhibitor, for drug-resistant HIV infection in North and South America. N Engl J Med. 2003; 348 (22): 2175-85.
2. Lazzarin A, Clotet B, Cooper D, et al. Efficacy of enfuvirtide in patients infected with drug-resistant HIV-1 in Europe and Australia. N Engl J Med. 2003; 348 (22): 2186-95.
3. Delfraissy J-F, Montaner J, Eron J, et al, for the TORO Study Team. Summary of Pooled Efficacy and Safety Analyzes of Enfuvirtide Treatment for 24 Weeks in Toro 1 and TORO 2 Phase III Trials in Highly Antiretroviral Treatment-experienced Patients. Program and abstracts of the 10th Conference on Retroviruses and Opportunistic Infections (CROI); February 10-14, 2003; Boston, MA. Poster 568
4. Cooper DA et al. (TORO 1 and TORO 2 study groups). The Effects of Enfuvirtide Therapy on Body Composition and Serum Lipids through 48 Weeks in the TORO Trials. Abstract 715 (poster). Abstract 34 (oral). Program and Abstracts of the 11th Conference on Retroviruses and Opportunistic Infections (11th CROI). February 8-12, 2004. San Francisco, CA.
5. Purnell JQ, Zambon A, Knopp RH, et al. Effect of ritonavir on lipids and post-heparin lipase activities in normal subjects. AIDS. 2000; 14: 51-57.
6. Noor MA, Lo JC, Mulligan K, et al. Metabolic effects of indinavir in healthy HIV-seronegative men. AIDS 2001; 15: 11-18.
7. Noor MA, Seneviratne T, Aweeka FT, et al. Indinavir acutely inhibited insulin-stimulated glucose disposal in humans: a randomized, placebo-controlled study. AIDS. 2002; 16: F1-F8.
8. Noor MA, Parker RA, O'Mara E, et al. The effects of HIV portease inhibitors atazanavir and lopinavir / ritonavir on insulin sensitivity in HIV-seronegative healthy adults. AIDS 2004; 18: 2137-2144.
9. Karch y cols. Clin. Pharmacol. Ther, 1977; 21:247 - 254.
