# Supplementary material for: Metabolic, mitochondrial, renal and hepatic safety of enfuvirtide and raltegravir antiretroviral administration: Randomized crossover clinical trial in healthy volunteers
Source: PLoS One. 2019 May 23;14(5):e0216712. doi: 10.1371/journal.pone.0216712 (PMC6532851; doi:10.1371/journal.pone.0216712)
Supplement: S2 File — (PDF) [file pone.0216712.s002.pdf]

Influence of raltegravir (Isentress®) into lipid and hydrocarbonate metabolism and mitochondrial function in healthy volunteers

CLINICAL TRIAL PROTOCOL

PROTOCOL CODE: RAL-MET-VOL-HCB
(Version 1.1, 07.11.08)
Nº EUDRACT: 2008-003288-37
Clinicaltrials.gov #: NCT00772720

CONFIDENTIAL

DEVELOPER: Dr. José Mª Gatell Artigas
Servicio de Enfermedades Infecciosas
Hospital Clínic de Barcelona
C/ Villarroel 170
08036 Barcelona

1. SUMMARY

1. Type of application

Phase I clinical trial, pharmacodynamics, with a pharmaceutical specialty already authorized.

2. Developer identification

Dr. José Mª Gatell Artigas
Infectious Disease Service
Hospital Clínic de Barcelona
C/ Villarroel 170
08036 Barcelona

3. Clinical trial tittle

Influence of raltegravir (Isentress®) into lipid and hydrocarbonate metabolism and mitochondrial function in healthy volunteers.

4. Protocol code

- RAL-MET-VOL-HCB
- Nº EUDRACT: 2008-00328-37

5. Principal investigator

Dr. Esteban Martínez
Infectious Disease Service
Hospital Clínic de Barcelona
C/ Villarroel 170
08036 Barcelona
Tel: 93 227 55 74
Fax: 93 451 44 38

6. Centers where the study will be conducted

Experimental phase:

Inther Unit
Hospital Clínic de Barcelona
Stairs 2, Floor 3
C/ Villarroel 170
08036 Barcelona

Analytical phase:

Laboratorio Central del Hospital Clínic: CDB (Centre de Diagnòstic Biomèdic)

Hospital Clínic de Barcelona
C/ Villarroel 170
08036 Barcelona

Statistical analysis:

Elisa de Lazzari
Infectious Diseases Service
Hospital Clínic de Barcelona
C/ Villarroel 170
08036 Barcelona
Tel: 93 227 54 00 / Extension 2790
Fax: 934515424

7. Ethical committees that have approved the trial

Clinical Research Ethics Committee of the Hospital Clínic de Barcelona (CEIC Hospital Clínic).

8. Responsible for monitoring

Dr. Joan Albert Arnaiz
Clinical Trials Unit (CTU). Clinical pharmacology service - UASP
Hospital Clínic de Barcelona
C/ Roselló 138
08036 Barcelona
Tel: 93 227 92 38
Fax: 93 227 98 77

9. Experimental and control drug

Experimental drug:

Isentress®: 400 mg-tablets of raltegravir. Administred orally.
Therapeuic group: J05AX08 antiviral for systemic use. Other antiretrovirals.
Administered every 12 hours within 7 days.

Control drug:

Placebo: Tablets. Administred orally.
Administered every 12 hours within 7 days.

10. Cinical trial phase

Clinical trial in phase I in healthy volunteers (Phase IV-I), pharmacodynamics.

11. Main objective

Study the modifications on the lipid and hydrocarbon metabolism, and mitochondrial function that raltegravir produces in healthy volunteers

12. Design

Unicentric, in healthy volunteers, double-blind, crossed, placebo-controlled clinical trial with random allocation of treatment sequence.

13. Pathology to study

This study will be conducted in healthy volunteers without concurrent pathology.

14. Main variable

Modifications in plasmatic total cholesterol levels.

15. Study population and number of patients

A total of 14 healthy volunteers will be included. All men.

16. Treatment duration

This trial comprises a total of two periods of 7 days each, separated from each other for a 4-week-period. In each of the periods, one of the two possible treatments (raltegravir or placebo) will be administered, with one dose taken orally every 12 for 7 days.

17. Calendar and expected completion date

Once the authorization of the CEIC and the Spanish Agency for Medication and Healthcare Products (AEMPS) will be received, the trial will be conducted. The experimental phase is schedule to December, 2008. Preliminary results may be informed in December, 2009.

2. INDEX

1. **Summary……………………………..…………………………..2**
2. **Index…………….……………..………………………………5**
3. **General information……...……………………………..…………7**

   3.1 Identification of the assay……….. ……………………………..7
   3.2 Type of clinical trial ……………………………………………..7
   3.3 Products description (experimental and control) ……...…………7
   3.4 Data relative to the promoter ……………………………...…..8
   3.5 Identification of the monitor ……………………………………..8
   3.6 Data of the investigators of the assay ……………………………8
   3.7 Center where the assay will be conducted ………………………8
   3.8 Estimated duration of the test ……………………………………9

4. **Justification and Objectives………………………………………9**

   4.1 Justification…………………………………………………9
   4.2 Objectives……………………………………………………11

5. **Type of clinical trial and design…………………………………12**
6. **Selection of subjects…………………………………………….12**

   6.1 Sample size…………………………………………………...12
   6.2 Inclusion criteria ………………………………………………..12
   6.3 Exclusion criteria ………………………………………………12
   6.4 Diagnostic criteria for pathologies in study ……………………13
   6.5 Withdrawals and abandonments ………………………………..13
   6.6 Treatment of loss prerandomization ……………………………13
   6.7 Recruitment period ……………………………………………..13

7. **Description of the treatment……………………………………..13**

   7.1 Dosage, dosage, route of administration and format …………...14
   7.2 Assignment of treatment………………………………………..14
   7.3 Preparation of the study medication ……………………………14
   7.4 Storage and handling …………………………………………...15
   7.5 Administration of study medication ……………………………15
   7.6 Concurring treatments …………………………………………15
   7.7 Assessment of the compliance …………………………………16

8. **Development of the assay and evaluation of the response……...16**

8.1 Selection of volunteers……………………………………………16
8.2 Study development……………………………………………...17
8.3 Specific rules for volunteers ……………………………………18
8.4 Clinical assessment criteria and evaluation ……………………19

9. Adverse events………………………..……………………...19

9.1 Definitions ……………………………..…………………………19
9.2 Description …………………………..…………………………19
9.3 Registration ……………………..……………………...20
9.4 Notification…………………………..………………………21

10. Ethical aspects…………………………………………………21

10.1 General considerations……………..……….. ……………...21
10.2 Information and informed consent……………………………21
10.3 Confidentiality……………………………………………....21
10.4 Insurance policy………………………….. ………………..22

11. Practical considerations ……………………..………………..22

11.1 Responsibilities of the participants ………..………………22
11.2 Corrections …………………………..……..………………22
11.3 Storage of the assay documentation…………….…………22
11.4 Conditions of publication ……………………………………..22
11.5 Interruption of the assay ………………………………………23

12. Statistical analysis …………………………..………………...23

12.1 Analysis of the main variable……………………………………23
12.2 Analysis of secondary variables………………………………23

13. Bibliography…………………………..……………………..25

3. GENERAL INFORMATION

3.1 IDENTIFICATION OF THE ASSAY

- **Title:** "Influence of raltegravir (Isentress®) into lipid and hydrocarbonate metabolism and mitochondrial function in healthy volunteers".
- **Protocol code:** RAL-MET-VOL-HCB (version 1.1, 07.11.08).
- **No EUDRACT**: 2008-003288-37

3.2. TYPE OF CLINICAL TRIAL

A pharmacodynamic phase I clinical trial with healthy volunteers will be conducted to evaluate the effects of raltegravir (authorized pharmaceutical specialty for HIV) on lipid and hydrocarbon metabolism, and mitochondrial function. This is a double-blind, placebo-controlled, masked, cross-over study (two formulations, Isentress® and placebo and two treatment periods) with random assignment of the treatment sequence. Both formulations, Isentress® and placebo will be administered orally every 12 hours for 7 days. All participants will receive both treatments (crossed) leaving a washing out period of at least 1 week to avoid a "carry over" effect between them.

3.3. PRODUCTS DESCRIPTION (EXPERIMENTAL AND CONTROL)

A. Generic name, trade name

- **Experimental drug:** Raltegravir, Isentress®.
- **Control drug**: Placebo.

B. Quantitative and qualitative composition. Notifiable excipients

- **Experimental drug**: Isentress® 400 mg tablets of raltegravir.
- **Control drug:** Placebo

C. Pharmaceutical form

- **Experimental drug:** Isentress® Each film-coated tablet contains 400 mg of raltegravir (potasic). Excipient: each tablet contains 26.06 mg of lactose monohydrate.
- **Control drug**: Placebo, provided by MSD.

D. Sampling entities

- **Experimental drug**: raltegravir (Isentress®). MSD Laboratories.
- **Drug control:** Placebo. MSD Laboratories

E. Dosage and route of administration

Oral administration of 400 mg every 12 hours of raltegravir or placebo for 7 days, respectively in each of the two periods of the study.

3.4. DATA RELATIVE TO THE PROMOTER

Dr. José Mª Gatell Artigas
Head of Infectious Diseases Service
Hospital Clínic de Barcelona
C/Villarroel 170, 08036, Barcelona
Tel .: 93 227 54 30 / Fax: 93 451 44 38

3.5. IDENTIFICATION OF THE MONITOR

Dr. Joan Albert Arnaiz Gargallo
Clinical Trials Unit (CTU). Clinical Pharmacology Service - UASP Hospital Clínic de Barcelona
C/Rosselló, 138, 08036, Barcelona.
Tel: 93 227 98 38 / Fax: 93 227 98 77

3.6. DATA OF THE INVESTIGATORS OF THE ASSAY

3.6.1. Principal investigator

Dr. Esteban Martínez
Infectious Disease Service
Hospital Clínic de Barcelona
C/ Villarroel 170
08036 Barcelona
Tel: 93 227 55 74
Fax: 93 451 44 38

3.6.2. Collaborating researchers

Maria larousse and Montse Loncà
Infectious Disease Service
Hospital Clínic de Barcelona
C/ Villarroel 170
08036 Barcelona
Tel: 93 227 55 74
Fax: 93 451 44 38

3.7. CENTER WHERE THE ASSAY WILL BE CONDUCTED

- **Experimental phase:** Inther Unit, Hospital Clínic de Barcelona
  Staircase 2, floor 3. C/ Villarroel, 170, 08036, Barcelona.

- **Analytical phase:** Central Laboratory of the Hospital Clínic: CDB ("Center de Diagnòstic Biomèdic") Hospital Clínic de Barcelona. C/ Villarroel, 170, 08036, Barcelona.
- **Statistic analysis:** Elisa de Lazzari. Infectious Diseases Service, Hospital Clínic de Barcelona. C/ Villarroel, 170, 08036, Barcelona. Tel: 93 227 54 00 / Extension 2790 / Fax: 93 451 54 24.
- **Committee approving the trial**: Clinical Research Ethics Committee of the Hospital Clínic de Barcelona (CEIC Hospital Clínic).
- **Laboratory for sample and security analytics:** Central Laboratory of the Hospital Clínic: CDB ("Center de Diagnòstic Biomèdic"). C/ Villarroel, 170, 08036, Barcelona.

3.8. ESTIMATED DURATION OF THE TEST

The clinical phase of the study will last approximately 24 weeks, time necessary to recruit and review the volunteers (8 weeks) and perform the two experimental periods (16 weeks). The analysis of the data will take 12 more weeks.

4. JUSTIFICATION AND OBJECTIVES

4.1. JUSTIFICATION

Antiretroviral treatment has to be used indefinitely to achieve a sustained inhibition of HIV replication so that immunity is preserved and no clinical consequences appear. Although current antiretroviral therapy has changed, the prognosis of HIV infection from a fatal disease to a chronic disease, antiretroviral drugs can cause toxicity. One of the complications of antiretroviral treatment that has generated most interest in recent years is metabolic toxicity, manifested as dyslipidemia, insulin resistance, and / or mitochondrial toxicity (1, 2). The consequences of metabolic toxicity are diabetes and cardiovascular disease. Both diseases seem more frequent in patients infected with HIV than in the general population (3, 4), so there is great interest in getting antiretroviral treatment does not increase this risk. Until now, it is known that certain drugs that are members of the families most frequently used in antiretroviral treatment may be associated with some type of metabolic toxicity. Some nucleoside analogs such as thymidine analogues (zidovudine and stavudine) or didanosine have been associated with the risk of dyslipidemia (5), diabetes mellitus (6, 7), and mitochondrial toxicity in the form of lactic acidosis (8) or of other manifestations; Abacavir has been associated with an increased risk of myocardial infarction due to a mechanism not yet known (9). Protease inhibitors, which require potentiation with low doses of ritonavir, are usually associated with dyslipidemia (5) and have been associated with a higher cardiovascular risk than non-nucleosides (10). Efavirenz is the most commonly used drug in the non-nucleoside family and has been associated with an increase in total cholesterol and LDL

more intense than nevirapine, the other non-nucleoside drug currently marketed (11). However, the benefits of current antiretroviral treatment far outweigh the possible risks of metabolic toxicity, both in terms of progression to AIDS and paradoxically in terms of general health not apparently related to HIV infection such as kidney, liver and even cardiovascular disease (12).

HIV infection itself is associated with a decrease in total cholesterol and its fractions and with a progressive increase in triglycerides as immunosuppression progresses (13, 14). These effects are reversed in part by the effective antiretroviral treatment, but as previously mencioned, some drugs may promote certain lipid alterations per se. Moreover, HIV infection is also associated with mitochondrial dysfunction per se (15). Therefore, the study of the potential effect of a specific antiretroviral drug on the lipid metabolism, insulin sensitivity, or mitochondrial function should contemplate that. This study should be performed in healthy volunteers to avoid bias.

Raltegravir is the first drug in a new family, integrase inhibitors, whose efficacy in salvage treatment has been demonstrated (16). In addition, its profile of tolerability and safety is very promising because it is metabolized in a different way to that of cytochrome P450 (glucuronidation), which avoids interactions of potential clinical importance and acts on the integrase which is an enzyme that does not exist naturally in the healthy human being, what makes a specific toxicity very unlikely due the lack of this target. In patients with antiretroviral treatment failure, where active raltegravir or placebo was administered together with other drugs, it was not shown that there was more incidence of dyslipidemia or other side effects in one of the two arms with respect to the other (17). In naive patients, the administration of raltegravir against efavirenz, in addition to tenofovir and emtricitabine, showed greater increases in lipids in those treated with efavirenz against minimal changes in lipid parameters in those treated with raltegravir (18).

Our hypothesis is that treatment with raltegravir is not associated with dyslipidemia, insulin resistance, or mitochondrial dysfunction. For this, we have determined the safety profile of this product (already available in the Spanish Market) we have designed the study of the metabolic effects in health volunteers. Metabolic tolerability studies of antiretroviral drugs in healthy volunteers are not new. These types of study have shown that some protease inhibitors such as indinavir and lopinavir/ritonavir can induce hyperlipidemia and insulin resistance per se (19, 20). As previously stated, studies in healthy volunteers allow to avoid the bias implied by HIV infection itself, since even in the absence of antiretroviral treatment, it may cause an alteration of plasma lipids, insulin sensitivity and mitochondrial dysfunction evidenced by a depletion of mitochondrial DNA, effects mediated in part by HIV infection itself (13, 15). Studies with antiretrovirals in healthy volunteers are conducted for a short period of time, from a single dose to a period of 4 weeks (19-24). This duration is justified by the ethical and technical difficulties of a longer period of study and by the fact that a possible metabolic impact would become evident immediately (25). The determination of plasma lipids and

the oral glucose tolerance test have been tests that have been used in these studies; both are simple and can be performed in any hospital center. Although more complex tests have also been performed in these studies for the determination of insulin sensitivity as the hyperinsulinemic euglycemic clamp, the correlation that showed this more sophisticated test with the oral glucose tolerance test was narrow.

The relative decrease in the amount of mitochondrial DNA with respect to nuclear DNA is the most widely used test to demonstrate mitochondrial toxicity of antiretroviral drugs. Although there is no data on the short-term impact (days) of the modification of antiretroviral treatment or its suspension on mitochondrial DNA, in vitro studies with NARTIs at therapeutic doses for periods of less than one week have allowed demonstrate a decrease in mitochondrial DNA as a marker of mitochondrial toxicity (26). Therefore, we hope if the treatment with raltegravir has some repercussion on the mitochondrial DNA, this could be evident. The determination of mitochondrial DNA is a more complex test than the determination of plasma lipids or the performance of an oral glucose tolerance test, although our center has extensive experience in carrying out this test. Our hypothesis is that treatment with raltegravir will not cause clinically evident metabolic alterations on plasma lipids or insulin sensitivity nor will it produce a reduction in mitochondrial DNA content.

4.2. OBJECTIVES

4.2.1. Main objective

The objective of this project is to determine if the administration of raltegravir (Isentress®) during 7 days, at the usual doses of 400 mg/12h, causes changes in lipid metabolism.

4.2.2. Secondary objectives

As a secondary objective, it will be evaluated whether this treatment regimen causes modifications in the metabolism of glucose and mitochondrial DNA.

5. TYPE OF CLINICAL TRIAL AND DESIGN

This is a pharmacodynamics-phase I clinical trial in healthy volunteers, to evaluate the effects of raltegravir (antiretroviral authorized for HIV) on lipid and hydrocarbon metabolism and mitochondrial function. The study, unicentric, has a double-blind, placebo-controlled design, masked, crossed (two formulations, Isentress® and placebo, and two treatment periods) and with random assignment of the treatment sequence. Both formulations, Isentress® and placebo will be administered orally every 12 hours for 7 days. All the participants will receive both treatments (crossed) leaving between them a washing period of at least 1 week to avoid a "carry over" effect. After the selection and review of the volunteers over three weeks, they will participate in two experimental periods, separated by a washout period of at least 1 week. In one of the two periods the participants will receive 7 days of treatment with raltegravir (400 mg,

every 12 hours orally) and in the other period they will be given the placebo, every 12 hours, also for 7 days and orally. All participants will receive both treatments, the order of the treatment sequence (raltegravir-placebo or placebo-raltegravir) will be decided randomly.

6. SELECTION OF SUBJECTS

Fourteen healthy volunteers, all male, who have previously been informed of the study and its risks, and who have signed the consent form will be included. They will be given a clinical history, a physical examination, an ECG and a blood test (biochemistry, hematology, urine, serologies for hepatitis B and C and HIV, drugs of abuse in urine). (See point 8.1.).

6.1. SAMPLE SIZE

The number of subjects was determined based on the hypothesis that the study drug will not cause changes in lipid metabolism. Elevations ≥25 mg/dl in the values of total cholesterol with respect to baseline values will be considered significant and indicative of lipid metabolism alteration. Based on this and using the values of interindividual variability in healthy population cholesterol levels (data from previous studies conducted with healthy volunteers) which is around 30 mg/dL, it was obtained that (with a power of 80 % and an alpha error of 0.025, for a unilateral test) will require 14 subjects to detect elevations of cholesterol levels ≥25 mg/dl.

6.2. INCLUSION CRITERIA

Those people who meet the following criteria will be included in the study:

- Healthy male subjects.
- Age between 18 and 45 years.
- Body mass greater than 19 and less than 25 kg/m2.
- Non-pathological clinical history.
- Findings of the physical examination, normal or without clinical relevance.
- Analytical findings, normal or without clinical relevance.
- Serology hepatitis B, Hepatitis C and HIV negative.
- Analysis of drugs in negative urine.
- The subject understands and accepts the trial procedures and gives written approval informed consent.

6.3. EXCLUSION CRITERIA

Those subjects who:

- Do not meet the above requirements.
- Suffering from organic disorders or have undergone major surgery in the 90 days prior to the start of the trial.
- Have a serious psychiatric history.
- History of dyslipidemia.

- Consumption of more than 30g of alcohol/day.
- Smokers (smoker is considered ≥1 cigarette, cigar or pipe a day).
- They consume a significant amount of food or drinks with xanthines (more than five units of coffee, tea or cola a day).
- Have been under medical treatment in the 30 days prior to inclusion in the study and/or any symptomatic medication in the 7 days prior to the start of the trial.
- Have participated in another clinical trial during the 3 months prior to the start of the present study.
- History of liver disease, kidney disease and any other disorder that may alter the absorption, metabolism distribution or excretion of the drug.
- History of drug allergy.
- Another type of non-drug allergy that is clinically relevant.
- Subjects with a history of myopathy and rhabdomyolysis

6.4. DIAGNOSTIC CRITERIA FOR PATHOLOGIES IN STUDY

Not applicable since it is a study in healthy volunteers (see inclusion and exclusion criteria).

6.5. WITHDRAWALS AND ABANDONMENTS

Participants may leave the study at any time they wish without specifying the reasons. All those persons who present adverse reactions that, in the opinion of the researcher, endanger their health, and those who demonstrate lack of collaboration or transgressions of the study norms will be withdrawn from the study. The reasons for dropouts or withdrawals from the study, if any, should also be included in the corresponding CRD sheets. The abandonments for non-medical reasons will be replaced by new cases so that the number of inclusions is the one prefixed in each treatment group. Only the results of the volunteers who conclude the study will be considered, except those who withdraw from the study due to alterations in some of the variables of assessment of lipid metabolism, glucose and mitochondrial toxicity. The data of all those who have participated, whether finalized or not, in the study will be duly collected in the CRDs.

6.6. TREATMENT OF LOSS PRERANDOMIZATION

Not applicable.

6.7. RECRUITMENT PERIOD

In the three weeks prior to the start of the first experimental period (administration of the drugs under study/placebo) the volunteers will be selected by clinical history and physical examination, EKG, blood and urine tests.

7. DESCRIPTION OF THE TREATMENT

7.1. DOSAGE, DOSAGE, ROUTE OF ADMINISTRATION AND FORMAT

The subject will receive the two formulations (raltegravir and placebo) in two different periods (Period 1 and 2) separated by at least one week of washing-out period. Each period will last 7 days, where volunteers will receive two doses per day (one every 12 h) of active treatment (raltegravir 400 mg) or placebo orally. This means that participants will receive a first period of treatment with raltegravir or placebo for 7 days with a regimen of 400 mg every 12 hours (Period 1). After the 7 days of treatment there will be a washout period (participants do not receive any medication) of 1 week ($t_{1/2\beta}$ of raltegravir: 9 hours). After washing-out period, participants will receive the other treatment (Period 2) with a schedule, dosage and duration identical to period 1 but those volunteers who received raltegravir in the first period, will receive placebo in the second and vice versa. The order in which the participants will receive the two treatments (raltegravir-placebo or placebo-raltegravir) will be established randomly (see point 7.2). The treatments will be administered in the Inther Unit by the research staff to ensure compliance. The administration of the medication will take place at 9 a.m. and 9 p.m. Both treatments (active and control) will be administered orally.

7.2. ASSIGNMENT OF TREATMENT

All participants will receive both treatments (raltegravir and placebo). The allocation of the treatment sequences (raltegravir-placebo or placebo-raltegravir) will be done in a randomized and balanced manner. The randomization list will be prepared through a computer program by the CTU (Clinical Trials Unit) of the Clinical Pharmacology Service of the Hospital Clínic of Barcelona. The CTU will provide the Trials Agency of the Hospital Clínic with the random assignment list so that they can proceed with the preparation of the medication for the study, which is administered following a double-blind scheme (see point 7.3). It will also provide, in a sealed envelope, the code of random assignment of each participant to the Principal Investigator.

7.3. PREPARATION OF THE STUDY MEDICATION

The preparation of the medication will be carried out by specialized staff of the Agency of Clinical Trials Units of the Hospital Clínic of Barcelona.

As this is a double-blind trial, the CTU will provide the staff of the Trial Agency with the list of random assignments to the treatment sequence (as specified in point 7.2.). Based on this list, the personnel of the Agency will be in charge of dispensing the corresponding medication for each volunteer and study period. The label will contain the following information:

- Study
- Code
- Promoter
- Principal Investigator
- No Volunteer (from 1 to 14)

- Period 1 or 2

7.3.1. Instructions for the preservation of Isentress® and placebo

This drugs do not require special conditions of conservation.

7.3.2. Masking

A double-blind masked trial will be conducted. All patients will receive in one order or another, the two treatments:

- Experimental treatment: Isentress®
- Control drug: Placebo

The tablets will be identical and indistinguishable from each other. MSD will provide the active product and its corresponding placebo.

Once the study medication is prepared, the staff of the Trial Agency of the Hospital Clínic of Barcelona will deliver it to the principal investigator for its administration to the participating volunteers.

7.4. STORAGE AND HANDLING

Special storage conditions for the medication under study are required.

7.5. ADMINISTRATION OF STUDY MEDICATION

The administration of the study treatments will be carried out in the presence of one of the investigators who must write down the time of administration in the corresponding register.

The medication will be administered at 9 a.m. and 9 p.m. (the pattern is one shot every 12 hours). In order to verify that there have been no medication errors, the researcher responsible of the administration it will copy in the register, on the corresponding page, the control digit of the label.

7.6. CONCURRING TREATMENTS

During the trial, additional medications are not authorized. Exceptionally, acetaminophen will be allowed. Other drugs for symptomatic treatment will be allowed, at a single dose, in case it is administered during the bleaching period and if it is not expected that this drug could interfere in the evaluation of cholesterol levels and other parameters of the trial. Dose, time and cause will be recorded in the corresponding sheets of the register of the subject in question. The principal investigator, together with the monitor, will assess the possible exclusion of the volunteer in case any of the aforementioned conditions is not met.

The minimum time that should elapse from the suspension of any prescription treatment until the inclusion of the subject in the study will be 30 days. In the case of single-dose symptomatic therapy, it will be allowed up to one week before the study day, assuming that the drug has been completely eliminated.

7.7. ASSESSMENT OF THE COMPLIANCE

The administration of the study medication to the volunteer participating in the study will be directly observed by the researcher, ensuring that the volunteer receives all doses.

7.7.1. Assessment of compliance with the diet

To verify that the volunteers have followed the recommended diet, they will be provided with a notebook in which they should write down what they eat each day. The notebook will be checked by the investigators daily (at the time to receive the study medication).

8. DEVELOPMENT OF THE ASSAY AND EVALUATION OF THE RESPONSE

8.1. SELECTION OF VOLUNTEERS

In the 3 weeks prior to the start of the study, volunteers will be selected by clinical history, physical examination, EKG, blood and urine tests. The general blood test will consist of a biochemical profile, blood count, coagulation, serology, elemental analysis of urine and determination of drugs of abuse in urine. The selection tests will be carried out to as many people as necessary to get 14 subjects that meet all the inclusion criteria and none of the exclusion criteria.

8.1.1. Selection tests

The selection tests will be based on:

- Information and signature of informed consent.
- Clinical history: filiation, allergies, pathological history, toxic habits, anamnesis per systems.
- Complete physical examination, including height, weight and calculation of BMI.
- Vital signs collection: Axillary temperature, heart rate and blood pressure after 5 minutes of sitting rest.
- 12-lead ECG.
- Blood biochemistry: glucose, urea, creatinine, sodium, potassium, total bilirubin, AST (GOT), ALT (GPT), gamma-GT (GGT), alkaline phosphatase, calcium, phosphorus, total proteins, cholesterol, HDL cholesterol, cholesterol LDL, triglycerides and lactate.
- Hemogram: red blood cells, hemoglobin, hematocrit, MCV, HCM, CCMH, count and leukocyte formula.
- Serologies: determinations for the hepatitis B virus, hepatitis C and HIV.
- Urine test: density, pH, glucose, ketone bodies, bilirubin, urobilinogen, proteins, red blood cells/hemoglobin, leukocytes and nitrites.

- Drugs of abuse in urine.

Blood tests will be performed with the volunteers fasting for 12 hours.

All volunteers who, after the selection tests, meet all the inclusion criteria and do not meet any of the exclusion criteria, will be included in the study.

8.2. STUDY DEVELOPMENT

All subjects included in the trial will participate in two experimental periods (period 1 and period 2) separated by one week of washing-out period. Both experimental periods will be identical, following the same development scheme.

The 14 volunteers that are included in the study (after the screening tests have been carried out) will begin, on Day 1 of Period 1, a standard diet (equal for all) with the intention of minimizing alterations in cholesterol levels. This diet maintains the proportion of 10-15% of proteins, 50-60% of carbohydrates and 30-35% of fats, as recommended by the WHO. To facilitate the following-up of this, the Dietetic Service of the Hospital Clínic of Barcelona will elaborate different menu options.

After 7 days of the standard diet, on day 8 of Period 1, the volunteers will undergo a blood test identical to that of the selection, plus a standard oral glucose tolerance test and a mitochondrial DNA quantification test. These are baseline tests before the administration of the study medication. The day after the pretreatment blood test (day 9 of Period 1), the volunteers will receive the corresponding medication according to the randomization sequence, raltegravir or placebo (see point 7.3). Since then and for 7 days, volunteers must go to the Inther Unit of the Hospital Clínic every 12 hours (approximately at 9 a.m. and 9 p.m.) where they will take a 400 mg dose of raltegravir or placebo orally. During all this time the volunteers should follow the standard diet.

After 7 days of treatment (day 16 of Period 1), volunteers will perform a blood test identical to the baseline (day 8 of Period 1) to assess possible changes in lipid and hydrocarbon metabolism, and mitochondrial function due to the treatment. Once the first period of the study ends, the standard diet ends, a wash-out period of at least 1 week is then started.

After the washing period, Period 2 will start identical to Period 1: 7 days of standard diet, pretreatment blood test (identical to days 8 and 16 of Period 1), 7 days of treatment, blood test after finishing the treatment. Thus, end of diet and end of the study.

Both periods of the study are identical, the only thing that varies is the order in which they receive the study treatment, that is, some will receive raltegravir in Period 1 and placebo in Period 2, while others will receive placebo in Period 1 and raltegravir in Period 2. The assignment to the treatment sequence in Period 1 and Period 2 will be carried out as specified in point 7.2. The administration of the medication by the investigators will be done double-blind.

All the analytical controls of the study will be carried out after 12 hours of fasting.

In case the deviations of the results are found in the last visit, the volunteer will be followed up until its normalization.

The two study periods are identical in terms of methodology, varying only the medication that participants will receive in each of them.

8.2.1. Safety

In addition to evaluating pharmacodynamic parameters (main objective of the study), tolerability and safety data will be collected. Before the administration of each of the doses of the treatments under study, blood pressure and heart rate will be recorded (after 5 minutes of sitting rest) and they will be asked about the appearance of adverse events by direct question. (How are you? Have you noticed any discomfort?). Likewise, possible adverse events will be recorded when the subject verbalizes them spontaneously, or is evident to the researchers (see section 9). All those analytical alterations that are observed in any of the visits that will be carried out throughout the study (specified in the previous section), will be recorded as an adverse event according to the WHO Toxicity scale, for the classification of acute or subacute toxic effects.

8.3. SPECIFIC RULES FOR VOLUNTEERS

Volunteers will be given, during the selection visit, a sheet containing all the recommendations to follow and a menu for the standard diet during the specified days, which they must commit to comply during the entire duration of the study. They will also be given a calendar with the activities to do during the entire study. The recommendations or rules to follow are the following:

- You must go to the Inther Unit on the days indicated by the researcher and that will appear on your calendar, at the indicated time, on time.
- A minimum of 8 h of night rest will be carried out throughout the duration of the study, except in the washout period, when it will not be mandatory.
- It is not allowed to perform intense physical exercise from the day the standard diet starts, each of the two study periods, and until the end of it.
- The intake of foods rich in xanthines (coffee, cola, tea or chocolate) or grapefruit juice is not allowed on the day that the standard diet is started each of the two periods of the study and until the end of the same, part of the amounts that come specified in the menu that has been delivered.
- Smoking and drinking alcoholic beverages are not allowed from the day the standard diet starts each of the two periods of the study and until the end of it.
- The ingestion of any drug is prohibited. If you need to take a drug as a symptomatic treatment, you must inform the researcher in advance
- It is recommended not to donate blood during the three months following the end of your participation in the study.
- For the proper development of this work it is essential that you follow the instructions provided by the researcher at all times, and that it be extremely timely.

8.4. CLINICAL ASSESSMENT CRITERIA AND EVALUATION

8.4.1 Main variable: Total serum cholesterol levels.

8.4.2. Secondary variables:

- Modifications of HDL and LDL cholesterol and triglycerides levels.
- Modifications of glycemic metabolism after an oral glucose tolerance test.
- Modifications of mitochondrial DNA.
- Safety: the safety of the study drug will be assessed by altering the analytical parameters and recording vital signs (see point 8.2.) as collecting adverse events. The adverse events manifested by the volunteers or evidenced by the researchers, and the subclinical alterations in blood and urine will be recorded.

9. ADVERSE EVENTS

9.1. DEFINITIONS

An adverse event is any unfavorable medical event that occurs in a patient or subject undergoing clinical research to which a pharmaceutical product is administered, regardless of whether or not it is related to the pharmaceutical product. Any sign (including the alteration of a laboratory parameter), symptom or illness, unfavorable and unintentional, which are temporarily associated with the use of a medicinal product may constitute an adverse event.

A **serious adverse** event is considered to be one that:

- Causes the death of the subject.
- Threatens the life of the subject.
- Produces permanent disability.
- Gives rise to hospitalization or prolongs it.
- Congenital anomalies and malignant processes will always be considered as serious events.

Unexpected adverse events are those experiences not described (in nature, severity or frequency) in the researcher's manual.

9.2. DESCRIPTION

The collection of adverse events should be carried out by the investigator (s) of the trial, describing the adverse event according to its duration, intensity, frequency and causality between the treatment and the adverse event.

The duration refers to the time elapsed from the occurrence of the event until its completion.

The intensity will be defined according to a scale of three degrees:

- **Slight**: Banal adverse events, of minor importance and short duration, which do not substantially affect the patient's life.
- **Moderate**: Adverse events that cause enough discomfort to interfere with the patient's normal life.
- **Severe**: Adverse events that imply an inability to work or perform the patient's usual activity and even force them to abandon the treatment.

The frequency will be defined as: punctual, recurrent (intermittent) or continuous (persistent).

The causal link of an adverse event with the study medication will be established according to the modified Karch and Lasagna classification, in which the following categories are defined:

- **Not related**:
  - Does not meet any of the criteria mentioned.
- **Unlikely**:
  - Reasonable temporal sequence
  - It does not coincide with the scheme of known adverse reactions
  - It can not be explained by the clinical situation of the subject
- **Possible**:
  - Reasonable time sequence
  - Coincides with the scheme of known adverse reactions.
  - It may be due to the clinical situation of the subject or to other drugs administered concomitantly.
- **Probable**:
  - Reasonable time sequence
  - Coincides with the scheme of known adverse reactions
  - Improvement by interrupting the administration of the drug
  - It is not explained by the clinical situation of the subject.
- **Certain**:
  - There is a reasonable temporal sequence in relation to the administration of the drug or its plasma or tissue levels.
  - The observed manifestation coincides with the scheme of known adverse reactions of the drug involved.
  - Improvement by interrupting the administration of the drug.
  - Reappears when administered again.

9.3. REGISTRATION

Possible adverse events will be recorded by the investigators as described in section 8.2.1, when they manifest spontaneously by the subjects, are communicated after a direct question from the investigator or are evident to the investigators. The information obtained will be noted in the corresponding section of the individual data collection

notebook for each subject, which will include the type of adverse event, its duration, intensity, frequency, final result, causality and the type of corrective measures, if they are used.

If adverse events (clinical or subclinical) are observed, the subjects will be monitored by the investigator during their evolution and until its resolution. An adverse event is considered to be that variation with respect to the reference values that, according to the WHO Toxicity scale for the classification of acute or subacute toxic effects, are within Grade II and successive. If the undesirable effects observed are clinically significant, the affected volunteer will be excluded from the trial.

9.4. NOTIFICATION

The investigator will notify the promoter of the study within 24 hours of serious or unexpected adverse events, by telephone or telefax. Within a maximum period of 72 hours, the Notification Sheet of serious or unexpected adverse events will be delivered to the promoter. If not all the information is available, it can be completed within 15 days. The promoter will notify the Spanish Agency for Medicines and Health Products and the Clinical Research Ethics Committees involved in the trial of serious and unexpected events that may be related with the research treatments. Information on expected serious adverse events, non-serious adverse events and those considered unrelated to the treatments under study will be included in a tabulated form in the final report of the clinical trial.

10. ETHICAL ASPECTS

10.1. GENERAL CONSIDERATIONS

At all times the clinical trial will respect the international ethical declarations of Helsinki (revised version of Edinburgh 2000), the recommendations of the WHO, the deontological code and those derived from the Spanish legislation on clinical trials (Medication Law 25/1990, Royal Decree 223/2004). Before including any subject in the study, the CEIC and the Spanish Agency for Medicines and Health Products must approve and authorize respectively the study protocol, the information that will be given to the subject and the model of informed consent that will be used. The study will be conducted under the Good Clinical Practice standards.

10.2. INFORMATION AND INFORMED CONSENT

The volunteers will be informed about the objectives, development and scope of the study by the principal investigator or collaborators, and will also receive the consent form where they will have in written the characteristics of the trial. Before being included, all volunteers must sign the informed consent form.

10.3. CONFIDENTIALITY

The researchers agree to keep discretion about the progress of the trial. The protocol and its attached documentation as well as the data collection notebooks and all the information generated during the study are considered confidential. They can only be used by researchers for the specific purposes of this protocol. To preserve the confidentiality of the personal data of the subjects, only the principal investigator, his collaborators and the technical staff who participate in the study will have access. For the same reason, complete filiation data and written consent will be kept in the investigator's file. In accordance with the Organic Law 15/99 of December 13, Protection of Personal Data, in all cases the confidentiality of the data will be strictly maintained. Participants may exercise their right of cancellation, rectification, etc. as provided in the legislation.

10.4. INSURANCE POLICY

The promoter of the trial has an insurance that covers the incidents that may arise from the action of the drugs tested.

11. PRACTICAL CONSIDERATIONS

11.1. RESPONSIBILITIES OF THE PARTICIPANTS

The promoter, the monitor and the researcher will comply with the obligations described in articles 35, 36 and 37 of RD 223/2004 of February 6.

11.2. CORRECTIONS

The corrections of any data in the data collection register should be made by drawing a straight line on the incorrect data (allowing it to be readable), next to it the correct data will be noted. The corrections must always be accompanied by the corresponding clarification signed and dated by the researcher.

11.3. STORAGE OF THE ASSAY DOCUMENTATION

The promoter is responsible for the storage of the trial documentation. Will comply with minimum times that are mentioned in the Royal Decree on Clinical Trials. The investigator will be responsible for keeping the identification codes of the subjects for at least fifteen years after the conclusion or interruption of the trial. The clinical records of the subjects and other original data will be kept for the maximum period of time allowed by the hospital, the institution or the private consultation where the trial was conducted.

11.4. CONDITIONS OF PUBLICATION

All individual and collective data, as well as the results derived from them, will be confidential, and may not be disseminated, commented or published without the knowledge and authorization of the trial promoter.

11.5. INTERRUPTION OF THE ASSAY

The clinical trial may be interrupted at the discretion of the principal investigator and / or the promoter at any time.

12. STATISTICAL ANALYSIS

A report on the statistical analysis of the data will be included in the final report.

12.1 ANALYSIS OF THE MAIN VARIABLE

With the main variable, a descriptive analysis will be made with mean and standard deviation (SD) in case the variable is distributed normally, otherwise it will be described with median and interquartile range.

The period, treatment and sequence effect will be evaluated with the variance test (ANOVA). To verify that there is no "carry-over" effect, a Student's T test will be performed on the ratio and absolute values in both periods, to verify that the means between the two periods are the same in both sequences.

To assess the magnitude of the change in total cholesterol during each period, this variable will be transformed into a ratio (final value/initial value), so this quotient will show an appreciation of the effect of the change between the values at the beginning of the period with respect to the values of the end of the period.

It was established that elevations of ≥25 mg/dl in the values of total cholesterol with respect to baseline values will be considered significant and indicative of impaired lipid metabolism. To assess this aspect, the difference between the final values of cholesterol in each period minus the initial values will be made.

12.2. ANALYSIS OF SECONDARY VARIABLES

12.2.1. Secondary variables.

For the secondary variables triglycerides, fractions of HDL and LDL cholesterol, oral glucose tolerance test and mitochondrial DNA, a description will be made with means and standard deviation (SD) in case they show a normal distribution or with medians and interquartile range. A Student`s T test will be made to all the variables to verify the existence of a "carry-over" effect.

For the variables triglycerides and HDL and LDL cholesterol, the magnitude of change between both periods will be evaluated as a percentage.

A variance test (ANOVA) will be performed to evaluate if there was a period, treatment or sequence effect.

The values of the oral glucose tolerance test curves will be classified according to their values: not altered (glucose at 120 minutes <140mg/dL) or altered (glucose at 120 minutes >140 mg/dL).

In each period and in each sequence, a contingency table (2 x 2) will be made and the number of volunteers that show changes in the state of the curve will be described.

12.2.2. Tolerability analysis

The individual values grouped by treatment received will be tabulated, applying a descriptive analysis (calculation of means and standard deviations) for the quantitative variables (blood pressure and heart rate), and frequencies for the qualitative variables (notification of symptoms). The clinical and analytical alterations will be analyzed individually, as well as considering their meaning in the treatment group. In any case, its relevance in terms of "clinically relevant changes" will be assessed. The adverse events observed during the development of the study will be described and discussed, specifying its duration, intensity, frequency and causality (as described in section 9.2).

12.2.3. Size of the sample

The number of subjects was determined based on the hypothesis that the study drug would not cause changes in lipid metabolism. Elevations ≥25 mg/dl in the values of total cholesterol with respect to baseline values would be considered significant and indicative of lipid metabolism alteration. Based on this and using the values of interindividual variability in healthy population cholesterol levels (data from previous studies conducted with healthy volunteers) which is around 30 mg/dL, it was obtained that (with a power of 80 % and an alpha error of 0.025, for a unilateral test) 14 subjects were needed to detect elevations of cholesterol levels ≥25 mg/dl.

13. BIBLIOGRAPHY

1. Havlir DV, Currier JS. Complications of HIV disease and antiretroviral therapy. Top HIV Med 2006; 14: 27-35
2. Lundgren JD, Battegay M, Behrens G, et al. European AIDS Clinical Society (EACS) guidelines on the prevention and management of metabolic diseases in HIV. HIV Medicine 2008; 9: 72-81
3. Currier JS, Boyd F, Kawabata H, Dezii C, Burtcel B, Hodder S. Diabetes Mellitus in HIV- Infected Individuals. En: Program and Abstracts of the 9th Conference on Retroviruses and Opportunistic Infections, Seattle Washington 2002 Feb 24-28; abstract no. 677-T
4. Currier JS, Taylor A, Boyd F, et al. Coronary heart disease in HIV-infected individuals. J Acquir Immune Defic Syndr. 2003; 33: 506-512
5. Oh J, Hegele RA. HIV-associated dyslipidemia: patogenesis and treatment. Lancet Infect Dis 2007; 7: 787-796
6. De Wit S, Sabin C, Weber R, et al. Relationship between the use of stavudine and new onset diabetes mellitus: the DAD study. 8th International Congress on drug Therapy in HIV Infection, Glasgow 2006; abstract PL9.5
7. Ledergerber B, Furrer H, Rickenbach M, et al. Factors associated with the incidence of type 2 diabetes mellitus in HIV-infected participants in the Swiss HIV Cohort Study. Clin Infect Dis 2007; 45: 111-119.
8. Lactic Acidosis International Study Group. Risk factors for lactic acidosis and severe hyperlactataemia in HIV-1-infected adults exposed to antiretroviral therapy. AIDS 2007; 21: 2455-2464.
9. DAD Study Group. Use of nucleoside reverse transcriptase inhibitors and risk of myocardial infarction in HIV-infected patients enrolled in the DAD study: a multi-cohort collaboration. Lancet 2008; 371: 1417-1426.
10. DAD Study Group. Class of Antiretroviral Drugs and the Risk of Myocardial Infarction. N Engl J Med 2007; 356: 1723-1735.
11. Van Leth F, Phanuphak P, Stroes E, Gazzard B, Cahn P, et al. Nevirapine and efavirenz elicit different changes in lipid profiles in antiretroviral-therapy-naive patients infected with HIV-1. PLoS Medicine Vol. 1, No. 1, e19 doi:10.1371/journal.pmed.0010019.
12. The Strategies for Management of Antiretroviral Therapy (SMART) Study Group . CD4+ Count–Guided Interruption of Antiretroviral Treatment. N Engl J Med 2006; 355: 2283-2296.
13. Sellmeyer DE, Grunfeld C. Endocrine and metabolic disturbances in human immunodeficiency virus infection and the acquired immune deficiency síndrome. Endocr Rev1996; 17: 518-532.

14. SA, Smit E, Cole SR, et al. Impact of HIV infection and HAART on serum lipids in men. JAMA 2003; 289: 2978-2982.

15. Miró O, López S, Martínez E, et al. Mitochondrial effects of HIV infection on peripheral blood mononuclear cells in HIV-infected patients never treated with antiretrovirals. Clin Infect Dis 2004; 39:710–716.

16. Cooper D, Gatell J, Rockstroh J, et al for the BENCHMRK-1 Study Group. Results of BENCHMRK-1, a Phase III Study Evaluating the Efficacy and Safety of MK-0518, a Novel HIV-1 Integrase Inhibitor, in Patients with Triple-class Resistant Virus. En: Program and Abstracts of the 14th Conference on Retroviruses and Opportunistic Infections, Los Angeles California 2007 Feb 25-28; abstract no. 105aLB.

17. Grinsztejn B, Nguyen BY, Katlama C, et al. Safety and efficacy of the HIV-1 integrase inhibitor raltegravir (MK-0518) in treatment-experienced patients with multidrug-resistant virus: a phase II randomised controlled trial. Lancet 2007; 369: 1261-1269.

18. Markowitz M, Nguyen BY, Gotuzzo E, et al. Rapid and durable antiretroviral effect of the HIV-1 integrase inhibitor raltegravir as part of combination therapy in treatment-naive patients with HIV-1 infection: results of a 48-week controlled study. J Acquir Immune Defic Syndr 2007; 46: 125-133.

19. Noor MA, Seneviratne T, Aweeka FT, et al. Indinavir acutely inhibits insulin-stimulated glucose disposal in humans : a randomized, placebo-controlled study. AIDS 2002 ; 16 : F1-F8.

20. Noor MA, Parker RA, O'Mara E, et al. The effects of HIV protease inhibitors atazanavir and lopinavir/ritonavir on insulin sensitivity in HIV-seronegative healthy adults. AIDS 2004 ; 18 : 2137-2144.

21. Noor MA, Flint OP, Maa JF, Parker RA. Effects of atazanavir/ritonavir and lopinavir/ritonavir on glucose uptake and insulin sensitivity: demonstrable differences in vitro and clinically. AIDS 2006; 20: 1813-1821.

22. Lee GA, Lo JC, Aweeka F, et al. Single-dose lopinavir-rionavir acutely inhibits insulin- mediated glucose disposal in healthy volunteers. Clin Infect Dis 2006; 43: 658-660

23. Dube M, Shen C, Greenwald ML, Mather K. Effects of 4 weeks of atazanavir, lopinavir/ritonavir or placebo on endothelial function and insulin sensitivity in healthy men. Antiviral Therapy 2007; 12 Suppl 2: L14.

24. Villarroel MC, Martinez E, Riba N, et al. Lack of evidence of metabolic abnormalities and mitochondrial toxicity with enfuvirtide: a double-blind, placebo-controlled, cross-over study with random sequence assignation in healthy adult volunteers. Antiviral Therapy 2007; 12 Suppl 2: L57.

25. Stein EA, Black DM. Lipoprotein changes with statins. Curr Atheroscler Rep 2002;4: 14-18 26. Pan-Zhou XR, Cui L, Zhou XJ, Sommadossi JP,1, Darley-Usmar VM. Differential Effects of Antiretroviral Nucleoside Analogs on Mitochondrial Function in HepG2 Cells . Antimicrob Agents Chemother 2000; 44: 496-503.
