# Supplementary material for: Metabolic, mitochondrial, renal and hepatic safety of enfuvirtide and raltegravir antiretroviral administration: Randomized crossover clinical trial in healthy volunteers
Source: PLoS One. 2019 May 23;14(5):e0216712. doi: 10.1371/journal.pone.0216712 (PMC6532851; doi:10.1371/journal.pone.0216712)

```
 GET
  FILE='/Users/SergioBarroso/Desktop/T20-RAL Reviewers/Base de datos/T20.sav'.
DATASET NAME Conjunto_de_datos1 WINDOW=FRONT.
DESCRIPTIVES VARIABLES=CHOL.PRE.P CHOL.POST.P CHOL.PRE.T20 CHOL.POST.T20 HDL.PRE.P HDL.POST.P [illegible]
PRO.POST.P PRO.PRE.T20 PROT.POST.T20
  /STATISTICS=MEAN STDDEV SEMEAN.
```

Descriptivos

Notas

Resultados creados		29-MAR-2019 00:32:43
Comentarios		
Entrada	Datos	/Users/SergioBarroso/Desktop/T20-RAL Reviewers/Base de datos/T20.sav
	Conjunto de datos activo	Conjunto_de_datos1
	Filtro	<ninguno>
	Peso	<ninguno>
	Dividir archivo	<ninguno>
	Núm. de filas del archivo de trabajo	12
Manipulación de los valores perdidos	Definición de los perdidos	Los valores perdidos definidos por el usuario son considerados como perdidos.
	Casos utilizados	Se han utilizado todos los datos no perdidos.
Sintaxis		DESCRIPTIVES VARIABLES=CHOL.PRE.P CHOL.POST.P CHOL.PRE.T20 CHOL.POST.T20 HDL.PRE.P HDL.POST.P HDL.PRE.T20 HDL.POST.T20 LDL.PRE.P LDL.POST.P LDL.PRE.T20 LDL.POST.T20 TAG.PRE.P TAG.POST.P TAG.PRE.T20 TAG.POST.T20 GLU.PRE.P GLU.POST.P GLU.PRE.T20 GLU.POST.T20 mtDNA.PRE.P mtDNA.POST.P mtDNA.PRE.T20 mtDNA.POST.T20 CRT.PRE.P CRT.POST.P CRT.PRE.T20 CRT.POST.T20 AST.PRE.P AST.POST.P AST.PRE.T20 AST.POST.T20 ALT.PRE.P ALT.POST.P ALT.PRE.T20 ALT.POST.T20 BIL.PRE.P BIL.POST.P BIL.PRE.T20 BIL.POST.T20 PROT.PRE.P PRO.POST.P PRO.PRE.T20 PROT.POST.T20 /STATISTICS=MEAN STDDEV SEMEAN.
Recursos	Tiempo de procesador	00:00:00.01
	Tiempo transcurrido	00:00:00.00

```
[Conjunto_de_datos1] /Users/SergioBarroso/Desktop/T20-RAL Reviewers/Base de datos/T20.sav
```

Estadísticos descriptivos

	N	Media		Desv. típ.
	Estadístico	Estadístico	Error típico	Estadístico
CHOL.PRE.P	12	181.8333	10.94327	37.90858
CHOL.POST.P	12	173.1667	11.55542	40.02916
CHOL.PRE.T20	12	183.9167	13.45951	46.62512
CHOL.POST.T20	12	172.4167	10.12756	35.08291
HDL.PRE.P	11	50.4545	2.83601	9.40599
HDL.POST.P	12	48.0833	2.61829	9.07001
HDL.PRE.T20	12	48.2500	2.20236	7.62919
HDL.POST.T20	12	49.4167	2.59504	8.98947
LDL.PRE.P	11	110.6364	7.56700	25.09690
LDL.POST.P	12	108.0000	8.92647	30.92219
LDL.PRE.T20	12	118.3333	11.36337	39.36388
LDL.POST.T20	12	104.4167	8.12167	28.13428
TAG.PRE.P	12	85.0833	14.64554	50.73363
TAG.POST.P	12	85.2500	13.08777	45.33737
TAG.PRE.T20	12	86.6667	10.76001	37.27376
TAG.POST.T20	12	92.7500	13.80389	47.81807
GLU.PRE.P	12	81.0000	2.36130	8.17980
GLU.POST.P	12	82.8333	2.40528	8.33212
GLU.PRE.T20	12	82.5000	1.97139	6.82908
GLU.POST.T20	12	84.7500	1.66572	5.77022
mtDNA.PRE.P	12	.7129	.07962	.27581
mtDNA.POST.P	12	.6251	.06851	.23734
mtDNA.PRE.T20	12	.6203	.05365	.18585
mtDNA.POST.T20	12	.6824	.07028	.24345
CRT.PRE.P	12	1.1500	.02611	.09045
CRT.POST.P	12	1.1417	.02876	.09962
CRT.PRE.T20	11	1.1091	.03426	.11362
CRT.POST.T20	11	1.1545	.02473	.08202
AST.PRE.P	12	26.0833	2.77832	9.62439
AST.POST.P	12	23.5000	1.77312	6.14225
AST.PRE.T20	11	23.4545	1.89432	6.28273
AST.POST.T20	11	23.7273	1.54385	5.12037
ALT.PRE.P	12	23.3333	1.52918	5.29723
ALT.POST.P	12	21.1667	1.34183	4.64823
ALT.PRE.T20	11	22.2727	2.43494	8.07578
ALT.POST.T20	11	23.6364	2.53080	8.39372
BIL.PRE.P	12	.9000	.09692	.33575
BIL.POST.P	12	.9667	.15635	.54160
BIL.PRE.T20	11	.7909	.04946	.16404
BIL.POST.T20	11	1.0727	.11839	.39266
PROT.PRE.P	12	74.5000	2.04680	7.09033
PRO.POST.P	12	74.2500	1.75432	6.07716
PRO.PRE.T20	11	74.2727	1.88820	6.26244
PROT.POST.T20	11	74.9091	2.08656	6.92033
N válido (según lista)	11			

```
 GET
  FILE='/Users/SergioBarroso/Desktop/T20-RAL Reviewers/Base de datos/T20.sav'.
DATASET NAME Conjunto_de_datos1 WINDOW=FRONT.
DESCRIPTIVES VARIABLES=CHOL.PRE.P CHOL.POST.P CHOL.PRE.T20 CHOL.POST.T20 HDL.PRE.P HDL.POST.P [illegible]
PRO.POST.P PRO.PRE.T20 PROT.POST.T20
  /STATISTICS=MEAN STDDEV SEMEAN.
```

Descriptivos

Notas

Resultados creados		29-MAR-2019 00:32:43
Comentarios		
Entrada	Datos	/Users/SergioBarroso/Desktop/T20-RAL Reviewers/Base de datos/T20.sav
	Conjunto de datos activo	Conjunto_de_datos1
	Filtro	<ninguno>
	Peso	<ninguno>
	Dividir archivo	<ninguno>
	Núm. de filas del archivo de trabajo	12
Manipulación de los valores perdidos	Definición de los perdidos	Los valores perdidos definidos por el usuario son considerados como perdidos.
	Casos utilizados	Se han utilizado todos los datos no perdidos.
Sintaxis		DESCRIPTIVES VARIABLES=CHOL.PRE.P CHOL.POST.P CHOL.PRE.T20 CHOL.POST.T20 HDL.PRE.P HDL.POST.P HDL.PRE.T20 HDL.POST.T20 LDL.PRE.P LDL.POST.P LDL.PRE.T20 LDL.POST.T20 TAG.PRE.P TAG.POST.P TAG.PRE.T20 TAG.POST.T20 GLU.PRE.P GLU.POST.P GLU.PRE.T20 GLU.POST.T20 mtDNA.PRE.P mtDNA.POST.P mtDNA.PRE.T20 mtDNA.POST.T20 CRT.PRE.P CRT.POST.P CRT.PRE.T20 CRT.POST.T20 AST.PRE.P AST.POST.P AST.PRE.T20 AST.POST.T20 ALT.PRE.P ALT.POST.P ALT.PRE.T20 ALT.POST.T20 BIL.PRE.P BIL.POST.P BIL.PRE.T20 BIL.POST.T20 PROT.PRE.P PRO.POST.P PRO.PRE.T20 PROT.POST.T20 /STATISTICS=MEAN STDDEV SEMEAN.
Recursos	Tiempo de procesador	00:00:00.01
	Tiempo transcurrido	00:00:00.00

[Conjunto_de_datos1] /Users/SergioBarroso/Desktop/T20-RAL Reviewers/Base de datos/T20.sav

Estadísticos descriptivos

	N	Media		Desv. típ.
	Estadístico	Estadístico	Error típico	Estadístico
CHOL.PRE.P	12	181.8333	10.94327	37.90858
CHOL.POST.P	12	173.1667	11.55542	40.02916
CHOL.PRE.T20	12	183.9167	13.45951	46.62512
CHOL.POST.T20	12	172.4167	10.12756	35.08291
HDL.PRE.P	11	50.4545	2.83601	9.40599
HDL.POST.P	12	48.0833	2.61829	9.07001
HDL.PRE.T20	12	48.2500	2.20236	7.62919
HDL.POST.T20	12	49.4167	2.59504	8.98947
LDL.PRE.P	11	110.6364	7.56700	25.09690
LDL.POST.P	12	108.0000	8.92647	30.92219
LDL.PRE.T20	12	118.3333	11.36337	39.36388
LDL.POST.T20	12	104.4167	8.12167	28.13428
TAG.PRE.P	12	85.0833	14.64554	50.73363
TAG.POST.P	12	85.2500	13.08777	45.33737
TAG.PRE.T20	12	86.6667	10.76001	37.27376
TAG.POST.T20	12	92.7500	13.80389	47.81807
GLU.PRE.P	12	81.0000	2.36130	8.17980
GLU.POST.P	12	82.8333	2.40528	8.33212
GLU.PRE.T20	12	82.5000	1.97139	6.82908
GLU.POST.T20	12	84.7500	1.66572	5.77022
mtDNA.PRE.P	12	.7129	.07962	.27581
mtDNA.POST.P	12	.6251	.06851	.23734
mtDNA.PRE.T20	12	.6203	.05365	.18585
mtDNA.POST.T20	12	.6824	.07028	.24345
CRT.PRE.P	12	1.1500	.02611	.09045
CRT.POST.P	12	1.1417	.02876	.09962
CRT.PRE.T20	11	1.1091	.03426	.11362
CRT.POST.T20	11	1.1545	.02473	.08202
AST.PRE.P	12	26.0833	2.77832	9.62439
AST.POST.P	12	23.5000	1.77312	6.14225
AST.PRE.T20	11	23.4545	1.89432	6.28273
AST.POST.T20	11	23.7273	1.54385	5.12037
ALT.PRE.P	12	23.3333	1.52918	5.29723
ALT.POST.P	12	21.1667	1.34183	4.64823
ALT.PRE.T20	11	22.2727	2.43494	8.07578
ALT.POST.T20	11	23.6364	2.53080	8.39372
BIL.PRE.P	12	.9000	.09692	.33575
BIL.POST.P	12	.9667	.15635	.54160
BIL.PRE.T20	11	.7909	.04946	.16404
BIL.POST.T20	11	1.0727	.11839	.39266
PROT.PRE.P	12	74.5000	2.04680	7.09033
PRO.POST.P	12	74.2500	1.75432	6.07716
PRO.PRE.T20	11	74.2727	1.88820	6.26244
PROT.POST.T20	11	74.9091	2.08656	6.92033
N válido (según lista)	11			

```
 GET
  FILE='/Users/SergioBarroso/Desktop/T20-RAL Reviewers/Base de datos/T20.sav'.
DATASET NAME Conjunto_de_datos1 WINDOW=FRONT.
EXECUTE.
*Nonparametric Tests: Related Samples.
NPTESTS
  /RELATED TEST(CHOL.PRE.P CHOL.PRE.T20) WILCOXON
  /MISSING SCOPE=ANALYSIS USERMISSING=EXCLUDE
  /CRITERIA ALPHA=0.05  CILEVEL=95.
```

Pruebas no paramétricas

Notas

Resultados creados		22-MAR-2019 17:25:13
Comentarios		
Entrada	Datos	/Users/SergioBarroso/Desktop/T20-RAL Reviewers/Base de datos/T20.sav
	Conjunto de datos activo	Conjunto_de_datos1
	Filtro	<ninguno>
	Peso	<ninguno>
	Dividir archivo	<ninguno>
	Núm. de filas del archivo de trabajo	12
Sintaxis		NPTESTS /RELATED TEST(CHOL.PRE.P CHOL.PRE.T20) WILCOXON /MISSING SCOPE=ANALYSIS USERMISSING=EXCLUDE /CRITERIA ALPHA=0.05 CILEVEL=95.
Recursos	Tiempo de procesador	00:00:00.65
	Tiempo transcurrido	00:00:00.00

```
[Conjunto_de_datos1] /Users/SergioBarroso/Desktop/T20-RAL Reviewers/Base de datos/T20.sav
```

Resumen de prueba de hipótesis

	Hipótesis nula	Test	Sig.	Decisión
1	La mediana de las diferencias entre CHOL.PRE.P y CHOL.PRE.T20 es igual a 0.	Prueba de Wilcoxon de los rangos con signo de muestras relacionadas	.695	Retener la hipótesis nula.

Se muestran las significancias asintóticas. El nivel de significancia es .05.

Prueba de Wilcoxon de los rangos con signo de muestras relacionadas

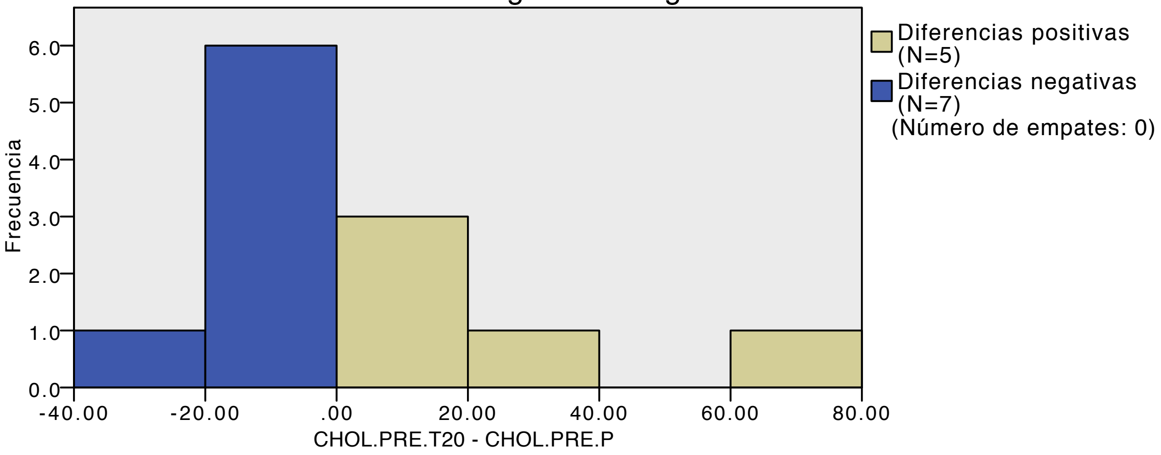

N total	12
Probar estadística	34.000
Error típico	12.743
Estadística de prueba estandarizada	-.392
Sig. asintótica (prueba de dos caras)	.695

Información de campo continuo

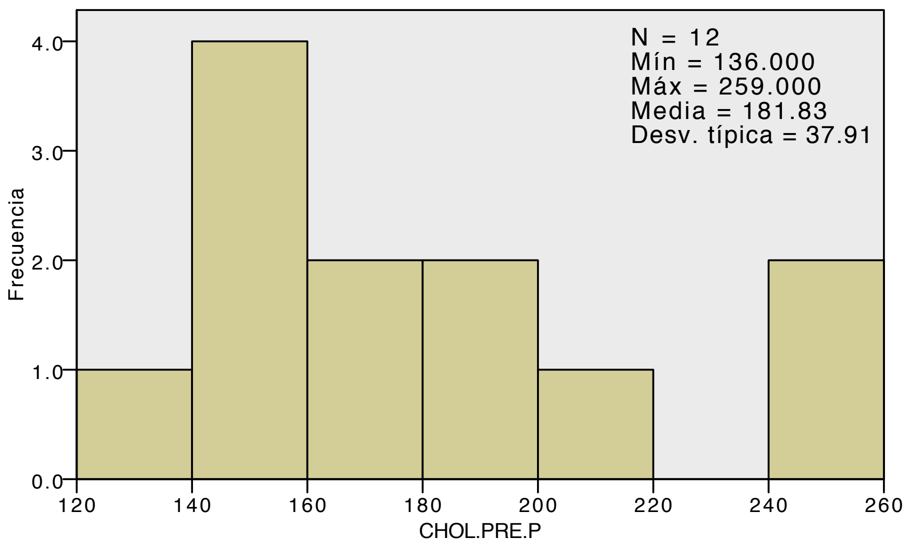

Información de campo continuo

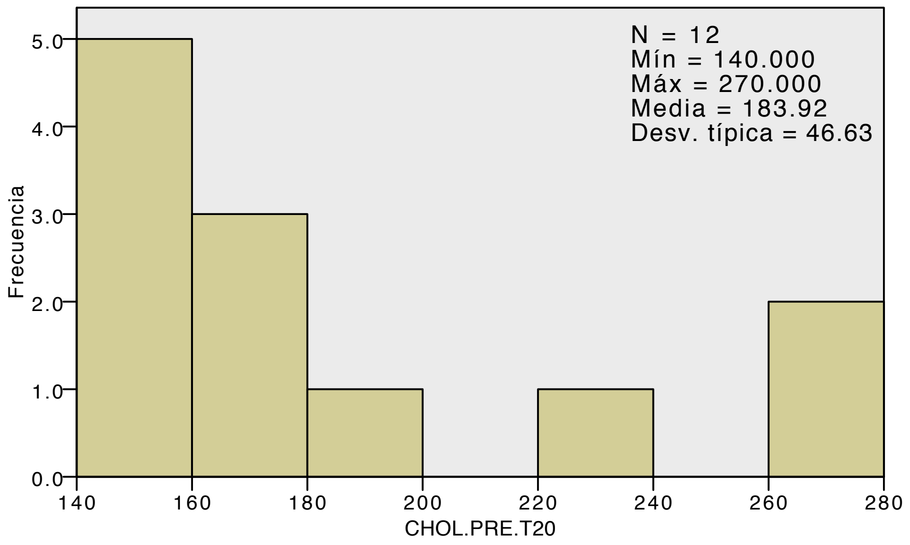


```
 *Nonparametric Tests: Related Samples.
NPTESTS
  /RELATED TEST(HDL.PRE.P HDL.PRE.T20) WILCOXON
  /MISSING SCOPE=ANALYSIS USERMISSING=EXCLUDE
  /CRITERIA ALPHA=0.05  CILEVEL=95.
```

Pruebas no paramétricas

Notas

Resultados creados		22-MAR-2019 17:26:15
Comentarios		
Entrada	Datos	/Users/SergioBarroso/Desktop/T20-RAL Reviewers/Base de datos/T20.sav
	Conjunto de datos activo	Conjunto_de_datos1
	Filtro	<ninguno>
	Peso	<ninguno>
	Dividir archivo	<ninguno>
	Núm. de filas del archivo de trabajo	12
Sintaxis		NPTESTS /RELATED TEST(HDL.PRE.P HDL.PRE.T20) WILCOXON /MISSING SCOPE=ANALYSIS USERMISSING=EXCLUDE /CRITERIA ALPHA=0.05 CILEVEL=95.
Recursos	Tiempo de procesador	00:00:00.11
	Tiempo transcurrido	00:00:00.00

```
[Conjunto_de_datos1] /Users/SergioBarroso/Desktop/T20-RAL Reviewers/Base de datos/T20.sav
```

Resumen de prueba de hipótesis

	Hipótesis nula	Test	Sig.	Decisión
1	La mediana de las diferencias entre HDL.PRE.P y HDL.PRE.T20 es igual a 0.	Prueba de Wilcoxon de los rangos con signo de muestras relacionadas	.075	Retener la hipótesis nula.

Se muestran las significancias asintóticas. El nivel de significancia es .05.

Prueba de Wilcoxon de los rangos con signo de muestras relacionadas

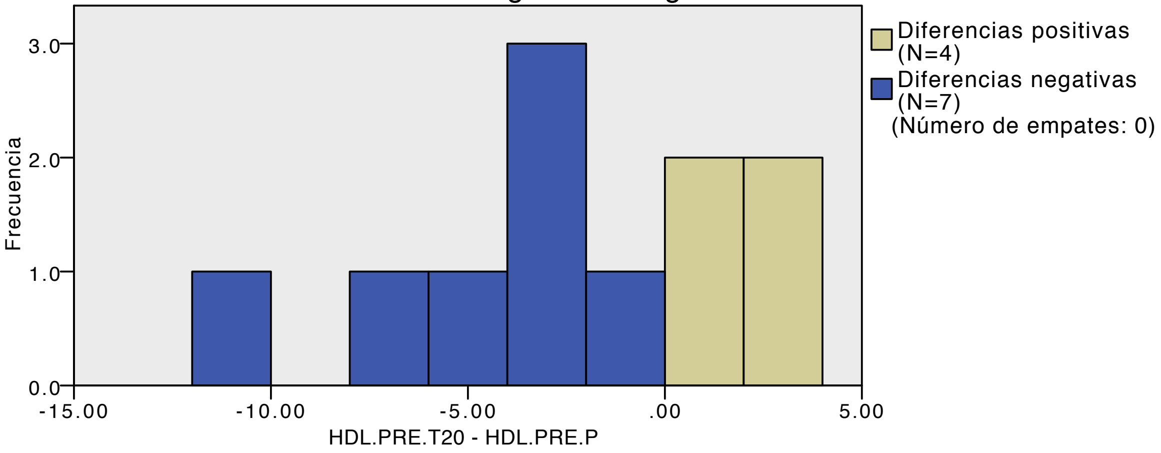

N total	11
Probar estadística	13.000
Error típico	11.214
Estadística de prueba estandarizada	-1.784
Sig. asintótica (prueba de dos caras)	.075

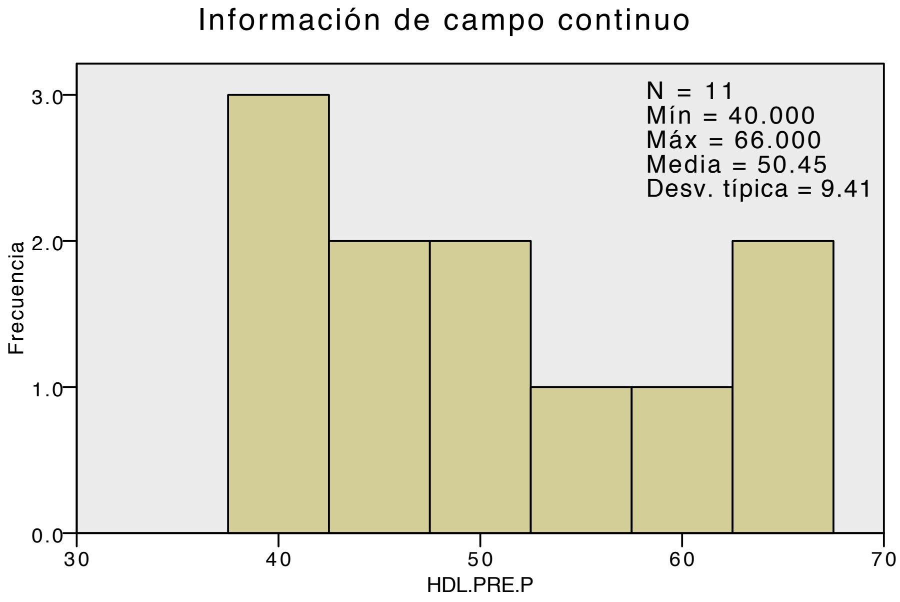

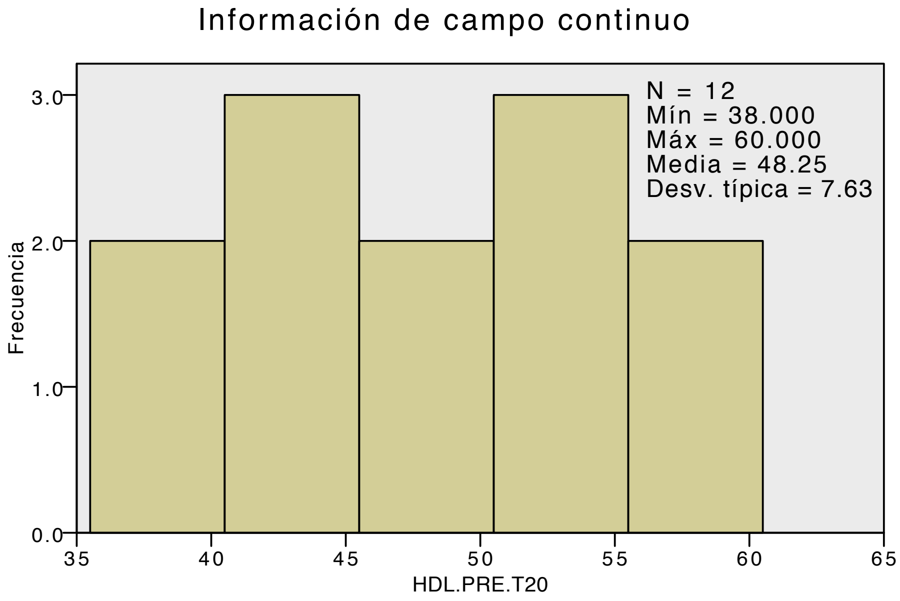


```
 *Nonparametric Tests: Related Samples.
NPTESTS
  /RELATED TEST(LDL.PRE.P LDL.PRE.T20) WILCOXON
  /MISSING SCOPE=ANALYSIS USERMISSING=EXCLUDE
  /CRITERIA ALPHA=0.05  CILEVEL=95.
```

Pruebas no paramétricas

Notas

Resultados creados		22-MAR-2019 17:27:03
Comentarios		
Entrada	Datos	/Users/SergioBarroso/Desktop/T20-RAL Reviewers/Base de datos/T20.sav
	Conjunto de datos activo	Conjunto_de_datos1
	Filtro	<ninguno>
	Peso	<ninguno>
	Dividir archivo	<ninguno>
	Núm. de filas del archivo de trabajo	12
Sintaxis		NPTESTS /RELATED TEST(LDL.PRE.P LDL.PRE.T20) WILCOXON /MISSING SCOPE=ANALYSIS USERMISSING=EXCLUDE /CRITERIA ALPHA=0.05 CILEVEL=95.
Recursos	Tiempo de procesador	00:00:00.13
	Tiempo transcurrido	00:00:00.00

[Conjunto_de_datos1] /Users/SergioBarroso/Desktop/T20-RAL Reviewers/Base de datos/T20.sav

Resumen de prueba de hipótesis

	Hipótesis nula	Test	Sig.	Decisión
1	La mediana de las diferencias entre LDL.PRE.P y LDL.PRE.T20 es igual a 0.	Prueba de Wilcoxon de los rangos con signo de muestras relacionadas	.211	Retener la hipótesis nula.

Se muestran las significancias asintóticas. El nivel de significancia es .05.

Prueba de Wilcoxon de los rangos con signo de muestras relacionadas

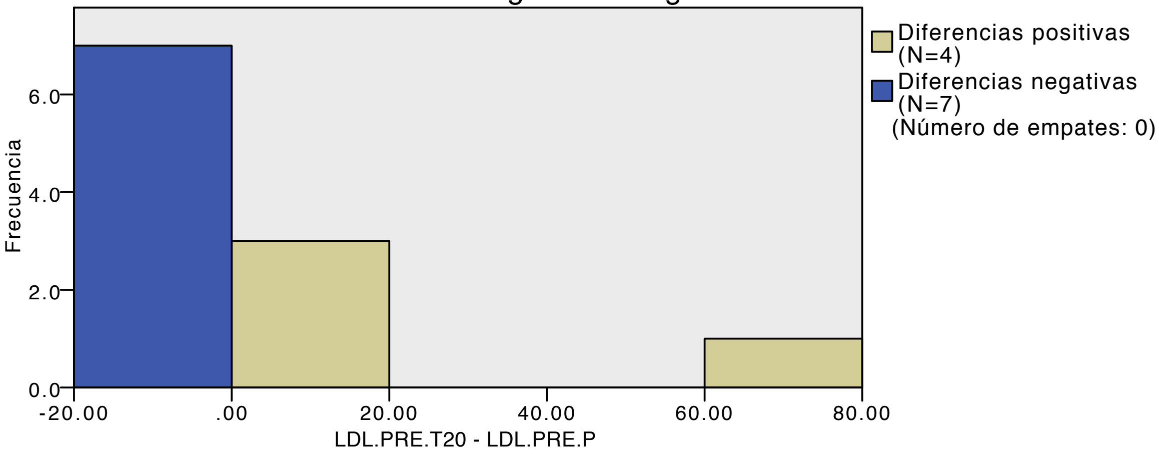

N total	11
Probar estadística	19.000
Error típico	11.186
Estadística de prueba estandarizada	-1.252
Sig. asintótica (prueba de dos caras)	.211

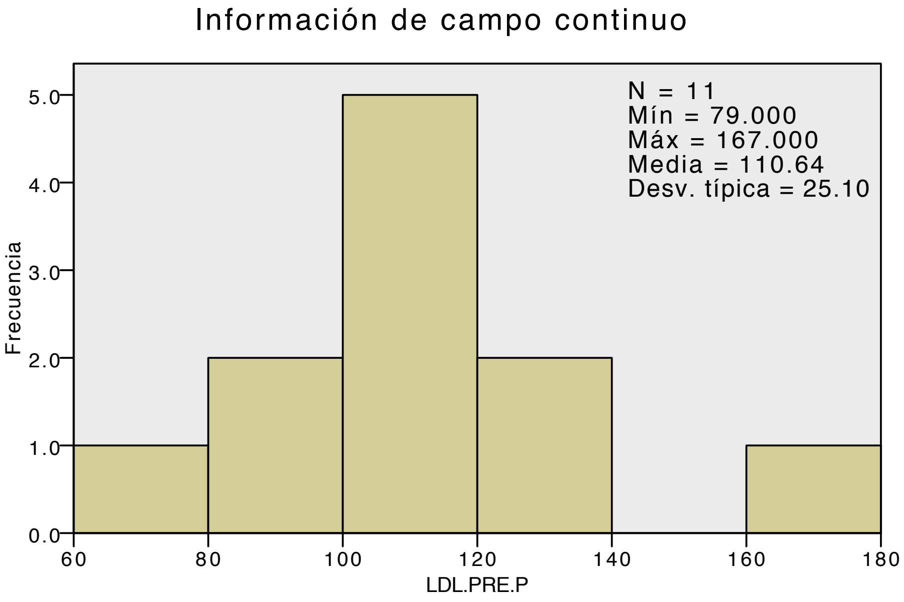

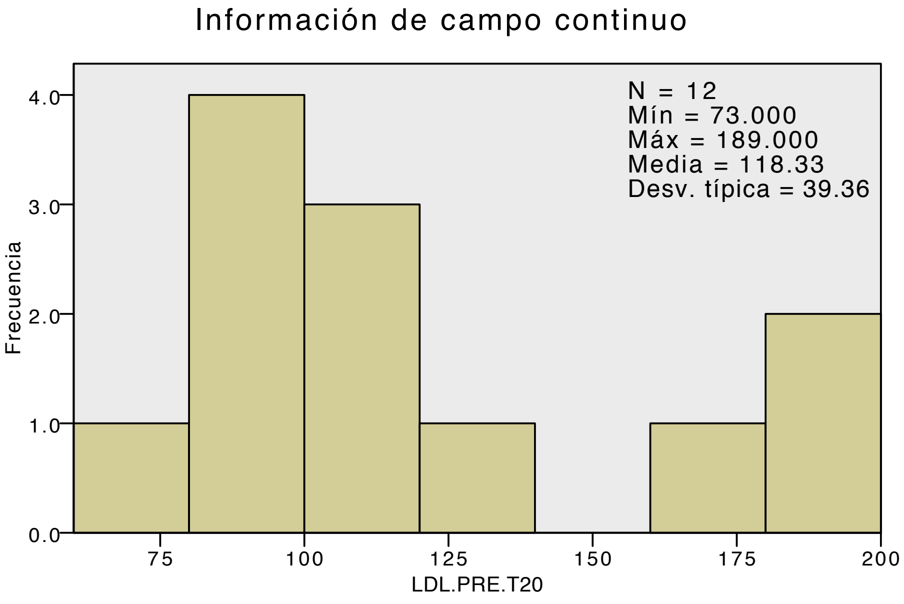


```
 *Nonparametric Tests: Related Samples.
NPTESTS
  /RELATED TEST(TAG.PRE.P TAG.PRE.T20) WILCOXON
  /MISSING SCOPE=ANALYSIS USERMISSING=EXCLUDE
  /CRITERIA ALPHA=0.05  CILEVEL=95.
```

Pruebas no paramétricas

Notas

Resultados creados		22-MAR-2019 17:27:24
Comentarios		
Entrada	Datos	/Users/SergioBarroso/Desktop/T20-RAL Reviewers/Base de datos/T20.sav
	Conjunto de datos activo	Conjunto_de_datos1
	Filtro	<ninguno>
	Peso	<ninguno>
	Dividir archivo	<ninguno>
	Núm. de filas del archivo de trabajo	12
Sintaxis		NPTESTS<br>/RELATED TEST(TAG.PRE.P TAG.PRE.T20) WILCOXON<br>/MISSING SCOPE=ANALYSIS USERMISSING=EXCLUDE<br>/CRITERIA ALPHA=0.05 CILEVEL=95.
Recursos	Tiempo de procesador	00:00:00.10
	Tiempo transcurrido	00:00:00.00

```
[Conjunto_de_datos1] /Users/SergioBarroso/Desktop/T20-RAL Reviewers/Base de datos/T20.sav
```

Resumen de prueba de hipótesis

	Hipótesis nula	Test	Sig.	Decisión
1	La mediana de las diferencias entre TAG.PRE.P y TAG.PRE.T20 es igual a 0.	Prueba de Wilcoxon de los rangos con signo de muestras relacionadas	.784	Retener la hipótesis nula.

Se muestran las significancias asintóticas. El nivel de significancia es .05.

Prueba de Wilcoxon de los rangos con signo de muestras relacionadas

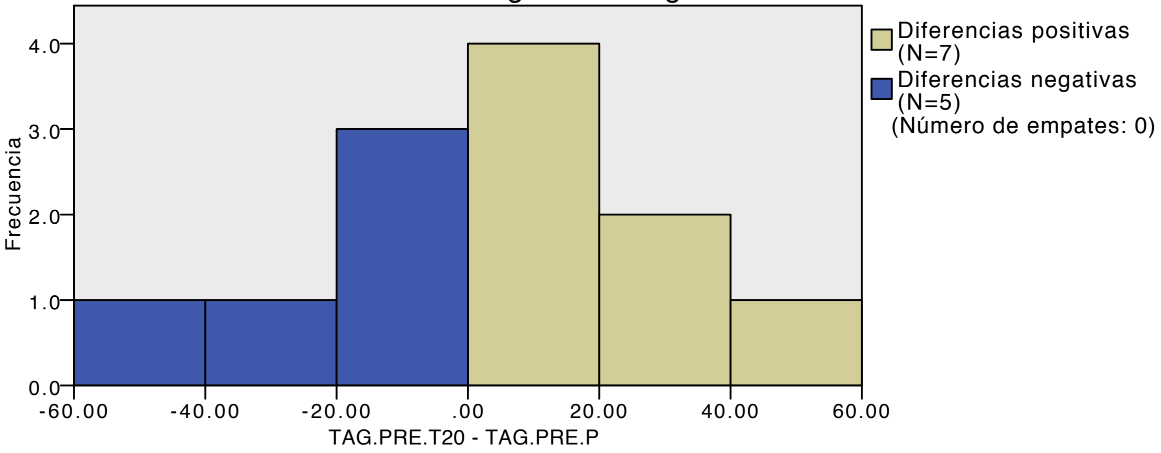

N total	12
Probar estadística	42.500
Error típico	12.743
Estadística de prueba estandarizada	.275
Sig. asintótica (prueba de dos caras)	.784

Información de campo continuo

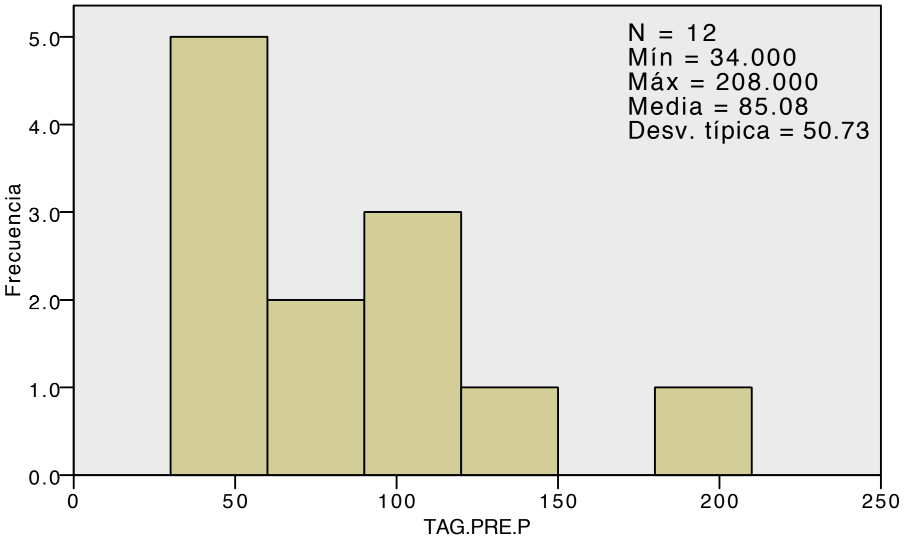

Información de campo continuo

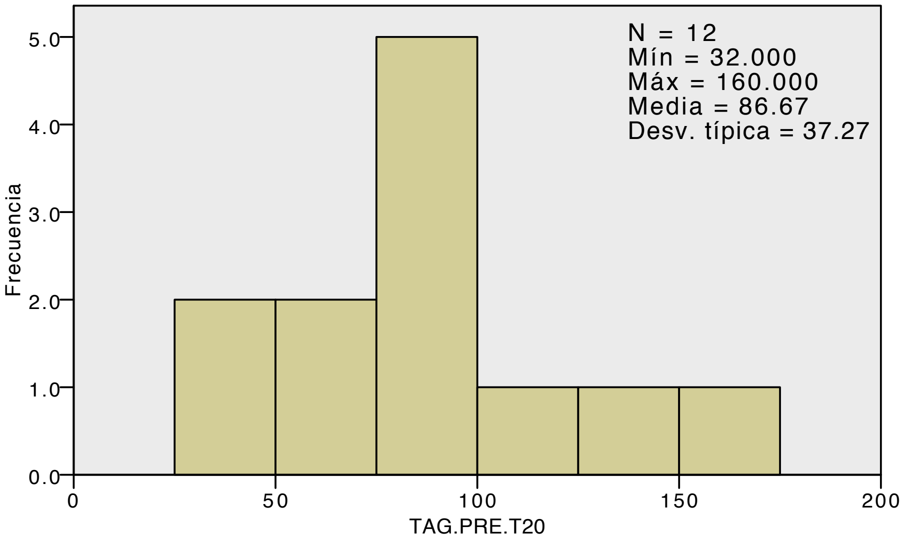


```
 *Nonparametric Tests: Related Samples.
NPTESTS
  /RELATED TEST(GLU.PRE.P GLU.PRE.T20) WILCOXON
  /MISSING SCOPE=ANALYSIS USERMISSING=EXCLUDE
  /CRITERIA ALPHA=0.05  CILEVEL=95.
```

Pruebas no paramétricas

Notas

Resultados creados		22-MAR-2019 17:27:50
Comentarios		
Entrada	Datos	/Users/SergioBarroso/Desktop/T20-RAL Reviewers/Base de datos/T20.sav
	Conjunto de datos activo	Conjunto_de_datos1
	Filtro	<ninguno>
	Peso	<ninguno>
	Dividir archivo	<ninguno>
	Núm. de filas del archivo de trabajo	12
Sintaxis		NPTESTS /RELATED TEST(GLU.PRE.P GLU.PRE.T20) WILCOXON /MISSING SCOPE=ANALYSIS USERMISSING=EXCLUDE /CRITERIA ALPHA=0.05 CILEVEL=95.
Recursos	Tiempo de procesador	00:00:00.15
	Tiempo transcurrido	00:00:00.00

```
[Conjunto_de_datos1] /Users/SergioBarroso/Desktop/T20-RAL Reviewers/Base de datos/T20.sav
```

Resumen de prueba de hipótesis

	Hipótesis nula	Test	Sig.	Decisión
1	La mediana de las diferencias entre GLU.PRE.P y GLU.PRE.T20 es igual a 0.	Prueba de Wilcoxon de los rangos con signo de muestras relacionadas	.594	Retener la hipótesis nula.

Se muestran las significancias asintóticas. El nivel de significancia es .05.

Prueba de Wilcoxon de los rangos con signo de muestras relacionadas

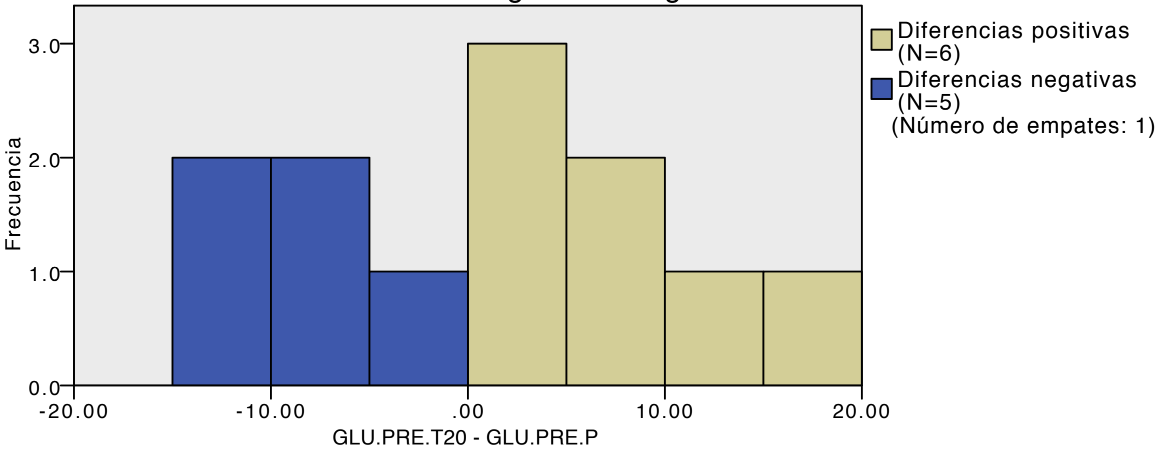

N total	12
Probar estadística	39.000
Error típico	11.242
Estadística de prueba estandarizada	.534
Sig. asintótica (prueba de dos caras)	.594

Información de campo continuo

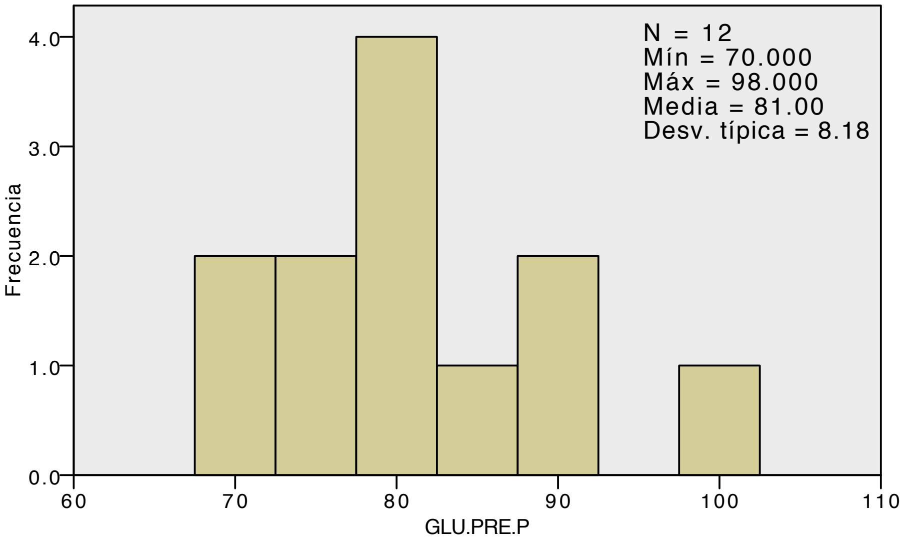

Información de campo continuo

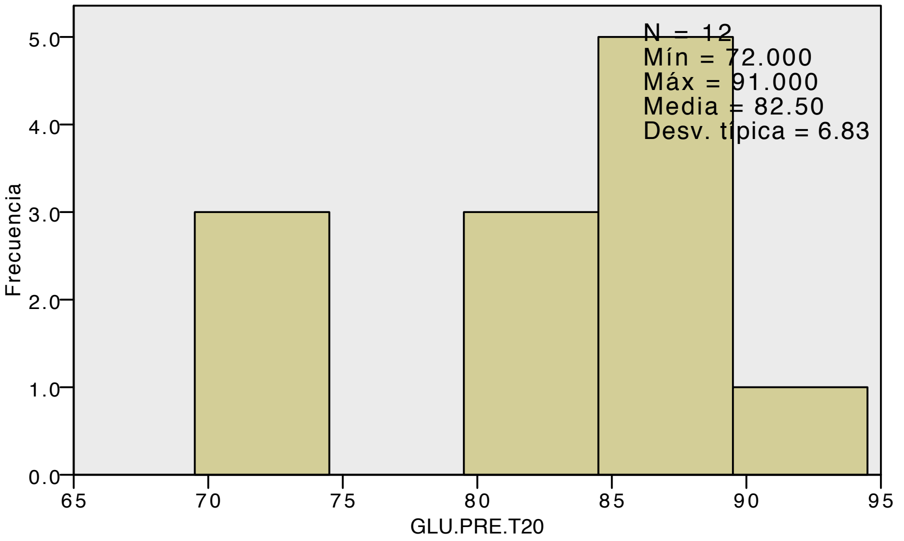


```
 *Nonparametric Tests: Related Samples.
NPTESTS
  /RELATED TEST(mtDNA.PRE.P mtDNA.PRE.T20) WILCOXON
  /MISSING SCOPE=ANALYSIS USERMISSING=EXCLUDE
  /CRITERIA ALPHA=0.05  CILEVEL=95.
```

Pruebas no paramétricas

Notas

Resultados creados		22-MAR-2019 17:28:08
Comentarios		
Entrada	Datos	/Users/SergioBarroso/Desktop/T20-RAL Reviewers/Base de datos/T20.sav
	Conjunto de datos activo	Conjunto_de_datos1
	Filtro	<ninguno>
	Peso	<ninguno>
	Dividir archivo	<ninguno>
	Núm. de filas del archivo de trabajo	12
Sintaxis		NPTESTS /RELATED TEST (mtDNA.PRE.P mtDNA.PRE.T20) WILCOXON /MISSING SCOPE=ANALYSIS USERMISSING=EXCLUDE /CRITERIA ALPHA=0.05 CILEVEL=95.
Recursos	Tiempo de procesador	00:00:00.07
	Tiempo transcurrido	00:00:00.00

```
[Conjunto_de_datos1] /Users/SergioBarroso/Desktop/T20-RAL Reviewers/Base de datos/T20.sav
```

Resumen de prueba de hipótesis

	Hipótesis nula	Test	Sig.	Decisión
1	La mediana de las diferencias entre mtDNA.PRE.P y mtDNA.PRE.T20 es igual a 0.	Prueba de Wilcoxon de los rangos con signo de muestras relacionadas	.239	Retener la hipótesis nula.

Se muestran las significancias asintóticas. El nivel de significancia es .05.

Prueba de Wilcoxon de los rangos con signo de muestras relacionadas

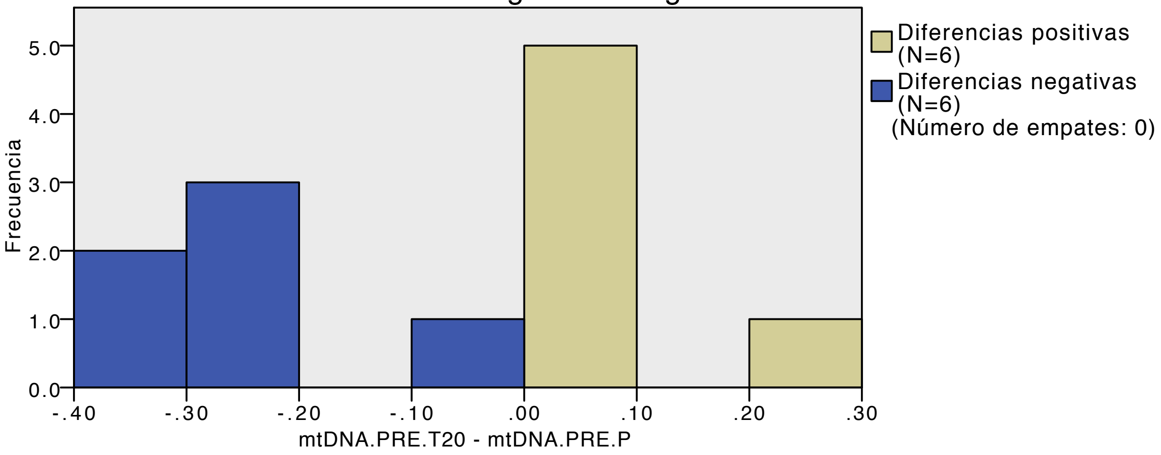

N total	12
Probar estadística	24.000
Error típico	12.748
Estadística de prueba estandarizada	-1.177
Sig. asintótica (prueba de dos caras)	.239

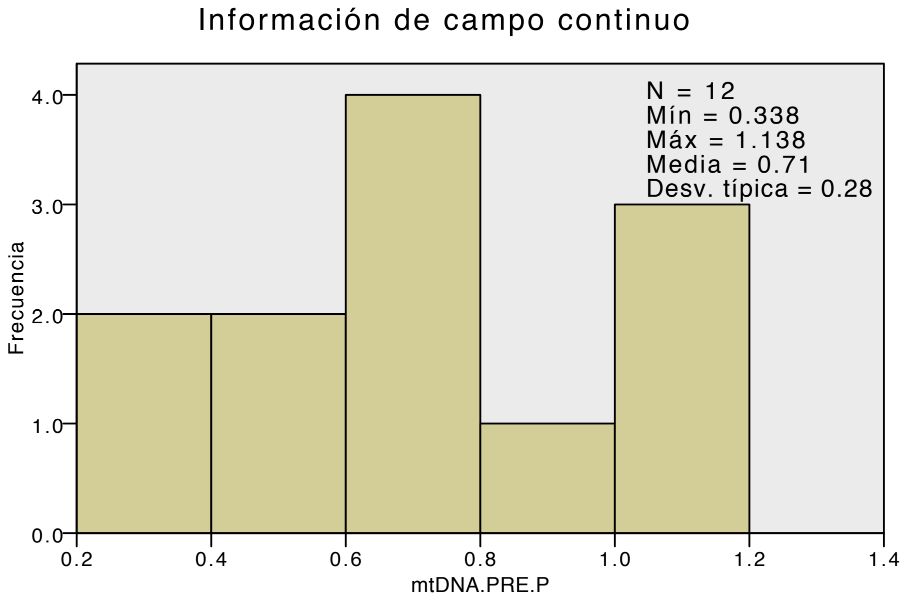

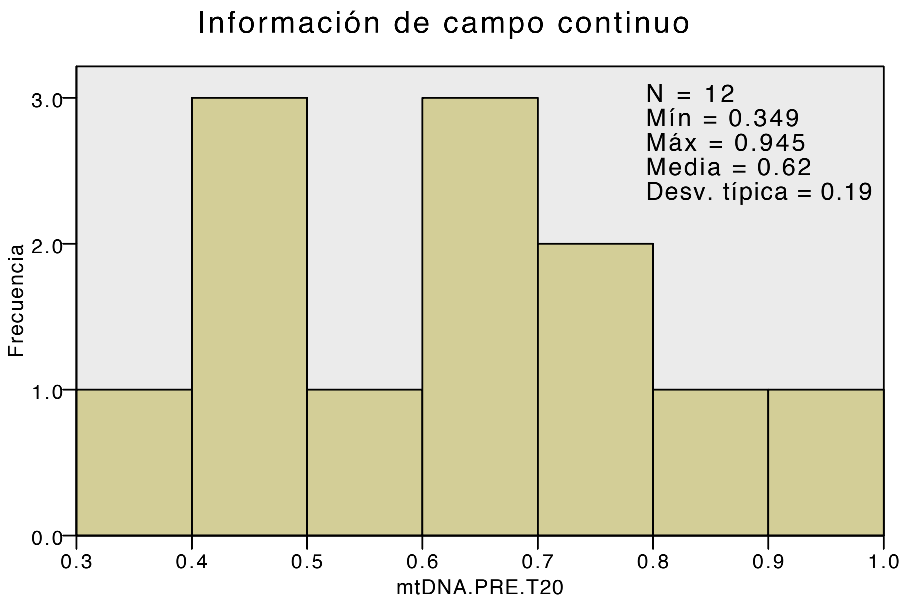


```
 *Nonparametric Tests: Related Samples.
NPTESTS
  /RELATED TEST(CRT.PRE.P CRT.PRE.T20) WILCOXON
  /MISSING SCOPE=ANALYSIS USERMISSING=EXCLUDE
  /CRITERIA ALPHA=0.05  CILEVEL=95.
```

Pruebas no paramétricas

Notas

**Resultados creados**		**22-MAR-2019 17:28:32**
**Comentarios**		
**Entrada**	**Datos**	**/Users/SergioBarroso/Desktop/T20-RAL Reviewers/Base de datos/T20.sav**
	**Conjunto de datos activo**	**Conjunto_de_datos1**
	**Filtro**	**<ninguno>**
	**Peso**	**<ninguno>**
	**Dividir archivo**	**<ninguno>**
	**Núm. de filas del archivo de trabajo**	**12**
**Sintaxis**		**NPTESTS /RELATED TEST(CRT.PRE.P CRT.PRE.T20) WILCOXON /MISSING SCOPE=ANALYSIS USERMISSING=EXCLUDE /CRITERIA ALPHA=0.05 CILEVEL=95.**
**Recursos**	**Tiempo de procesador**	**00:00:00.11**
	**Tiempo transcurrido**	**00:00:00.00**

```
[Conjunto_de_datos1] /Users/SergioBarroso/Desktop/T20-RAL Reviewers/Base de datos/T20.sav
```

Resumen de prueba de hipótesis

	Hipótesis nula	Test	Sig.	Decisión
1	La mediana de las diferencias entre CRT.PRE.P y CRT.PRE.T20 es igual a 0.	Prueba de Wilcoxon de los rangos con signo de muestras relacionadas	.334	Retener la hipótesis nula.

Se muestran las significancias asintóticas. El nivel de significancia es .05.

Prueba de Wilcoxon de los rangos con signo de muestras relacionadas

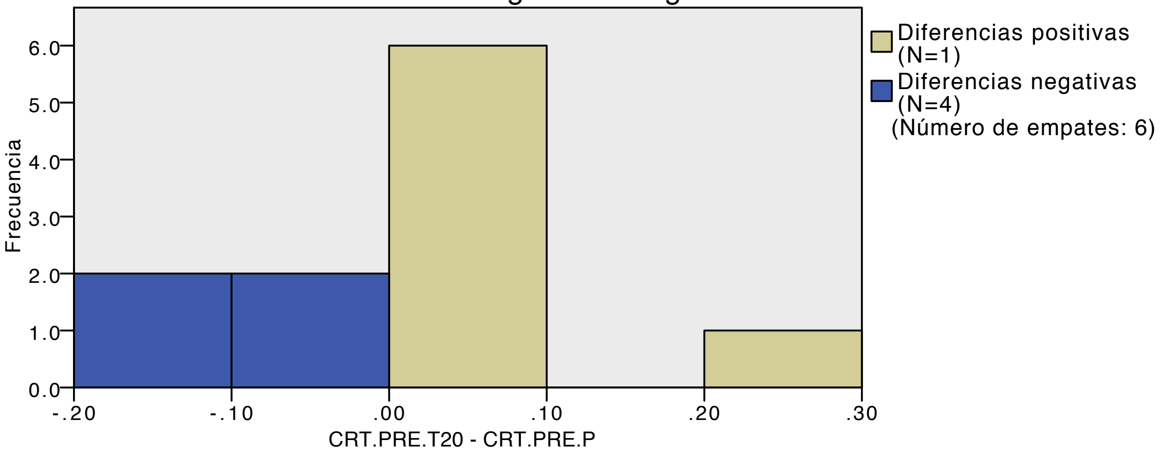

N total	11
Probar estadística	4.000
Error típico	3.623
Estadística de prueba estandarizada	-.966
Sig. asintótica (prueba de dos caras)	.334

Información de campo continuo

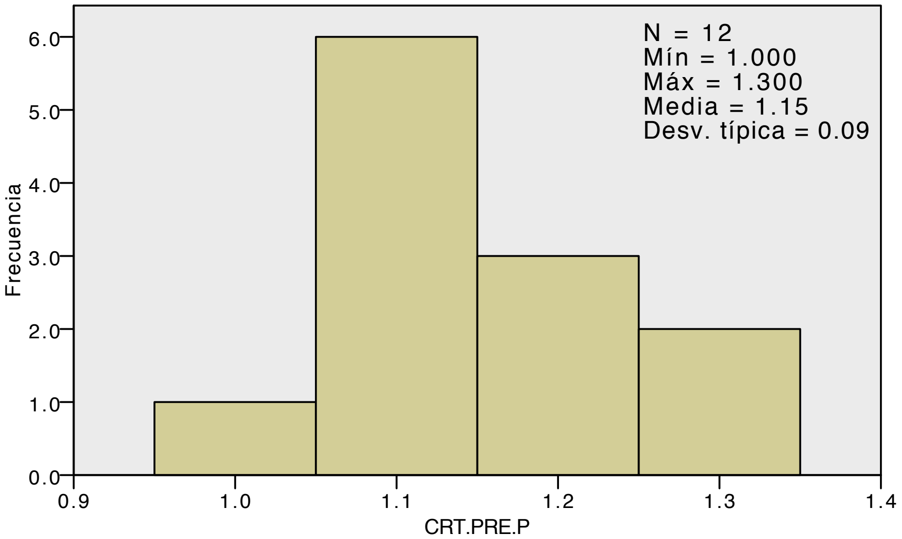

Información de campo continuo

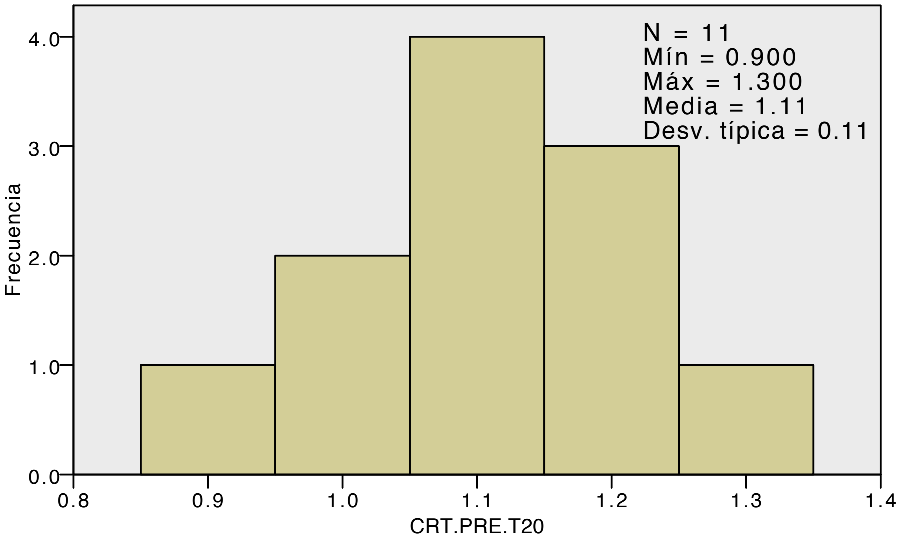


```
 *Nonparametric Tests: Related Samples.
NPTESTS
  /RELATED TEST(AST.PRE.P AST.PRE.T20) WILCOXON
  /MISSING SCOPE=ANALYSIS USERMISSING=EXCLUDE
  /CRITERIA ALPHA=0.05  CILEVEL=95.
```

Pruebas no paramétricas

Notas

Resultados creados		22-MAR-2019 17:30:31
Comentarios		
Entrada	Datos	/Users/SergioBarroso/Desktop/T20-RAL Reviewers/Base de datos/T20.sav
	Conjunto de datos activo	Conjunto_de_datos1
	Filtro	<ninguno>
	Peso	<ninguno>
	Dividir archivo	<ninguno>
	Núm. de filas del archivo de trabajo	12
Sintaxis		NPTESTS /RELATED TEST(AST.PRE.P AST.PRE.T20) WILCOXON /MISSING SCOPE=ANALYSIS USERMISSING=EXCLUDE /CRITERIA ALPHA=0.05 CILEVEL=95.
Recursos	Tiempo de procesador	00:00:00.15
	Tiempo transcurrido	00:00:00.00

```
[Conjunto_de_datos1] /Users/SergioBarroso/Desktop/T20-RAL Reviewers/Base de datos/T20.sav
```

Resumen de prueba de hipótesis

	Hipótesis nula	Test	Sig.	Decisión
1	La mediana de las diferencias entre AST.PRE.P y AST.PRE.T20 es igual a 0.	Prueba de Wilcoxon de los rangos con signo de muestras relacionadas	.893	Retener la hipótesis nula.

Se muestran las significancias asintóticas. El nivel de significancia es .05.

Prueba de Wilcoxon de los rangos con signo de muestras relacionadas

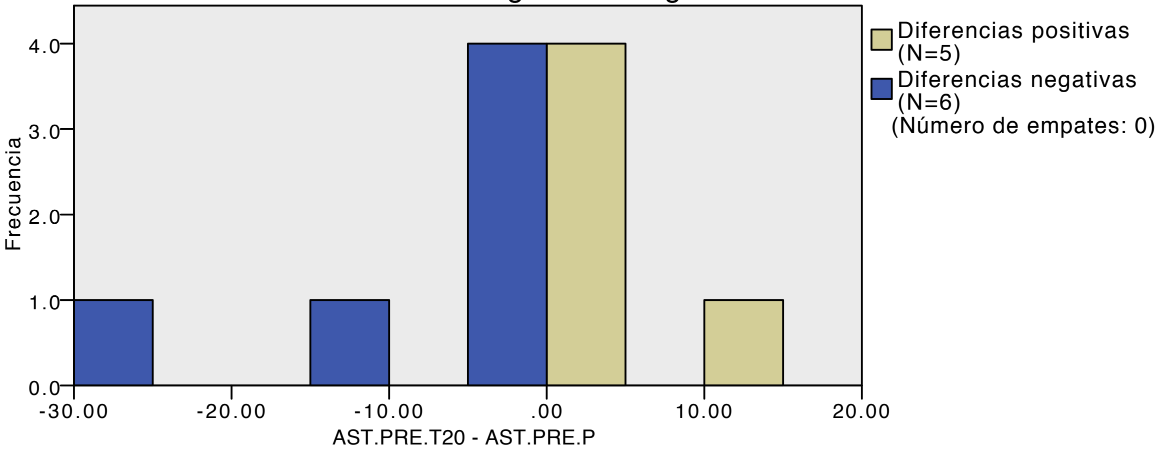

N total	11
Probar estadística	31.500
Error típico	11.197
Estadística de prueba estandarizada	-.134
Sig. asintótica (prueba de dos caras)	.893

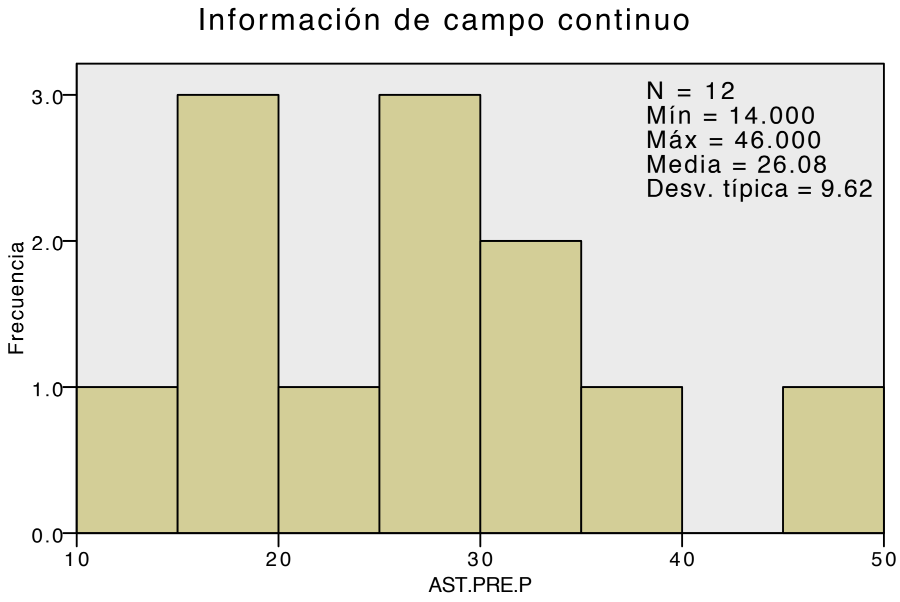

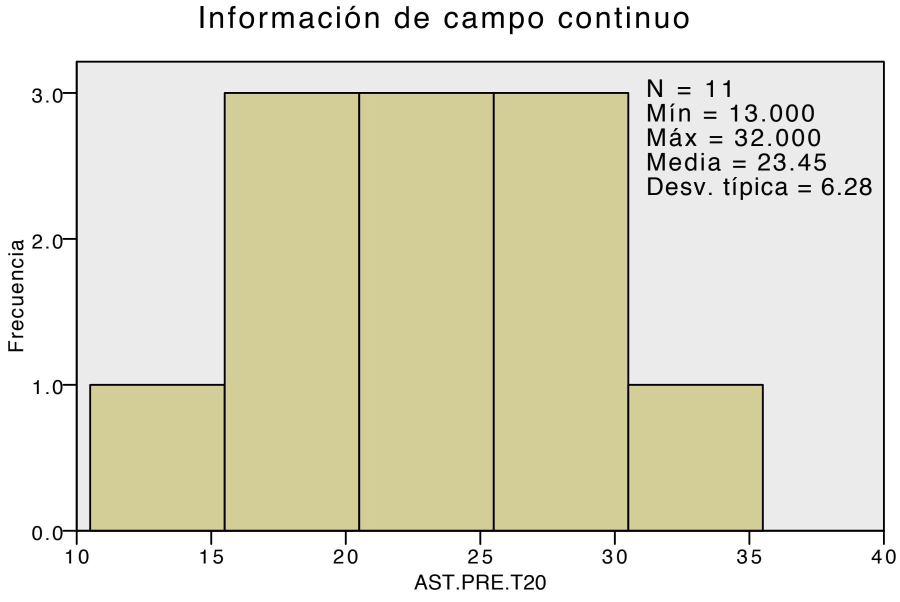


```
 *Nonparametric Tests: Related Samples.
NPTESTS
  /RELATED TEST(ALT.PRE.P ALT.PRE.T20) WILCOXON
  /MISSING SCOPE=ANALYSIS USERMISSING=EXCLUDE
  /CRITERIA ALPHA=0.05  CILEVEL=95.
```

Pruebas no paramétricas

Notas

**Resultados creados**		**22-MAR-2019 17:31:06**
**Comentarios**		
**Entrada**	**Datos**	**/Users/SergioBarroso/Desktop/T20-RAL Reviewers/Base de datos/T20.sav**
	**Conjunto de datos activo**	**Conjunto_de_datos1**
	**Filtro**	**<ninguno>**
	**Peso**	**<ninguno>**
	**Dividir archivo**	**<ninguno>**
	**Núm. de filas del archivo de trabajo**	**12**
**Sintaxis**		**NPTESTS /RELATED TEST(ALT.PRE.P ALT.PRE.T20) WILCOXON /MISSING SCOPE=ANALYSIS USERMISSING=EXCLUDE /CRITERIA ALPHA=0.05 CILEVEL=95.**
**Recursos**	**Tiempo de procesador**	**00:00:00.06**
	**Tiempo transcurrido**	**00:00:00.00**

```
[Conjunto_de_datos1] /Users/SergioBarroso/Desktop/T20-RAL Reviewers/Base de datos/T20.sav
```

Resumen de prueba de hipótesis

	Hipótesis nula	Test	Sig.	Decisión
1	La mediana de las diferencias entre ALT.PRE.P y ALT.PRE.T20 es igual a 0.	Prueba de Wilcoxon de los rangos con signo de muestras relacionadas	.422	Retener la hipótesis nula.

Se muestran las significancias asintóticas. El nivel de significancia es .05.

Prueba de Wilcoxon de los rangos con signo de muestras relacionadas

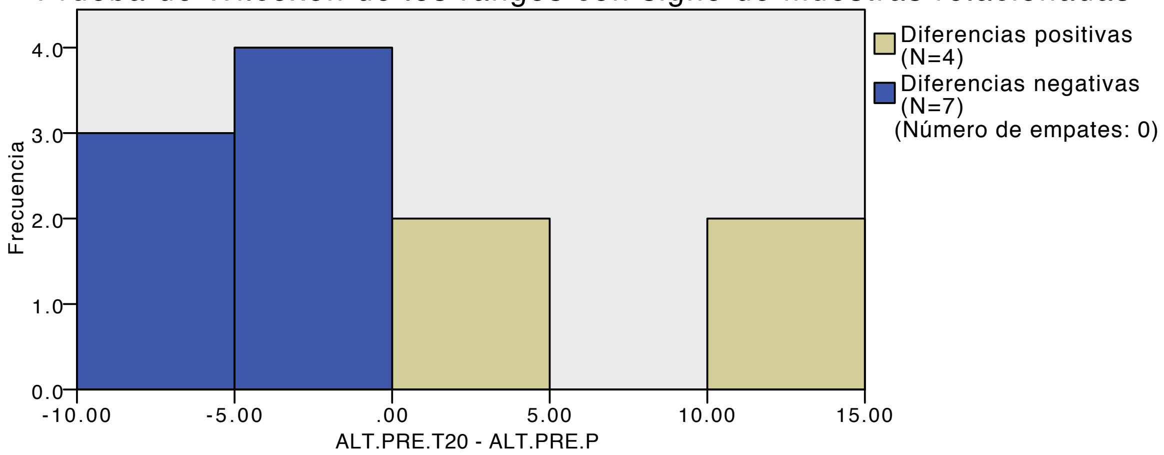

N total	11
Probar estadística	24.000
Error típico	11.219
Estadística de prueba estandarizada	-.802
Sig. asintótica (prueba de dos caras)	.422

Información de campo continuo

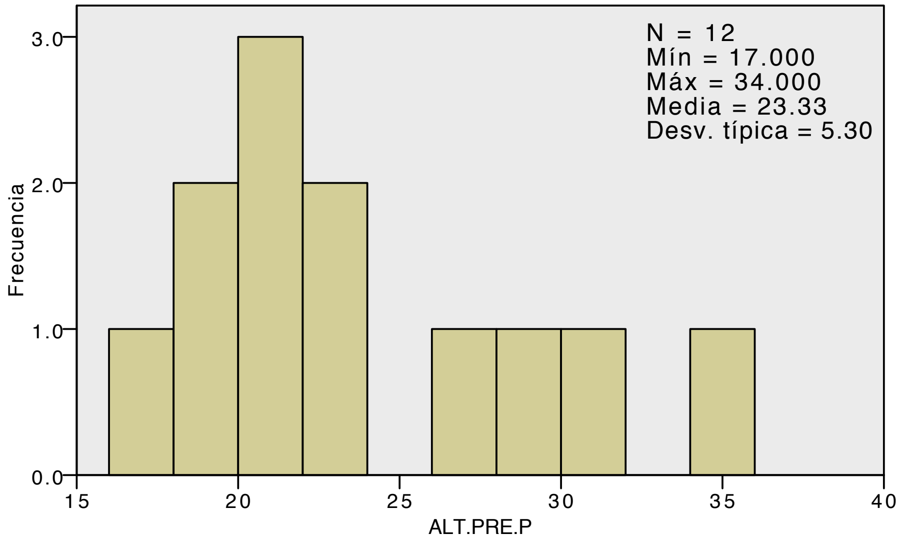

Información de campo continuo

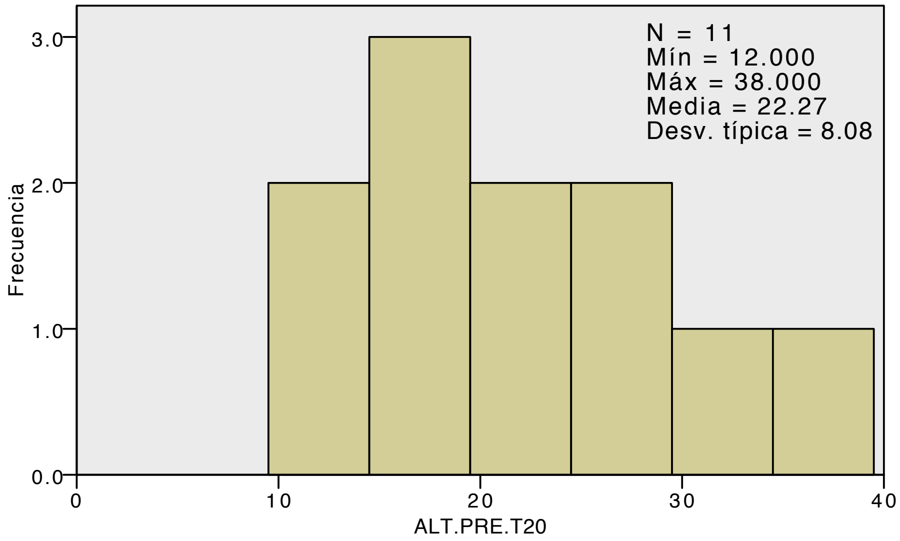


```
 *Nonparametric Tests: Related Samples.
NPTESTS
  /RELATED TEST(BIL.PRE.P BIL.PRE.T20) WILCOXON
  /MISSING SCOPE=ANALYSIS USERMISSING=EXCLUDE
  /CRITERIA ALPHA=0.05  CILEVEL=95.
```

Pruebas no paramétricas

Notas

**Resultados creados**		**22-MAR-2019 17:31:43**
**Comentarios**		
**Entrada**	**Datos**	**/Users/SergioBarroso/Desktop/T20-RAL Reviewers/Base de datos/T20.sav**
	**Conjunto de datos activo**	**Conjunto_de_datos1**
	**Filtro**	**<ninguno>**
	**Peso**	**<ninguno>**
	**Dividir archivo**	**<ninguno>**
	**Núm. de filas del archivo de trabajo**	**12**
**Sintaxis**		**NPTESTS /RELATED TEST(BIL.PRE.P BIL.PRE.T20) WILCOXON /MISSING SCOPE=ANALYSIS USERMISSING=EXCLUDE /CRITERIA ALPHA=0.05 CILEVEL=95.**
**Recursos**	**Tiempo de procesador**	**00:00:00.22**
	**Tiempo transcurrido**	**00:00:00.00**

```
[Conjunto_de_datos1] /Users/SergioBarroso/Desktop/T20-RAL Reviewers/Base de datos/T20.sav
```

Resumen de prueba de hipótesis

	Hipótesis nula	Test	Sig.	Decisión
1	La mediana de las diferencias entre BIL.PRE.P y BIL.PRE.T20 es igual a 0.	Prueba de Wilcoxon de los rangos con signo de muestras relacionadas	.177	Retener la hipótesis nula.

Se muestran las significancias asintóticas. El nivel de significancia es .05.

Prueba de Wilcoxon de los rangos con signo de muestras relacionadas

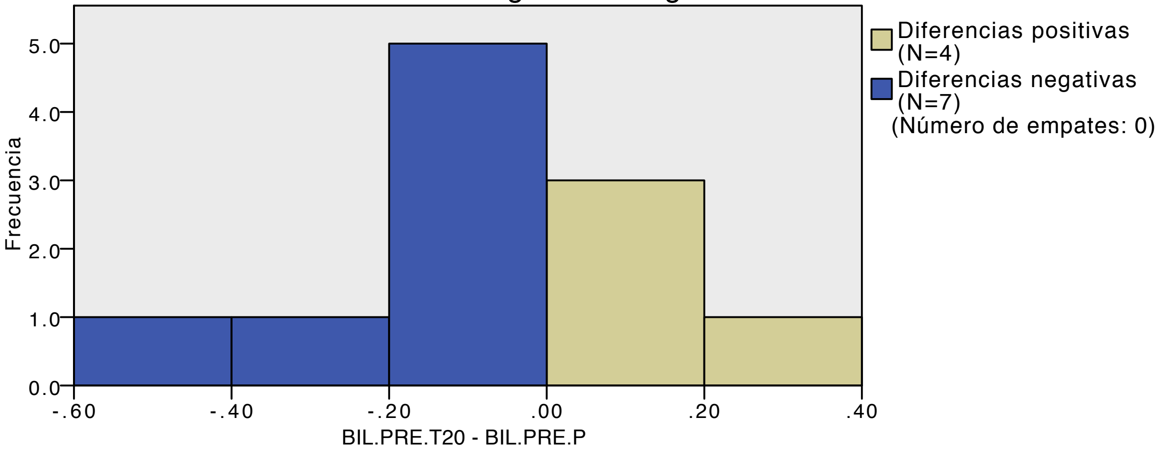

N total	11
Probar estadística	18.000
Error típico	11.113
Estadística de prueba estandarizada	-1.350
Sig. asintótica (prueba de dos caras)	.177

Información de campo continuo

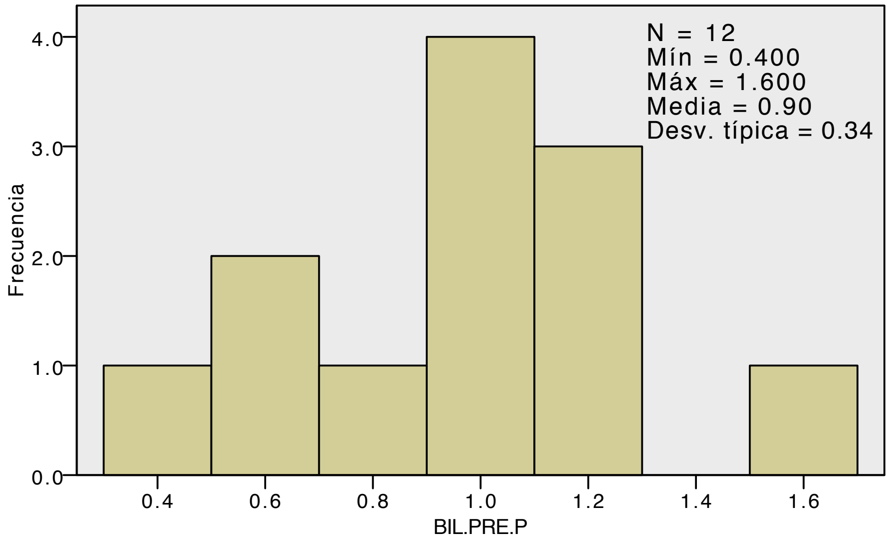

Información de campo continuo

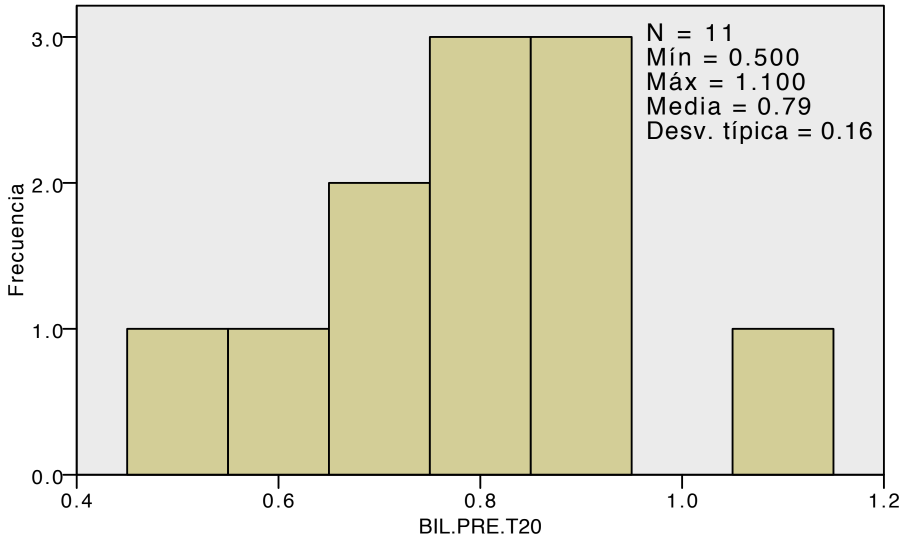


```
 *Nonparametric Tests: Related Samples.
NPTESTS
  /RELATED TEST(PROT.PRE.P PRO.PRE.T20) WILCOXON
  /MISSING SCOPE=ANALYSIS USERMISSING=EXCLUDE
  /CRITERIA ALPHA=0.05  CILEVEL=95.
```

Pruebas no paramétricas

Notas

Resultados creados		22-MAR-2019 17:32:30
Comentarios		
Entrada	Datos	/Users/SergioBarroso/Desktop/T20-RAL Reviewers/Base de datos/T20.sav
	Conjunto de datos activo	Conjunto_de_datos1
	Filtro	<ninguno>
	Peso	<ninguno>
	Dividir archivo	<ninguno>
	Núm. de filas del archivo de trabajo	12
Sintaxis		NPTESTS /RELATED TEST(PROT.PRE.P PRO.PRE.T20) WILCOXON /MISSING SCOPE=ANALYSIS USERMISSING=EXCLUDE /CRITERIA ALPHA=0.05 CILEVEL=95.
Recursos	Tiempo de procesador	00:00:00.11
	Tiempo transcurrido	00:00:00.00

```
[Conjunto_de_datos1] /Users/SergioBarroso/Desktop/T20-RAL Reviewers/Base de datos/T20.sav
```

Resumen de prueba de hipótesis

	Hipótesis nula	Test	Sig.	Decisión
1	La mediana de las diferencias entre PROT.PRE.P y PRO.PRE.T20 es igual a 0.	Prueba de Wilcoxon de los rangos con signo de muestras relacionadas	.503	Retener la hipótesis nula.

Se muestran las significancias asintóticas. El nivel de significancia es .05.

Prueba de Wilcoxon de los rangos con signo de muestras relacionadas

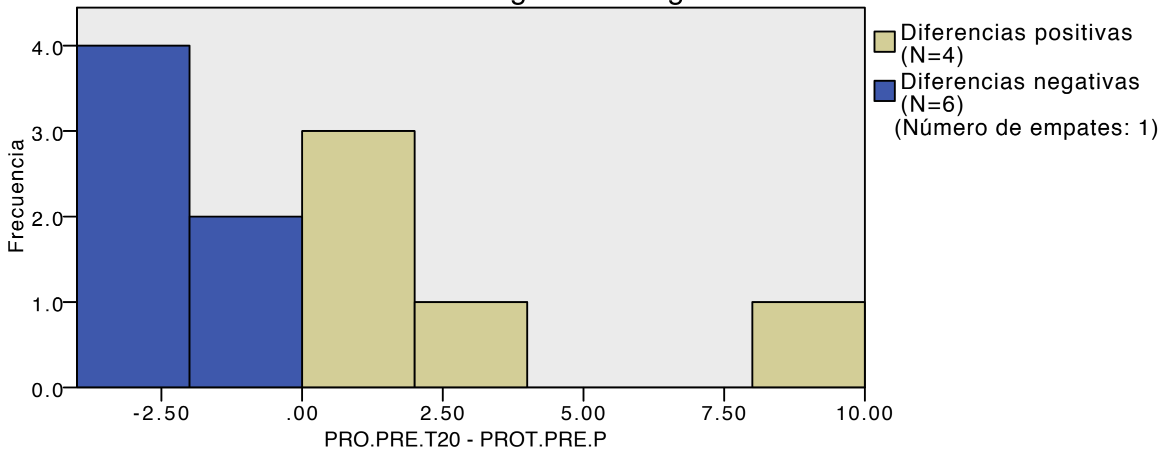

N total	11
Probar estadística	21.000
Error típico	9.715
Estadística de prueba estandarizada	-.669
Sig. asintótica (prueba de dos caras)	.503

Información de campo continuo

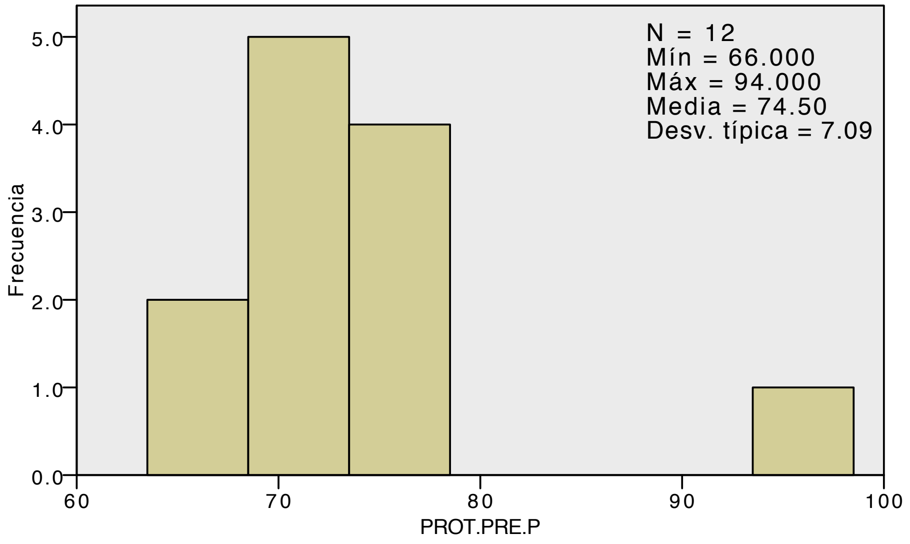

Información de campo continuo

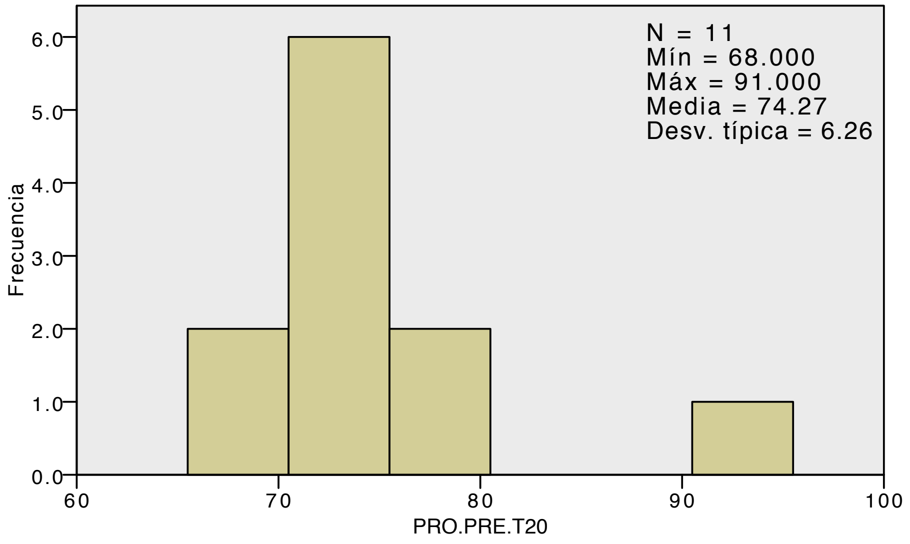

```
 DATASET ACTIVATE Conjunto_de_datos1.

SAVE OUTFILE='/Users/SergioBarroso/Desktop/T20-RAL Reviewers/Base de datos/T20.sav'
  /COMPRESSED.
DATASET ACTIVATE Conjunto_de_datos1.

SAVE OUTFILE='/Users/SergioBarroso/Desktop/T20-RAL Reviewers/Base de datos/T20.sav'
  /COMPRESSED.
*Nonparametric Tests: Related Samples.
NPTESTS
  /RELATED TEST(CHOL.DIF.P CHOL.DIF.T20) WILCOXON
  /MISSING SCOPE=ANALYSIS USERMISSING=EXCLUDE
  /CRITERIA ALPHA=0.05  CILEVEL=95.
```

Pruebas no paramétricas

Notas

Resultados creados		23-MAR-2019 17:40:20
Comentarios		
Entrada	Datos	/Users/SergioBarroso/Desktop/T20-RAL Reviewers/Base de datos/T20.sav
	Conjunto de datos activo	Conjunto_de_datos1
	Filtro	<ninguno>
	Peso	<ninguno>
	Dividir archivo	<ninguno>
	Núm. de filas del archivo de trabajo	12
Sintaxis		NPTESTS /RELATED TEST(CHOL.DIF.P CHOL.DIF.T20) WILCOXON /MISSING SCOPE=ANALYSIS USERMISSING=EXCLUDE /CRITERIA ALPHA=0.05 CILEVEL=95.
Recursos	Tiempo de procesador	00:00:00.13
	Tiempo transcurrido	00:00:00.00

```
[Conjunto_de_datos1] /Users/SergioBarroso/Desktop/T20-RAL Reviewers/Base de datos/T20.sav
```

Resumen de prueba de hipótesis

	Hipótesis nula	Test	Sig.	Decisión
1	La mediana de las diferencias entre CHOL POST-PRE Placebo y CHOL POST-PRE T20 es igual a 0.	Prueba de Wilcoxon de los rangos con signo de muestras relacionadas	.937	Retener la hipótesis nula.

Se muestran las significancias asintóticas. El nivel de significancia es .05.

Prueba de Wilcoxon de los rangos con signo de muestras relacionadas

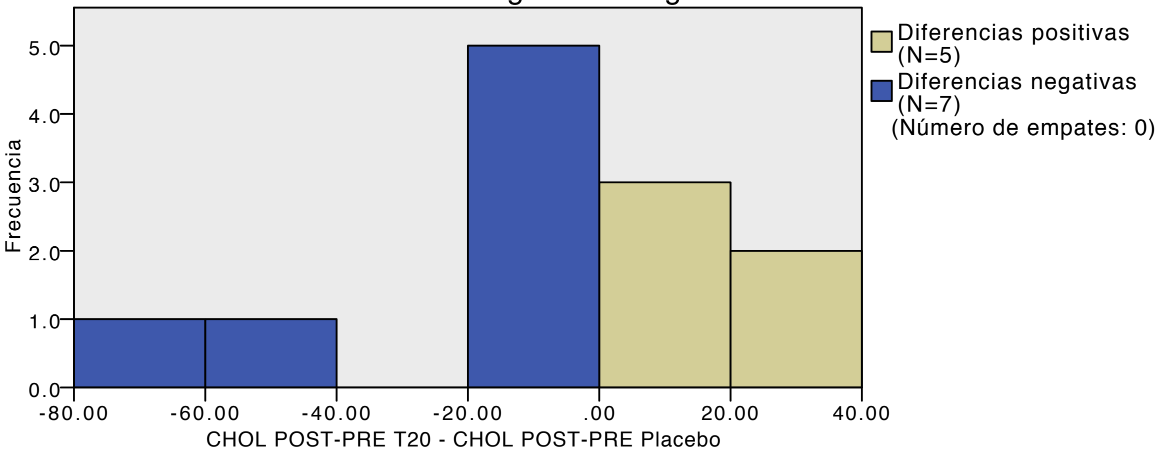

N total	12
Probar estadística	40.000
Error típico	12.743
Estadística de prueba estandarizada	.078
Sig. asintótica (prueba de dos caras)	.937

Información de campo continuo

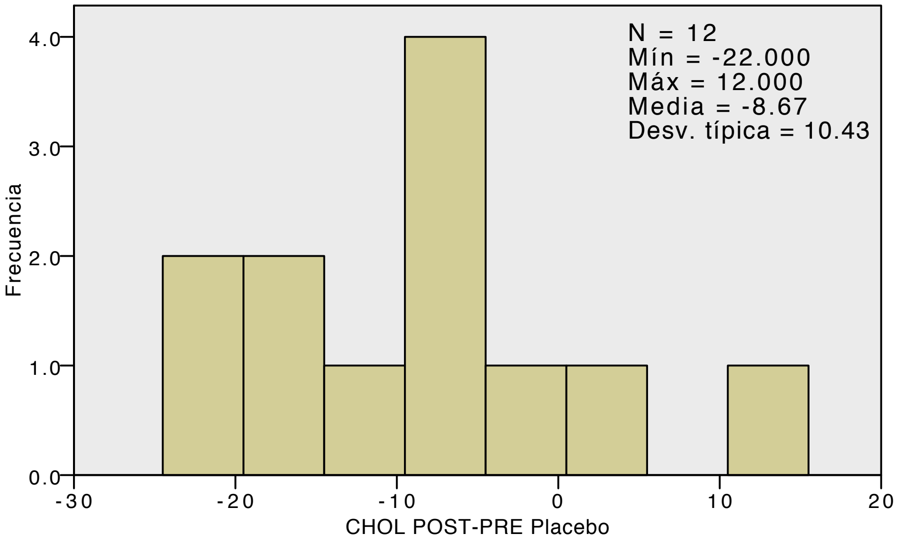

Información de campo continuo

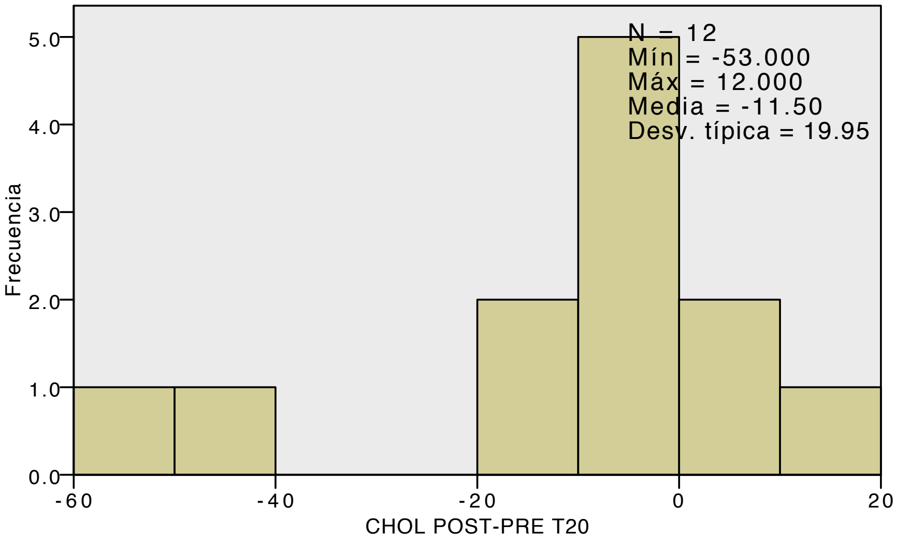


```
 *Nonparametric Tests: Related Samples.
NPTESTS
  /RELATED TEST(HDL.DIF.P HDL.DIF.T20) WILCOXON
  /MISSING SCOPE=ANALYSIS USERMISSING=EXCLUDE
  /CRITERIA ALPHA=0.05  CILEVEL=95.
```

Pruebas no paramétricas

Notas

**Resultados creados**		**23-MAR-2019 17:40:51**
**Comentarios**		
**Entrada**	**Datos**	**/Users/SergioBarroso/Desktop/T20-RAL Reviewers/Base de datos/T20.sav**
	**Conjunto de datos activo**	**Conjunto_de_datos1**
	**Filtro**	**<ninguno>**
	**Peso**	**<ninguno>**
	**Dividir archivo**	**<ninguno>**
	**Núm. de filas del archivo de trabajo**	**12**
**Sintaxis**		**NPTESTS /RELATED TEST(HDL.DIF.P HDL.DIF.T20) WILCOXON /MISSING SCOPE=ANALYSIS USERMISSING=EXCLUDE /CRITERIA ALPHA=0.05 CILEVEL=95.**
**Recursos**	**Tiempo de procesador**	**00:00:00.08**
	**Tiempo transcurrido**	**00:00:00.00**

```
[Conjunto_de_datos1] /Users/SergioBarroso/Desktop/T20-RAL Reviewers/Base de datos/T20.sav
```

Resumen de prueba de hipótesis

	Hipótesis nula	Test	Sig.	Decisión
1	La mediana de las diferencias entre HDL POST-PRE Placebo y HDL POST-PRE T20 es igual a 0.	Prueba de Wilcoxon de los rangos con signo de muestras relacionadas	.200	Retener la hipótesis nula.

Se muestran las significancias asintóticas. El nivel de significancia es .05.

Prueba de Wilcoxon de los rangos con signo de muestras relacionadas

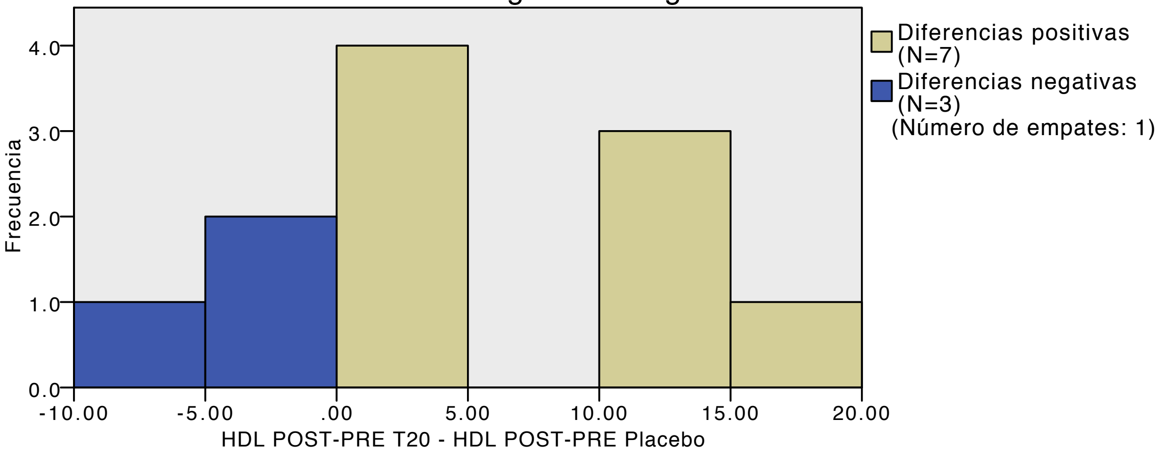

N total	11
Probar estadística	40.000
Error típico	9.760
Estadística de prueba estandarizada	1.281
Sig. asintótica (prueba de dos caras)	.200

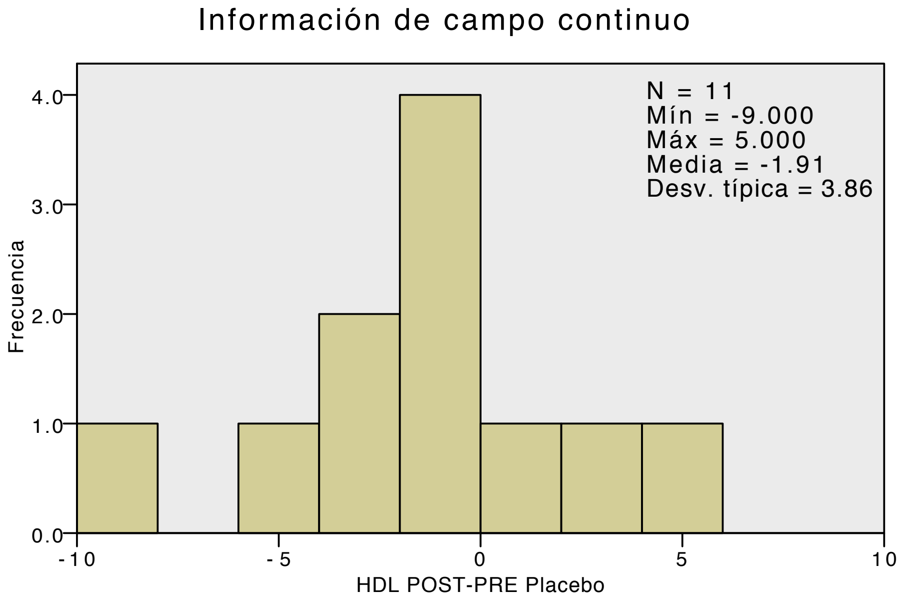

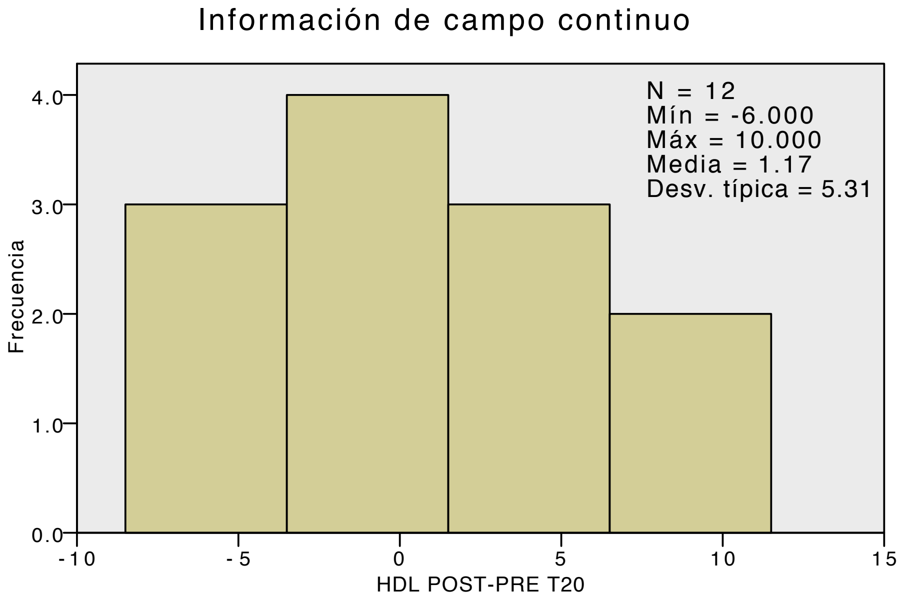


```
 *Nonparametric Tests: Related Samples.
NPTESTS
  /RELATED TEST(LDL.DIF.P LDL.DIF.T20) WILCOXON
  /MISSING SCOPE=ANALYSIS USERMISSING=EXCLUDE
  /CRITERIA ALPHA=0.05  CILEVEL=95.
```

Pruebas no paramétricas

Notas

Resultados creados		23-MAR-2019 17:41:21
Comentarios		
Entrada	Datos	/Users/SergioBarroso/Desktop/T20-RAL Reviewers/Base de datos/T20.sav
	Conjunto de datos activo	Conjunto_de_datos1
	Filtro	<ninguno>
	Peso	<ninguno>
	Dividir archivo	<ninguno>
	Núm. de filas del archivo de trabajo	12
Sintaxis		NPTESTS /RELATED TEST(LDL.DIF.P LDL.DIF.T20) WILCOXON /MISSING SCOPE=ANALYSIS USERMISSING=EXCLUDE /CRITERIA ALPHA=0.05 CILEVEL=95.
Recursos	Tiempo de procesador	00:00:00.08
	Tiempo transcurrido	00:00:00.00

```
[Conjunto_de_datos1] /Users/SergioBarroso/Desktop/T20-RAL Reviewers/Base de datos/T20.sav
```

Resumen de prueba de hipótesis

	Hipótesis nula	Test	Sig.	Decisión
1	La mediana de las diferencias entre LDL POST-PRE Placebo y LDL POST-PRE T20 es igual a 0.	Prueba de Wilcoxon de los rangos con signo de muestras relacionadas	.798	Retener la hipótesis nula.

Se muestran las significancias asintóticas. El nivel de significancia es .05.

Prueba de Wilcoxon de los rangos con signo de muestras relacionadas

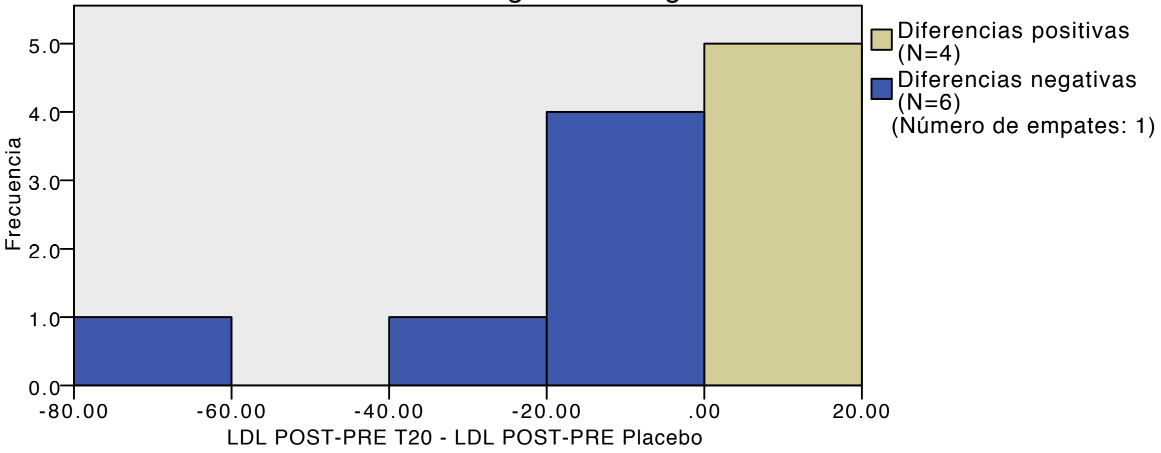

N total	11
Probar estadística	25.000
Error típico	9.785
Estadística de prueba estandarizada	-.255
Sig. asintótica (prueba de dos caras)	.798

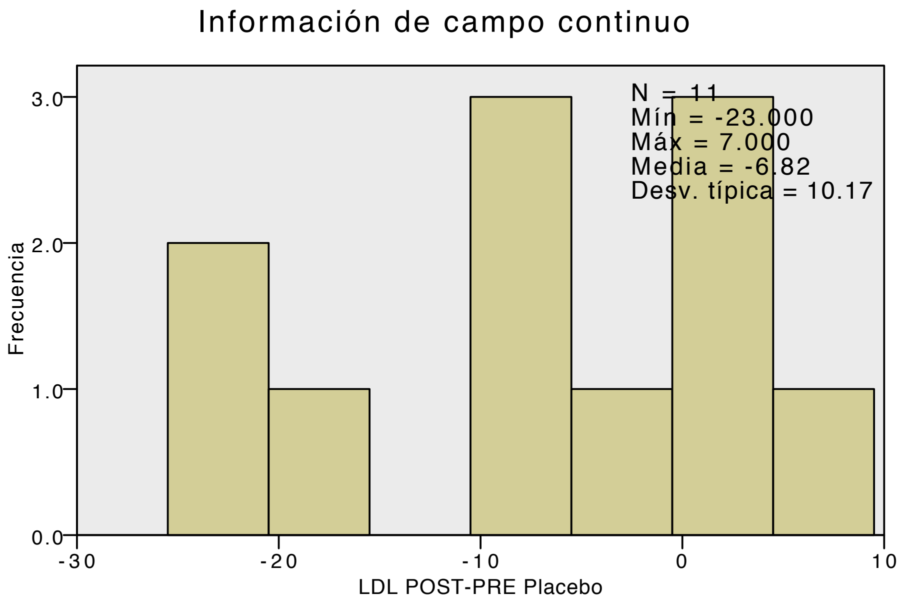

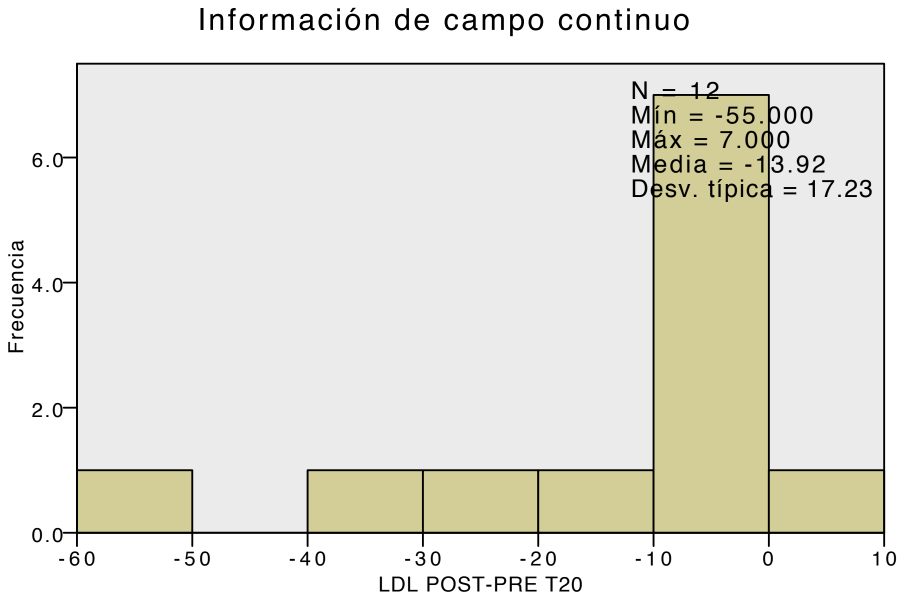


```
 *Nonparametric Tests: Related Samples.
NPTESTS
  /RELATED TEST(TAG.DIF.P TAG.DIF.T20) WILCOXON
  /MISSING SCOPE=ANALYSIS USERMISSING=EXCLUDE
  /CRITERIA ALPHA=0.05  CILEVEL=95.
```

Pruebas no paramétricas

Notas

Resultados creados		23-MAR-2019 17:41:48
Comentarios		
Entrada	Datos	/Users/SergioBarroso/Desktop/T20-RAL Reviewers/Base de datos/T20.sav
	Conjunto de datos activo	Conjunto_de_datos1
	Filtro	<ninguno>
	Peso	<ninguno>
	Dividir archivo	<ninguno>
	Núm. de filas del archivo de trabajo	12
Sintaxis		NPTESTS<br>/RELATED TEST(TAG.DIF.P TAG.DIF.T20) WILCOXON<br>/MISSING SCOPE=ANALYSIS USERMISSING=EXCLUDE<br>/CRITERIA ALPHA=0.05 CILEVEL=95.
Recursos	Tiempo de procesador	00:00:00.15
	Tiempo transcurrido	00:00:00.00

```
[Conjunto_de_datos1] /Users/SergioBarroso/Desktop/T20-RAL Reviewers/Base de datos/T20.sav
```

Resumen de prueba de hipótesis

	Hipótesis nula	Test	Sig.	Decisión
1	La mediana de las diferencias entre TAG POST-PRE Placebo y TAG POST-PRE T20 es igual a 0.	Prueba de Wilcoxon de los rangos con signo de muestras relacionadas	.695	Retener la hipótesis nula.

Se muestran las significancias asintóticas. El nivel de significancia es .05.

Prueba de Wilcoxon de los rangos con signo de muestras relacionadas

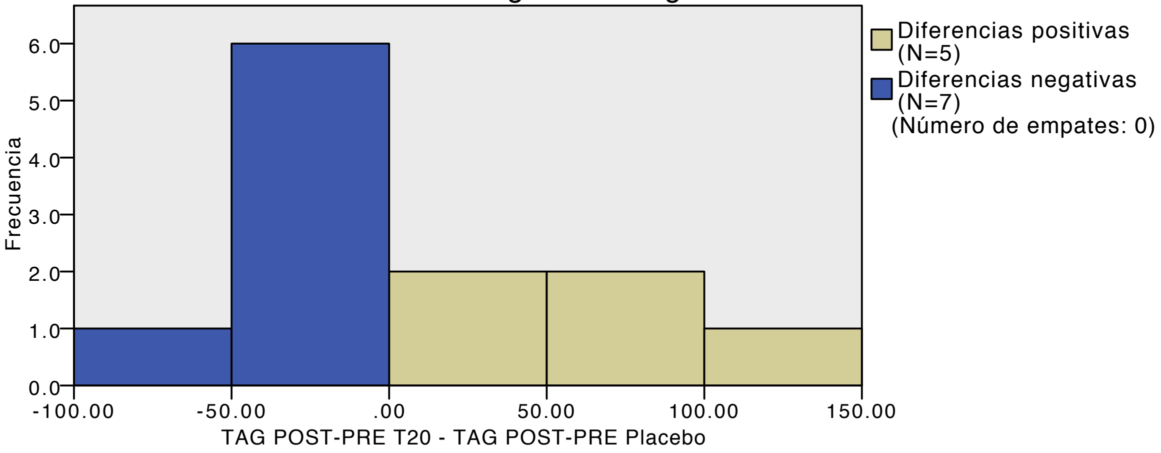

N total	12
Probar estadística	34.000
Error típico	12.743
Estadística de prueba estandarizada	-.392
Sig. asintótica (prueba de dos caras)	.695

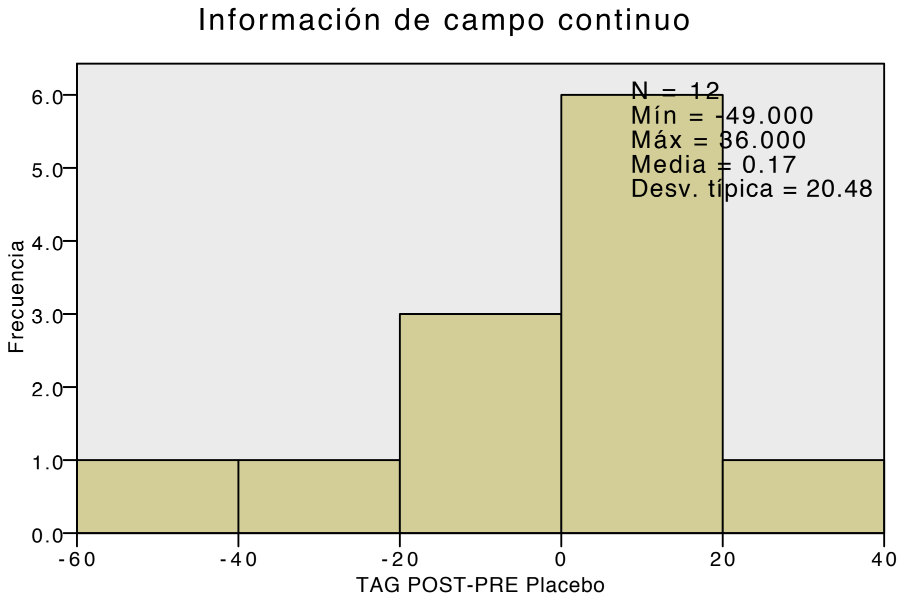

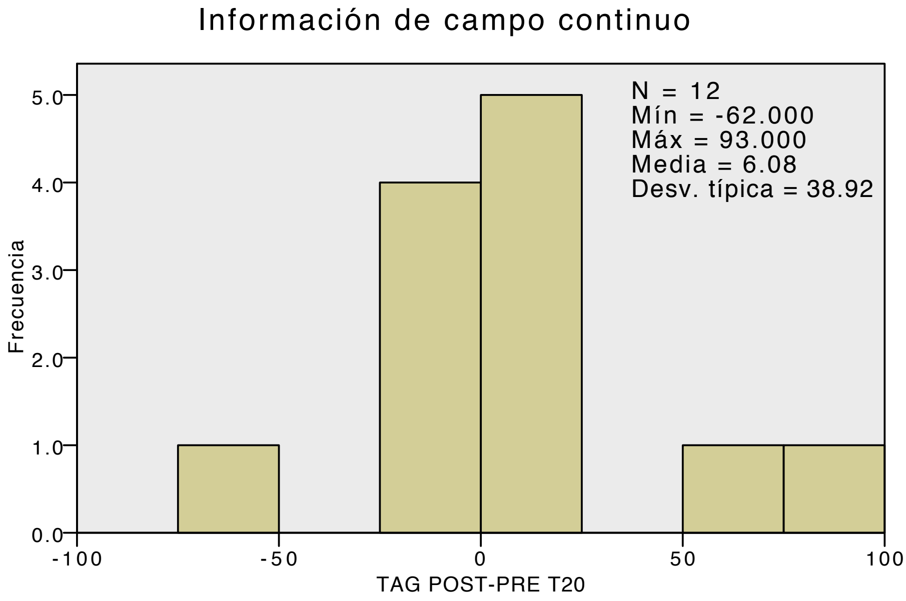


```
 *Nonparametric Tests: Related Samples.
NPTESTS
  /RELATED TEST(GLU.DIF.P GLU.DIF.T20) WILCOXON
  /MISSING SCOPE=ANALYSIS USERMISSING=EXCLUDE
  /CRITERIA ALPHA=0.05  CILEVEL=95.
```

Pruebas no paramétricas

Notas

Resultados creados		23-MAR-2019 17:42:06
Comentarios		
Entrada	Datos	/Users/SergioBarroso/Desktop/T20-RAL Reviewers/Base de datos/T20.sav
	Conjunto de datos activo	Conjunto_de_datos1
	Filtro	<ninguno>
	Peso	<ninguno>
	Dividir archivo	<ninguno>
	Núm. de filas del archivo de trabajo	12
Sintaxis		NPTESTS /RELATED TEST(GLU.DIF.P GLU.DIF.T20) WILCOXON /MISSING SCOPE=ANALYSIS USERMISSING=EXCLUDE /CRITERIA ALPHA=0.05 CILEVEL=95.
Recursos	Tiempo de procesador	00:00:00.09
	Tiempo transcurrido	00:00:00.00

[Conjunto_de_datos1] /Users/SergioBarroso/Desktop/T20-RAL Reviewers/Base de datos/T20.sav

Resumen de prueba de hipótesis

	Hipótesis nula	Test	Sig.	Decisión
1	La mediana de las diferencias entre GLU POST-PRE Placebo y GLU POST-PRE T20 es igual a 0.	Prueba de Wilcoxon de los rangos con signo de muestras relacionadas	.656	Retener la hipótesis nula.

Se muestran las significancias asintóticas. El nivel de significancia es .05.

Prueba de Wilcoxon de los rangos con signo de muestras relacionadas

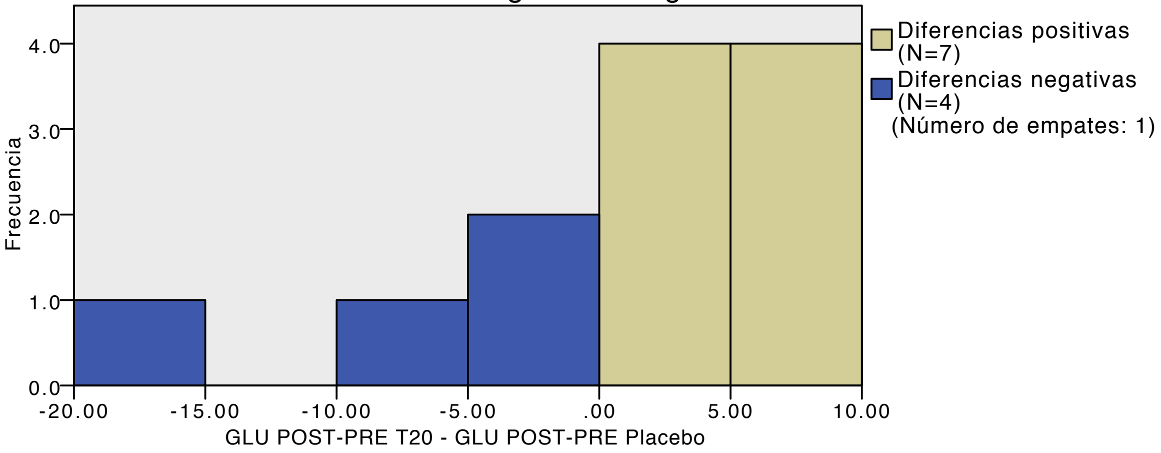

N total	12
Probar estadística	38.000
Error típico	11.231
Estadística de prueba estandarizada	.445
Sig. asintótica (prueba de dos caras)	.656

Información de campo continuo

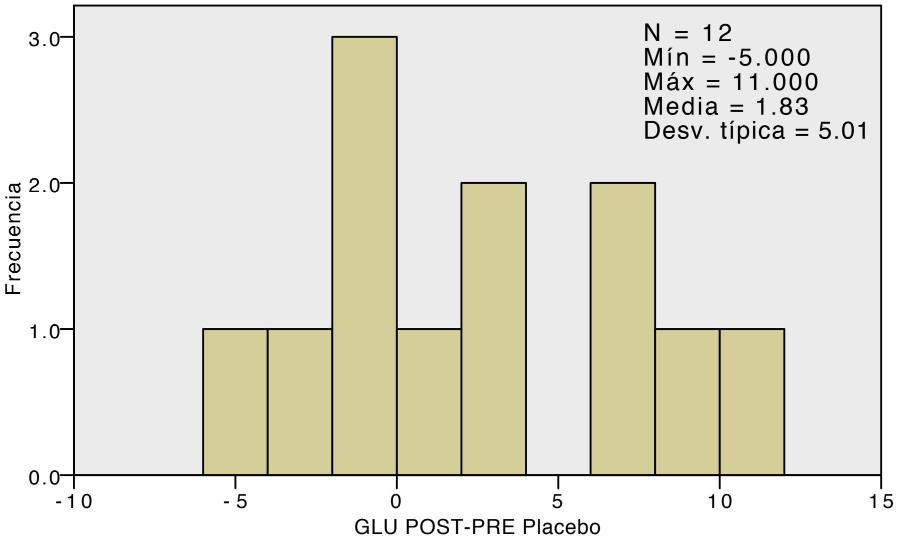

Información de campo continuo

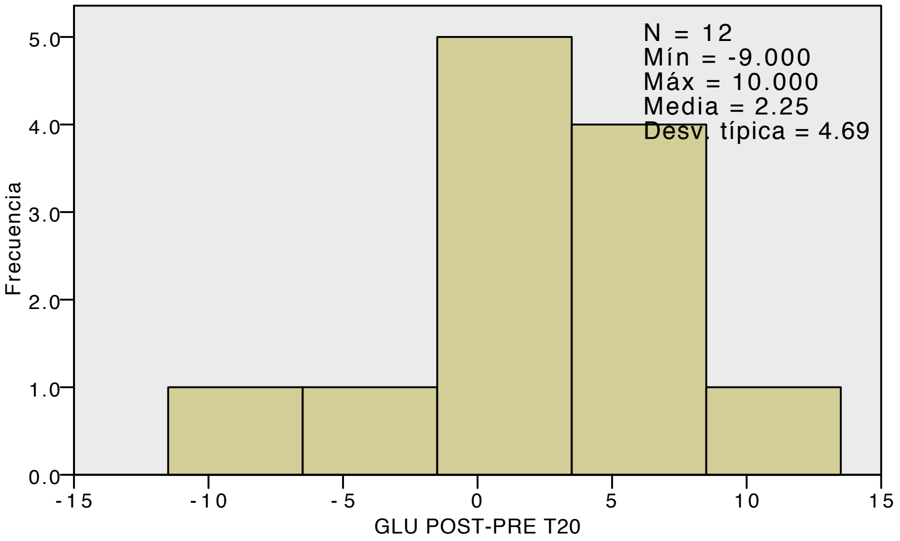


```
 *Nonparametric Tests: Related Samples.
NPTESTS
  /RELATED TEST(mtDNA.DIF.P mtDNA.DIF.T20) WILCOXON
  /MISSING SCOPE=ANALYSIS USERMISSING=EXCLUDE
  /CRITERIA ALPHA=0.05  CILEVEL=95.
```

Pruebas no paramétricas

Notas

Resultados creados		23-MAR-2019 17:42:33
Comentarios		
Entrada	Datos	/Users/SergioBarroso/Desktop/T20-RAL Reviewers/Base de datos/T20.sav
	Conjunto de datos activo	Conjunto_de_datos1
	Filtro	<ninguno>
	Peso	<ninguno>
	Dividir archivo	<ninguno>
	Núm. de filas del archivo de trabajo	12
Sintaxis		NPTESTS /RELATED TEST (mtDNA.DIF.P mtDNA.DIF.T20) WILCOXON /MISSING SCOPE=ANALYSIS USERMISSING=EXCLUDE /CRITERIA ALPHA=0.05 CILEVEL=95.
Recursos	Tiempo de procesador	00:00:00.09
	Tiempo transcurrido	00:00:00.00

```
[Conjunto_de_datos1] /Users/SergioBarroso/Desktop/T20-RAL Reviewers/Base de datos/T20.sav
```

Resumen de prueba de hipótesis

	Hipótesis nula	Test	Sig.	Decisión
1	La mediana de las diferencias entre mtDNA POST-PRE Placebo y mtDNA POST-PRE T20 es igual a 0.	Prueba de Wilcoxon de los rangos con signo de muestras relacionadas	.060	Retener la hipótesis nula.

Se muestran las significancias asintóticas. El nivel de significancia es .05.

Prueba de Wilcoxon de los rangos con signo de muestras relacionadas

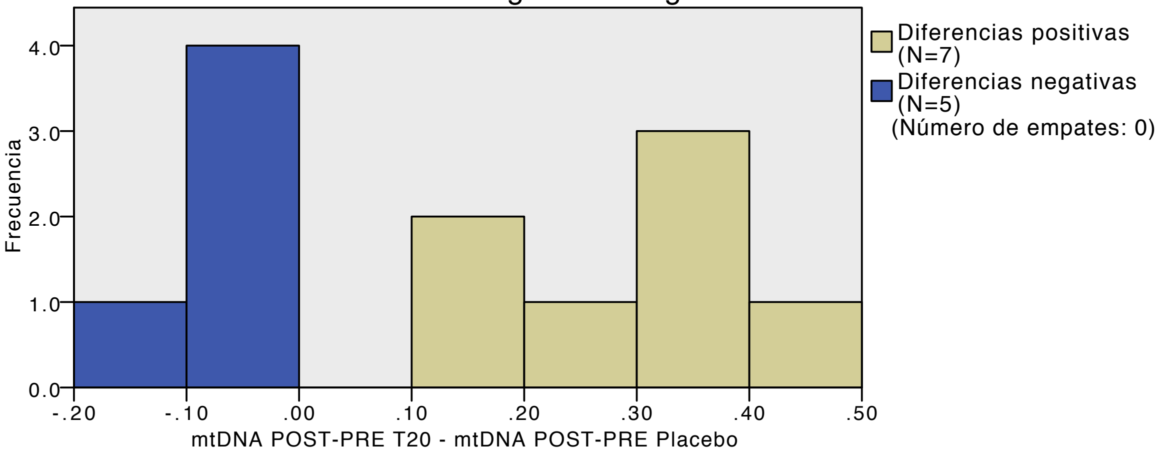

N total	12
Probar estadística	63.000
Error típico	12.748
Estadística de prueba estandarizada	1.883
Sig. asintótica (prueba de dos caras)	.060

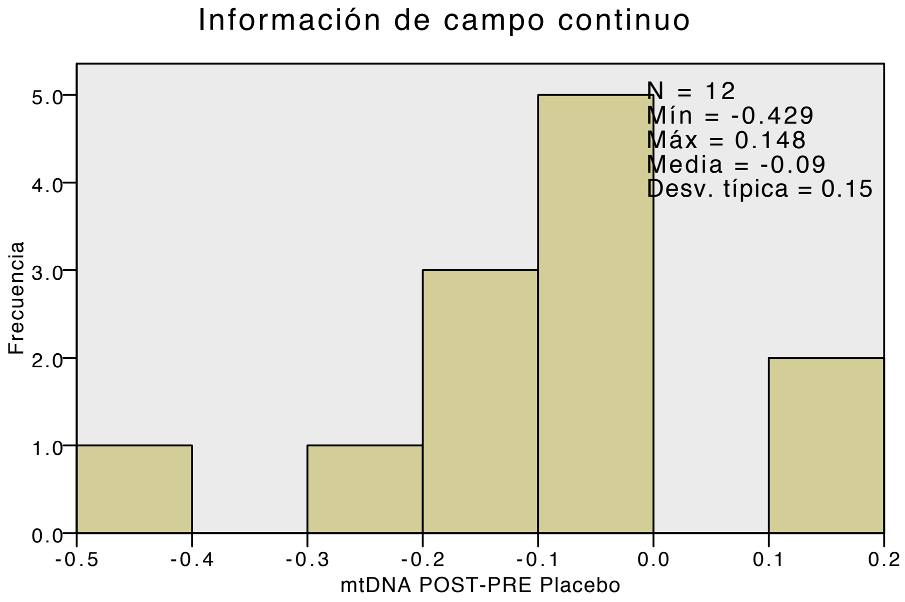

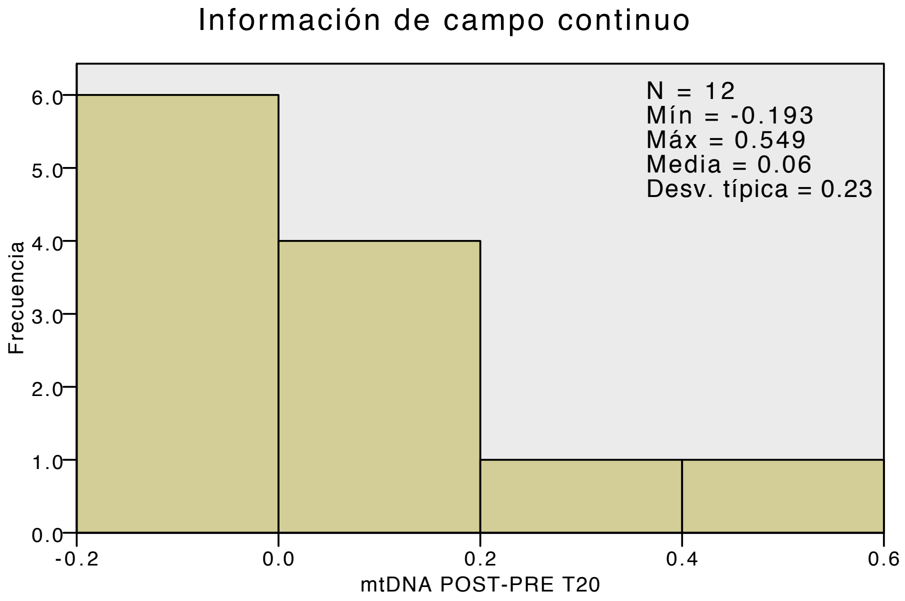


```
 *Nonparametric Tests: Related Samples.
NPTESTS
  /RELATED TEST(CRT.DIF.P CRT.DIF.T20) WILCOXON
  /MISSING SCOPE=ANALYSIS USERMISSING=EXCLUDE
  /CRITERIA ALPHA=0.05  CILEVEL=95.
```

Pruebas no paramétricas

Notas

**Resultados creados**		**23-MAR-2019 17:42:59**
**Comentarios**		
**Entrada**	**Datos**	**/Users/SergioBarroso/Desktop/T20-RAL Reviewers/Base de datos/T20.sav**
	**Conjunto de datos activo**	**Conjunto_de_datos1**
	**Filtro**	**<ninguno>**
	**Peso**	**<ninguno>**
	**Dividir archivo**	**<ninguno>**
	**Núm. de filas del archivo de trabajo**	**12**
**Sintaxis**		**NPTESTS /RELATED TEST(CRT.DIF.P CRT.DIF.T20) WILCOXON /MISSING SCOPE=ANALYSIS USERMISSING=EXCLUDE /CRITERIA ALPHA=0.05 CILEVEL=95.**
**Recursos**	**Tiempo de procesador**	**00:00:00.20**
	**Tiempo transcurrido**	**00:00:00.00**

```
[Conjunto_de_datos1] /Users/SergioBarroso/Desktop/T20-RAL Reviewers/Base de datos/T20.sav
```

Resumen de prueba de hipótesis

	Hipótesis nula	Test	Sig.	Decisión
1	La mediana de las diferencias entre CRT POST-PRE Placebo y CRT POST-PRE T20 es igual a 0.	Prueba de Wilcoxon de los rangos con signo de muestras relacionadas	.194	Retener la hipótesis nula.

Se muestran las significancias asintóticas. El nivel de significancia es .05.

Prueba de Wilcoxon de los rangos con signo de muestras relacionadas

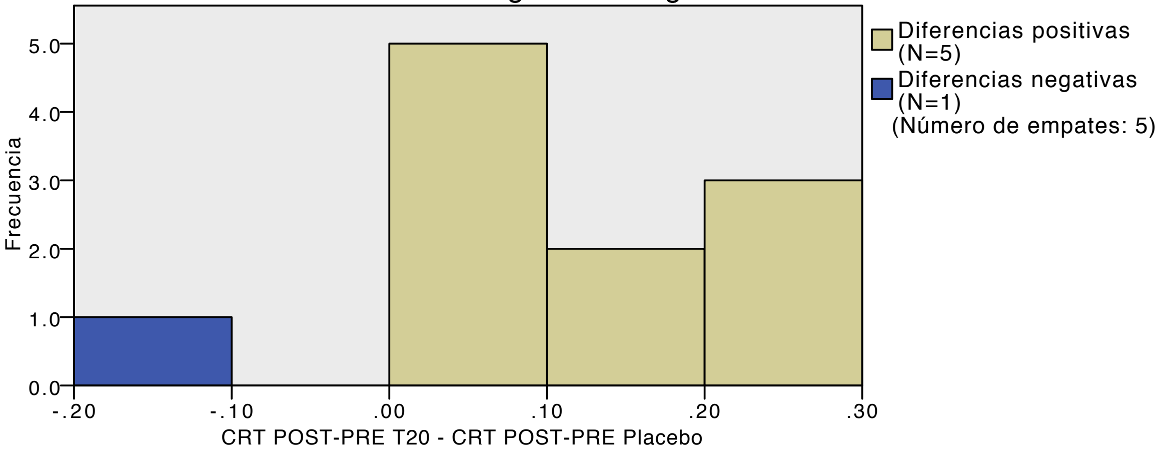

N total	11
Probar estadística	16.500
Error típico	4.623
Estadística de prueba estandarizada	1.298
Sig. asintótica (prueba de dos caras)	.194

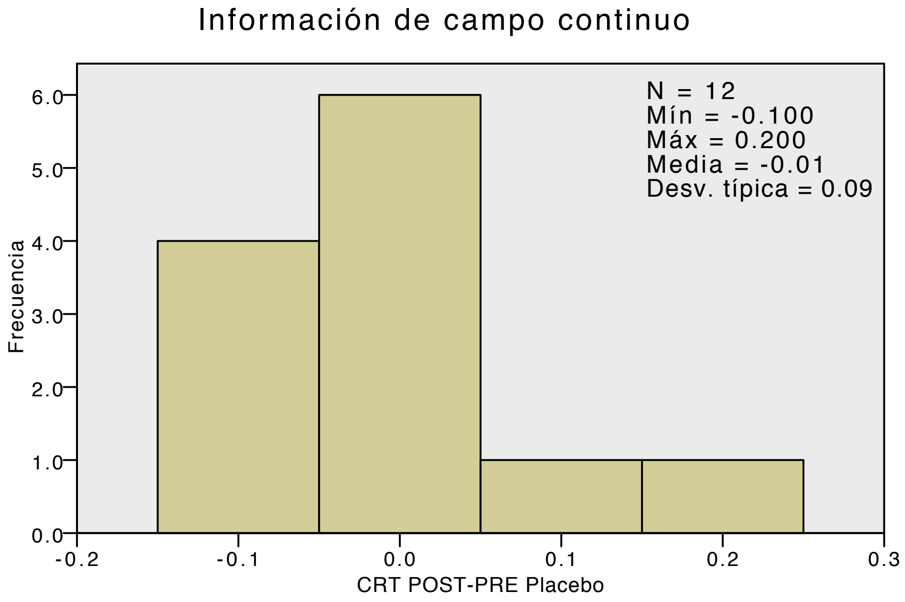

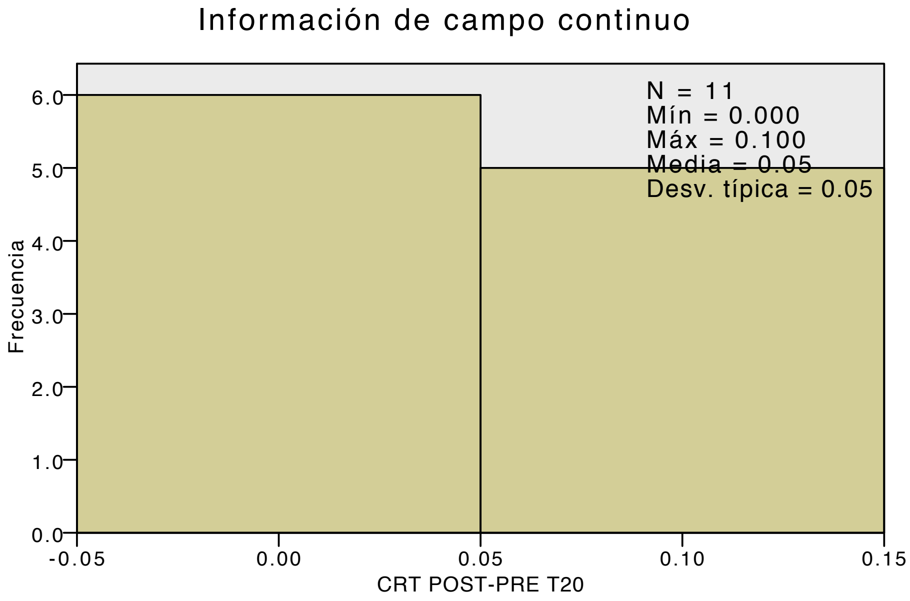


```
 *Nonparametric Tests: Related Samples.
NPTESTS
  /RELATED TEST(AST.DIF.P AST.DIF.T20) WILCOXON
  /MISSING SCOPE=ANALYSIS USERMISSING=EXCLUDE
  /CRITERIA ALPHA=0.05  CILEVEL=95.
```

Pruebas no paramétricas

Notas

**Resultados creados**		**23-MAR-2019 17:43:25**
**Comentarios**		
**Entrada**	**Datos**	**/Users/SergioBarroso/Desktop/T20-RAL Reviewers/Base de datos/T20.sav**
	**Conjunto de datos activo**	**Conjunto_de_datos1**
	**Filtro**	**<ninguno>**
	**Peso**	**<ninguno>**
	**Dividir archivo**	**<ninguno>**
	**Núm. de filas del archivo de trabajo**	**12**
**Sintaxis**		**NPTESTS /RELATED TEST(AST.DIF.P AST.DIF.T20) WILCOXON /MISSING SCOPE=ANALYSIS USERMISSING=EXCLUDE /CRITERIA ALPHA=0.05 CILEVEL=95.**
**Recursos**	**Tiempo de procesador**	**00:00:00.09**
	**Tiempo transcurrido**	**00:00:00.00**

```
[Conjunto_de_datos1] /Users/SergioBarroso/Desktop/T20-RAL Reviewers/Base de datos/T20.sav
```

Resumen de prueba de hipótesis

	Hipótesis nula	Test	Sig.	Decisión
1	La mediana de las diferencias entre AST POST-PRE Placebo y AST POST-PRE T20 es igual a 0.	Prueba de Wilcoxon de los rangos con signo de muestras relacionadas	.722	Retener la hipótesis nula.

Se muestran las significancias asintóticas. El nivel de significancia es .05.

Prueba de Wilcoxon de los rangos con signo de muestras relacionadas

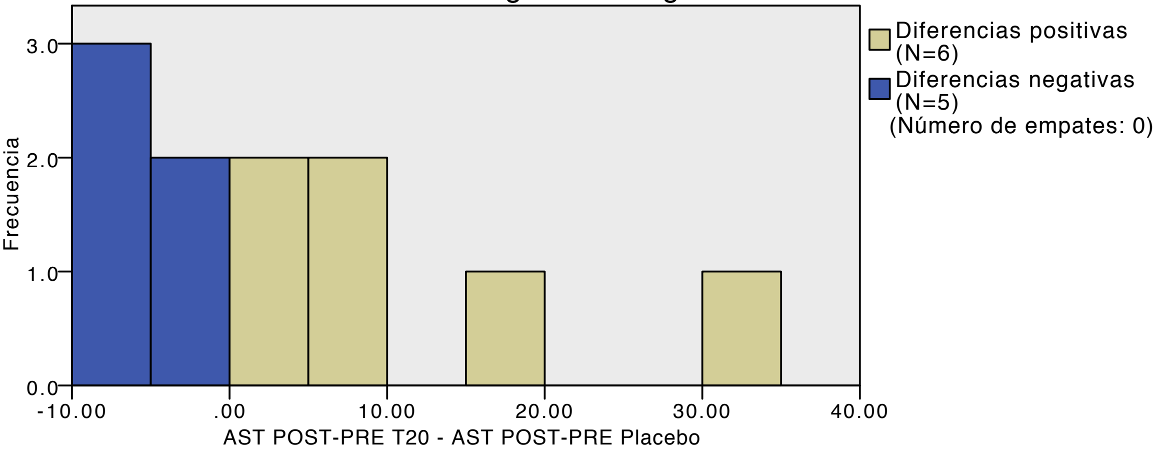

N total	11
Probar estadística	37.000
Error típico	11.242
Estadística de prueba estandarizada	.356
Sig. asintótica (prueba de dos caras)	.722

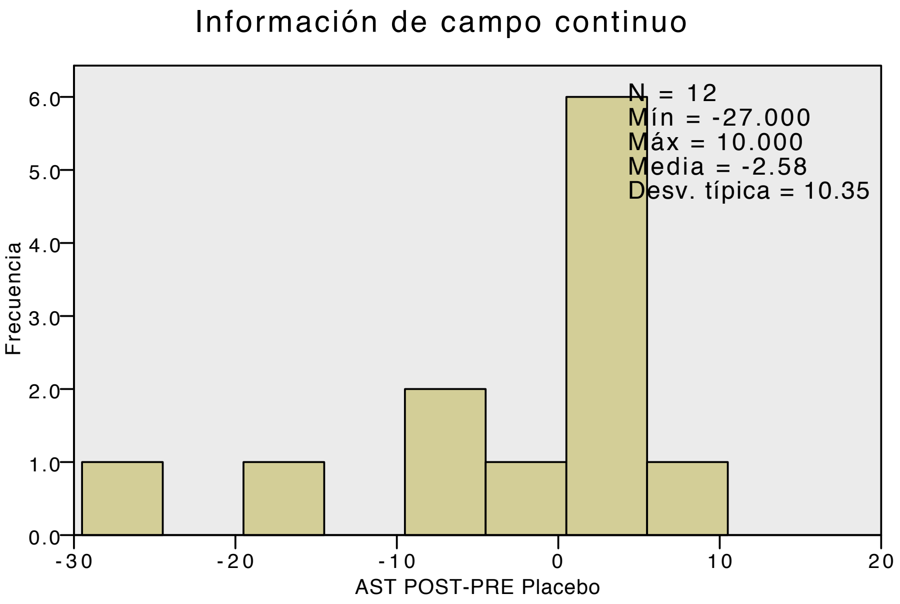

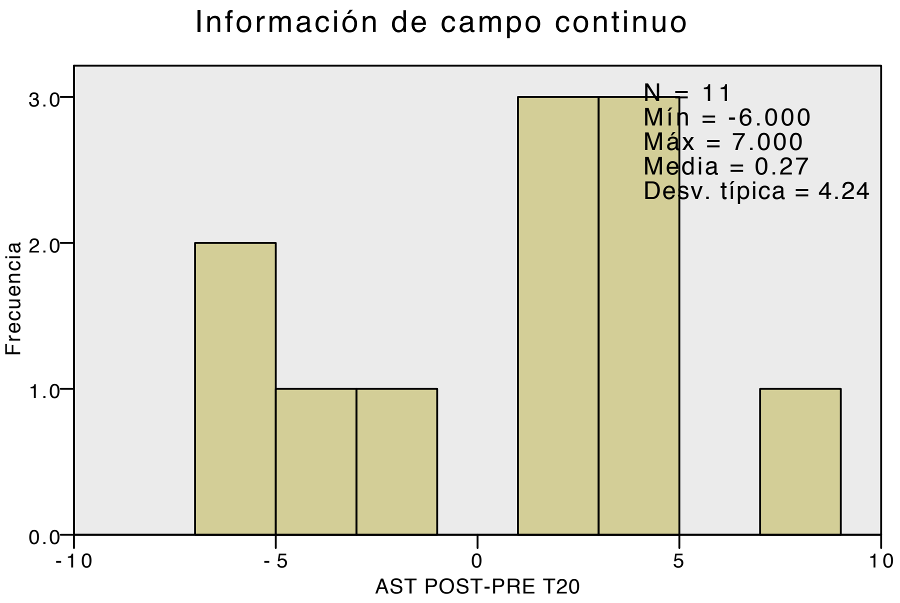


```
 *Nonparametric Tests: Related Samples.
NPTESTS
  /RELATED TEST(ALT.DIF.P ALT.DIF.T20) WILCOXON
  /MISSING SCOPE=ANALYSIS USERMISSING=EXCLUDE
  /CRITERIA ALPHA=0.05  CILEVEL=95.
```

Pruebas no paramétricas

Notas

Resultados creados		23-MAR-2019 17:43:48
Comentarios		
Entrada	Datos	/Users/SergioBarroso/Desktop/T20-RAL Reviewers/Base de datos/T20.sav
	Conjunto de datos activo	Conjunto_de_datos1
	Filtro	<ninguno>
	Peso	<ninguno>
	Dividir archivo	<ninguno>
	Núm. de filas del archivo de trabajo	12
Sintaxis		NPTESTS /RELATED TEST(ALT.DIF.P ALT.DIF.T20) WILCOXON /MISSING SCOPE=ANALYSIS USERMISSING=EXCLUDE /CRITERIA ALPHA=0.05 CILEVEL=95.
Recursos	Tiempo de procesador	00:00:00.14
	Tiempo transcurrido	00:00:00.00

[Conjunto_de_datos1] /Users/SergioBarroso/Desktop/T20-RAL Reviewers/Base de datos/T20.sav

Resumen de prueba de hipótesis

	Hipótesis nula	Test	Sig.	Decisión
1	La mediana de las diferencias entre ALT POST-PRE Placebo y ALT POST-PRE T20 es igual a 0.	Prueba de Wilcoxon de los rangos con signo de muestras relacionadas	.192	Retener la hipótesis nula.

Se muestran las significancias asintóticas. El nivel de significancia es .05.

Prueba de Wilcoxon de los rangos con signo de muestras relacionadas

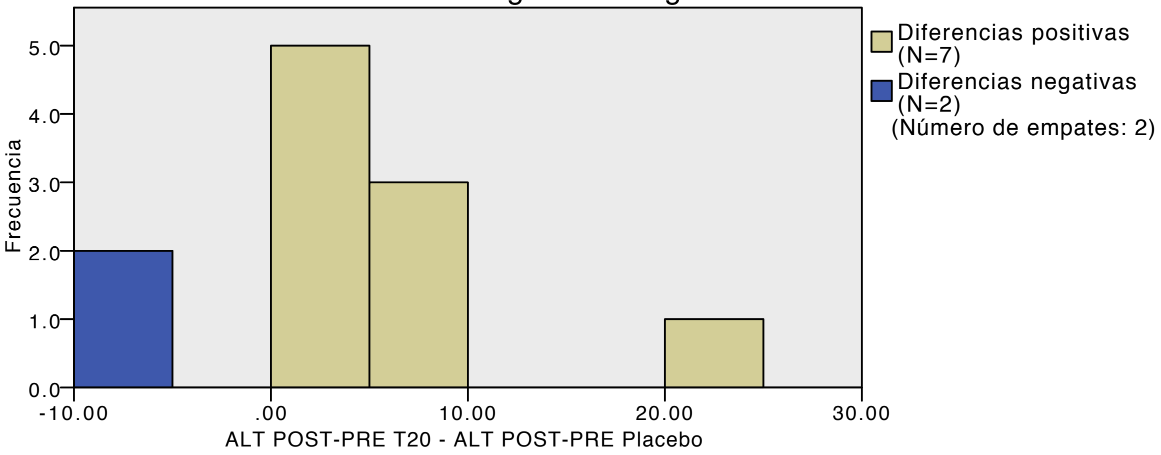

N total	11
Probar estadística	33.500
Error típico	8.426
Estadística de prueba estandarizada	1.305
Sig. asintótica (prueba de dos caras)	.192

Información de campo continuo

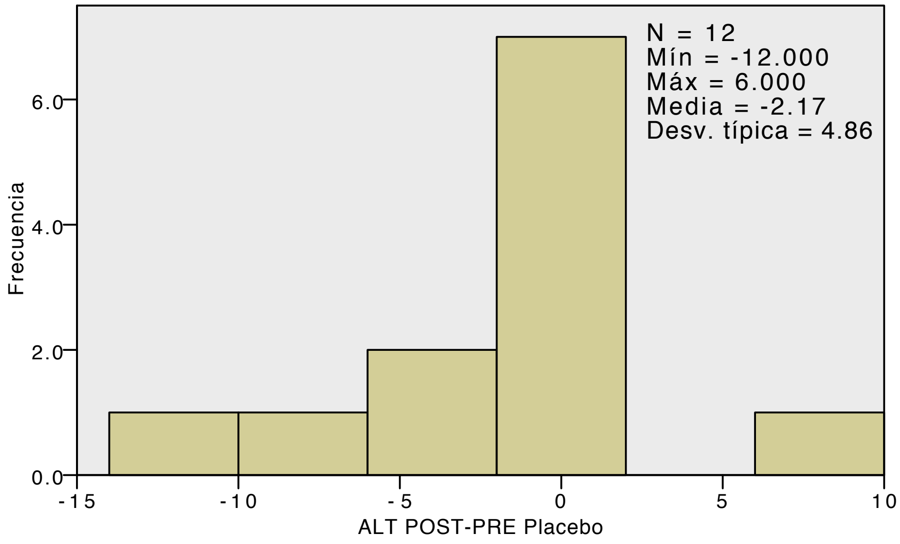

Información de campo continuo

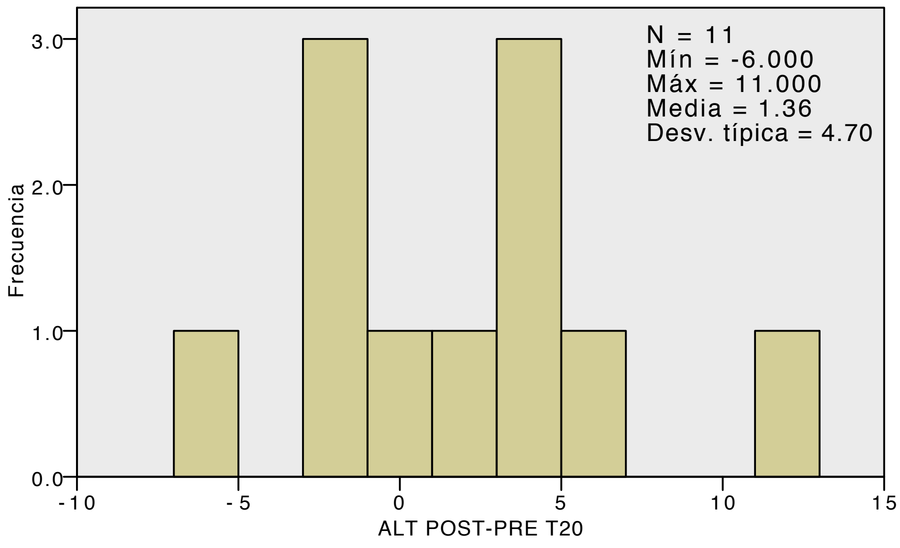


```
 *Nonparametric Tests: Related Samples.
NPTESTS
  /RELATED TEST(BIL.DIF.P BIL.DIF.T20) WILCOXON
  /MISSING SCOPE=ANALYSIS USERMISSING=EXCLUDE
  /CRITERIA ALPHA=0.05  CILEVEL=95.
```

Pruebas no paramétricas

Notas

Resultados creados		23-MAR-2019 17:44:12
Comentarios		
Entrada	Datos	/Users/SergioBarroso/Desktop/T20-RAL Reviewers/Base de datos/T20.sav
	Conjunto de datos activo	Conjunto_de_datos1
	Filtro	<ninguno>
	Peso	<ninguno>
	Dividir archivo	<ninguno>
	Núm. de filas del archivo de trabajo	12
Sintaxis		NPTESTS /RELATED TEST(BIL.DIF.P BIL.DIF.T20) WILCOXON /MISSING SCOPE=ANALYSIS USERMISSING=EXCLUDE /CRITERIA ALPHA=0.05 CILEVEL=95.
Recursos	Tiempo de procesador	00:00:00.10
	Tiempo transcurrido	00:00:00.00

[Conjunto_de_datos1] /Users/SergioBarroso/Desktop/T20-RAL Reviewers/Base de datos/T20.sav

Resumen de prueba de hipótesis

	Hipótesis nula	Test	Sig.	Decisión
1	La mediana de las diferencias entre BIL POST-PRE Placebo y BIL POST-PRE T20 es igual a 0.	Prueba de Wilcoxon de los rangos con signo de muestras relacionadas	.349	Retener la hipótesis nula.

Se muestran las significancias asintóticas. El nivel de significancia es .05.

Prueba de Wilcoxon de los rangos con signo de muestras relacionadas

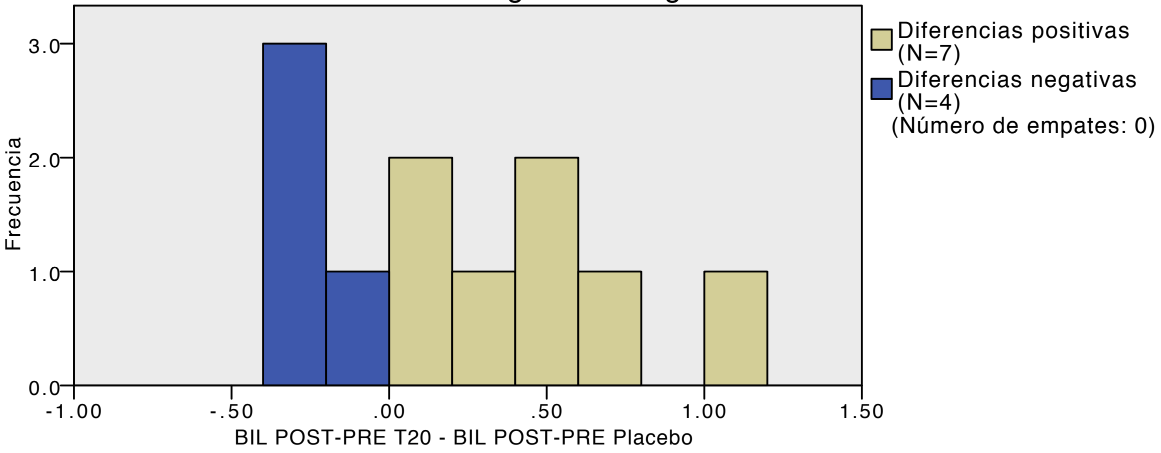

N total	11
Probar estadística	43.500
Error típico	11.214
Estadística de prueba estandarizada	.936
Sig. asintótica (prueba de dos caras)	.349

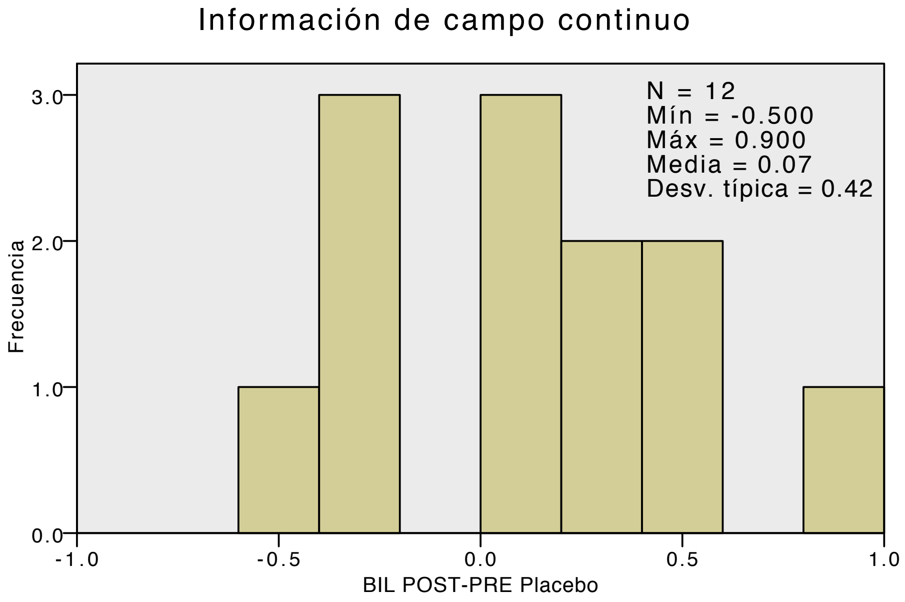

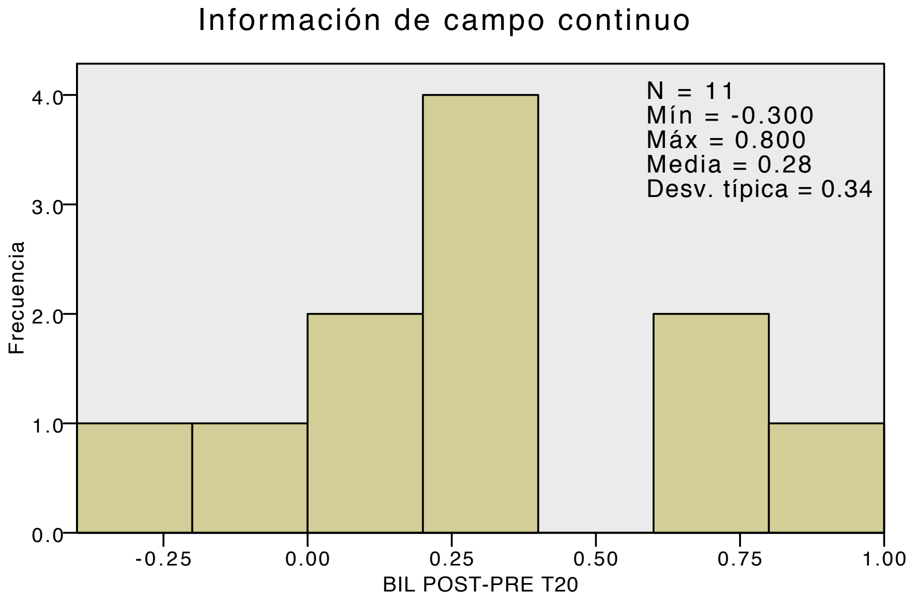


```
 *Nonparametric Tests: Related Samples.
NPTESTS
  /RELATED TEST(PROT.DIF.P PROT.DIF.T20) WILCOXON
  /MISSING SCOPE=ANALYSIS USERMISSING=EXCLUDE
  /CRITERIA ALPHA=0.05  CILEVEL=95.
```

Pruebas no paramétricas

Notas

**Resultados creados**		**23-MAR-2019 17:44:28**
**Comentarios**		
**Entrada**	**Datos**	**/Users/SergioBarroso/Desktop/T20-RAL Reviewers/Base de datos/T20.sav**
	**Conjunto de datos activo**	**Conjunto_de_datos1**
	**Filtro**	**<ninguno>**
	**Peso**	**<ninguno>**
	**Dividir archivo**	**<ninguno>**
	**Núm. de filas del archivo de trabajo**	**12**
**Sintaxis**		**NPTESTS /RELATED TEST(PROT.DIF.P PROT.DIF.T20) WILCOXON /MISSING SCOPE=ANALYSIS USERMISSING=EXCLUDE /CRITERIA ALPHA=0.05 CILEVEL=95.**
**Recursos**	**Tiempo de procesador**	**00:00:00.08**
	**Tiempo transcurrido**	**00:00:00.00**

```
[Conjunto_de_datos1] /Users/SergioBarroso/Desktop/T20-RAL Reviewers/Base de datos/T20.sav
```

Resumen de prueba de hipótesis

	Hipótesis nula	Test	Sig.	Decisión
1	La mediana de las diferencias entre PROT POST-PRE Placebo y PROT POST-PRE T20 es igual a 0.	Prueba de Wilcoxon de los rangos con signo de muestras relacionadas	.473	Retener la hipótesis nula.

Se muestran las significancias asintóticas. El nivel de significancia es .05.

Prueba de Wilcoxon de los rangos con signo de muestras relacionadas

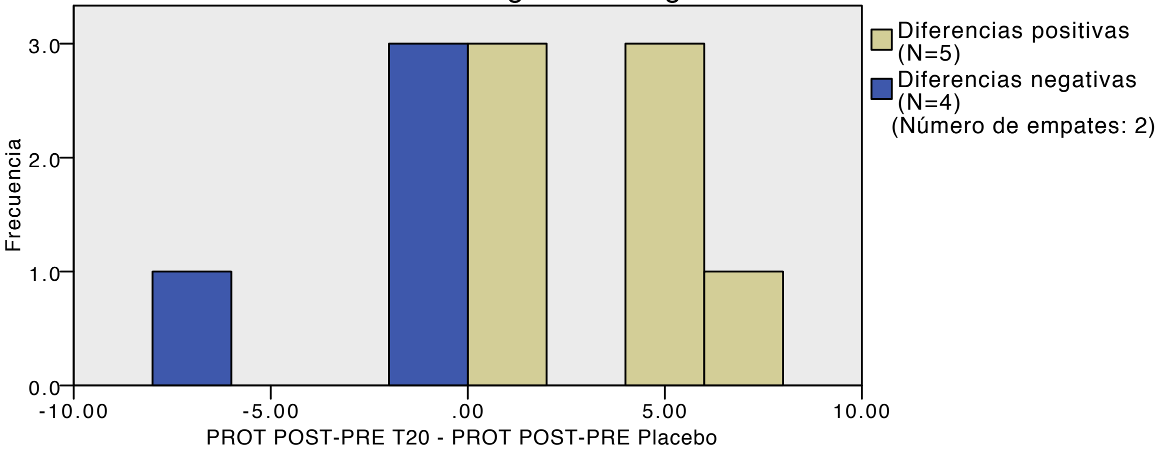

N total	11
Probar estadística	28.500
Error típico	8.359
Estadística de prueba estandarizada	.718
Sig. asintótica (prueba de dos caras)	.473

Información de campo continuo

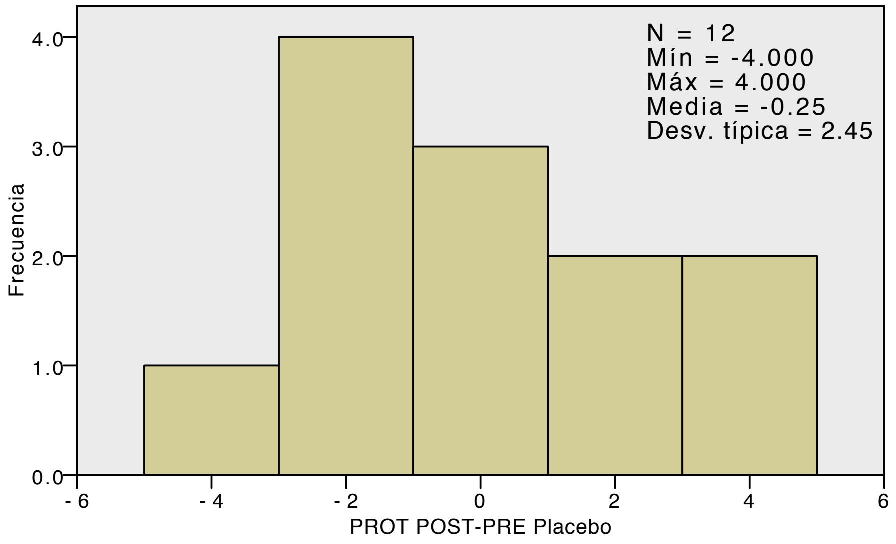

Información de campo continuo

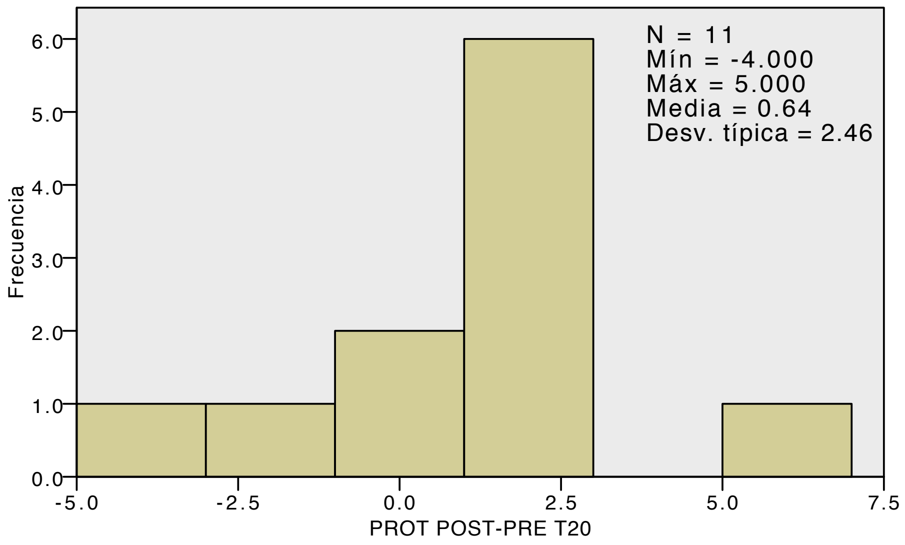

Supplement: S4 File — File containing SPSS output for descriptive data and Wilcoxon test for the differences between PRE values and the change. (PDF) [file pone.0216712.s004.pdf]
