# Supplementary material for: Metabolic, mitochondrial, renal and hepatic safety of enfuvirtide and raltegravir antiretroviral administration: Randomized crossover clinical trial in healthy volunteers
Source: PLoS One. 2019 May 23;14(5):e0216712. doi: 10.1371/journal.pone.0216712 (PMC6532851; doi:10.1371/journal.pone.0216712)
Supplement: S5 File — File containing SPSS output for descriptive data and Wilcoxon test for the differences between PRE values and the change. (PDF) [file pone.0216712.s005.pdf]

```
 GET
  FILE='/Users/SergioBarroso/Desktop/T20-RAL Reviewers/Base de datos/RAL.sav'.
DATASET NAME Conjunto_de_datos1 WINDOW=FRONT.
DESCRIPTIVES VARIABLES=CHOL.PRE.P CHOL.POST.P CHOL.PRE.RAL CHOL.POST.RAL HDL.PRE.P HDL.POST.P
PRO.POST.P PRO.PRE.RAL PROT.POST.RAL
  /STATISTICS=MEAN STDDEV SEMEAN.
```

Descriptivos

Notas

Resultados creados		29-MAR-2019 00:35:53
Comentarios		
Entrada	Datos	/Users/SergioBarroso/Desktop/T20-RAL Reviewers/Base de datos/RAL.sav
	Conjunto de datos activo	Conjunto_de_datos1
	Filtro	<ninguno>
	Peso	<ninguno>
	Dividir archivo	<ninguno>
	Núm. de filas del archivo de trabajo	14
Manipulación de los valores perdidos	Definición de los perdidos	Los valores perdidos definidos por el usuario son considerados como perdidos.
	Casos utilizados	Se han utilizado todos los datos no perdidos.
Sintaxis		DESCRIPTIVES VARIABLES=CHOL.PRE.P CHOL.POST.P CHOL.PRE.RAL CHOL.POST.RAL HDL.PRE.P HDL.POST.P HDL.PRE.RAL HDL.POST.RAL LDL.PRE.P LDL.POST.P LDL.PRE.RAL LDL.POST.RAL TAG.PRE.P TAG.POST.P TAG.PRE.RAL TAG.POST.RAL GLU.PRE.P GLU.POST.P GLU.PRE.RAL GLU.POST.RAL mtDNA.PRE.P mtDNA.POST.P mtDNA.PRE.RAL mtDNA.POST.RAL CRT.PRE.P CRT.POST.P CRT.PRE.RAL CRT.POST.RAL AST.PRE.P AST.POST.P AST.PRE.RAL AST.POST.RAL ALT.PRE.P ALT.POST.P ALT.PRE.RAL ALT.POST.RAL BIL.PRE.P BIL.POST.P BIL.PRE.RAL BIL.POST.RAL PROT.PRE.P PRO.POST.P PRO.PRE.RAL PROT.POST.RAL /STATISTICS=MEAN STDDEV SEMEAN.
Recursos	Tiempo de procesador	00:00:00.01
	Tiempo transcurrido	00:00:00.00

```
[Conjunto_de_datos1] /Users/SergioBarroso/Desktop/T20-RAL Reviewers/Base de datos/RAL.sav
```

Estadísticos descriptivos

	N	Media		Desv. típ.
	Estadístico	Estadístico	Error típico	Estadístico
CHOL.PRE.P	14	155.2857	7.90703	29.58541
CHOL.POST.P	14	152.7143	7.61392	28.48867
CHOL.PRE.RAL	14	152.5714	7.69166	28.77957
CHOL.POST.RAL	14	154.3571	8.29246	31.02755
HDL.PRE.P	14	50.8571	2.53298	9.47756
HDL.POST.P	14	50.9286	2.27375	8.50759
HDL.PRE.RAL	14	50.7857	2.63572	9.86196
HDL.POST.RAL	14	52.0714	2.49717	9.34356
LDL.PRE.P	14	85.7857	6.36541	23.81718
LDL.POST.P	14	88.4286	6.81120	25.48518
LDL.PRE.RAL	14	79.7143	5.59378	20.93002
LDL.POST.RAL	14	88.8571	7.57734	28.35179
TAG.PRE.P	14	92.7857	14.51001	54.29149
TAG.POST.P	14	66.6429	7.19936	26.93755
TAG.PRE.RAL	14	110.1429	22.25202	83.25943
TAG.POST.RAL	14	67.6429	7.19096	26.90612
GLU.PRE.P	14	87.4286	2.22551	8.32710
GLU.POST.P	14	87.7143	1.36046	5.09039
GLU.PRE.RAL	14	87.7857	1.50887	5.64567
GLU.POST.RAL	14	85.0714	1.76182	6.59212
mtDNA.PRE.P	14	1.7474	.15065	.56369
mtDNA.POST.P	14	1.9171	.15601	.58373
mtDNA.PRE.RAL	14	1.7625	.16524	.61827
mtDNA.POST.RAL	14	1.9960	.16727	.62588
CRT.PRE.P	14	.9893	.01820	.06810
CRT.POST.P	14	1.0164	.02517	.09419
CRT.PRE.RAL	14	.9986	.02265	.08475
CRT.POST.RAL	14	1.0200	.02169	.08115
AST.PRE.P	14	23.9286	1.23797	4.63207
AST.POST.P	14	23.9286	1.25122	4.68162
AST.PRE.RAL	14	28.0714	3.37161	12.61540
AST.POST.RAL	14	26.5714	2.91022	10.88905
ALT.PRE.P	14	25.0714	2.46618	9.22759
ALT.POST.P	14	26.2143	3.43092	12.83732
ALT.PRE.RAL	14	28.0714	4.11839	15.40961
ALT.POST.RAL	14	25.7143	1.90554	7.12988
BIL.PRE.P	14	.6929	.08546	.31977
BIL.POST.P	14	.7643	.07079	.26489
BIL.PRE.RAL	14	.7143	.08042	.30091
BIL.POST.RAL	14	.8286	.09913	.37092
PROT.PRE.P	14	74.7143	.84794	3.17269
PRO.POST.P	13	75.0000	.63043	2.27303
PRO.PRE.RAL	14	73.2857	.87347	3.26823
PROT.POST.RAL	14	75.4286	.95339	3.56725
N válido (según lista)	13			

```
 EXECUTE.
*Nonparametric Tests: Related Samples.
NPTESTS
  /RELATED TEST(CHOL.PRE.P CHOL.PRE.RAL) WILCOXON
  /MISSING SCOPE=ANALYSIS USERMISSING=EXCLUDE
  /CRITERIA ALPHA=0.05  CILEVEL=95.
```

Pruebas no paramétricas

Notas

Resultados creados		23-MAR-2019 18:09:54
Comentarios		
Entrada	Datos	/Users/SergioBarroso/Desktop/T20-RAL Reviewers/Base de datos/RAL.sav
	Conjunto de datos activo	Conjunto_de_datos2
	Filtro	<ninguno>
	Peso	<ninguno>
	Dividir archivo	<ninguno>
	Núm. de filas del archivo de trabajo	14
Sintaxis		NPTESTS /RELATED TEST(CHOL.PRE.P CHOL.PRE.RAL) WILCOXON /MISSING SCOPE=ANALYSIS USERMISSING=EXCLUDE /CRITERIA ALPHA=0.05 CILEVEL=95.
Recursos	Tiempo de procesador	00:00:00.08
	Tiempo transcurrido	00:00:00.00

```
[Conjunto_de_datos2] /Users/SergioBarroso/Desktop/T20-RAL Reviewers/Base de datos/RAL.sav
```

Resumen de prueba de hipótesis

	Hipótesis nula	Test	Sig.	Decisión
1	La mediana de las diferencias entre CHOL.PRE.P y CHOL.PRE.RAL es igual a 0.	Prueba de Wilcoxon de los rangos con signo de muestras relacionadas	.366	Retener la hipótesis nula.

Se muestran las significancias asintóticas. El nivel de significancia es .05.

Prueba de Wilcoxon de los rangos con signo de muestras relacionadas

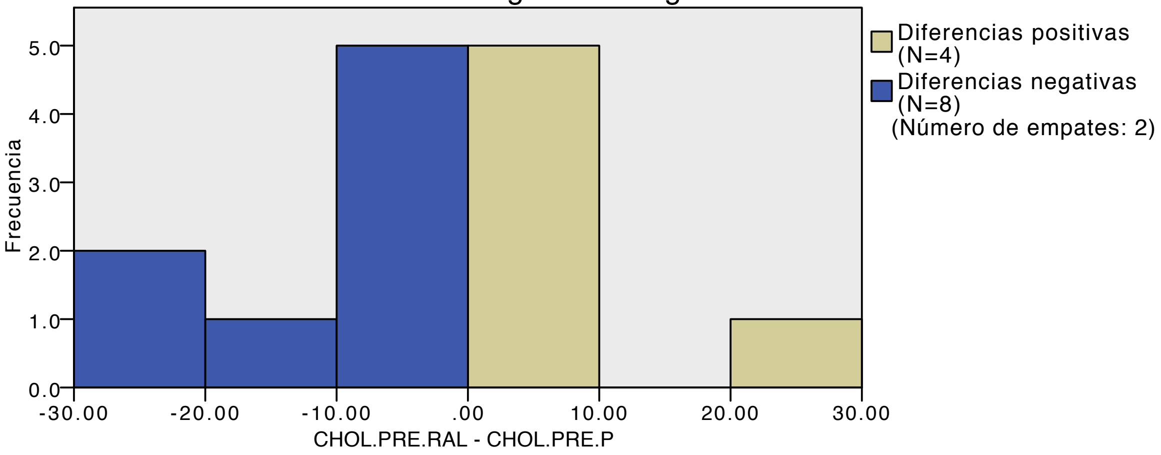

N total	14
Probar estadística	27.500
Error típico	12.733
Estadística de prueba estandarizada	-.903
Sig. asintótica (prueba de dos caras)	.366

Información de campo continuo

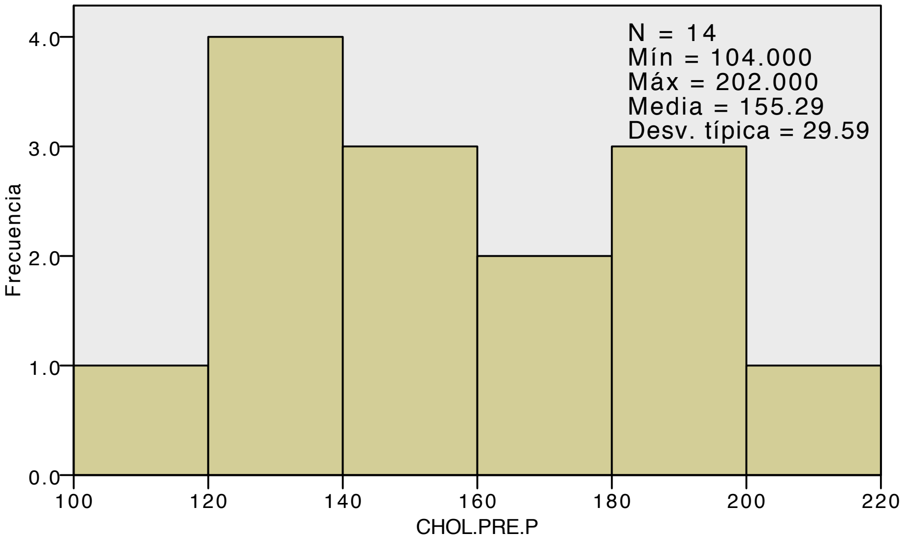

Información de campo continuo

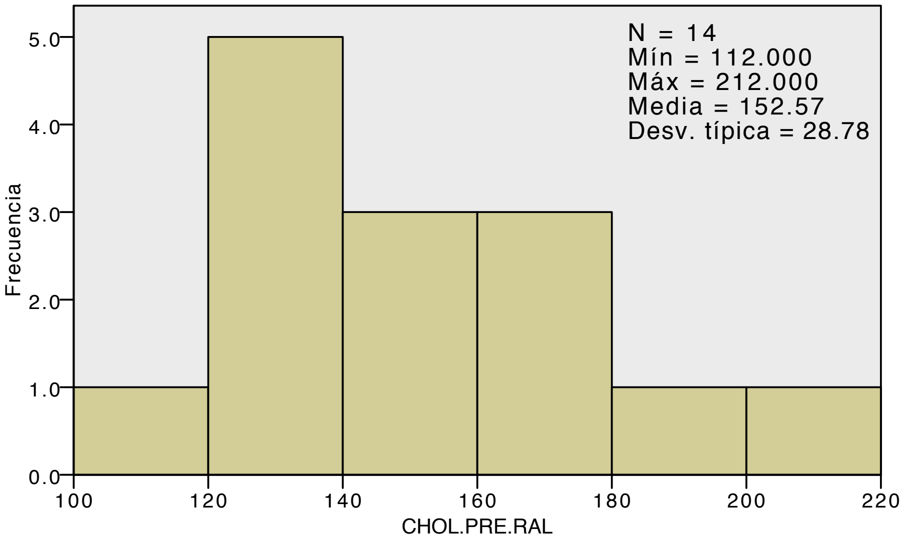


```
 *Nonparametric Tests: Related Samples.
NPTESTS
  /RELATED TEST(HDL.PRE.P HDL.PRE.RAL) WILCOXON
  /MISSING SCOPE=ANALYSIS USERMISSING=EXCLUDE
  /CRITERIA ALPHA=0.05  CILEVEL=95.
```

Pruebas no paramétricas

Notas

Resultados creados		23-MAR-2019 18:10:17
Comentarios		
Entrada	Datos	/Users/SergioBarroso/Desktop/T20-RAL Reviewers/Base de datos/RAL.sav
	Conjunto de datos activo	Conjunto_de_datos2
	Filtro	<ninguno>
	Peso	<ninguno>
	Dividir archivo	<ninguno>
	Núm. de filas del archivo de trabajo	14
Sintaxis		NPTESTS<br>/RELATED TEST(HDL.PRE.P HDL.PRE.RAL) WILCOXON<br>/MISSING SCOPE=ANALYSIS USERMISSING=EXCLUDE<br>/CRITERIA ALPHA=0.05 CILEVEL=95.
Recursos	Tiempo de procesador	00:00:00.10
	Tiempo transcurrido	00:00:00.00

```
[Conjunto_de_datos2] /Users/SergioBarroso/Desktop/T20-RAL Reviewers/Base de datos/RAL.sav
```

Resumen de prueba de hipótesis

	Hipótesis nula	Test	Sig.	Decisión
1	La mediana de las diferencias entre HDL.PRE.P y HDL.PRE.RAL es igual a 0.	Prueba de Wilcoxon de los rangos con signo de muestras relacionadas	.779	Retener la hipótesis nula.

Se muestran las significancias asintóticas. El nivel de significancia es .05.

Prueba de Wilcoxon de los rangos con signo de muestras relacionadas

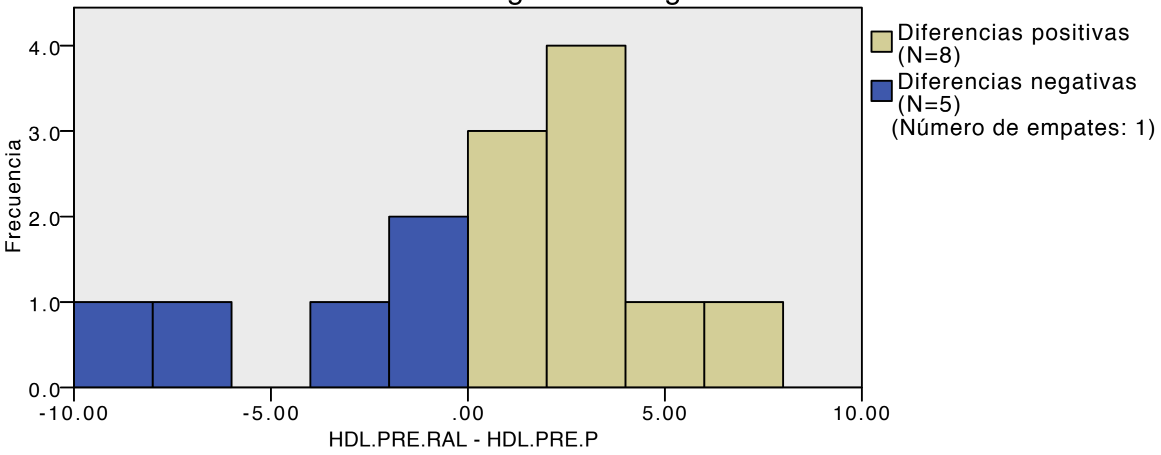

N total	14
Probar estadística	49.500
Error típico	14.265
Estadística de prueba estandarizada	.280
Sig. asintótica (prueba de dos caras)	.779

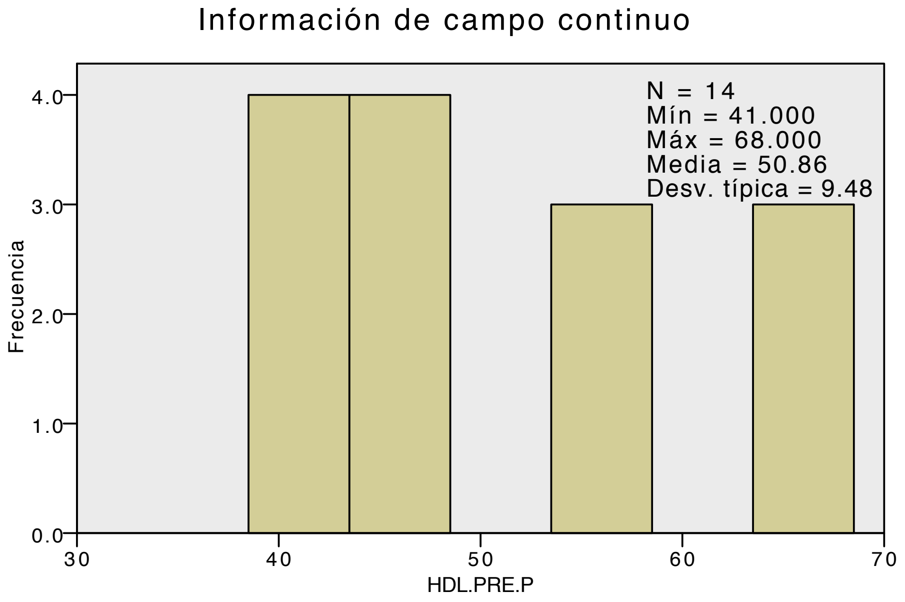

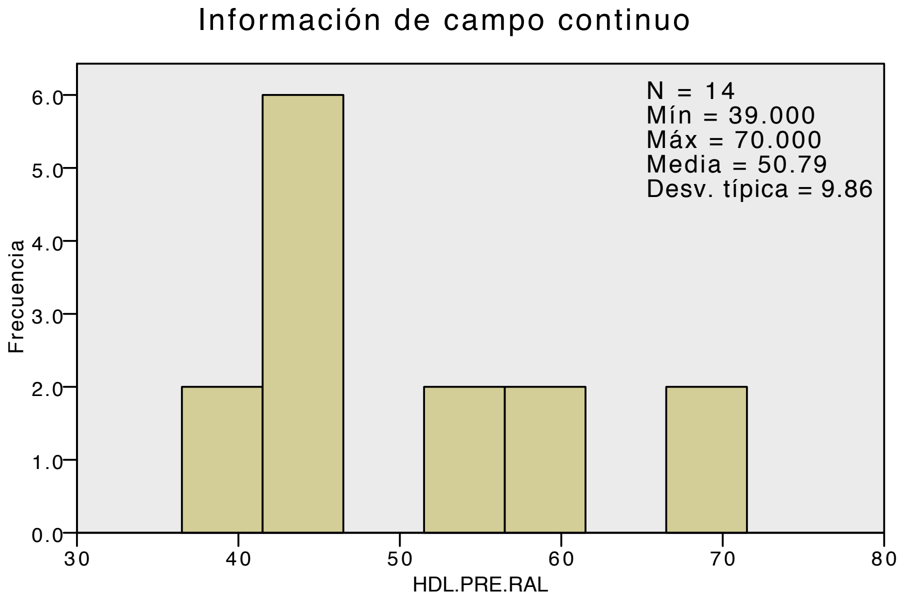


```
 *Nonparametric Tests: Related Samples.
NPTESTS
  /RELATED TEST(LDL.PRE.P LDL.PRE.RAL) WILCOXON
  /MISSING SCOPE=ANALYSIS USERMISSING=EXCLUDE
  /CRITERIA ALPHA=0.05  CILEVEL=95.
```

Pruebas no paramétricas

Notas

Resultados creados		23-MAR-2019 18:10:41
Comentarios		
Entrada	Datos	/Users/SergioBarroso/Desktop/T20-RAL Reviewers/Base de datos/RAL.sav
	Conjunto de datos activo	Conjunto_de_datos2
	Filtro	<ninguno>
	Peso	<ninguno>
	Dividir archivo	<ninguno>
	Núm. de filas del archivo de trabajo	14
Sintaxis		NPTESTS /RELATED TEST(LDL.PRE.P LDL.PRE.RAL) WILCOXON /MISSING SCOPE=ANALYSIS USERMISSING=EXCLUDE /CRITERIA ALPHA=0.05 CILEVEL=95.
Recursos	Tiempo de procesador	00:00:00.07
	Tiempo transcurrido	00:00:00.00

```
[Conjunto_de_datos2] /Users/SergioBarroso/Desktop/T20-RAL Reviewers/Base de datos/RAL.sav
```

Resumen de prueba de hipótesis

	Hipótesis nula	Test	Sig.	Decisión
1	La mediana de las diferencias entre LDL.PRE.P y LDL.PRE.RAL es igual a 0.	Prueba de Wilcoxon de los rangos con signo de muestras relacionadas	.220	Retener la hipótesis nula.

Se muestran las significancias asintóticas. El nivel de significancia es .05.

Prueba de Wilcoxon de los rangos con signo de muestras relacionadas

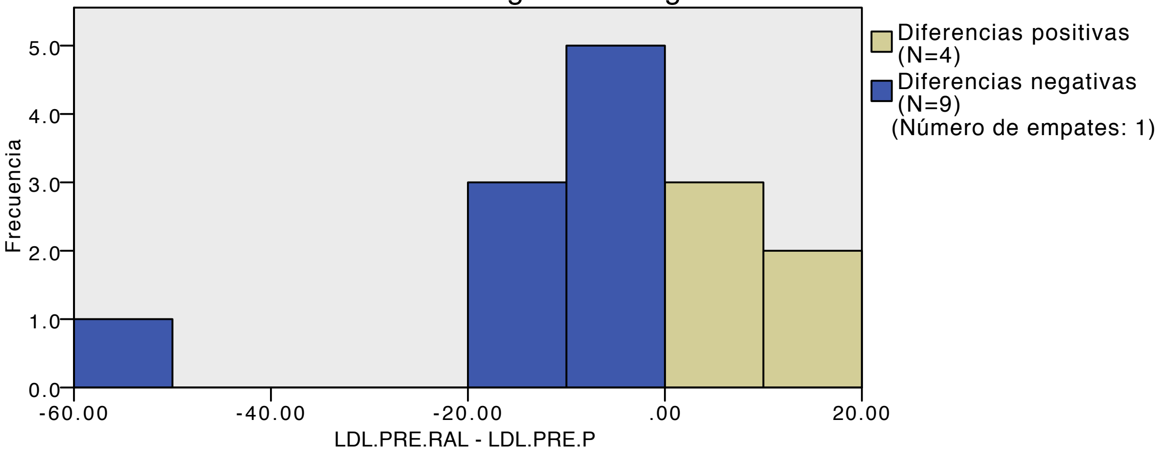

N total	14
Probar estadística	28.000
Error típico	14.283
Estadística de prueba estandarizada	-1.225
Sig. asintótica (prueba de dos caras)	.220

Información de campo continuo

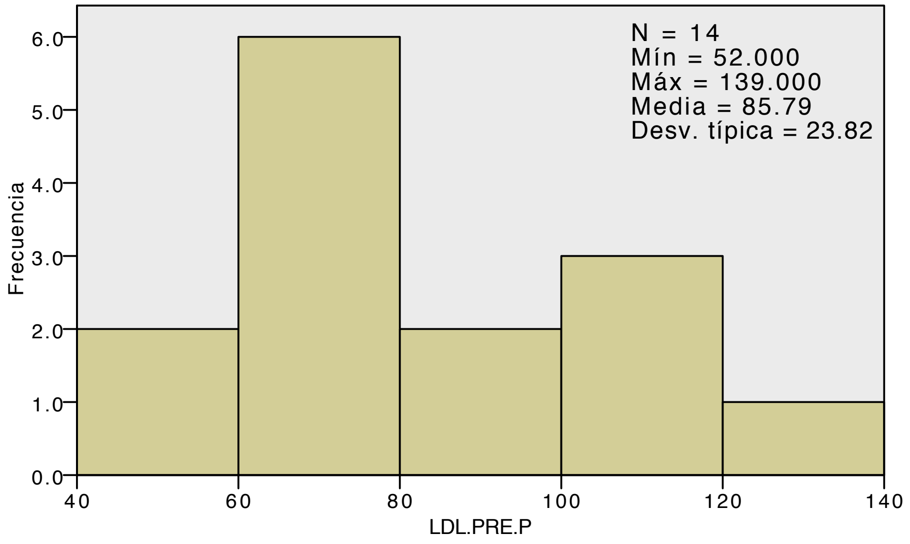

Información de campo continuo

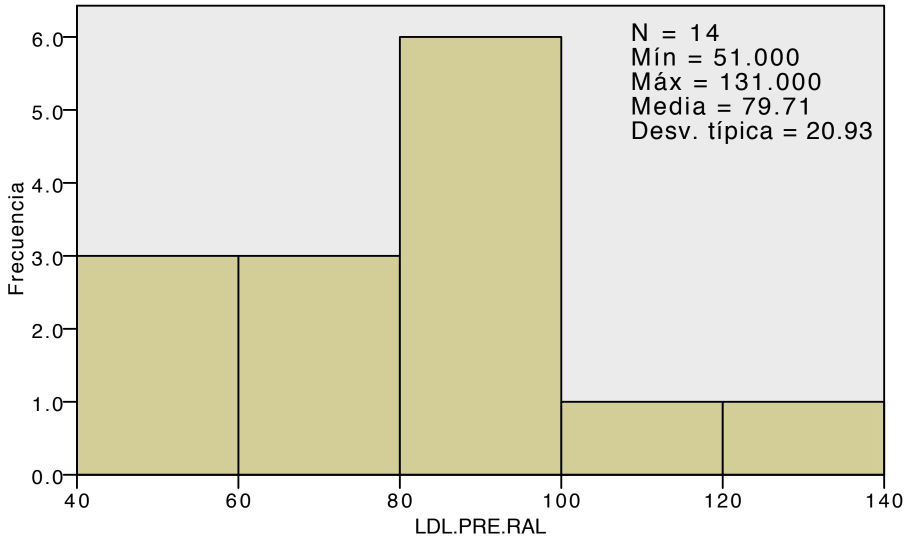


```
 *Nonparametric Tests: Related Samples.
NPTESTS
  /RELATED TEST(TAG.PRE.P TAG.PRE.RAL) WILCOXON
  /MISSING SCOPE=ANALYSIS USERMISSING=EXCLUDE
  /CRITERIA ALPHA=0.05  CILEVEL=95.
```

Pruebas no paramétricas

Notas

Resultados creados		23-MAR-2019 18:11:05
Comentarios		
Entrada	Datos	/Users/SergioBarroso/Desktop/T20-RAL Reviewers/Base de datos/RAL.sav
	Conjunto de datos activo	Conjunto_de_datos2
	Filtro	<ninguno>
	Peso	<ninguno>
	Dividir archivo	<ninguno>
	Núm. de filas del archivo de trabajo	14
Sintaxis		NPTESTS /RELATED TEST(TAG.PRE.P TAG.PRE.RAL) WILCOXON /MISSING SCOPE=ANALYSIS USERMISSING=EXCLUDE /CRITERIA ALPHA=0.05 CILEVEL=95.
Recursos	Tiempo de procesador	00:00:00.07
	Tiempo transcurrido	00:00:00.00

```
[Conjunto_de_datos2] /Users/SergioBarroso/Desktop/T20-RAL Reviewers/Base de datos/RAL.sav
```

Resumen de prueba de hipótesis

	Hipótesis nula	Test	Sig.	Decisión
1	La mediana de las diferencias entre TAG.PRE.P y TAG.PRE.RAL es igual a 0.	Prueba de Wilcoxon de los rangos con signo de muestras relacionadas	.510	Retener la hipótesis nula.

Se muestran las significancias asintóticas. El nivel de significancia es .05.

Prueba de Wilcoxon de los rangos con signo de muestras relacionadas

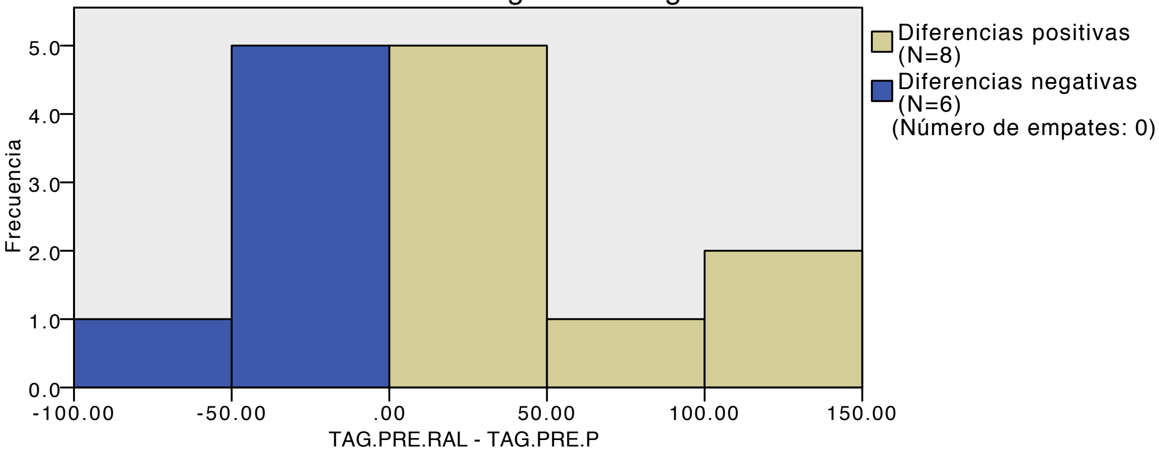

N total	14
Probar estadística	63.000
Error típico	15.930
Estadística de prueba estandarizada	.659
Sig. asintótica (prueba de dos caras)	.510

Información de campo continuo

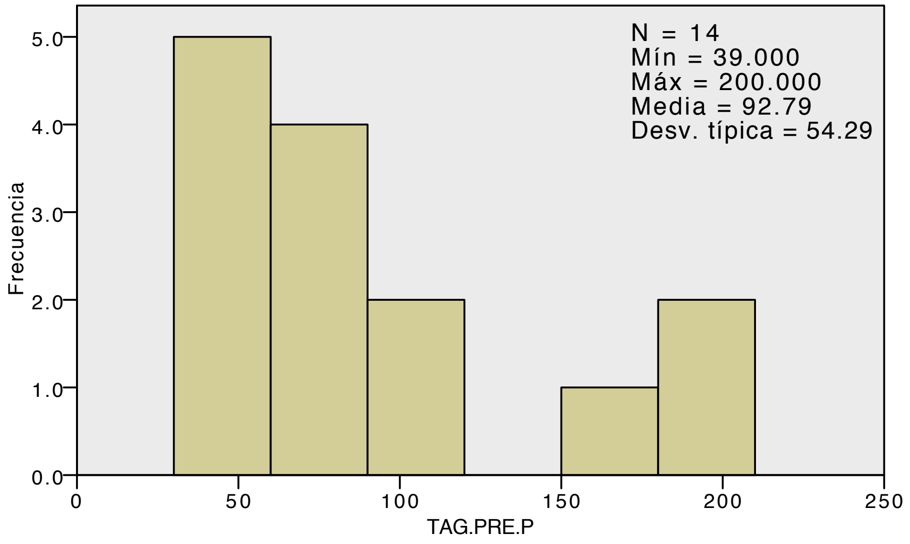

Información de campo continuo

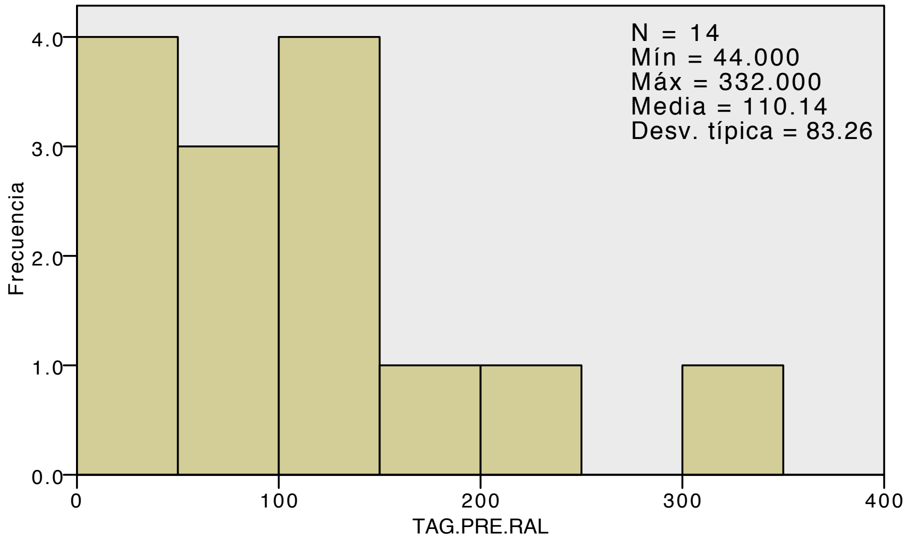


```
 *Nonparametric Tests: Related Samples.
NPTESTS
  /RELATED TEST(GLU.PRE.P GLU.PRE.RAL) WILCOXON
  /MISSING SCOPE=ANALYSIS USERMISSING=EXCLUDE
  /CRITERIA ALPHA=0.05  CILEVEL=95.
```

Pruebas no paramétricas

Notas

**Resultados creados**		**23-MAR-2019 18:11:35**
**Comentarios**		
**Entrada**	**Datos**	**/Users/SergioBarroso/Desktop/T20-RAL Reviewers/Base de datos/RAL.sav**
	**Conjunto de datos activo**	**Conjunto_de_datos2**
	**Filtro**	**<ninguno>**
	**Peso**	**<ninguno>**
	**Dividir archivo**	**<ninguno>**
	**Núm. de filas del archivo de trabajo**	**14**
**Sintaxis**		**NPTESTS /RELATED TEST(GLU.PRE.P GLU.PRE.RAL) WILCOXON /MISSING SCOPE=ANALYSIS USERMISSING=EXCLUDE /CRITERIA ALPHA=0.05 CILEVEL=95.**
**Recursos**	**Tiempo de procesador**	**00:00:00.08**
	**Tiempo transcurrido**	**00:00:00.00**

```
[Conjunto_de_datos2] /Users/SergioBarroso/Desktop/T20-RAL Reviewers/Base de datos/RAL.sav
```

Resumen de prueba de hipótesis

	Hipótesis nula	Test	Sig.	Decisión
1	La mediana de las diferencias entre GLU.PRE.P y GLU.PRE.RAL es igual a 0.	Prueba de Wilcoxon de los rangos con signo de muestras relacionadas	.972	Retener la hipótesis nula.

Se muestran las significancias asintóticas. El nivel de significancia es .05.

Prueba de Wilcoxon de los rangos con signo de muestras relacionadas

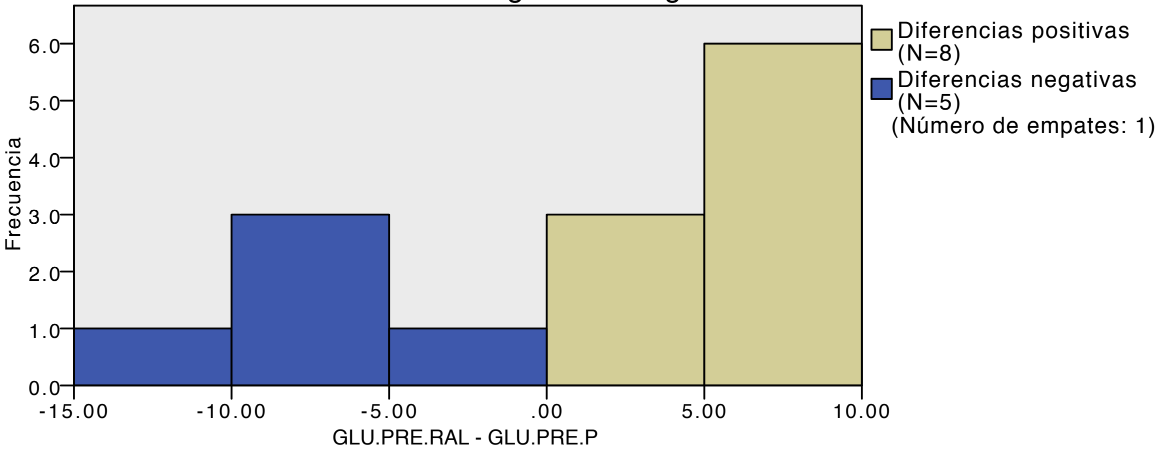

N total	14
Probar estadística	46.000
Error típico	14.257
Estadística de prueba estandarizada	.035
Sig. asintótica (prueba de dos caras)	.972

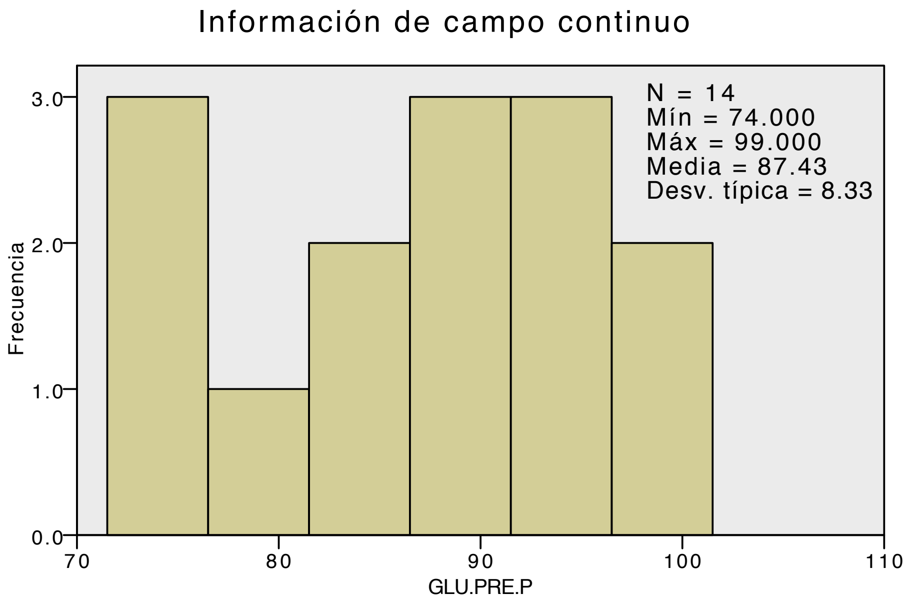

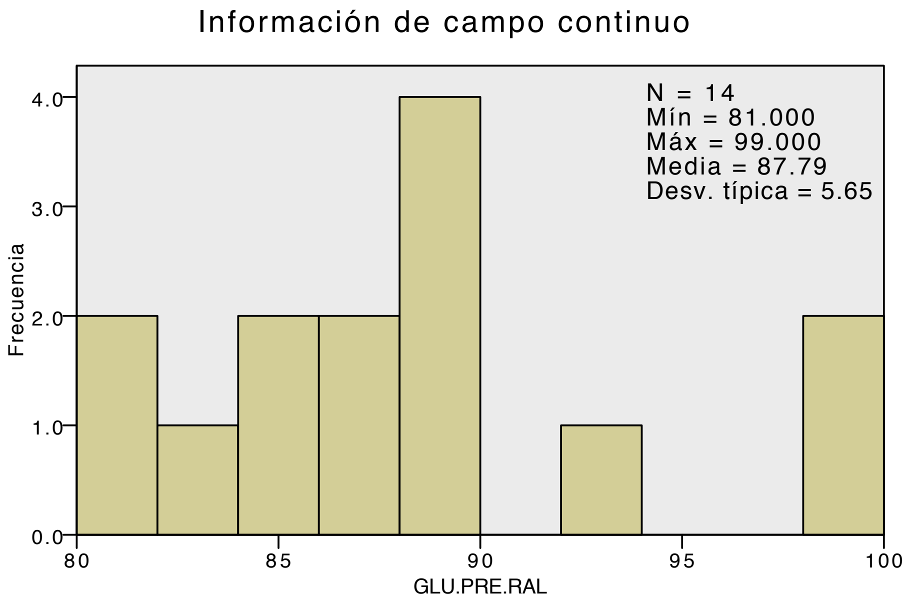


```
 *Nonparametric Tests: Related Samples.
NPTESTS
  /RELATED TEST(mtDNA.PRE.P mtDNA.PRE.RAL) WILCOXON
  /MISSING SCOPE=ANALYSIS USERMISSING=EXCLUDE
  /CRITERIA ALPHA=0.05  CILEVEL=95.
```

Pruebas no paramétricas

Notas

Resultados creados		23-MAR-2019 18:12:07
Comentarios		
Entrada	Datos	/Users/SergioBarroso/Desktop/T20-RAL Reviewers/Base de datos/RAL.sav
	Conjunto de datos activo	Conjunto_de_datos2
	Filtro	<ninguno>
	Peso	<ninguno>
	Dividir archivo	<ninguno>
	Núm. de filas del archivo de trabajo	14
Sintaxis		NPTESTS /RELATED TEST (mtDNA.PRE.P mtDNA.PRE.RAL) WILCOXON /MISSING SCOPE=ANALYSIS USERMISSING=EXCLUDE /CRITERIA ALPHA=0.05 CILEVEL=95.
Recursos	Tiempo de procesador	00:00:00.08
	Tiempo transcurrido	00:00:00.00

```
[Conjunto_de_datos2] /Users/SergioBarroso/Desktop/T20-RAL Reviewers/Base de datos/RAL.sav
```

Resumen de prueba de hipótesis

	Hipótesis nula	Test	Sig.	Decisión
1	La mediana de las diferencias entre mtDNA.PRE.P y mtDNA.PRE.RAL es igual a 0.	Prueba de Wilcoxon de los rangos con signo de muestras relacionadas	.975	Retener la hipótesis nula.

Se muestran las significancias asintóticas. El nivel de significancia es .05.

Prueba de Wilcoxon de los rangos con signo de muestras relacionadas

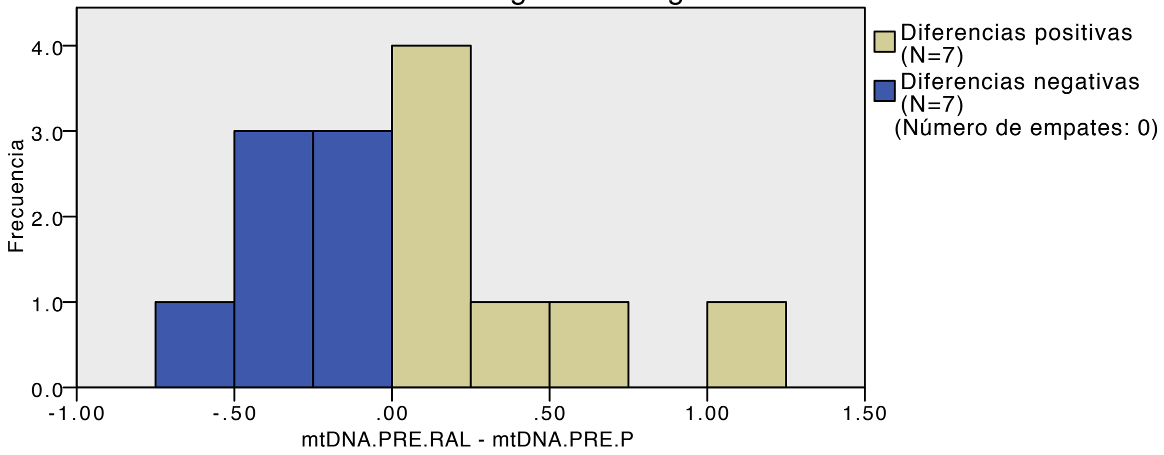

N total	14
Probar estadística	53.000
Error típico	15.930
Estadística de prueba estandarizada	.031
Sig. asintótica (prueba de dos caras)	.975

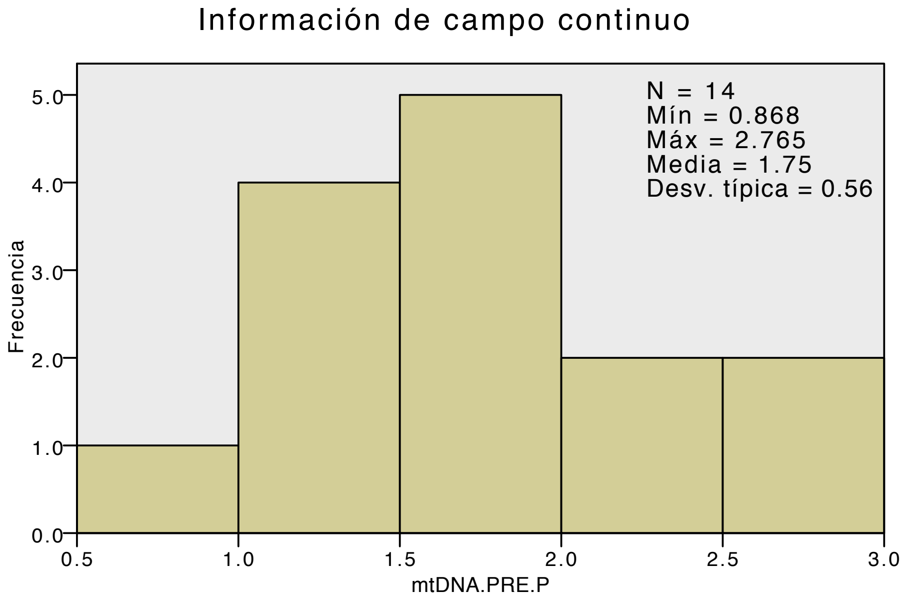

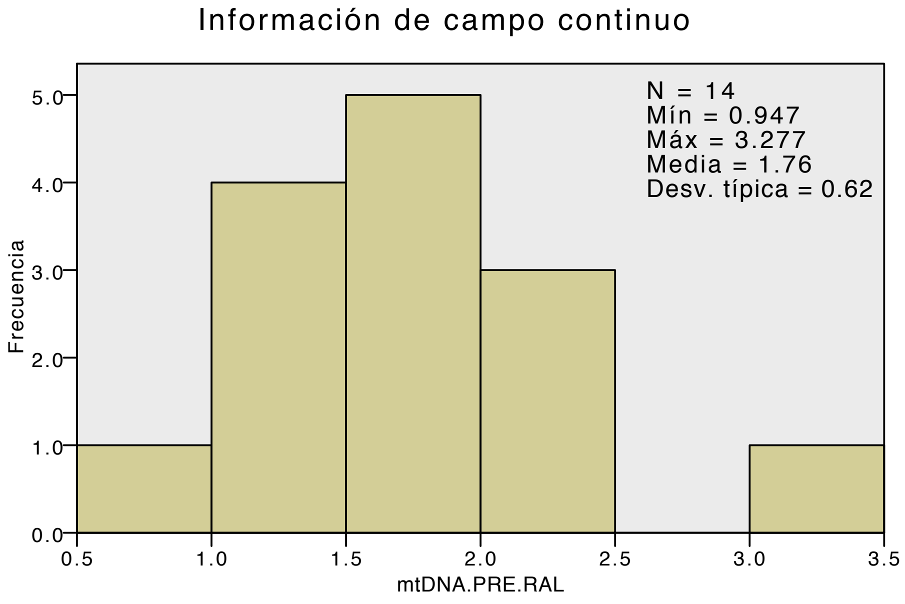


```
 *Nonparametric Tests: Related Samples.
NPTESTS
  /RELATED TEST(CRT.PRE.P CRT.PRE.RAL) WILCOXON
  /MISSING SCOPE=ANALYSIS USERMISSING=EXCLUDE
  /CRITERIA ALPHA=0.05  CILEVEL=95.
```

Pruebas no paramétricas

Notas

**Resultados creados**		**23-MAR-2019 18:12:25**
**Comentarios**		
**Entrada**	**Datos**	**/Users/SergioBarroso/Desktop/T20-RAL Reviewers/Base de datos/RAL.sav**
	**Conjunto de datos activo**	**Conjunto_de_datos2**
	**Filtro**	**<ninguno>**
	**Peso**	**<ninguno>**
	**Dividir archivo**	**<ninguno>**
	**Núm. de filas del archivo de trabajo**	**14**
**Sintaxis**		**NPTESTS /RELATED TEST(CRT.PRE.P CRT.PRE.RAL) WILCOXON /MISSING SCOPE=ANALYSIS USERMISSING=EXCLUDE /CRITERIA ALPHA=0.05 CILEVEL=95.**
**Recursos**	**Tiempo de procesador**	**00:00:00.07**
	**Tiempo transcurrido**	**00:00:00.00**

```
[Conjunto_de_datos2] /Users/SergioBarroso/Desktop/T20-RAL Reviewers/Base de datos/RAL.sav
```

Resumen de prueba de hipótesis

	Hipótesis nula	Test	Sig.	Decisión
1	La mediana de las diferencias entre CRT.PRE.P y CRT.PRE.RAL es igual a 0.	Prueba de Wilcoxon de los rangos con signo de muestras relacionadas	.925	Retener la hipótesis nula.

Se muestran las significancias asintóticas. El nivel de significancia es .05.

Prueba de Wilcoxon de los rangos con signo de muestras relacionadas

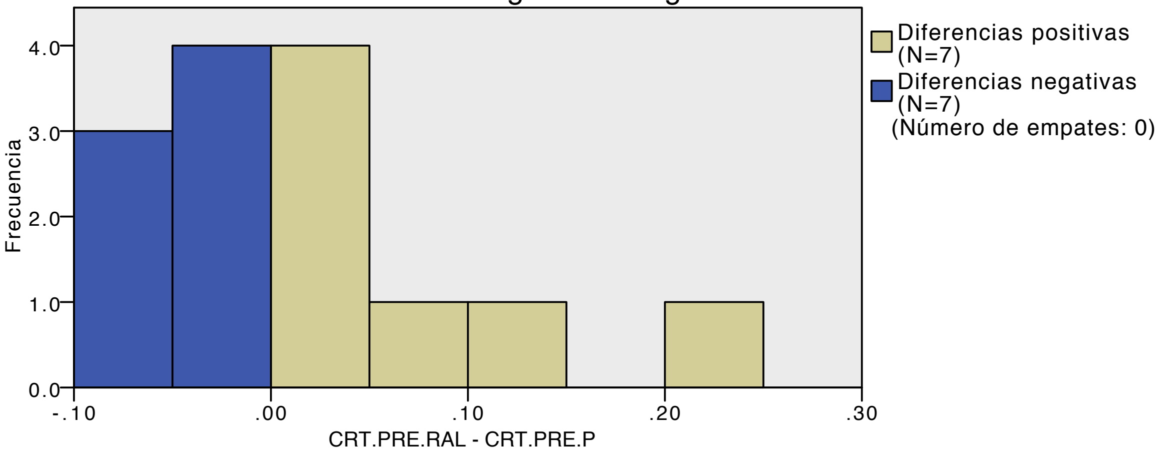

N total	14
Probar estadística	51.000
Error típico	15.882
Estadística de prueba estandarizada	-.094
Sig. asintótica (prueba de dos caras)	.925

Información de campo continuo

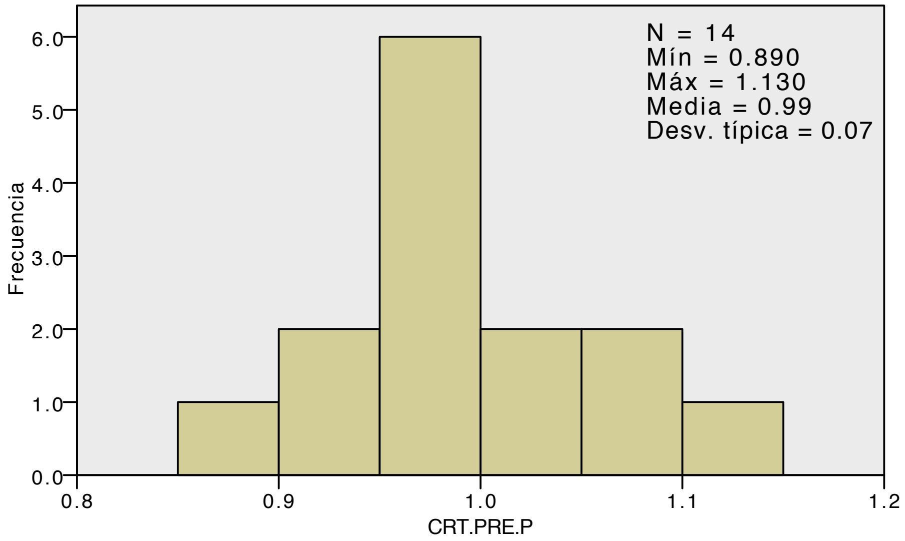

Información de campo continuo

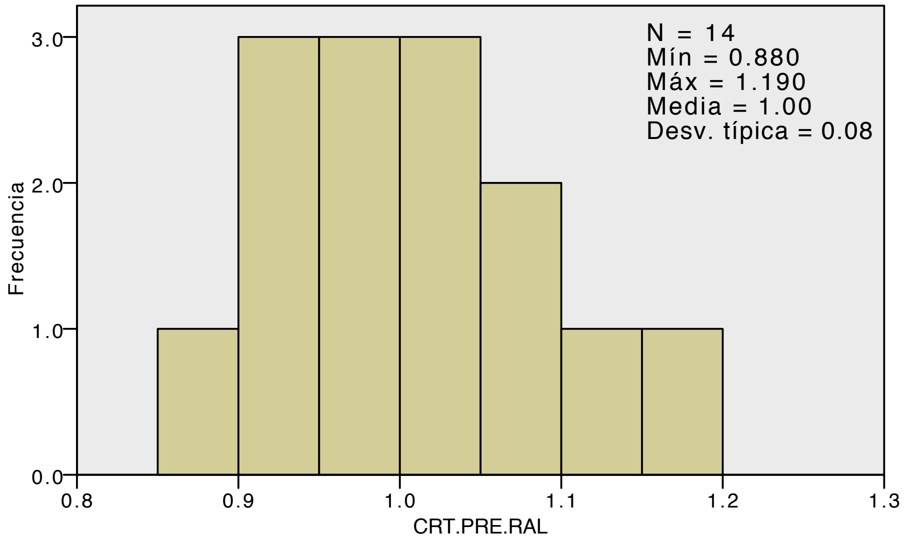


```
 *Nonparametric Tests: Related Samples.
NPTESTS
  /RELATED TEST(AST.PRE.P AST.PRE.RAL) WILCOXON
  /MISSING SCOPE=ANALYSIS USERMISSING=EXCLUDE
  /CRITERIA ALPHA=0.05  CILEVEL=95.
```

Pruebas no paramétricas

Notas

**Resultados creados**		**23-MAR-2019 18:12:52**
**Comentarios**		
**Entrada**	**Datos**	**/Users/SergioBarroso/Desktop/T20-RAL Reviewers/Base de datos/RAL.sav**
	**Conjunto de datos activo**	**Conjunto_de_datos2**
	**Filtro**	**<ninguno>**
	**Peso**	**<ninguno>**
	**Dividir archivo**	**<ninguno>**
	**Núm. de filas del archivo de trabajo**	**14**
**Sintaxis**		**NPTESTS /RELATED TEST(AST.PRE.P AST.PRE.RAL) WILCOXON /MISSING SCOPE=ANALYSIS USERMISSING=EXCLUDE /CRITERIA ALPHA=0.05 CILEVEL=95.**
**Recursos**	**Tiempo de procesador**	**00:00:00.13**
	**Tiempo transcurrido**	**00:00:00.00**

```
[Conjunto_de_datos2] /Users/SergioBarroso/Desktop/T20-RAL Reviewers/Base de datos/RAL.sav
```

Resumen de prueba de hipótesis

	Hipótesis nula	Test	Sig.	Decisión
1	La mediana de las diferencias entre AST.PRE.P y AST.PRE.RAL es igual a 0.	Prueba de Wilcoxon de los rangos con signo de muestras relacionadas	.659	Retener la hipótesis nula.

Se muestran las significancias asintóticas. El nivel de significancia es .05.

Prueba de Wilcoxon de los rangos con signo de muestras relacionadas

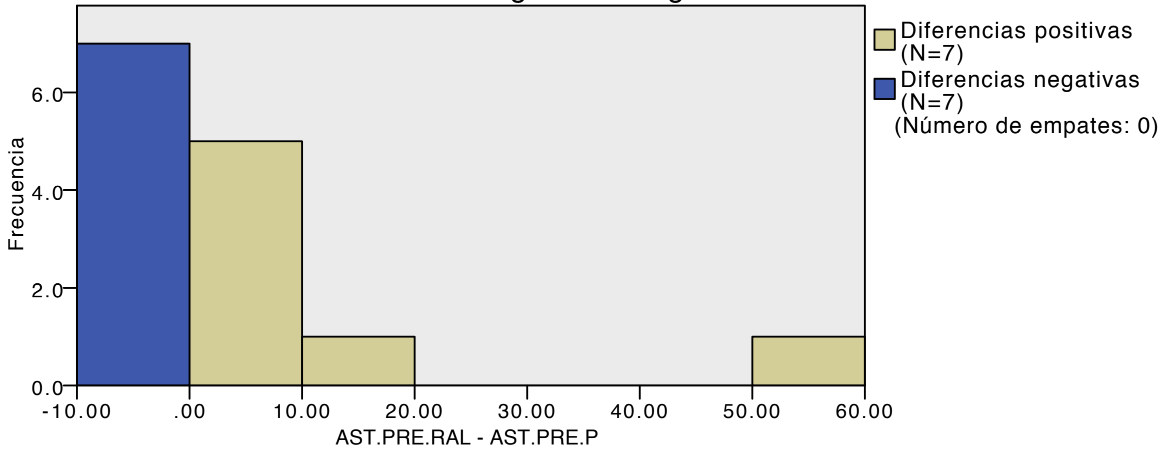

N total	14
Probar estadística	59.500
Error típico	15.847
Estadística de prueba estandarizada	.442
Sig. asintótica (prueba de dos caras)	.659

Información de campo continuo

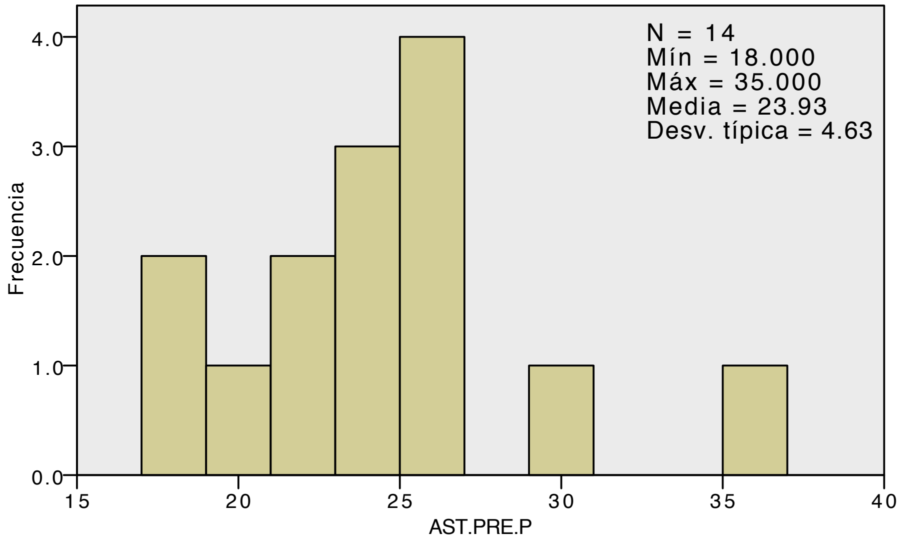

Información de campo continuo

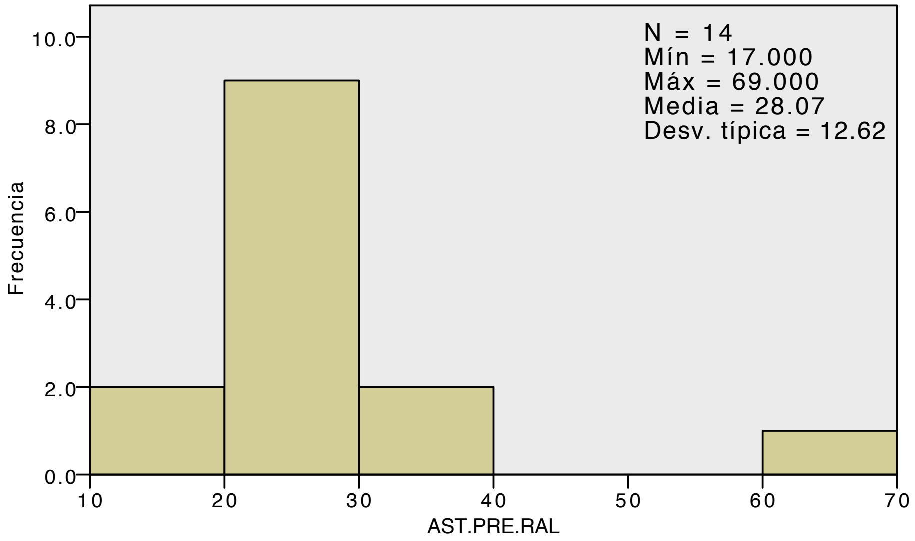


```
 *Nonparametric Tests: Related Samples.
NPTESTS
  /RELATED TEST(ALT.PRE.P ALT.PRE.RAL) WILCOXON
  /MISSING SCOPE=ANALYSIS USERMISSING=EXCLUDE
  /CRITERIA ALPHA=0.05  CILEVEL=95.
```

Pruebas no paramétricas

Notas

**Resultados creados**		**23-MAR-2019 18:13:16**
**Comentarios**		
**Entrada**	**Datos**	**/Users/SergioBarroso/Desktop/T20-RAL Reviewers/Base de datos/RAL.sav**
	**Conjunto de datos activo**	**Conjunto_de_datos2**
	**Filtro**	**<ninguno>**
	**Peso**	**<ninguno>**
	**Dividir archivo**	**<ninguno>**
	**Núm. de filas del archivo de trabajo**	**14**
**Sintaxis**		**NPTESTS /RELATED TEST(ALT.PRE.P ALT.PRE.RAL) WILCOXON /MISSING SCOPE=ANALYSIS USERMISSING=EXCLUDE /CRITERIA ALPHA=0.05 CILEVEL=95.**
**Recursos**	**Tiempo de procesador**	**00:00:00.09**
	**Tiempo transcurrido**	**00:00:00.00**

```
[Conjunto_de_datos2] /Users/SergioBarroso/Desktop/T20-RAL Reviewers/Base de datos/RAL.sav
```

Resumen de prueba de hipótesis

	Hipótesis nula	Test	Sig.	Decisión
1	La mediana de las diferencias entre ALT.PRE.P y ALT.PRE.RAL es igual a 0.	Prueba de Wilcoxon de los rangos con signo de muestras relacionadas	.562	Retener la hipótesis nula.

Se muestran las significancias asintóticas. El nivel de significancia es .05.

Prueba de Wilcoxon de los rangos con signo de muestras relacionadas

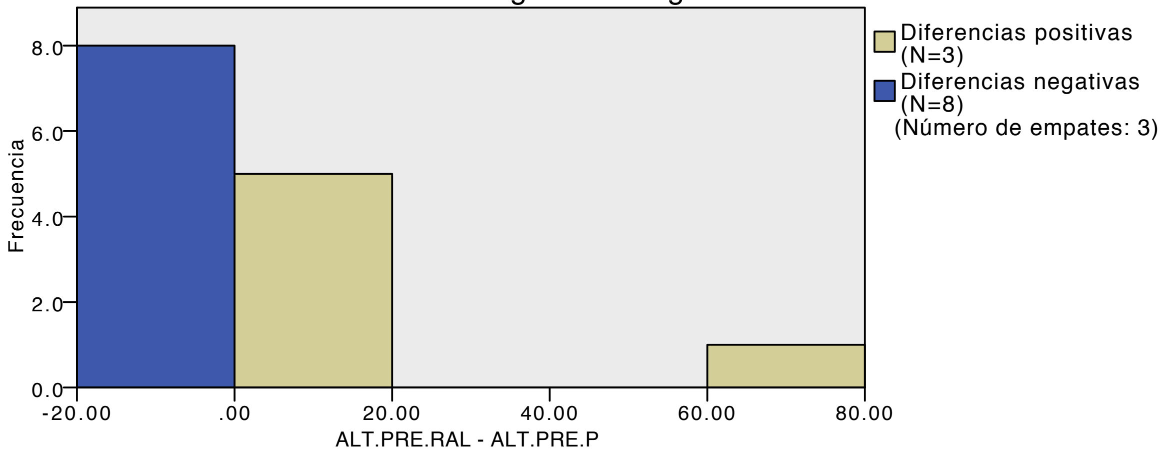

N total	14
Probar estadística	26.500
Error típico	11.214
Estadística de prueba estandarizada	-.580
Sig. asintótica (prueba de dos caras)	.562

Información de campo continuo

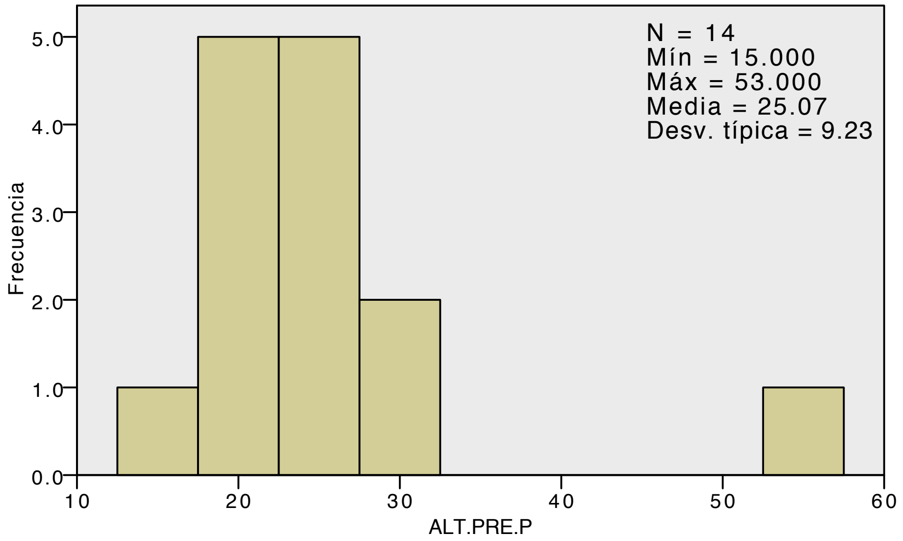

Información de campo continuo

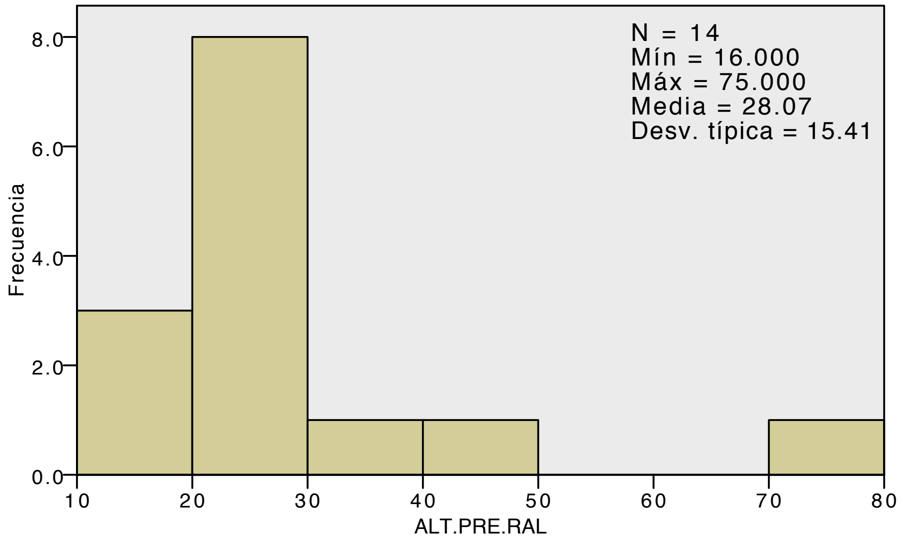


```
 *Nonparametric Tests: Related Samples.
NPTESTS
  /RELATED TEST(BIL.PRE.P BIL.PRE.RAL) WILCOXON
  /MISSING SCOPE=ANALYSIS USERMISSING=EXCLUDE
  /CRITERIA ALPHA=0.05  CILEVEL=95.
```

Pruebas no paramétricas

Notas

**Resultados creados**		**23-MAR-2019 18:13:48**
**Comentarios**		
**Entrada**	**Datos**	**/Users/SergioBarroso/Desktop/T20-RAL Reviewers/Base de datos/RAL.sav**
	**Conjunto de datos activo**	**Conjunto_de_datos2**
	**Filtro**	**<ninguno>**
	**Peso**	**<ninguno>**
	**Dividir archivo**	**<ninguno>**
	**Núm. de filas del archivo de trabajo**	**14**
**Sintaxis**		**NPTESTS /RELATED TEST(BIL.PRE.P BIL.PRE.RAL) WILCOXON /MISSING SCOPE=ANALYSIS USERMISSING=EXCLUDE /CRITERIA ALPHA=0.05 CILEVEL=95.**
**Recursos**	**Tiempo de procesador**	**00:00:00.06**
	**Tiempo transcurrido**	**00:00:00.00**

```
[Conjunto_de_datos2] /Users/SergioBarroso/Desktop/T20-RAL Reviewers/Base de datos/RAL.sav
```

Resumen de prueba de hipótesis

	Hipótesis nula	Test	Sig.	Decisión
1	La mediana de las diferencias entre BIL.PRE.P y BIL.PRE.RAL es igual a 0.	Prueba de Wilcoxon de los rangos con signo de muestras relacionadas	.549	Retener la hipótesis nula.

Se muestran las significancias asintóticas. El nivel de significancia es .05.

Prueba de Wilcoxon de los rangos con signo de muestras relacionadas

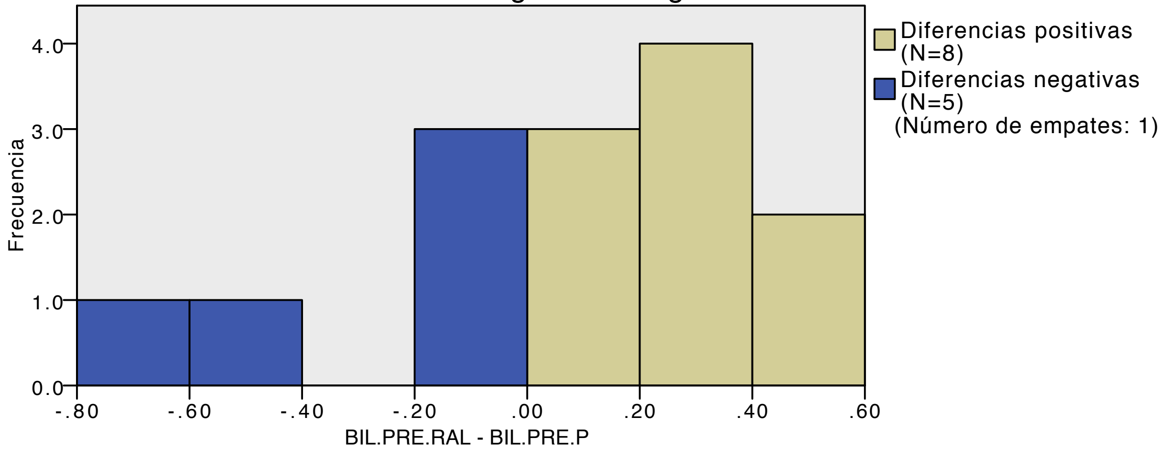

N total	14
Probar estadística	54.000
Error típico	14.173
Estadística de prueba estandarizada	.600
Sig. asintótica (prueba de dos caras)	.549

Información de campo continuo

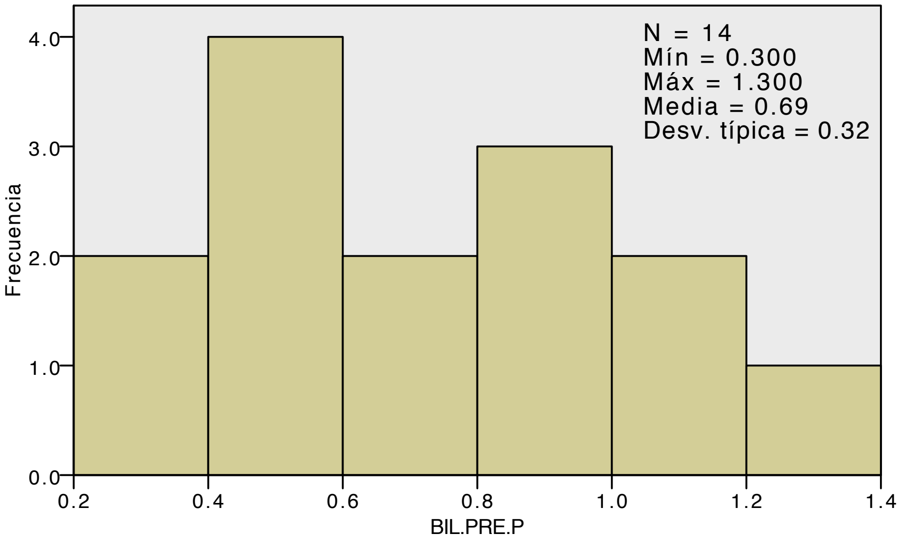

Información de campo continuo

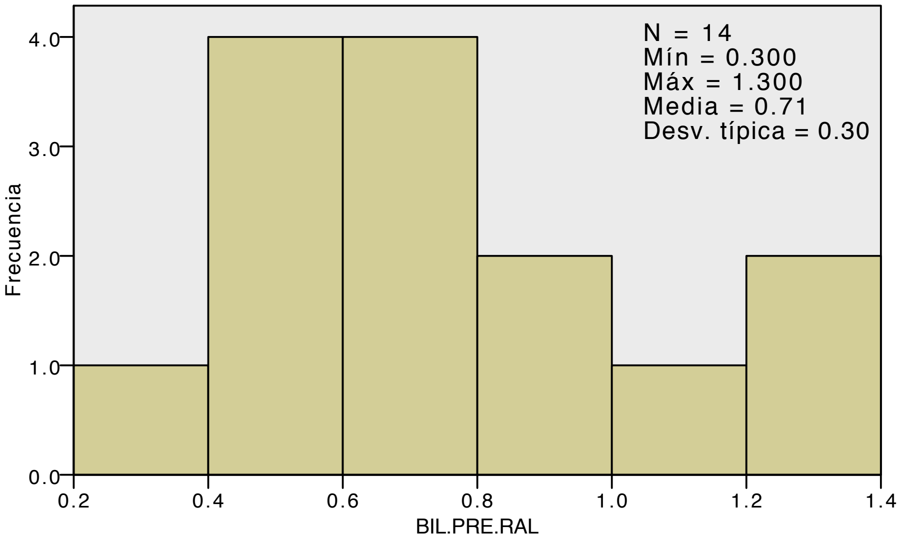


```
 *Nonparametric Tests: Related Samples.
NPTESTS
  /RELATED TEST(PROT.PRE.P PRO.PRE.RAL) WILCOXON
  /MISSING SCOPE=ANALYSIS USERMISSING=EXCLUDE
  /CRITERIA ALPHA=0.05  CILEVEL=95.
```

Pruebas no paramétricas

Notas

Resultados creados		23-MAR-2019 18:14:30
Comentarios		
Entrada	Datos	/Users/SergioBarroso/Desktop/T20-RAL Reviewers/Base de datos/RAL.sav
	Conjunto de datos activo	Conjunto_de_datos2
	Filtro	<ninguno>
	Peso	<ninguno>
	Dividir archivo	<ninguno>
	Núm. de filas del archivo de trabajo	14
Sintaxis		NPTESTS /RELATED TEST(PROT.PRE.P PRO.PRE.RAL) WILCOXON /MISSING SCOPE=ANALYSIS USERMISSING=EXCLUDE /CRITERIA ALPHA=0.05 CILEVEL=95.
Recursos	Tiempo de procesador	00:00:00.13
	Tiempo transcurrido	00:00:00.00

```
[Conjunto_de_datos2] /Users/SergioBarroso/Desktop/T20-RAL Reviewers/Base de datos/RAL.sav
```

Resumen de prueba de hipótesis

	Hipótesis nula	Test	Sig.	Decisión
1	La mediana de las diferencias entre PROT.PRE.P y PRO.PRE.RAL es igual a 0.	Prueba de Wilcoxon de los rangos con signo de muestras relacionadas	.170	Retener la hipótesis nula.

Se muestran las significancias asintóticas. El nivel de significancia es .05.

Prueba de Wilcoxon de los rangos con signo de muestras relacionadas

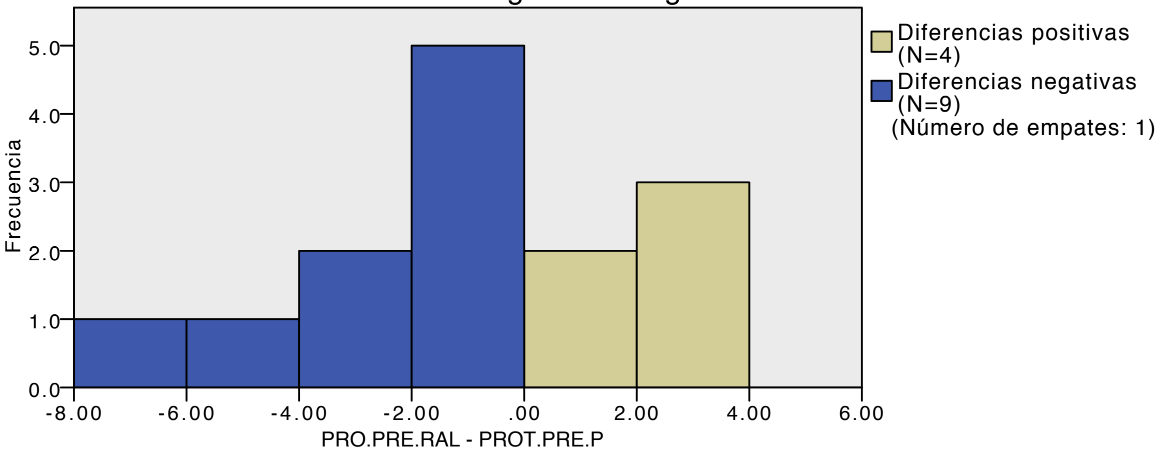

N total	14
Probar estadística	26.000
Error típico	14.199
Estadística de prueba estandarizada	-1.373
Sig. asintótica (prueba de dos caras)	.170

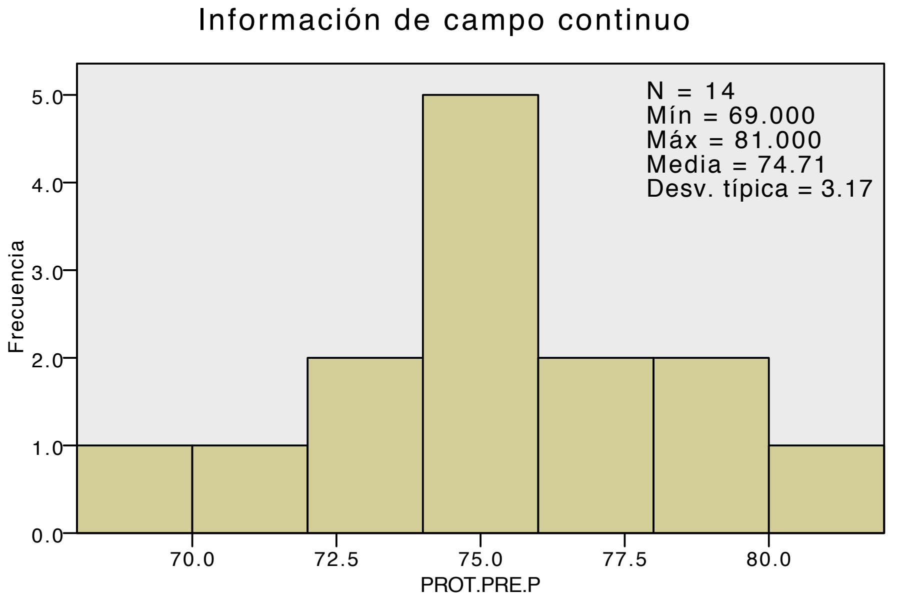
Información de campo continuo
N = 14
Mín = 69.000
Máx = 81.000
Media = 74.71
Desv. típica = 3.17
Frecuencia
5.0
4.0
3.0
2.0
1.0
0.0
70.0
72.5
75.0
77.5
80.0
PROT.PRE.P

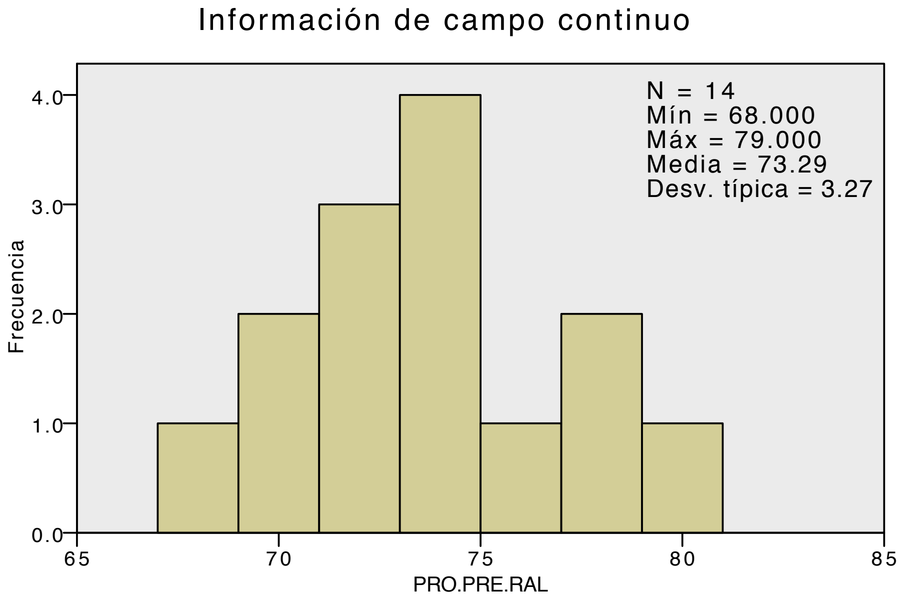
Información de campo continuo
N = 14
Mín = 68.000
Máx = 79.000
Media = 73.29
Desv. típica = 3.27
Frecuencia
4.0
3.0
2.0
1.0
0.0
65
70
75
80
85
PRO.PRE.RAL

```
 *Nonparametric Tests: Related Samples.
NPTESTS
  /RELATED TEST(CHOL.DIF.P CHOL.DIF.T20) WILCOXON
  /MISSING SCOPE=ANALYSIS USERMISSING=EXCLUDE
  /CRITERIA ALPHA=0.05  CILEVEL=95.
```

Pruebas no paramétricas

Notas

Resultados creados		23-MAR-2019 18:41:17
Comentarios		
Entrada	Datos	/Users/SergioBarroso/Desktop/T20-RAL Reviewers/Base de datos/RAL.sav
	Conjunto de datos activo	Conjunto_de_datos2
	Filtro	<ninguno>
	Peso	<ninguno>
	Dividir archivo	<ninguno>
	Núm. de filas del archivo de trabajo	14
Sintaxis		NPTESTS /RELATED TEST(CHOL.DIF.P CHOL.DIF.T20) WILCOXON /MISSING SCOPE=ANALYSIS USERMISSING=EXCLUDE /CRITERIA ALPHA=0.05 CILEVEL=95.
Recursos	Tiempo de procesador	00:00:00.06
	Tiempo transcurrido	00:00:00.00

```
[Conjunto_de_datos2] /Users/SergioBarroso/Desktop/T20-RAL Reviewers/Base de datos/RAL.sav
```

Resumen de prueba de hipótesis

	Hipótesis nula	Test	Sig.	Decisión
1	La mediana de las diferencias entre CHOL POST-PRE Placebo y CHOL POST-PRE T20 es igual a 0.	Prueba de Wilcoxon de los rangos con signo de muestras relacionadas	.414	Retener la hipótesis nula.

Se muestran las significancias asintóticas. El nivel de significancia es .05.

Prueba de Wilcoxon de los rangos con signo de muestras relacionadas

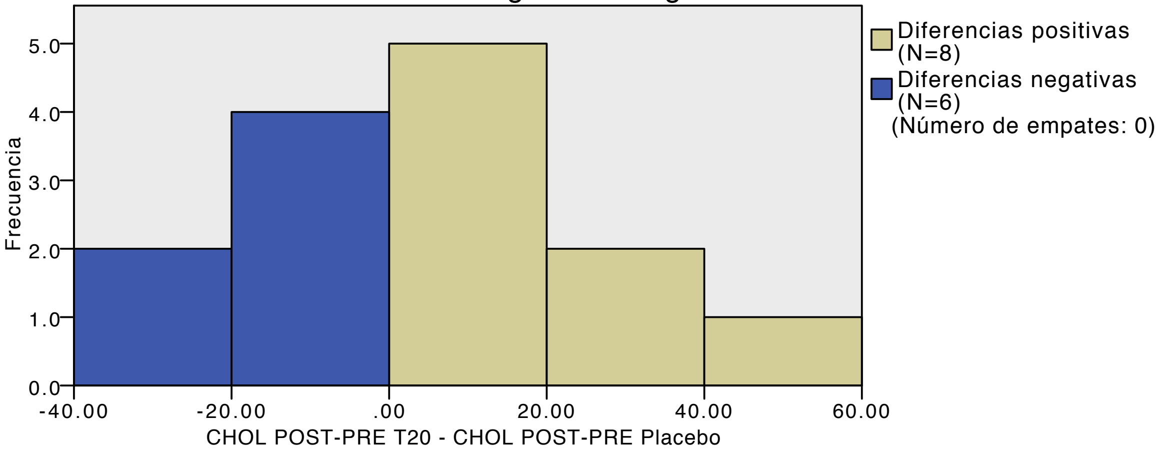

N total	14
Probar estadística	65.500
Error típico	15.910
Estadística de prueba estandarizada	.817
Sig. asintótica (prueba de dos caras)	.414

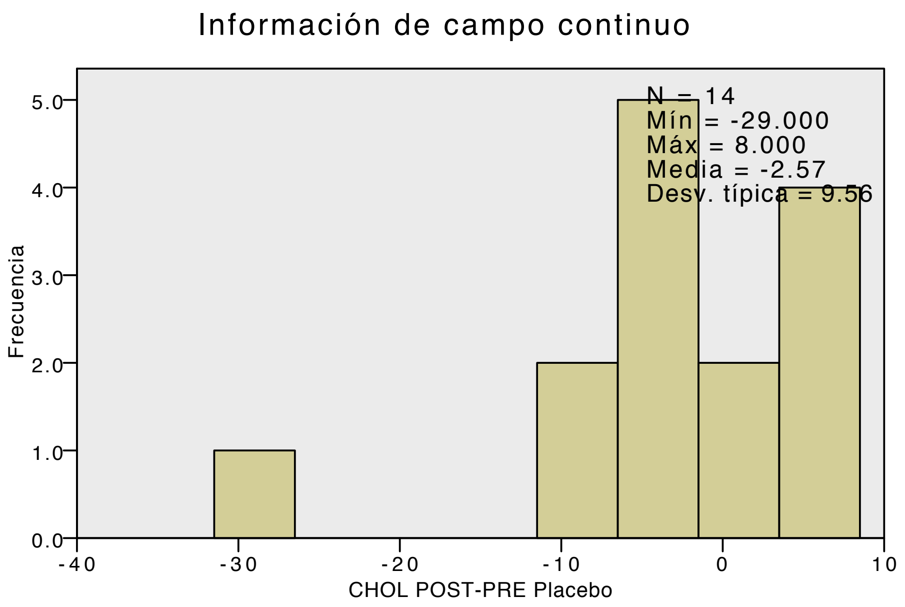

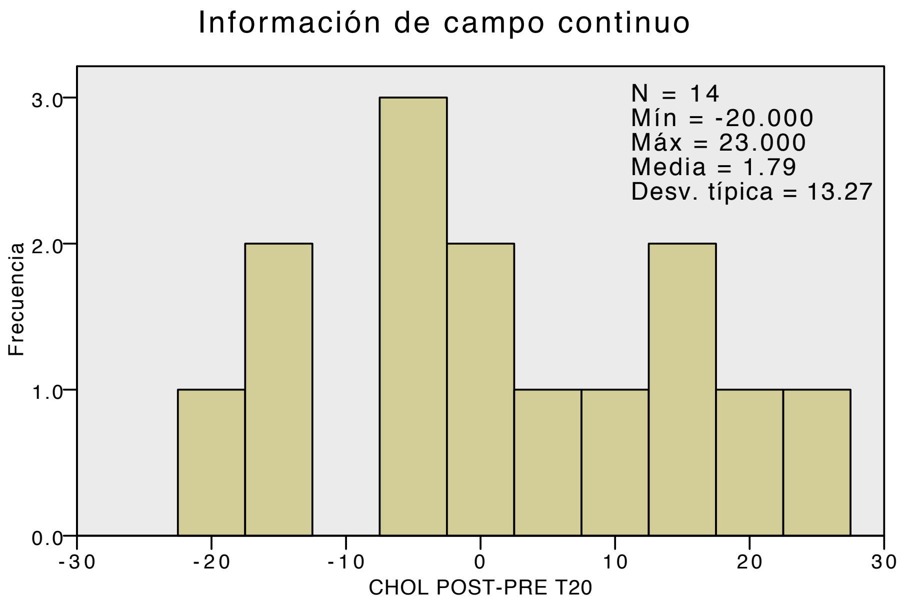


```
 *Nonparametric Tests: Related Samples.
NPTESTS
  /RELATED TEST(HDL.DIF.P HDL.DIF.T20) WILCOXON
  /MISSING SCOPE=ANALYSIS USERMISSING=EXCLUDE
  /CRITERIA ALPHA=0.05  CILEVEL=95.
```

Pruebas no paramétricas

Notas

Resultados creados		23-MAR-2019 18:41:35
Comentarios		
Entrada	Datos	/Users/SergioBarroso/Desktop/T20-RAL Reviewers/Base de datos/RAL.sav
	Conjunto de datos activo	Conjunto_de_datos2
	Filtro	<ninguno>
	Peso	<ninguno>
	Dividir archivo	<ninguno>
	Núm. de filas del archivo de trabajo	14
Sintaxis		NPTESTS /RELATED TEST(HDL.DIF.P HDL.DIF.T20) WILCOXON /MISSING SCOPE=ANALYSIS USERMISSING=EXCLUDE /CRITERIA ALPHA=0.05 CILEVEL=95.
Recursos	Tiempo de procesador	00:00:00.15
	Tiempo transcurrido	00:00:00.00

[Conjunto_de_datos2] /Users/SergioBarroso/Desktop/T20-RAL Reviewers/Base de datos/RAL.sav

Resumen de prueba de hipótesis

	Hipótesis nula	Test	Sig.	Decisión
1	La mediana de las diferencias entre HDL POST-PRE Placebo y HDL POST-PRE T20 es igual a 0.	Prueba de Wilcoxon de los rangos con signo de muestras relacionadas	.472	Retener la hipótesis nula.

Se muestran las significancias asintóticas. El nivel de significancia es .05.

Prueba de Wilcoxon de los rangos con signo de muestras relacionadas

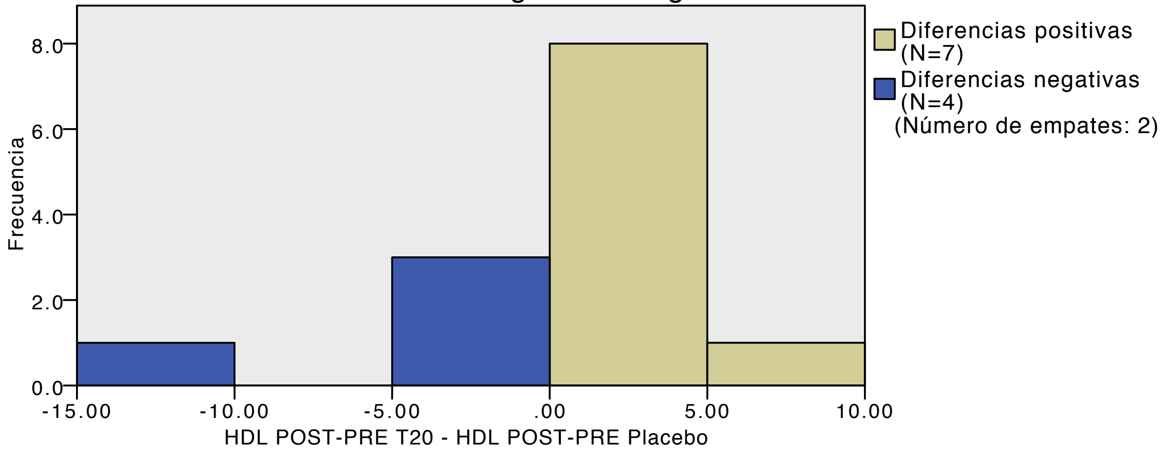

N total	13
Probar estadística	41.000
Error típico	11.113
Estadística de prueba estandarizada	.720
Sig. asintótica (prueba de dos caras)	.472

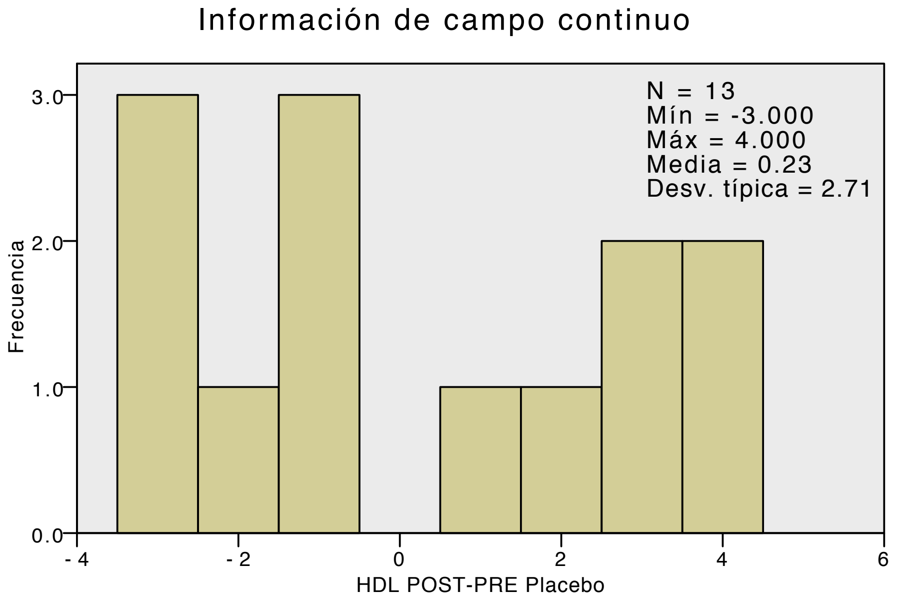

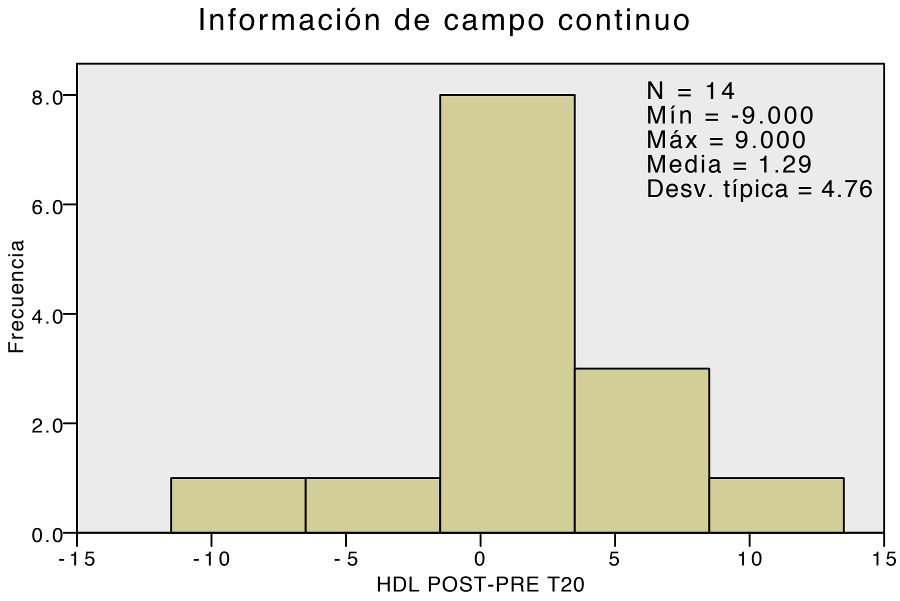


```
 *Nonparametric Tests: Related Samples.
NPTESTS
  /RELATED TEST(LDL.DIF.P LDL.DIF.T20) WILCOXON
  /MISSING SCOPE=ANALYSIS USERMISSING=EXCLUDE
  /CRITERIA ALPHA=0.05  CILEVEL=95.
```

Pruebas no paramétricas

Notas

**Resultados creados**		**23-MAR-2019 18:42:11**
**Comentarios**		
**Entrada**	**Datos**	**/Users/SergioBarroso/Desktop/T20-RAL Reviewers/Base de datos/RAL.sav**
	**Conjunto de datos activo**	**Conjunto_de_datos2**
	**Filtro**	**<ninguno>**
	**Peso**	**<ninguno>**
	**Dividir archivo**	**<ninguno>**
	**Núm. de filas del archivo de trabajo**	**14**
**Sintaxis**		**NPTESTS /RELATED TEST(LDL.DIF.P LDL.DIF.T20) WILCOXON /MISSING SCOPE=ANALYSIS USERMISSING=EXCLUDE /CRITERIA ALPHA=0.05 CILEVEL=95.**
**Recursos**	**Tiempo de procesador**	**00:00:00.19**
	**Tiempo transcurrido**	**00:00:00.00**

```
[Conjunto_de_datos2] /Users/SergioBarroso/Desktop/T20-RAL Reviewers/Base de datos/RAL.sav
```

Resumen de prueba de hipótesis

	Hipótesis nula	Test	Sig.	Decisión
1	La mediana de las diferencias entre LDL POST-PRE Placebo y LDL POST-PRE T20 es igual a 0.	Prueba de Wilcoxon de los rangos con signo de muestras relacionadas	.223	Retener la hipótesis nula.

Se muestran las significancias asintóticas. El nivel de significancia es .05.

Prueba de Wilcoxon de los rangos con signo de muestras relacionadas

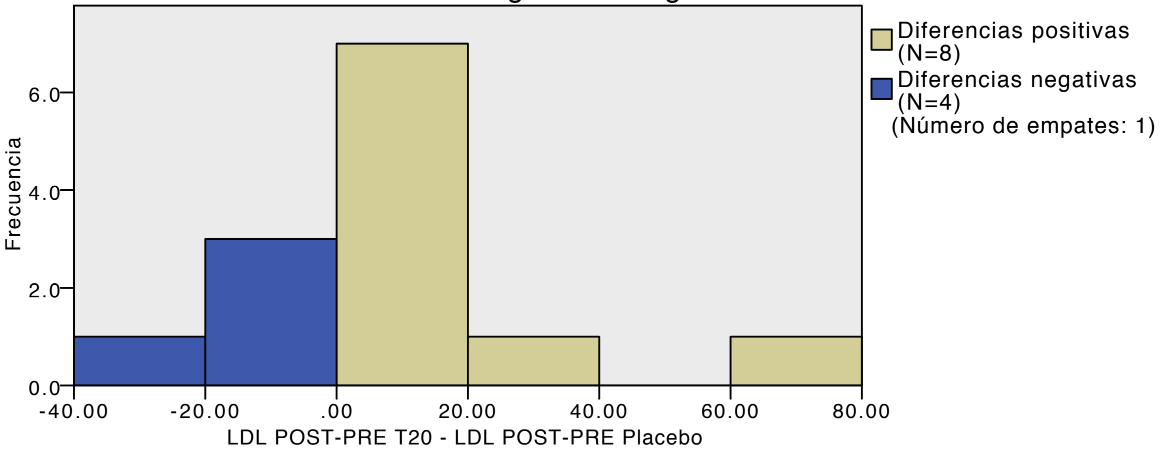

N total	13
Probar estadística	54.500
Error típico	12.733
Estadística de prueba estandarizada	1.217
Sig. asintótica (prueba de dos caras)	.223

Información de campo continuo

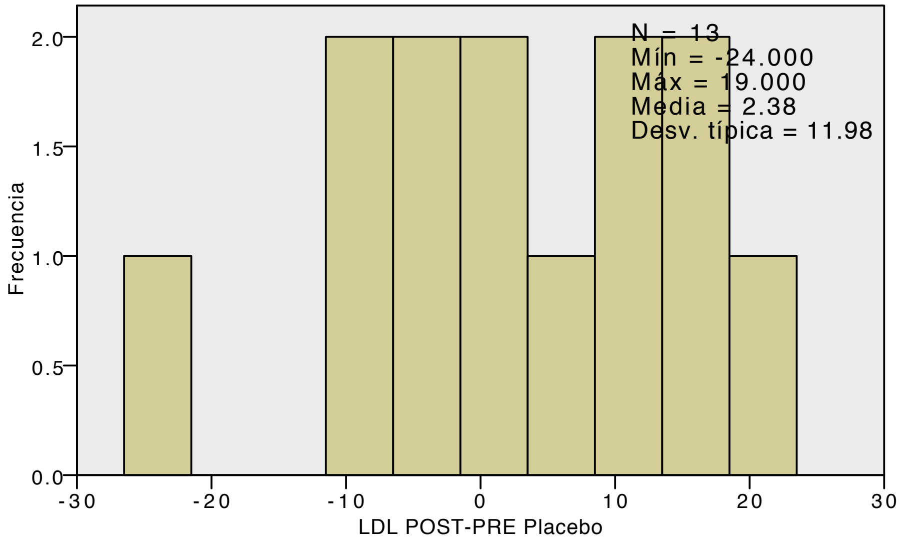

Información de campo continuo

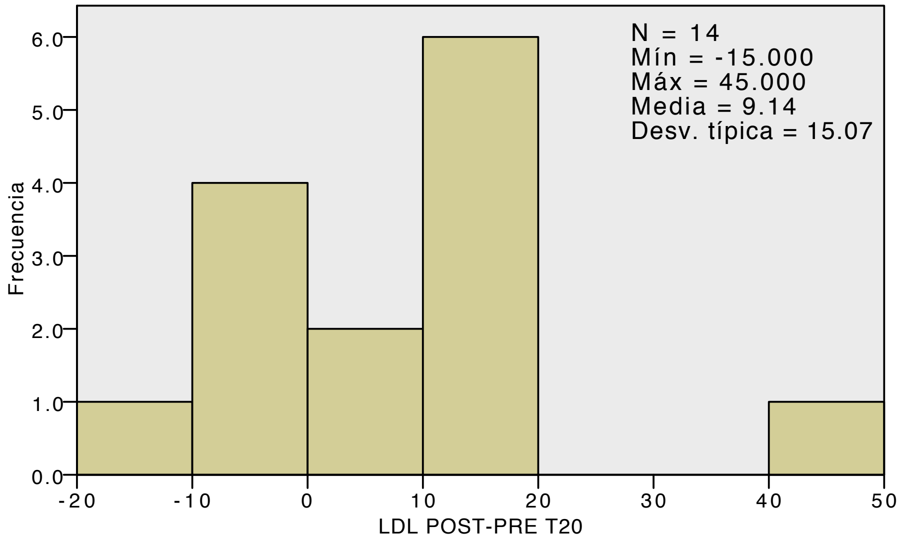


```
 *Nonparametric Tests: Related Samples.
NPTESTS
  /RELATED TEST(TAG.DIF.P TAG.DIF.T20) WILCOXON
  /MISSING SCOPE=ANALYSIS USERMISSING=EXCLUDE
  /CRITERIA ALPHA=0.05  CILEVEL=95.
```

Pruebas no paramétricas

Notas

Resultados creados		23-MAR-2019 18:42:28
Comentarios		
Entrada	Datos	/Users/SergioBarroso/Desktop/T20-RAL Reviewers/Base de datos/RAL.sav
	Conjunto de datos activo	Conjunto_de_datos2
	Filtro	<ninguno>
	Peso	<ninguno>
	Dividir archivo	<ninguno>
	Núm. de filas del archivo de trabajo	14
Sintaxis		NPTESTS /RELATED TEST(TAG.DIF.P TAG.DIF.T20) WILCOXON /MISSING SCOPE=ANALYSIS USERMISSING=EXCLUDE /CRITERIA ALPHA=0.05 CILEVEL=95.
Recursos	Tiempo de procesador	00:00:00.07
	Tiempo transcurrido	00:00:00.00

```
[Conjunto_de_datos2] /Users/SergioBarroso/Desktop/T20-RAL Reviewers/Base de datos/RAL.sav
```

Resumen de prueba de hipótesis

	Hipótesis nula	Test	Sig.	Decisión
1	La mediana de las diferencias entre TAG POST-PRE Placebo y TAG POST-PRE T20 es igual a 0.	Prueba de Wilcoxon de los rangos con signo de muestras relacionadas	.397	Retener la hipótesis nula.

Se muestran las significancias asintóticas. El nivel de significancia es .05.

Prueba de Wilcoxon de los rangos con signo de muestras relacionadas

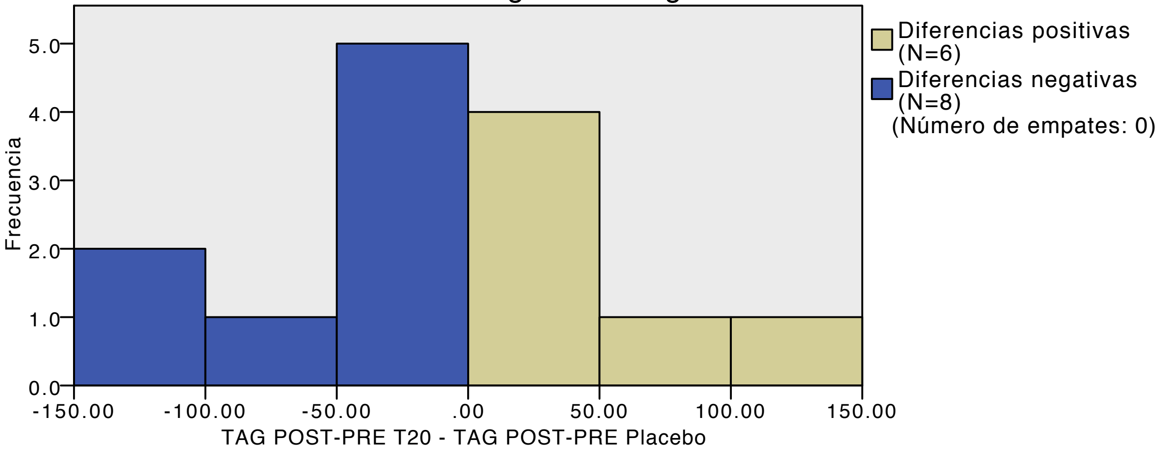

N total	14
Probar estadística	39.000
Error típico	15.930
Estadística de prueba estandarizada	-.847
Sig. asintótica (prueba de dos caras)	.397

Información de campo continuo

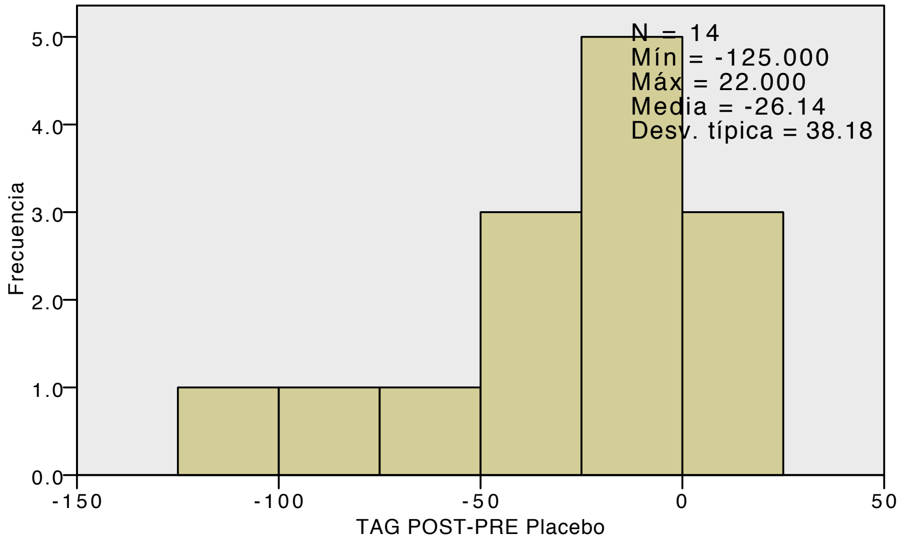

Información de campo continuo

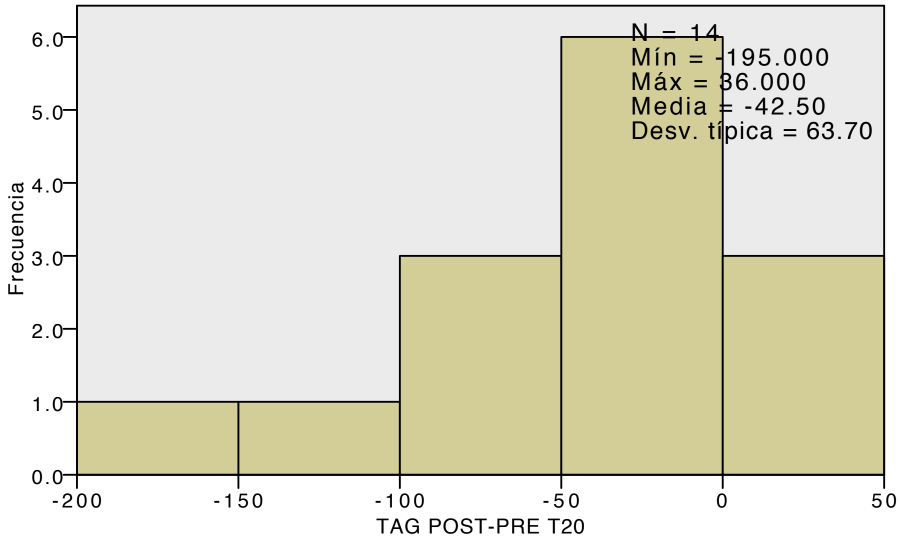


```
 *Nonparametric Tests: Related Samples.
NPTESTS
  /RELATED TEST(GLU.DIF.P GLU.DIF.T20) WILCOXON
  /MISSING SCOPE=ANALYSIS USERMISSING=EXCLUDE
  /CRITERIA ALPHA=0.05  CILEVEL=95.
```

Pruebas no paramétricas

Notas

Resultados creados		23-MAR-2019 18:42:44
Comentarios		
Entrada	Datos	/Users/SergioBarroso/Desktop/T20-RAL Reviewers/Base de datos/RAL.sav
	Conjunto de datos activo	Conjunto_de_datos2
	Filtro	<ninguno>
	Peso	<ninguno>
	Dividir archivo	<ninguno>
	Núm. de filas del archivo de trabajo	14
Sintaxis		NPTESTS<br>/RELATED TEST(GLU.DIF.P GLU.DIF.T20) WILCOXON<br>/MISSING SCOPE=ANALYSIS USERMISSING=EXCLUDE<br>/CRITERIA ALPHA=0.05 CILEVEL=95.
Recursos	Tiempo de procesador	00:00:00.05
	Tiempo transcurrido	00:00:00.00

```
[Conjunto_de_datos2] /Users/SergioBarroso/Desktop/T20-RAL Reviewers/Base de datos/RAL.sav
```

Resumen de prueba de hipótesis

	Hipótesis nula	Test	Sig.	Decisión
1	La mediana de las diferencias entre GLU POST-PRE Placebo y GLU POST-PRE T20 es igual a 0.	Prueba de Wilcoxon de los rangos con signo de muestras relacionadas	.157	Retener la hipótesis nula.

Se muestran las significancias asintóticas. El nivel de significancia es .05.

Prueba de Wilcoxon de los rangos con signo de muestras relacionadas

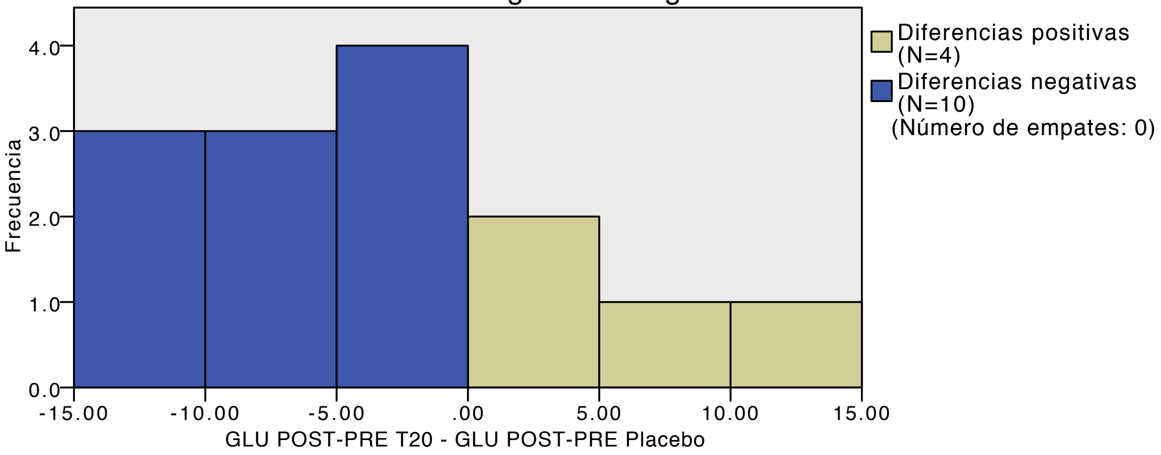

N total	14
Probar estadística	30.000
Error típico	15.890
Estadística de prueba estandarizada	-1.416
Sig. asintótica (prueba de dos caras)	.157

Información de campo continuo

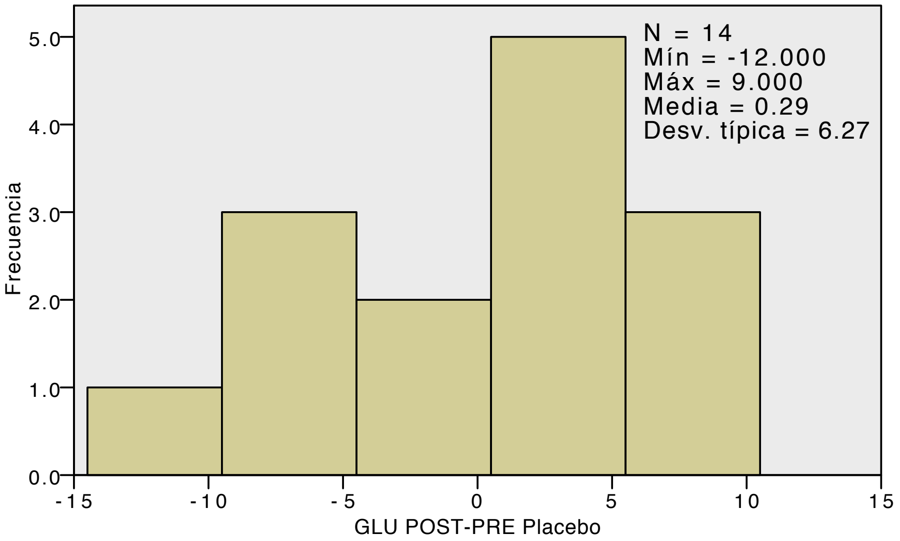

Información de campo continuo

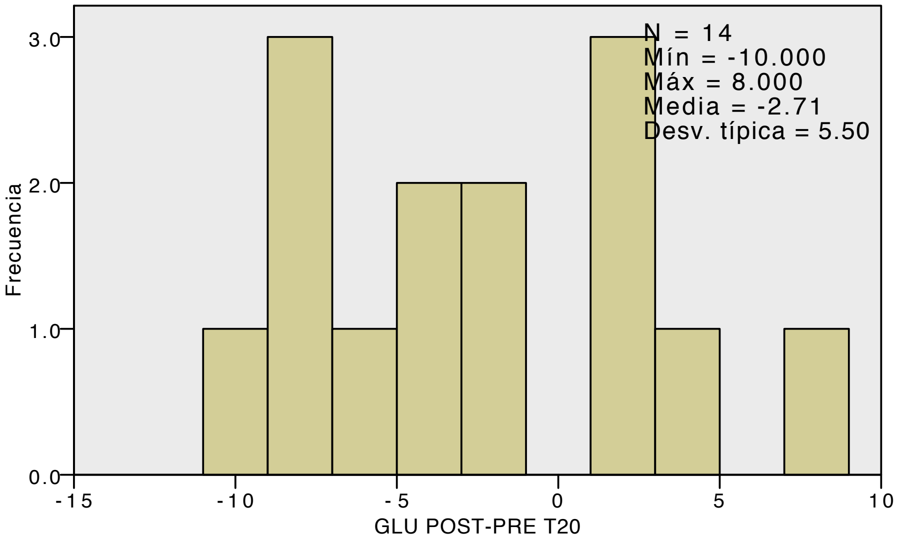


```
 *Nonparametric Tests: Related Samples.
NPTESTS
  /RELATED TEST(mtDNA.DIF.P mtDNA.DIF.T20) WILCOXON
  /MISSING SCOPE=ANALYSIS USERMISSING=EXCLUDE
  /CRITERIA ALPHA=0.05  CILEVEL=95.
```

Pruebas no paramétricas

Notas

**Resultados creados**		**23-MAR-2019 18:43:02**
**Comentarios**		
**Entrada**	**Datos**	**/Users/SergioBarroso/Desktop/T20-RAL Reviewers/Base de datos/RAL.sav**
	**Conjunto de datos activo**	**Conjunto_de_datos2**
	**Filtro**	**<ninguno>**
	**Peso**	**<ninguno>**
	**Dividir archivo**	**<ninguno>**
	**Núm. de filas del archivo de trabajo**	**14**
**Sintaxis**		**NPTESTS /RELATED TEST (mtDNA.DIF.P mtDNA.DIF.T20) WILCOXON /MISSING SCOPE=ANALYSIS USERMISSING=EXCLUDE /CRITERIA ALPHA=0.05 CILEVEL=95.**
**Recursos**	**Tiempo de procesador**	**00:00:00.06**
	**Tiempo transcurrido**	**00:00:00.00**

[Conjunto_de_datos2] /Users/SergioBarroso/Desktop/T20-RAL Reviewers/Base de datos/RAL.sav

Resumen de prueba de hipótesis

	Hipótesis nula	Test	Sig.	Decisión
1	La mediana de las diferencias entre mtDNA POST-PRE Placebo y mtDNA POST-PRE T20 es igual a 0.	Prueba de Wilcoxon de los rangos con signo de muestras relacionadas	.683	Retener la hipótesis nula.

Se muestran las significancias asintóticas. El nivel de significancia es .05.

Prueba de Wilcoxon de los rangos con signo de muestras relacionadas

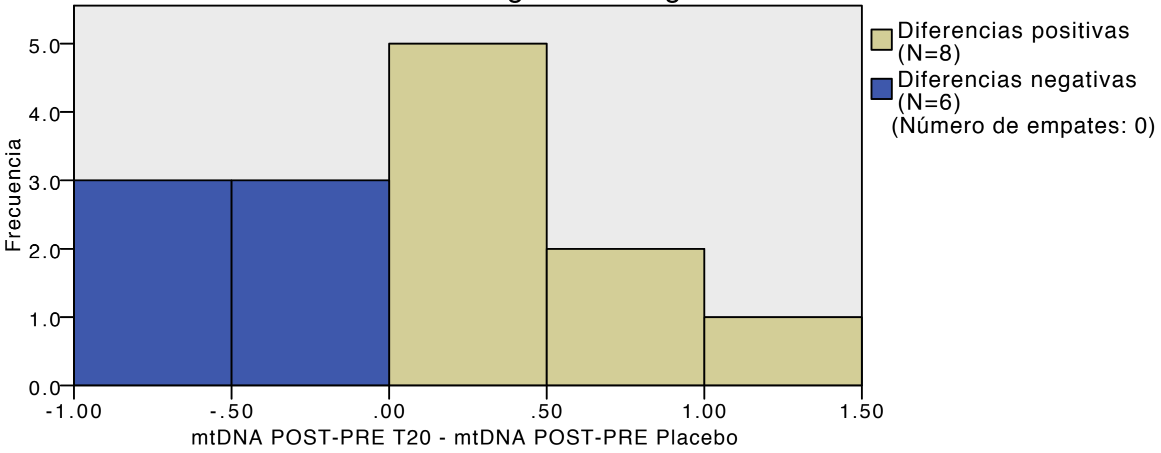

N total	14
Probar estadística	59.000
Error típico	15.926
Estadística de prueba estandarizada	.408
Sig. asintótica (prueba de dos caras)	.683

Información de campo continuo

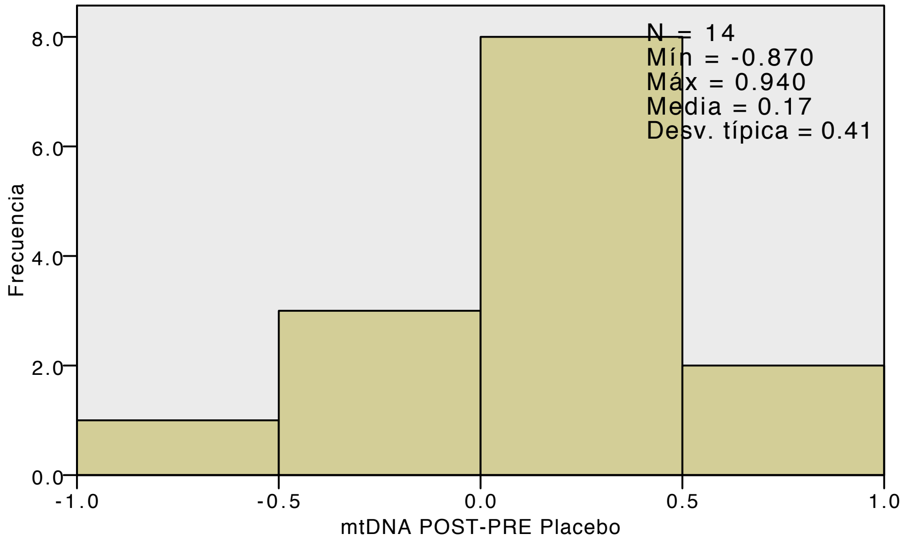

Información de campo continuo

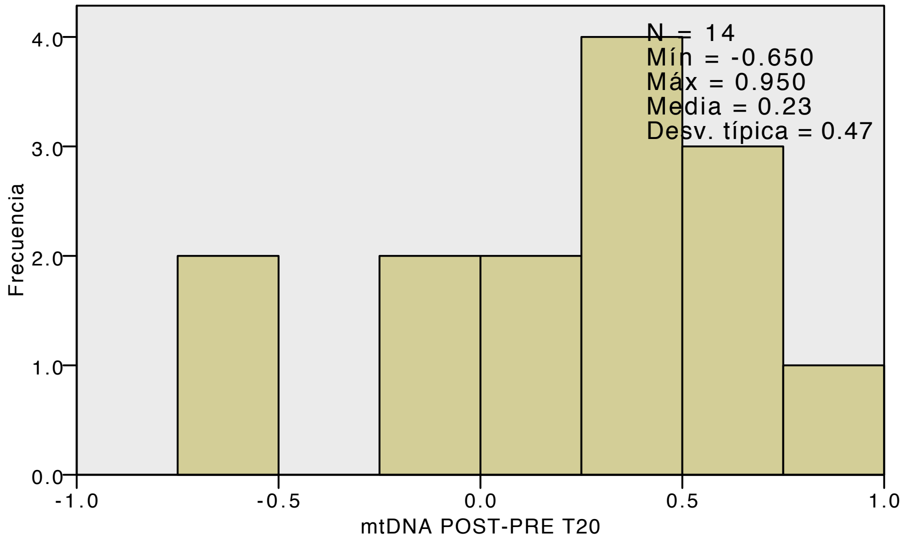


```
 *Nonparametric Tests: Related Samples.
NPTESTS
  /RELATED TEST(CRT.DIF.P CRT.DIF.T20) WILCOXON
  /MISSING SCOPE=ANALYSIS USERMISSING=EXCLUDE
  /CRITERIA ALPHA=0.05  CILEVEL=95.
```

Pruebas no paramétricas

Notas

**Resultados creados**		**23-MAR-2019 18:43:16**
**Comentarios**		
**Entrada**	**Datos**	**/Users/SergioBarroso/Desktop/T20-RAL Reviewers/Base de datos/RAL.sav**
	**Conjunto de datos activo**	**Conjunto_de_datos2**
	**Filtro**	**<ninguno>**
	**Peso**	**<ninguno>**
	**Dividir archivo**	**<ninguno>**
	**Núm. de filas del archivo de trabajo**	**14**
**Sintaxis**		**NPTESTS /RELATED TEST(CRT.DIF.P CRT.DIF.T20) WILCOXON /MISSING SCOPE=ANALYSIS USERMISSING=EXCLUDE /CRITERIA ALPHA=0.05 CILEVEL=95.**
**Recursos**	**Tiempo de procesador**	**00:00:00.07**
	**Tiempo transcurrido**	**00:00:00.00**

```
[Conjunto_de_datos2] /Users/SergioBarroso/Desktop/T20-RAL Reviewers/Base de datos/RAL.sav
```

Resumen de prueba de hipótesis

	Hipótesis nula	Test	Sig.	Decisión
1	La mediana de las diferencias entre CRT POST-PRE Placebo y CRT POST-PRE T20 es igual a 0.	Prueba de Wilcoxon de los rangos con signo de muestras relacionadas	.693	Retener la hipótesis nula.

Se muestran las significancias asintóticas. El nivel de significancia es .05.

Prueba de Wilcoxon de los rangos con signo de muestras relacionadas

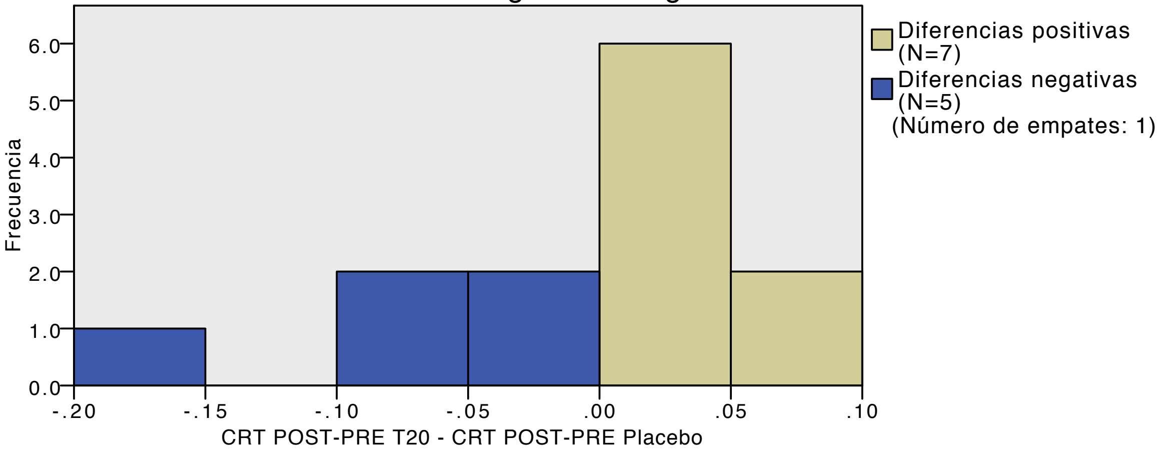

N total	13
Probar estadística	34.000
Error típico	12.674
Estadística de prueba estandarizada	-.395
Sig. asintótica (prueba de dos caras)	.693

Información de campo continuo

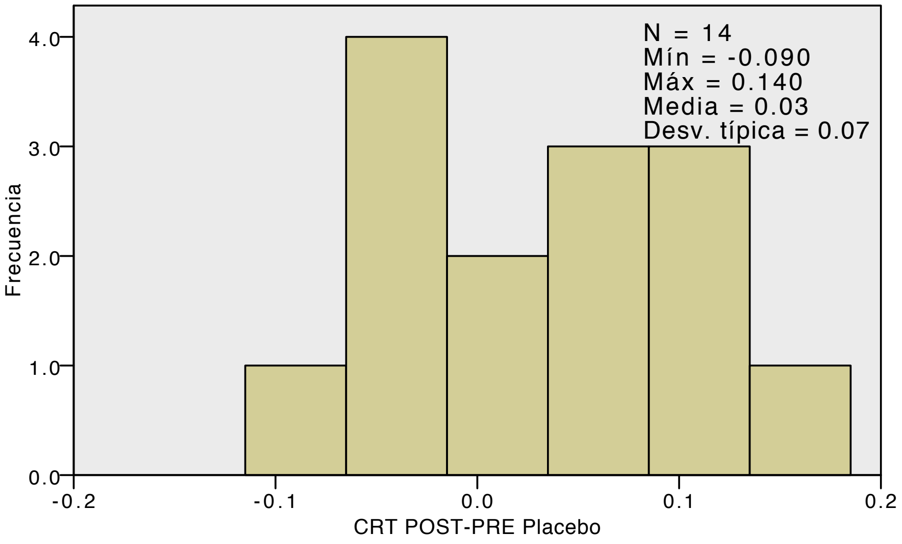

Información de campo continuo

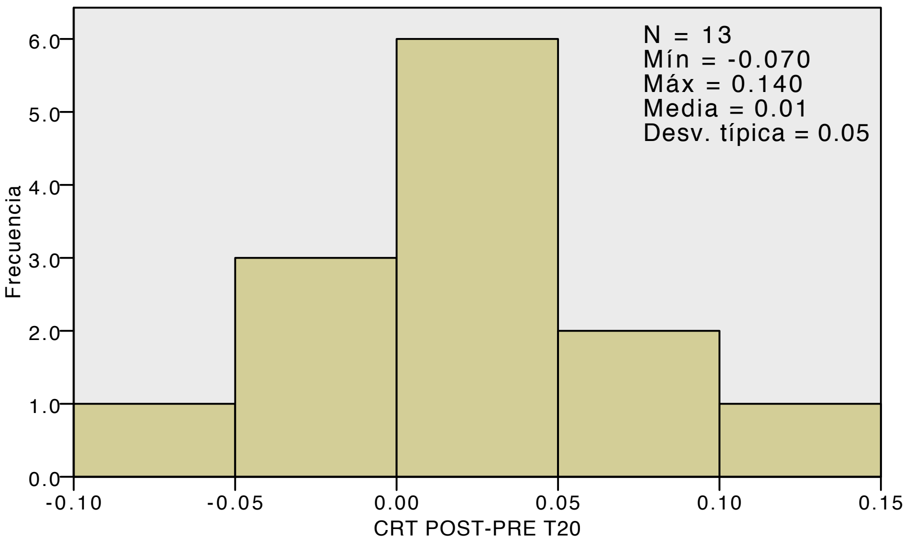


```
 *Nonparametric Tests: Related Samples.
NPTESTS
  /RELATED TEST(AST.DIF.P AST.DIF.T20) WILCOXON
  /MISSING SCOPE=ANALYSIS USERMISSING=EXCLUDE
  /CRITERIA ALPHA=0.05  CILEVEL=95.
```

Pruebas no paramétricas

Notas

**Resultados creados**		**23-MAR-2019 18:43:35**
**Comentarios**		
**Entrada**	**Datos**	**/Users/SergioBarroso/Desktop/T20-RAL Reviewers/Base de datos/RAL.sav**
	**Conjunto de datos activo**	**Conjunto_de_datos2**
	**Filtro**	**<ninguno>**
	**Peso**	**<ninguno>**
	**Dividir archivo**	**<ninguno>**
	**Núm. de filas del archivo de trabajo**	**14**
**Sintaxis**		**NPTESTS /RELATED TEST(AST.DIF.P AST.DIF.T20) WILCOXON /MISSING SCOPE=ANALYSIS USERMISSING=EXCLUDE /CRITERIA ALPHA=0.05 CILEVEL=95.**
**Recursos**	**Tiempo de procesador**	**00:00:00.06**
	**Tiempo transcurrido**	**00:00:00.00**

```
[Conjunto_de_datos2] /Users/SergioBarroso/Desktop/T20-RAL Reviewers/Base de datos/RAL.sav
```

Resumen de prueba de hipótesis

	Hipótesis nula	Test	Sig.	Decisión
1	La mediana de las diferencias entre AST POST-PRE Placebo y AST POST-PRE T20 es igual a 0.	Prueba de Wilcoxon de los rangos con signo de muestras relacionadas	.593	Retener la hipótesis nula.

Se muestran las significancias asintóticas. El nivel de significancia es .05.

Prueba de Wilcoxon de los rangos con signo de muestras relacionadas

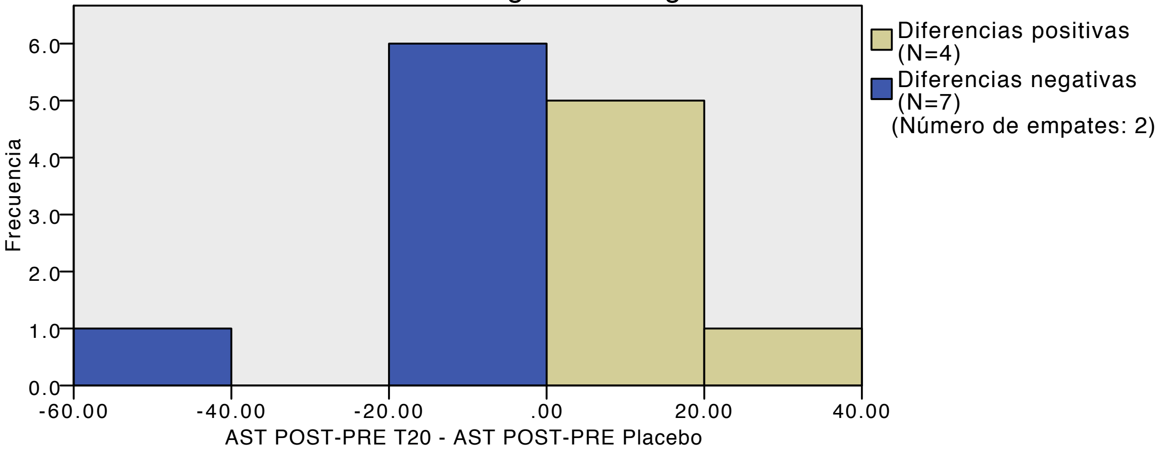

N total	13
Probar estadística	27.000
Error típico	11.231
Estadística de prueba estandarizada	-.534
Sig. asintótica (prueba de dos caras)	.593

Información de campo continuo

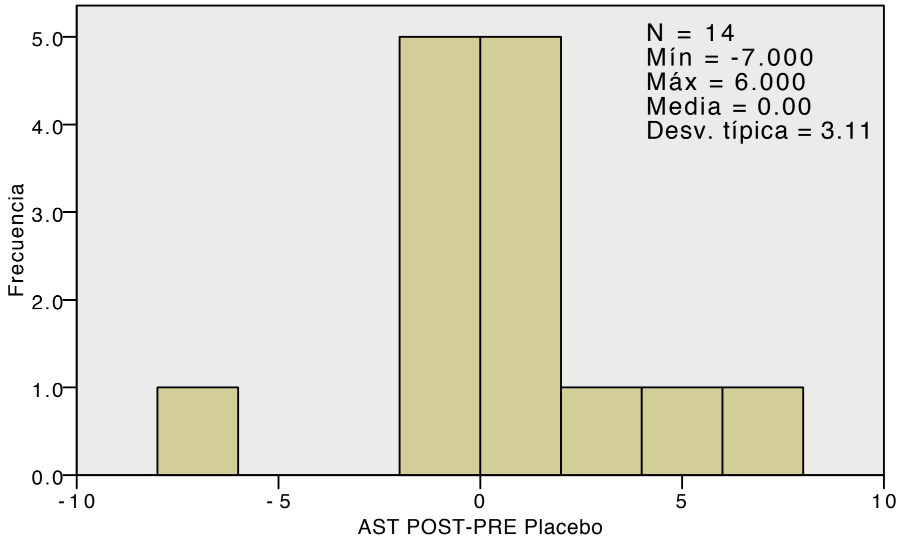

Información de campo continuo

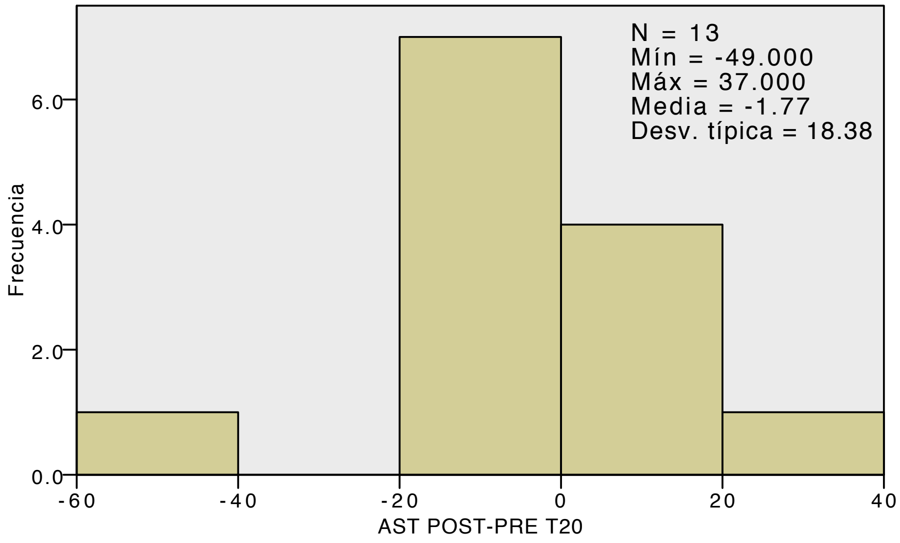


```
 *Nonparametric Tests: Related Samples.
NPTESTS
  /RELATED TEST(ALT.DIF.P ALT.DIF.T20) WILCOXON
  /MISSING SCOPE=ANALYSIS USERMISSING=EXCLUDE
  /CRITERIA ALPHA=0.05  CILEVEL=95.
```

Pruebas no paramétricas

Notas

**Resultados creados**		**23-MAR-2019 18:43:55**
**Comentarios**		
**Entrada**	**Datos**	**/Users/SergioBarroso/Desktop/T20-RAL Reviewers/Base de datos/RAL.sav**
	**Conjunto de datos activo**	**Conjunto_de_datos2**
	**Filtro**	**<ninguno>**
	**Peso**	**<ninguno>**
	**Dividir archivo**	**<ninguno>**
	**Núm. de filas del archivo de trabajo**	**14**
**Sintaxis**		**NPTESTS /RELATED TEST(ALT.DIF.P ALT.DIF.T20) WILCOXON /MISSING SCOPE=ANALYSIS USERMISSING=EXCLUDE /CRITERIA ALPHA=0.05 CILEVEL=95.**
**Recursos**	**Tiempo de procesador**	**00:00:00.07**
	**Tiempo transcurrido**	**00:00:00.00**

```
[Conjunto_de_datos2] /Users/SergioBarroso/Desktop/T20-RAL Reviewers/Base de datos/RAL.sav
```

Resumen de prueba de hipótesis

	Hipótesis nula	Test	Sig.	Decisión
1	La mediana de las diferencias entre ALT POST-PRE Placebo y ALT POST-PRE T20 es igual a 0.	Prueba de Wilcoxon de los rangos con signo de muestras relacionadas	.345	Retener la hipótesis nula.

Se muestran las significancias asintóticas. El nivel de significancia es .05.

Prueba de Wilcoxon de los rangos con signo de muestras relacionadas

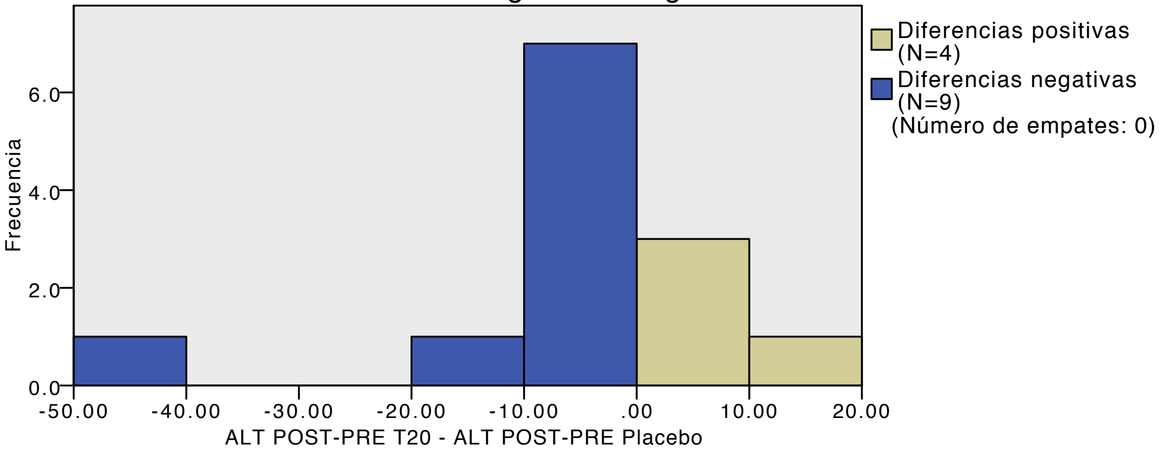

N total	13
Probar estadística	32.000
Error típico	14.292
Estadística de prueba estandarizada	-.945
Sig. asintótica (prueba de dos caras)	.345

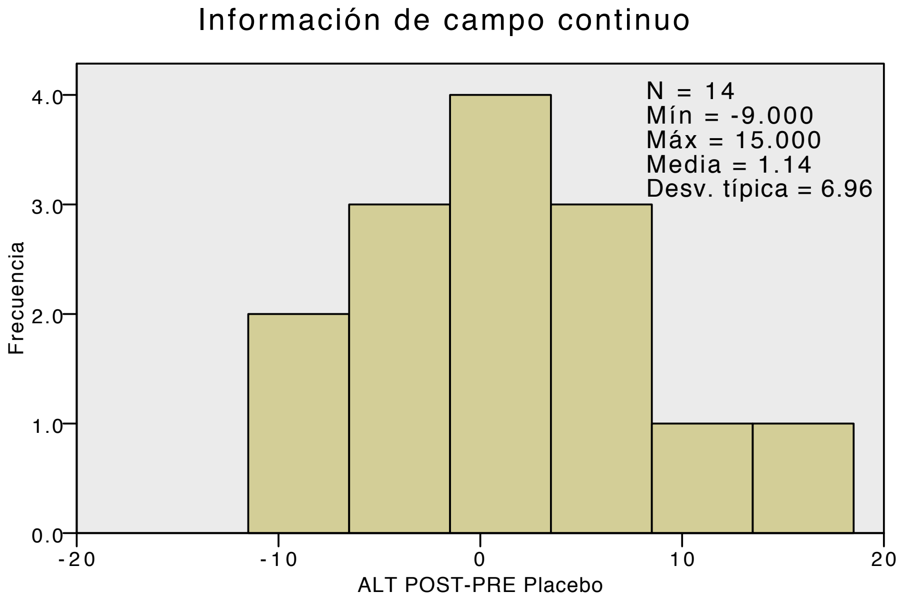

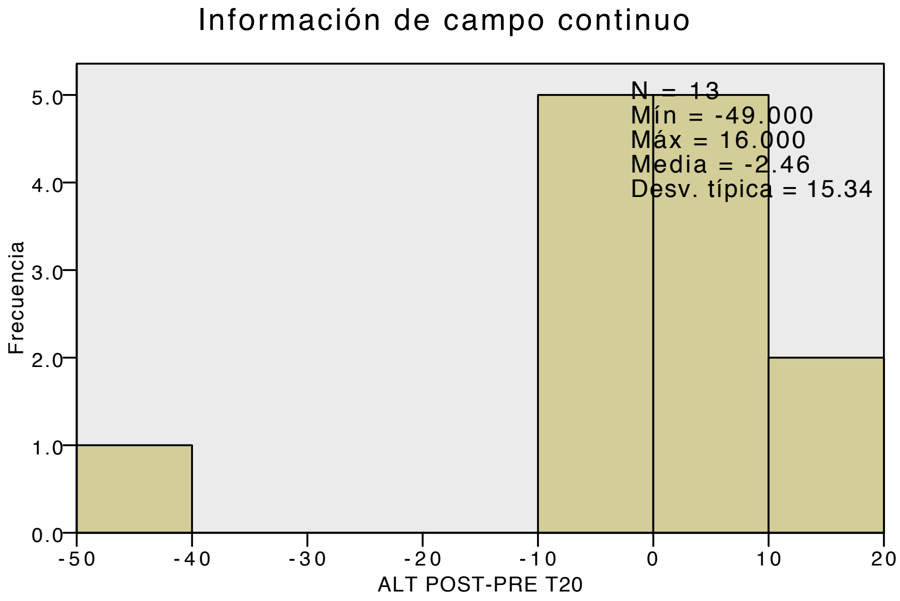


```
 *Nonparametric Tests: Related Samples.
NPTESTS
  /RELATED TEST(BIL.DIF.P BIL.DIF.T20) WILCOXON
  /MISSING SCOPE=ANALYSIS USERMISSING=EXCLUDE
  /CRITERIA ALPHA=0.05  CILEVEL=95.
```

Pruebas no paramétricas

Notas

Resultados creados		23-MAR-2019 18:44:15
Comentarios		
Entrada	Datos	/Users/SergioBarroso/Desktop/T20-RAL Reviewers/Base de datos/RAL.sav
	Conjunto de datos activo	Conjunto_de_datos2
	Filtro	<ninguno>
	Peso	<ninguno>
	Dividir archivo	<ninguno>
	Núm. de filas del archivo de trabajo	14
Sintaxis		NPTESTS<br>/RELATED TEST(BIL.DIF.P BIL.DIF.T20) WILCOXON<br>/MISSING SCOPE=ANALYSIS USERMISSING=EXCLUDE<br>/CRITERIA ALPHA=0.05 CILEVEL=95.
Recursos	Tiempo de procesador	00:00:00.07
	Tiempo transcurrido	00:00:00.00

[Conjunto_de_datos2] /Users/SergioBarroso/Desktop/T20-RAL Reviewers/Base de datos/RAL.sav

Resumen de prueba de hipótesis

	Hipótesis nula	Test	Sig.	Decisión
1	La mediana de las diferencias entre BIL POST-PRE Placebo y BIL POST-PRE T20 es igual a 0.	Prueba de Wilcoxon de los rangos con signo de muestras relacionadas	.929	Retener la hipótesis nula.

Se muestran las significancias asintóticas. El nivel de significancia es .05.

Prueba de Wilcoxon de los rangos con signo de muestras relacionadas

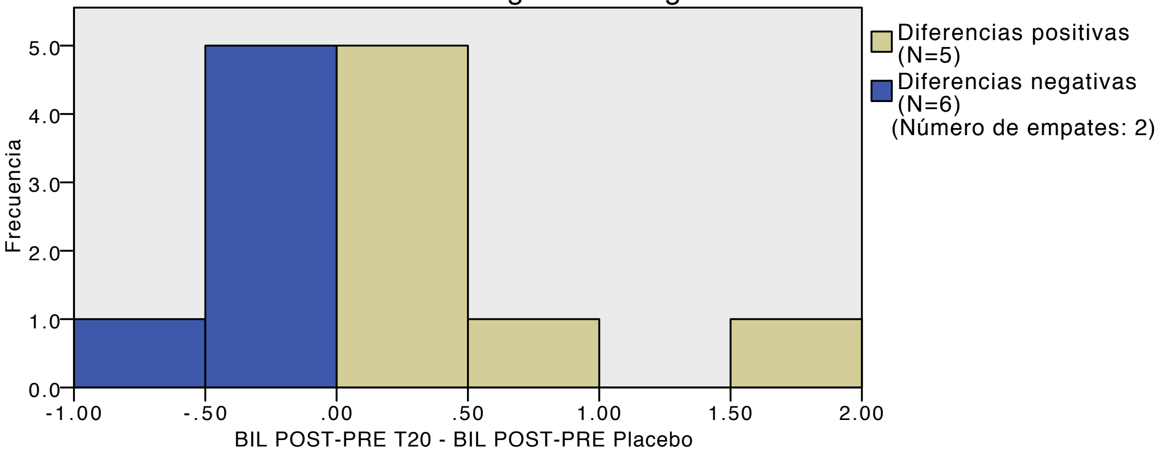

N total	13
Probar estadística	32.000
Error típico	11.214
Estadística de prueba estandarizada	-.089
Sig. asintótica (prueba de dos caras)	.929

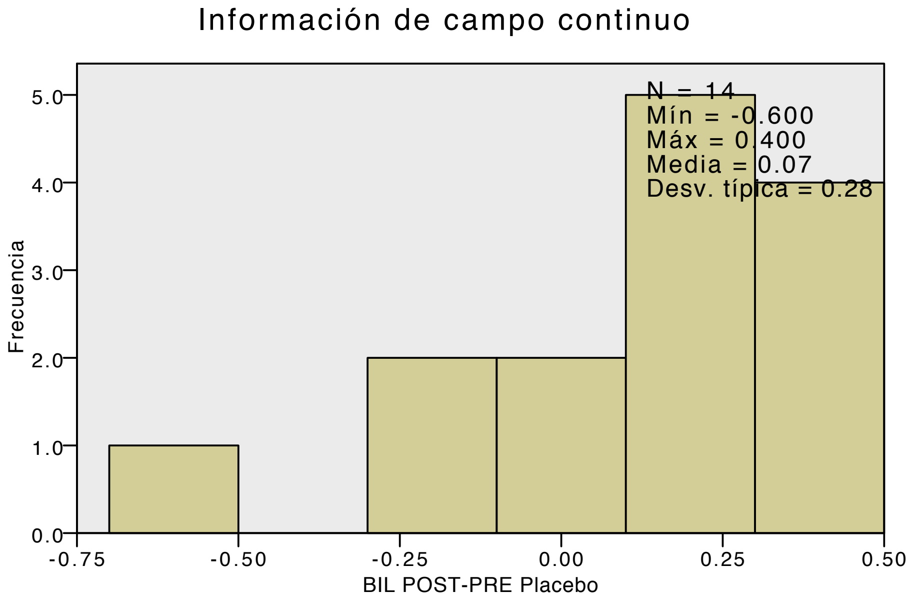

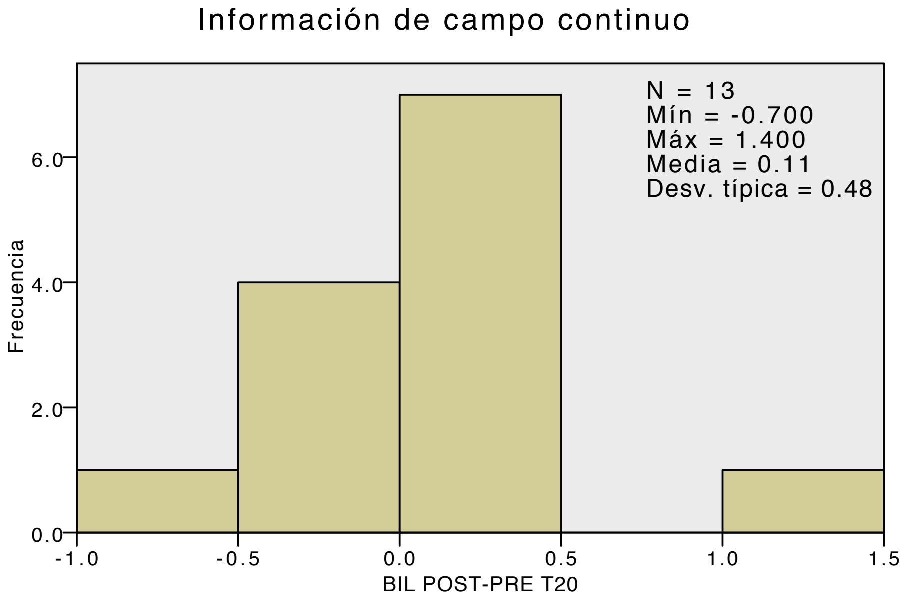


```
 *Nonparametric Tests: Related Samples.
NPTESTS
  /RELATED TEST(PROT.DIF.P PROT.DIF.T20) WILCOXON
  /MISSING SCOPE=ANALYSIS USERMISSING=EXCLUDE
  /CRITERIA ALPHA=0.05  CILEVEL=95.
```

Pruebas no paramétricas

Notas

Resultados creados		23-MAR-2019 18:44:40
Comentarios		
Entrada	Datos	/Users/SergioBarroso/Desktop/T20-RAL Reviewers/Base de datos/RAL.sav
	Conjunto de datos activo	Conjunto_de_datos2
	Filtro	<ninguno>
	Peso	<ninguno>
	Dividir archivo	<ninguno>
	Núm. de filas del archivo de trabajo	14
Sintaxis		NPTESTS /RELATED TEST(PROT.DIF.P PROT.DIF.T20) WILCOXON /MISSING SCOPE=ANALYSIS USERMISSING=EXCLUDE /CRITERIA ALPHA=0.05 CILEVEL=95.
Recursos	Tiempo de procesador	00:00:00.06
	Tiempo transcurrido	00:00:00.00

```
[Conjunto_de_datos2] /Users/SergioBarroso/Desktop/T20-RAL Reviewers/Base de datos/RAL.sav
```

Resumen de prueba de hipótesis

	Hipótesis nula	Test	Sig.	Decisión
1	La mediana de las diferencias entre PROT POST-PRE Placebo y PROT POST-PRE T20 es igual a 0.	Prueba de Wilcoxon de los rangos con signo de muestras relacionadas	.086	Retener la hipótesis nula.

Se muestran las significancias asintóticas. El nivel de significancia es .05.

Prueba de Wilcoxon de los rangos con signo de muestras relacionadas

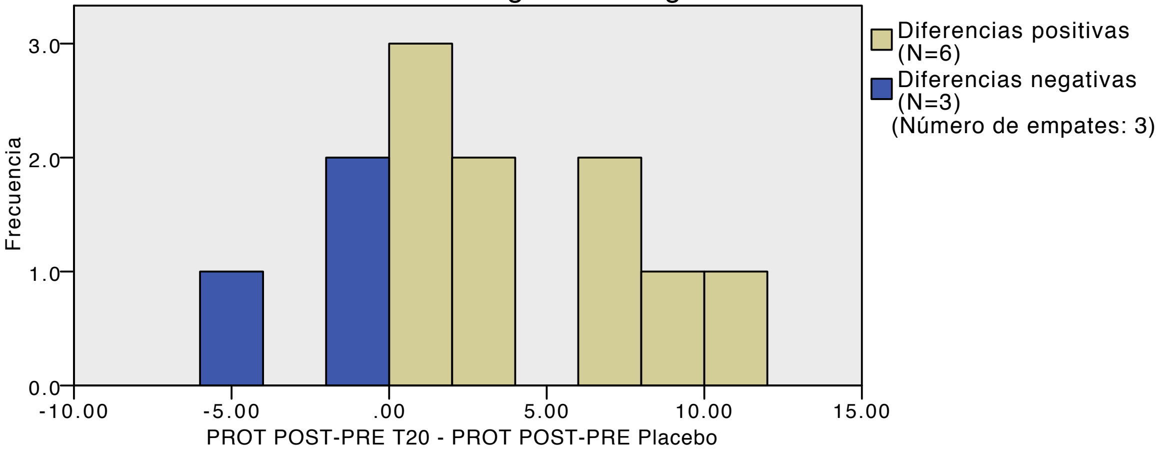

N total	12
Probar estadística	37.000
Error típico	8.434
Estadística de prueba estandarizada	1.719
Sig. asintótica (prueba de dos caras)	.086

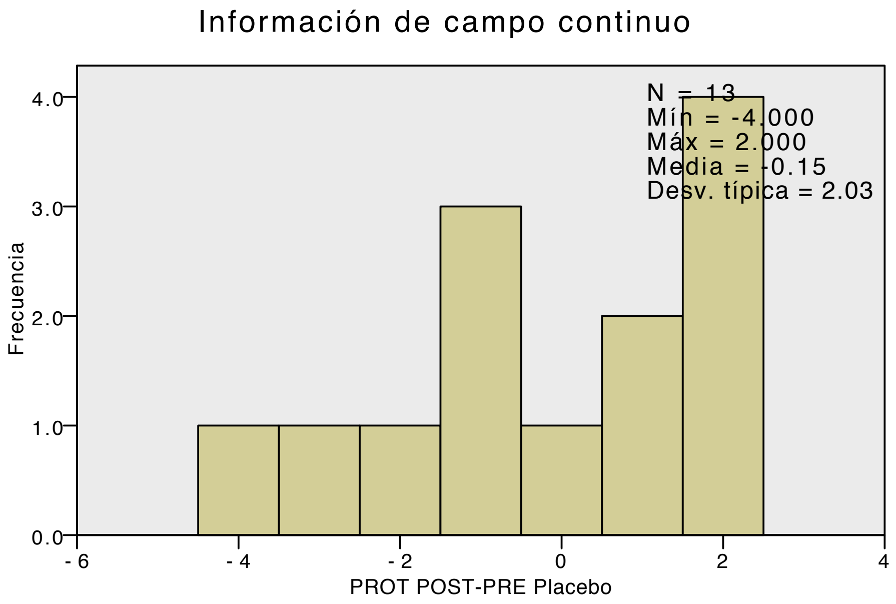
Información de campo continuo
N = 13
Mín = -4.000
Máx = 2.000
Media = -0.15
Desv. típica = 2.03
Frecuencia
4.0
3.0
2.0
1.0
0.0
- 6
- 4
- 2
0
2
4
PROT POST-PRE Placebo

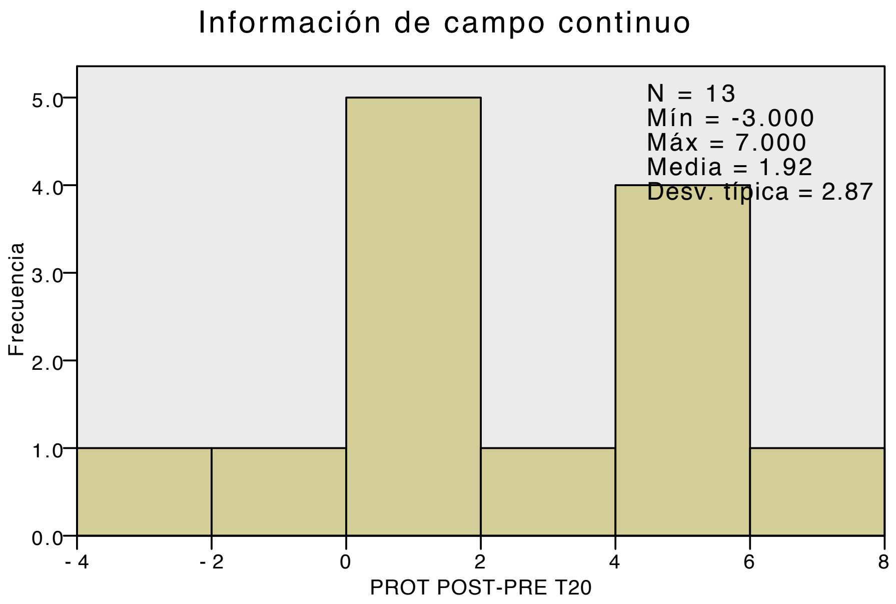
Información de campo continuo
N = 13
Mín = -3.000
Máx = 7.000
Media = 1.92
Desv. típica = 2.87
Frecuencia
5.0
4.0
3.0
2.0
1.0
0.0
- 4
- 2
0
2
4
6
8
PROT POST-PRE T20
